# A Comparative Proteomic Analysis Reveals a New Bi-Lobe Protein Required for Bi-Lobe Duplication and Cell Division in *Trypanosoma brucei*

**DOI:** 10.1371/journal.pone.0009660

**Published:** 2010-03-15

**Authors:** Qing Zhou, Ladan Gheiratmand, Yixin Chen, Teck Kwang Lim, Jun Zhang, Shaowei Li, Ningshao Xia, Binghai Liu, Qingsong Lin, Cynthia Y. He

**Affiliations:** 1 Department of Biological Sciences, National University of Singapore, Singapore, Singapore; 2 School of Life Sciences, Xiamen University, Xiamen, China; Texas A&M University, United States of America

## Abstract

A Golgi-associated bi-lobed structure was previously found to be important for Golgi duplication and cell division in *Trypanosoma brucei*. To further understand its functions, comparative proteomics was performed on extracted flagellar complexes (including the flagellum and flagellum-associated structures such as the basal bodies and the bi-lobe) and purified flagella to identify new bi-lobe proteins. A leucine-rich repeats containing protein, TbLRRP1, was characterized as a new bi-lobe component. The anterior part of the TbLRRP1-labeled bi-lobe is adjacent to the single Golgi apparatus, and the posterior side is tightly associated with the flagellar pocket collar marked by TbBILBO1. Inducible depletion of TbLRRP1 by RNA interference inhibited duplication of the bi-lobe as well as the adjacent Golgi apparatus and flagellar pocket collar. Formation of a new flagellum attachment zone and subsequent cell division were also inhibited, suggesting a central role of bi-lobe in Golgi, flagellar pocket collar and flagellum attachment zone biogenesis.

## Introduction

*Trypanosoma brucei* is a parasitic pathogen causing sleeping sickness in human and Nagana in cattle, both imposing major economic burdens in Sub-Saharan Africa [1]. This single-celled parasite contains a single nucleus, a single kinetoplast (aggregate of mitochondrial DNA), a single Golgi apparatus and a single flagellum which exits the cell through an adhesion zone named flagellar pocket collar (FPC) and remains attached to the cell body via a cytoplasmic structure known as the flagellum attachment zone (FAZ). Each single-copy organelle occupies a defined cellular location, and duplicates and segregates in a highly ordered sequence during the cell cycle [2]–[4]. The division of the duplicated kinetoplasts, Golgi and ER exit sites are all linked to the segregation of the duplicated basal bodies, which are located at the base of the flagellum and seed the growth of the microtubular axoneme. While the co-ordinated division between kinetoplast and basal bodies is mediated by a tripartite attachment complex (TAC) that physically links the basal bodies to the kinetoplast DNA [5], the co-ordinated duplication between Golgi/ER exit sites and the basal bodies appears to be mediated by a bi-lobed structure, which duplicates and segregates synchronously with the basal bodies [6].

The bi-lobed structure was first discovered using a pantopic antibody against centrins [6], which are highly conserved calcium-binding proteins frequently found associated with microtubule organizing centers and required for their duplication [7], [8]. Both TbCentrin2 and TbCentrin4 (also known as TbCen1 [9]) are found localized to both the bi-lobed structure and the basal bodies in *T. brucei*. While depletion of TbCentrin2 inhibits the duplication/segregation of basal bodies, Golgi, kinetoplast, FAZ and final cell division, depletion of TbCentrin4 did not affect the duplication of organelles, but did disrupt the timing between nuclear division and the subsequent cytokinesis through an unknown mechanism [10].

In addition to the centrins, a polo-like kinase, TbPLK1 and a MORN- (Membrane Occupation and Recognition Nexus) containing protein, TbMORN1, were recently identified to be on the bi-lobe [11], [12]. TbPLK1 was transiently associated with the bi-lobe at the time of its duplication and later found at the tip of the new, elongating FAZ. Absence of TbPLK1 resulted in malformed bi-lobed structures and an increase in the number of duplicated Golgi [11]. Cell division was also affected when TbPLK1 was depleted or over-expressed [11], [13]. Knockdown of TbMORN1 had a moderate inhibitory effect on cell growth in procyclic (the insect stage) *T. brucei*, but was lethal in the bloodstream stage parasite [12].

The association of the bi-lobe with centrins and the polo-like kinase, which are key regulators of cell cycle progress in yeast and mammalian cells [8], [14], suggests a critical function for this structure in the *T. brucei* cell cycle. To gain further functional insights, we have used a comparative proteomics approach to identify new protein components on the bi-lobe and have identified a leucine-rich repeat protein, TbLRRP1. Further characterization of TbLRRP1 revealed a tight association between bi-lobe and FPC, and an essential role of TbLRRP1 in bi-lobe, FPC and Golgi duplication. TbLRRP1 depletion also led to defects in parasite motility, new FAZ formation and subsequent cell division.

## Materials and Methods

### Cell lines

Procyclic form *T. brucei* (*T. brucei rhodesiense*), YTat1.1 cells [15] were maintained in Cunningham medium containing 15% heat-inactivated fetal bovine serum (BD Biosciences) at 28°C and used for cell fractionation, recombinant expression and endogenous replacement with YFP-tagged fusions. Procyclic 29.13 cell line [16] (*T. brucei brucei*), which was genetically modified to express T7 RNA polymerase and the tetracycline repressor, was used for inducible RNA interference (RNAi) studies. The 29.13 cells were maintained in Cunningham medium containing 15% heat-inactivated, tetracycline-free fetal bovine serum (Clonetech) in the presence of 15 µg/ml G418 and 50 µg/ml hygromycin at 28°C.

### Preparation of flagellum and flagellar complex

The flagellar complex was prepared using a two-step extraction method previously described [17], [18]. Sequential extractions of whole parasite cells (3×10^9^), using PEM buffer (100 mM PIPES, 1 mM EGTA, 1 mM MgSO_4_, pH 6.9) containing 1% NP-40 and 1 M KCl, removed most cellular components, leaving behind only the core protein components of the flagellum, and organelles tightly associated with the flagellum such as the basal bodies and the bi-lobed structure [12].

Flagellum free of basal bodies and bi-lobes was purified using a procedure described previously [19] with the following modification. Briefly, 3×10^9^ procyclic cells were harvested, washed and resuspended in homogenization buffer containing 0.32 M sucrose, 25 mM Tris HCl, pH 7.4, 5 mM MgCl_2_, 0.1 mM CaCl_2_, 0.2 mM EDTA, 12 mM beta-mercaptoethanol and 1% bovine serum albumin. The cells were sonicated for 5 cycles with 20 s pulse and 40 s rest, at 20% maximum output (130 W) to detach flagella from intact cell bodies. The homogenate was centrifuged at 800 g for 15 min to remove unbroken cells. The supernatant containing the detached flagella was then centrifuged at 6900 g for 15 min in a JA20 rotor pre-chilled to 4°C. The precipitated flagella were resuspended in homogenization buffer, loaded on top of a 6 ml 0.61 M sucrose cushion, and centrifuged at 850 g for 15 min in a SW41 rotor. The top layer and the interface containing the detached flagella were then loaded on top of a step sucrose gradient containing 2 ml each of 2 M, 1.84 M, 1.66 M and 1.5 M sucrose, and centrifuged at 75,000 g for 16 hours in a SW41 rotor. Highly enriched flagella were collected from the 1.66 M/1.84 M interface and sequentially extracted with 1% NP-40 and 1 M KCl made in PEM buffer.

### iTRAQ labeling and liquid chromatography

Samples containing flagellar complexes and flagella only were quantified for protein concentration, denatured and the cysteines blocked as described in iTRAQ protocol (Applied Biosystems, CA). The protein samples were then digested with trypsin and labeled with iTRAQ reagents as follows: flagellum fraction with 114 and 116 reagents; flagellar complex with 115 and 117 reagents. The iTRAQ-labeled samples were pooled together, cleaned up using a cation exchange column, separated by reversed-phase high performance liquid chromatography and analyzed by ABI 4800 MALDI TOF/TOF MS/MS.

### Proteomic data processing and statistical analysis

GPS Explorer software version 3.6 (Applied Biosystems) employing MASCOT 2.1 search engine (Matrix Science) was used for protein identification. To minimize false positive identifications, a combined database with a total of 76708 entries that include all predicted amino acid sequences in the *Trypanosoma brucei* Genome Project (ftp://ftp.sanger.ac.uk/pub/databases/T.brucei_sequences/) as well as the International Protein Index Human database version 3.33 was used for the search. The searching parameters were as follows: cysteine methanethiolation, N-terminal iTRAQ labeling, and iTRAQ-labeled lysine were selected as fixed modifications; methionine oxidation was considered as a variable modification; precursor tolerance was set to 100 ppm; MS/MS fragment tolerance was set to 0.4 Da; maximum peptide rank was set to 1; the minimum ion score C. I. % was set to 1%. For all protein identifications, only those with the best ion score C.I. > = 95% were considered as acceptable identifications. For those proteins with only one matching peptide, the MS/MS spectra were manually inspected to validate the protein identifications (see Text S1). To validate the iTRAQ ratio of all protein identifications, *t* tests were performed and P values were calculated based on the null hypothesis that the observed iTRAQ ratio (115/114 and 117/114) is not different from a ratio of 1.0 (i.e. the protein is equally abundant in the two samples). Previous results showed that the standard deviation of iTRAQ ratio of the two identical iTRAQ-labeled samples was 0.15 for the MS system used in this study [20]. Therefore a 1.3-fold change could be used as the cutoff value for up-regulated proteins, and 0.77 as the cutoff threshold for down-regulated proteins. In this study, a more stringent cutoff threshold (2-fold change) was applied to ensure the detection of proteins with abundance differences. The abundance ratio was then calculated as Log2 (average iTRAQ ratio). A protein with an abundance ratio > = 1 was considered more abundant in the flagellar complex; < = −1 being more abundant in the flagellum fraction; <1 and >−1 being equally abundant.

### Bioinformatics methods

Position-Specific Iterated Blast (PSI-Blast) was performed against the non-redundant protein database (http://blast.ncbi.nlm.nih.gov/Blast.cgi) and proteome databases as described in Table S2 using the predicted full-length amino acid sequence of candidate proteins identified by comparative proteomics. Hits with E values below 10^−5^ were accepted as homologs. Annotation and ortholog information were also obtained from *Trypanosoma brucei* GeneDB (http://www.genedb.org/genedb/tryp).

### Plasmids construction and transfection

To study protein localization, the full-length coding sequence of each protein was amplified from genomic DNA by PCR and inserted into either 5′ or 3′- end of the yellow fluorescence protein (YFP) coding sequence cloned in the pXS2 vector [21]. For endogenous expression of TbLRRP1 (Tb11.01.0680) with YFP epitope fused to its N-terminus, a modified pCR4Blunt-TOPO vector was used [12]. 500 bp 5′-UTR sequence immediately upstream of the TbLRRP1 start codon was cloned between PacI and HindIII sites. A 500 bp fragment of the TbLRRP1-coding sequence immediately downstream of the start codon was cloned into BamHI and NsiI sites. For TbLRRP1-RNAi, an automated, web-based program was used to search for suitable RNAi target [22] (http://trypanofan.path.cam.ac.uk/software/RNAit.html). A 506 bp fragment specific to TbLRRP1 coding sequence (nucleotide 140–645) was amplified and cloned into the pZJM vector [23].

For stable transfections, 15 µg of linearized plasmid was transfected into YTat1.1 or 29.13 cells by electroporation (1500 V, 25 µF). Stable cell lines were selected with antibiotics at appropriate concentration.

### Growth assays

Log-phase culture was diluted to 2×10^5^ cells/ml to initiate the growth assays. Cell density was then measured every 24 hours using a hemocytometer. During this process, to maintain exponential growth conditions and avoid saturation, the cultures were diluted to 10^6^ cells/ml whenever the cell density exceeded 10^6^ cells/ml. The doubling number was calculated as follows: doubling number  =  log_2_ (N_t_×DF)/N_0_, where N_t_ was the cell density at each time point, N_0_ was the cell density at t = 0 (i.e. 2×10^5^), and DF was the dilution factor (calculated as the cell density before dilution divided by the cell density after dilution).

### Anti-TbLRRP1 antibody

A 699 bp fragment of TbLRRP1 coding sequence (aa480–713), which excluded the N-terminal hydrophobic leucine-rich repeats, was fused to 6xHis tag and expressed in *E. coli* strain BL21. The fusion protein was affinity purified in urea using Ni-NTA resin as described in User's Manual (Amersham Pharmacia Biotech), and then used to produce mouse monoclonal antibodies.

### Immunofluorescence analysis

Cells were attached to coverslips, fixed with methanol, blocked with 3% BSA in PBS and then incubated with corresponding antibodies. For labeling with anti-TbMORN1, the cells were extracted with 0.5% Nonidet P-40 in PEM, fixed with 4% paraformaldehyde and then processed for antibody labeling. Anti-TbGRASP [3], L3B2 [24], YL1/2 [25], anti-TbBILBO1 [4], anti-TbMORN1 [12], and anti-TbCentrin4 [10] were used to label the Golgi, the FAZ, the basal bodies, the FPC and the bi-lobed structure, respectively. DAPI (2 µg/ml) was used to stain kinetoplast and nuclear DNA. Images were acquired using Observer Z1 (Zeiss) equipped with a 63X NA1.4 objective and a CoolSNAP HQ^2^ CCD camera (Photometrics), or LSM 510 META (Zeiss) equipped with an EC Plan-Neofluar 100x NA1.3 objective, and processed with ImageJ and Adobe Photoshop.

### Cell motility assay

At various points of TbLRRP1-RNAi induction, cells were diluted using fresh culture medium to approximately 10^5^ cells/ml.10 µl of diluted cell culture were loaded onto a hemocytometer and visualized using a 20×NA0.4 objective. Images were captured every second for a total of 60 seconds using a high-speed HSM camera (Zeiss). The movement of individual cells was traced using ImageJ software with MtrackJ plugin. The mean velocity of individual cells was calculated according to the moving distance and moving time. Sedimentation assays were performed following previously described method [26]. Briefly, TbLRRP1-RNAi cells were diluted to 5×10^6^ cells/ml using fresh medium with or without tetracycline. 1 ml diluted cell culture was maintained at 28°C and measured for optical density at 600 nm every two hours for a total of 8 h. The change in OD value (ΔOD @600 nm) was calculated by subtracting the initial OD value obtained at t = 0 from the OD values obtained at later time points.

### Electron microscopy

Cells were fixed overnight with 2.5% glutaraldehyde at 4°C, postfixed with 1% (w/v) OsO4 for 1 h, dehydrated in increasing concentration of ethanol and embedded in Spurr's resin. Ultrathin sections were stained with 0.2% uranyl acetate and contrasted with lead citrate. The samples were observed using a JEM-1230 electron microscope (JOEL).

## Results

### Purification of *T. brucei* flagella

Flagellar complexes, which contain flagella and flagellum-associated basal bodies and bi-lobed structures, were purified as previously described using a two-step extraction with 1%NP-40 and 1 M KCl [12], [17], [18]. To purify flagella free of associated structures, flagella were detached from the cell bodies by sonication and then purified using sucrose density gradient ultracentrifugation. As with the flagellar complex, the purified flagellum was also extracted with 1% NP-40 and 1 M KCl, which further removed membrane and soluble components.

**Figure 1:**
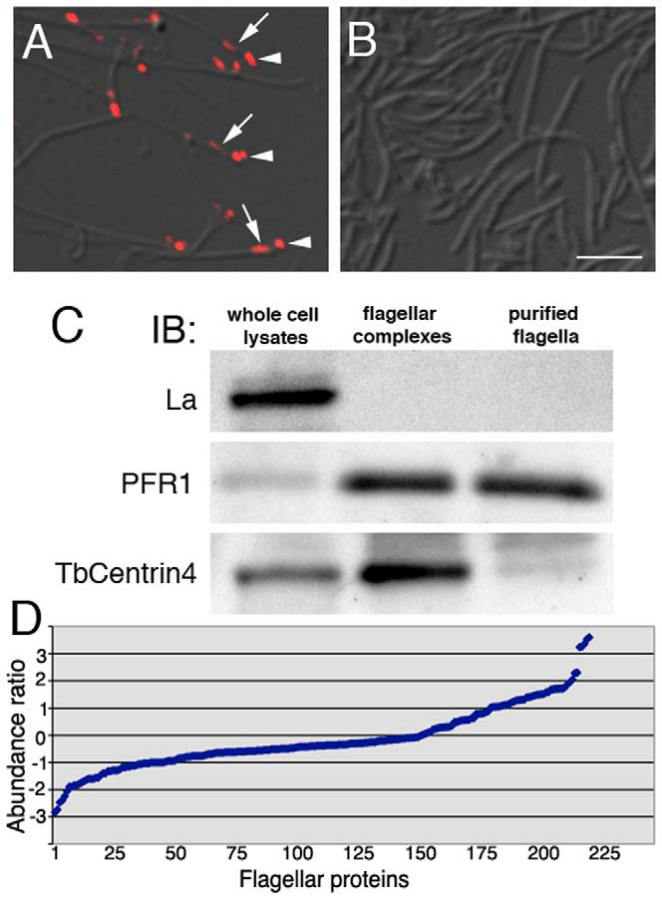
Purification of flagellar complexes and detached flagella. (A) Flagellar complexes purified through sequential extraction with 1%NP40 and 1 M KCl contained basal bodies (arrowheads) and bi-lobed structures (arrows) in addition to the flagella, as shown by anti-TbCentrin4 staining (red). (B) Purified detached flagella extracted and immunolabeled as in A were free of basal bodies and bi-lobed structures. Bar, 2 µm. (C) Immunoblotting demonstrates the absence of nuclear La protein and the enrichment of PFR1 in both flagellar complexes and detached flagella samples. TbCentrin4, however, was enriched only in flagellar complexes but not in purified flagella. Equal amount of protein (12 µg) was loaded in each lane. (D) The flagellar complexes and detached flagella samples were analyzed by iTRAQ and the abundance ratio (for calculations see Materials and Methods) was plotted for each of the 223 proteins identified (Table S1).

To confirm the lack of basal bodies and bi-lobed structures, the purified flagella fraction was fixed and labeled with an antibody against TbCentrin4, which stains both the basal bodies and the bi-lobed structure [10]. While TbCentrin4 labeling was readily identifiable at the tip of each flagellum in the extracted flagellar complex, no TbCentrin4 staining was detected in the detached flagella fraction (Fig. 1A, B). Immuno-blotting analyses further confirmed the absence of La, a small nuclear protein, and the enrichment of PFR1, a paraflagellar rod protein in both the flagellar complex and the flagella fraction. TbCentrin4 was enriched in the flagellar complex, but mostly eliminated in the detached flagella fraction (Fig. 1C).

### Comparative proteomics using iTRAQ

iTRAQ was used to identify proteins present in the flagellar complex but not in the detached flagellum fraction. A total of 223 proteins were identified (Table S1), of which 151 were also found in the previously published flagellar proteome [18]. The abundance ratio was calculated and plotted for each protein (see Materials and Methods; Fig. 1D). 135 of the 223 proteins showed abundance ratios between −1 and 1, and these proteins were considered equally abundant in both samples. 46 proteins showed abundance ratios  = <−1 and these were more abundant in the flagellum. A detailed analysis of the 46 proteins (Table S1) identified 18 paraflagellar rod (PFR) proteins and 7 putative axonemal proteins. The PFR is associated with the flagellar axoneme only after its exit from the cell body, and it was this part of the flagellum that was detached and enriched in the flagellum fraction (see Materials and Methods). The flagellar complexes, on the other hand, contained the entire flagellum including the intracellular, PFR-free region that was also tightly linked to basal bodies and bi-lobe (Fig. 1A). This may explain the relative abundance of PFR proteins in the flagellum fraction.

42 proteins were found to be more abundant in the flagellar complex with an abundance ratio > = 1. Further analysis identified 14 putative ribosomal proteins, and 10 putative mitochondrial, glycosomal or nuclear proteins. Many of these represented contaminants and were also found in the flagellar proteome [18]. Remarkably, TbMORN1 and TbBILBO1 were both found in this dataset. TbMORN1 was previously characterized by Morriswood *et al.* [12] as a component of the bi-lobed structure. TbBILBO1 is a structural protein found at the FPC and is tightly associated with the flagellar complex [4]. The presence of TbMORN1 and TbBILBO1 validated the comparative proteomics approach to identify proteins tightly associated with the flagellum. No centrin proteins were found enriched in the flagellar complex using this approach. It is possible that these proteins are of low abundance in the parasite cell or the flagellar complex.

### Subcellular localization of the candidate proteins

Proteins more abundant in the flagellar complex were reasoned to be proteins present on the basal bodies, the bi-lobe or other structures (e.g. FPC) that are tightly associated with the flagellum. The actual localization of the candidate proteins, however, needed to be verified. The cDNA of 16 selected candidates (including 13 proteins with an abundance ratio > = 1 and 3 proteins with an abundance ratio <1 but >0.5, all proteins with a coding sequence <4.5 Kb that was successfully amplified by PCR) were fused with a yellow fluorescent protein (YFP) reporter and over-expressed in *T. brucei* cells. The intracellular localizations of these proteins are summarized in Table S2. 4 proteins were present on the basal bodies, 2 associated with the flagellum or FAZ and 1 at the flagellar pocket. In addition, one protein was found present in the nucleus and a Sec31 homolog was localized to the ER exit site. 6 candidates, however, showed YFP labeling all over the parasite cells, but no particular localization to the flagellum complex when over-expressed as YFP fusions.

**Figure 2:**
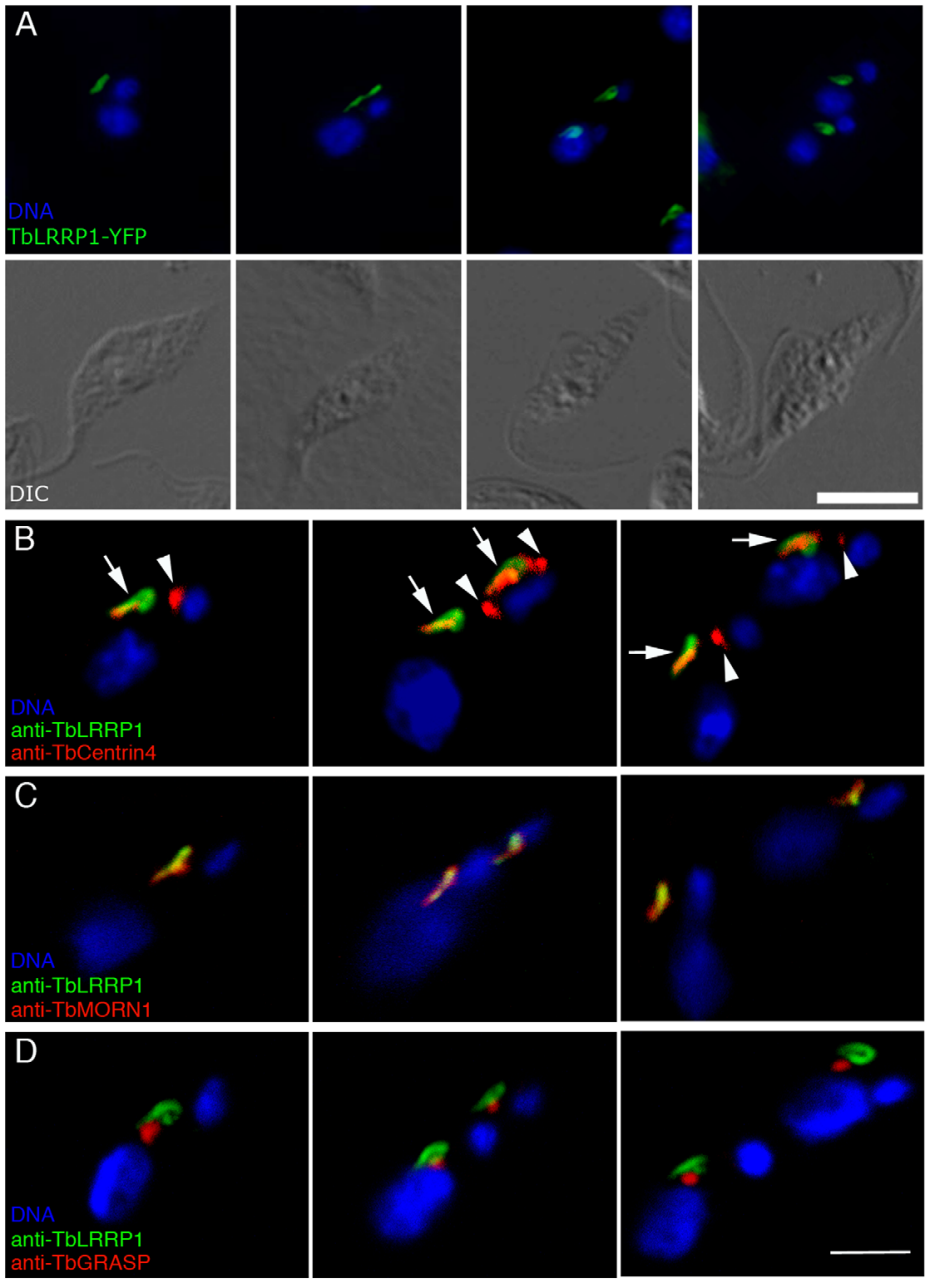
TbLRRP1 is a bi-lobe protein. (A) Cells stably over-expressing TbLRRP1-YFP fusion showed staining of looped or bi-lobed structures (green) that duplicated and segregated during the cell cycle. DIC images of corresponding fluorescence images are shown in the bottom panel. Bar, 5 µm. (B, C) YTat1.1 cells (B) or detergent-extracted cytoskeletons of YTat1.1 cells (C) were stained for TbLRRP1 (green) and bi-lobe markers TbCentrin4 (B; red) or TbMORN1 (C; red), and images acquired by confocal microscope. While TbLRRP1 partially overlapped with TbCentrin4 and TbMORN1 on the bi-lobe (arrows), it was not found on the basal bodies labeled by TbCentrin4 (arrowheads). (D) YTat1.1 cells were stained for TbLRRP1 (green) and a Golgi matrix marker TbGRASP (red). The Golgi apparatus was adjacent to the TbLRRP1-labeled bi-lobe during replication and segregation throughout the cell cycle. Bar, 3 µm.

A leucine-rich repeats-containing protein previously known as TbHERTS [18], now re-named TbLRRP1 due to the presence of leucine-rich repeats in its N-terminal half, was found to be present on a bi-lobed or looped structure (Fig. 2A), which were reminiscent of the Golgi-adjacent bi-lobed structure marked by TbCentrin2, TbCentrin4 and TbMORN1. Orthologs to TbLRRP1 were found in *Trypanosoma cruzi* and *Leishmania major*. Homologs were also found in other eukaryotes (Table S2). Examination of the amino acid sequence alignments suggested that the homology was entirely attributed to sequence similarity in the leucine-rich repeats, which form structural motif involved in protein-protein interactions and are ubiquitously found in all organisms in proteins with unrelated biological functions [27].

### TbLRRP1 localizes to the Golgi-associated bi-lobed structure

To confirm the bi-lobe localization, specific mouse monoclonal antibodies were raised to a 6xHis-tagged TbLRRP1 fragment (aa 481–713) (Fig. S1), and used together with anti-TbCentrin4 or anti-TbMORN1 in immunofluorescence assays (Fig. 2B, C). TbLRRP1 was found only on the bi-lobed structure containing TbMORN1 and TbCentrin4, but not at the basal bodies that were also stained by anti-TbCentrin4. Many TbLRRP1-labeled structures showed a loop-like staining at the posterior end, similar to that observed with TbMORN1 [12].

The association of the TbLRRP1-labeled structure with the Golgi apparatus was also examined (Fig. 2D). Similar to the results observed previously with TbCentrin2 [6], TbCentrin4 [10] and TbMORN1 [12], the single Golgi apparatus was found adjacent to the anterior half of the TbLRRP1-labeled bi-lobed structure. This close association persisted throughout the entire cell cycle.

### TbLRRP1 is essential for parasite growth and motility

**Figure 3:**
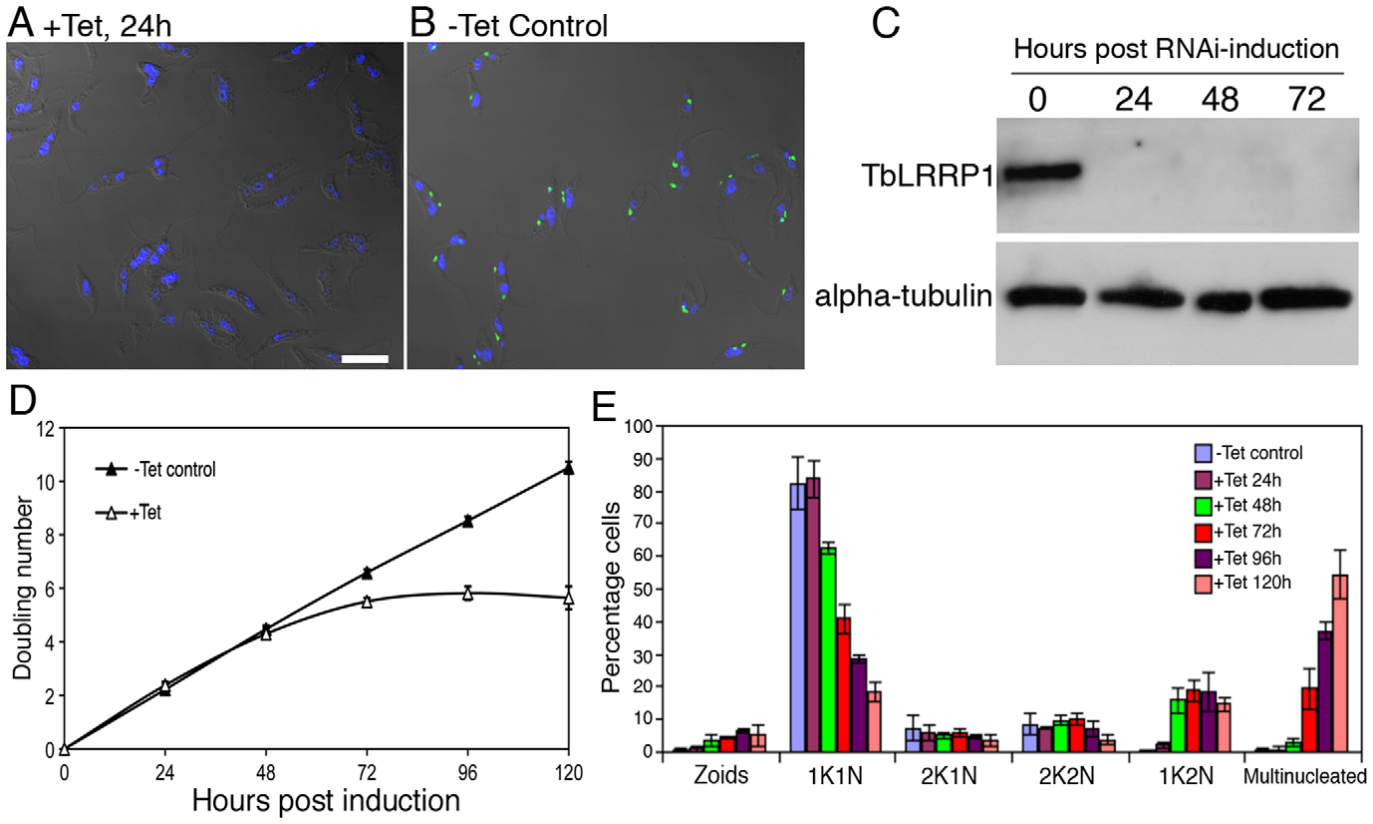
TbLRRP1-RNAi inhibits kinetoplast division and cytokinesis. Cells with a stably integrated TbLRRP1-RNAi construct were grown with tetracycline to induce RNAi or without. Samples were then taken for immunofluorecence labeling with anti-TbLRRP1 (A, B; bar, 10 µm) and immunoblottings with anti-TbLRRP1 and anti-alpha-tubulin (C) to check protein depletion efficiency. Growth assay (D; results shown as mean±SD, n = 3) and quantitation of various cell types (E; results shown as mean±SD, 500 cells were counted in each of 3 independent experiments) were also performed during the course of RNAi induction to monitor effects on growth rate and cell cycle.

To evaluate the function of TbLRRP1, the protein was depleted by tetracycline-inducible RNA interference (RNAi). Efficient protein depletion (>95%) was evident at 24 hours post-induction using both immunofluorescence (Fig. 3A, B) and immunoblotting (Fig. 3C) with the anti-TbLRRP1 antibody. Cell duplication slowed and then stopped after 72 hours post-induction (Fig. 3D), kinetics similar to that observed in TbCentrin2-RNAi cells but slower than that using TbCentrin4-RNAi [6], [10].

To analyze the effect of TbLRRP1 depletion on cell division, the TbLRRP1-RNAi cells were fixed and stained with DAPI to monitor their DNA contents at different time points (Fig. 3E). The unsynchronized, un-induced control population contained ∼82% cells with 1K1N (a single kinetoplast and a single nucleus), ∼9% with 2K1N (duplicated kinetoplasts but one nucleus) and ∼9% with 2K2N (two kinetoplasts and two nuclei). The cells induced for TbLRRP1 depletion, however, showed a decrease in 1K1N cells over time. A significant increase in 1K2N cells (*p*<0.01) was first observed at 48 hours post-induction, and this was followed by the accumulation of multinucleated cells at 72 hours post-induction. The number of zoids (cells lacking a nucleus) increased only slightly during the course of induction and represented at most ∼5% of the population. Together, these suggest an inhibition of kinetoplast division and cell division (but not nuclear division), similar to what was observed using TbCentrin2-RNAi [6].

**Figure 4:**
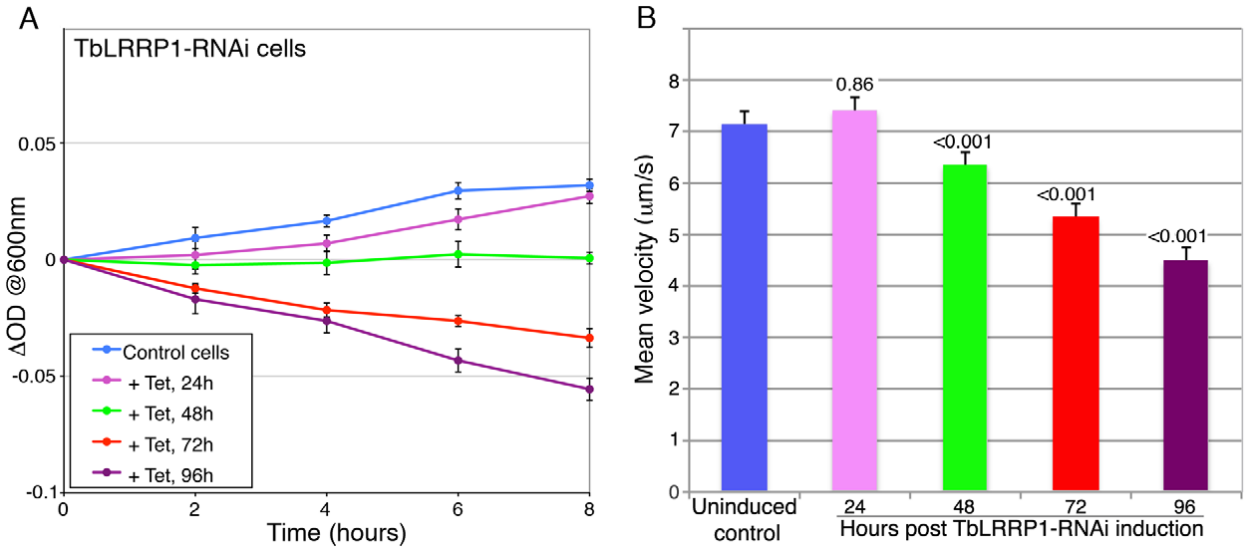
TbLRRP1-RNAi leads to defects in cell motility. At different time points post-RNAi induction, cells were diluted using fresh medium to desired concentrations for sedimentation assays (A) and individual cell tracking analyses (B). Results are shown as mean±SD, n = 3 (A) or mean velocity±SEM, 20–25 cells were measured in each of 3 independent experiments (B). P values are indicated on top of each measurement for the TbLRRP1-RNAi cells shown in (B). Sample movies used for the tracking analyses are shown in Movies S1, S2, S3, S4, S5.

Previous report suggested that TbLRRP1 depletion had inhibitory effects on parasite motility [18]. To verify this, TbLRRP1-RNAi cells were analyzed for cell motility by sedimentation analysis [26] as well as cell tracking analysis [28]. Consistent with previous observations, increasingly severe motility defects were observed over the course of RNAi-induction (Fig. 4, Movies S1, S2, S3, S4, S5).

### The effects of TbLRRP1-RNAi on kinetoplast division

**Figure 5:**
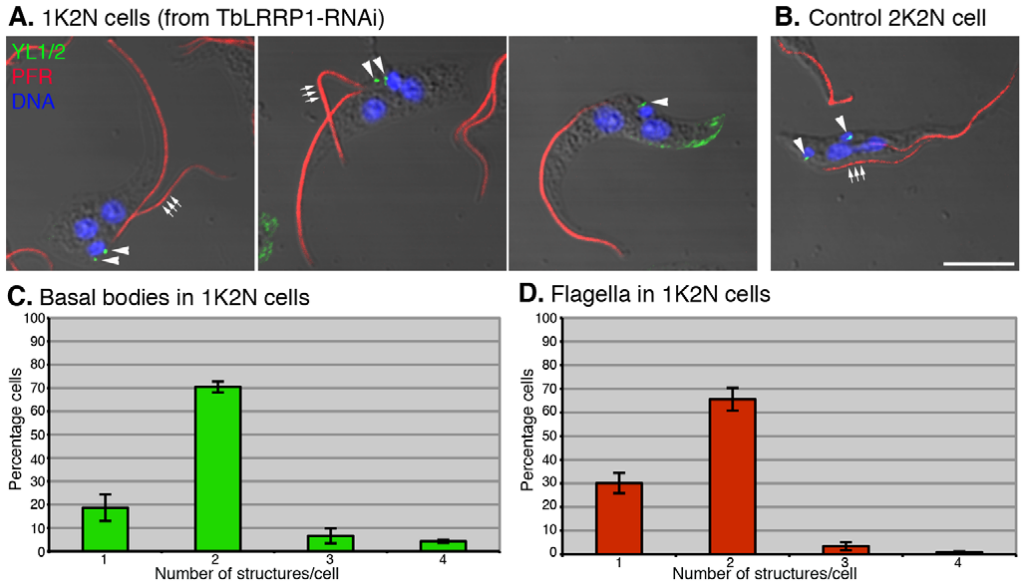
TbLRRP1-RNAi inhibits segregation, rather than duplication of basal bodies and flagellum. (A) Cells induced for TbLRRP1-RNAi for 48 hours and (B) control cells were fixed and labeled with DAPI for DNA (blue), YL1/2 for basal bodies (green; arrowheads) and anti-PFR1 for flagellum (red). Triple arrows indicate the new flagella. While new flagella in 1K2N cells were mostly detached from the cell bodies, new flagella in control 2K2N cells were always attached. Bar, 5 µm. The 1K2N cells in the TbLRRP1-RNAi population were counted for the number of (C) basal bodies and (D) flagellum present in each cell. Results were presented as means ± SD, n = 3. 250 cells were counted in each experiment.

In *T. brucei*, division of kinetoplast DNA is driven by basal body segregation [2]. TbLRRP1, however, was present exclusively on the bi-lobed structure and not detected on the basal bodies. It was therefore surprising to observe an inhibitory effect on kinetoplast division in TbLRRP1-RNAi cells. To analyze the duplication and segregation of the basal bodies, cells at 48 h after RNAi induction were stained with the YL1/2 antibody for the basal bodies and anti-PFR1 [29] for the flagella, which grow from mature basal bodies (Fig. 5). In TbLRRP1-depleted cells, ∼70% of 1K2N cells had two YL1/2-positive basal body structures and two flagella, while others contained a single basal body structure and a single flagellum, suggesting only a modest inhibition on the duplication of these structures. However, the duplicated basal bodies and flagella remained clustered, suggesting failure in segregation. Also interestingly, in cells with two flagella, the new (and more posterior) flagella were often (>70% of the cases) found detached from the cell body (Fig. 5A, B), which may possibly explain the motility defects observed in TbLRRP1-RNAi cells (Fig. 4) [18].

### The effects of TbLRRP1-RNAi on Golgi duplication

**Figure 6:**
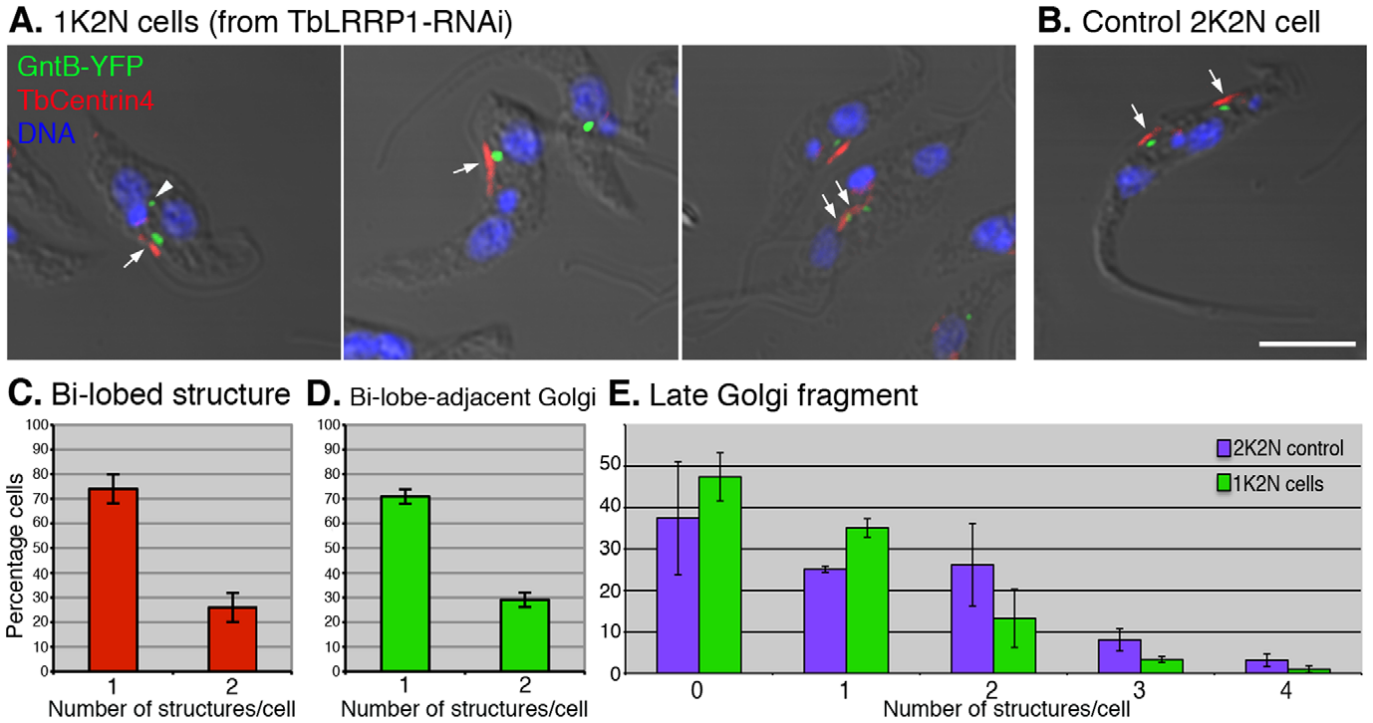
TbLRRP1-RNAi inhibits duplication of bi-lobe and Golgi. (A, B) Cells stably expressing GntB (56)-YFP (green) were induced for TbLRRP1-RNAi for 48 hours or not. Cells were fixed and labeled with DAPI for DNA (blue), and anti-TbCentrin4 for bi-lobe (red; arrows). Bar, 5 µm. The number of (C) bi-lobed structures, (D) bi-lobe-assoicated Golgi and (E) late Golgi fragments (arrowhead in A) present in each cell were counted for 1K2N cells in the TbLRRP1-RNAi population, as well as the 2K2N cells in the control population (E only). Results are presented as means ± SD, 250 cells were counted in each of 3 independent experiments.

As the bi-lobed structure was previously found to be important for Golgi duplication, the effect of TbLRRP1-depletion on Golgi duplication was examined. To allow visualization of the Golgi apparatus simultaneously with the bi-lobed structure, the TbLRRP1-RNAi cell line was stably transfected with a TbGntB(56)-YFP construct that contains the N-terminal 56aa Golgi-localizing sequence of a putative Golgi enzyme TbGntB fused to YFP reporter [3], [30]. RNAi was then induced and the cells were fixed and stained with DAPI for DNA and anti-TbCentrin4 for the bi-lobed structure (Fig. 6). To ensure accurate quantification, the 1K2N cells (∼20% of the population at 48 h post-induction; Fig. 3E) were counted for the number of Golgi and the bi-lobed structure. As expected, the duplication of bi-lobe and the bi-lobe-associated Golgi was inhibited. >70% 1K2N cells contained a single bi-lobed structure (Fig. 6C) and a single bi-lobe-associated Golgi (Fig. 6D). On occasions, larger bi-lobed structures were observed (Fig. 6A, middle panel). Using currently available markers, it was not possible to distinguish if these larger structures contained duplicated bi-lobes that could not segregate. In comparison, all un-induced control cells with two nuclei contained two segregated bi-lobed structures and two bi-lobe-associated Golgi (Fig. 6B).

Late Golgi fragments are often observed in cells during division. These Golgi structures are highly variable in number, size and location, and they are not associated with the bi-lobed structure [6], [31]. To evaluate how late Golgi were affected by TbLRRP1-depletion, the number of Golgi that were not associated with the bi-lobe was counted in the 1K2N cells and control 2N2K cells (Fig. 6E). While ∼40% 1K2N cells did not contain detectable late Golgi, ∼35% contained one late Golgi structure, and the rest contained 2-4. On average, each 1K2N cell contained 0.75±0.12 late Golgi. In comparison, control 2N2K cells contained 1.14±0.32 late Golgi/cell in addition to the two bi-lobe associated Golgi.

### The effects of TbLRRP1-RNAi on FAZ formation

**Figure 7:**
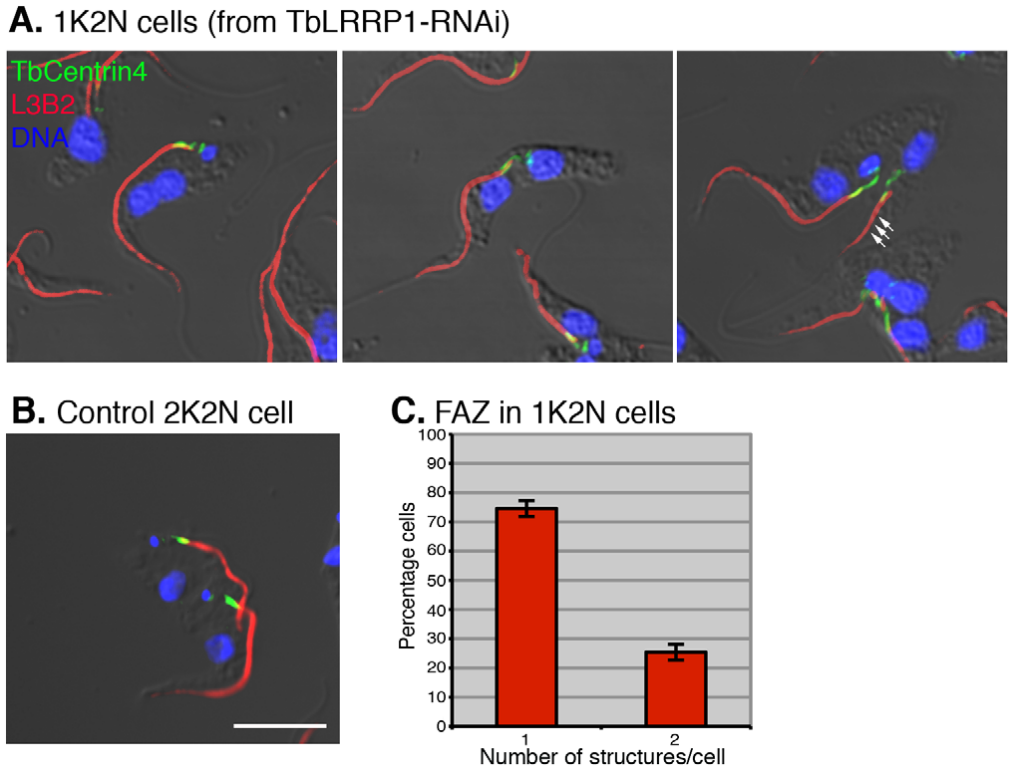
TbLRRP1-RNAi inhibits the formation of new FAZ. (A) TbLRRP1-RNAi (48 h post-induction) and (B) control cells were fixed and labeled with DAPI for DNA (blue), anti-TbCentrin4 for basal bodies and bi-lobed structure (green) and L3B2 for FAZ (red). Triple arrows indicate the new, truncated FAZ. Bar, 5 µm. (C) The number of FAZ present in each 1K2N cell was counted and results were presented as means ± SD, n = 3. 250 cells were counted in each experiment.

FAZ is a cytoplasmic structure responsible for attaching the flagellum to the cell body [32], [33]. Defects in FAZ can lead to detachment of flagellum from cell body and cytokinesis arrest [34], [35]. As TbLRRP1-depleted cells showed detached flagellum (Fig. 5A) and were unable to divide (Fig. 3D, E), the duplication of FAZ was examined. TbLRRP1-RNAi cells were labeled with anti-TbCentrin4 and L3B2, a monoclonal antibody specific to FAZ filament (Fig. 7A, B). Quantitation showed that >70% 1K2N cells contained a single bi-lobed structure as well as a single FAZ (Fig. 7C). In cells with two bi-lobed and two FAZ structures, the more posterior, new FAZ often appeared shorter (Fig. 7A; triple arrows). The length of both old and new FAZ was therefore measured in both 1K2N cells and control 2K2N cells (Table 1). In 1K2N cells where a new FAZ was found, the average length of the new FAZ (6.3±1.7 µm) was significantly shorter (*p = *1.85×10^−141^) than new FAZ in control 2K2N cells (10.6±1.7 µm). The average length of new flagella in 1K2N cells (10.9±2.7 µm) was also shorter (*p = *4.4×10^−6^) than new flagella in control 2K2N cells (12.1±1.7 µm), though the inhibition on new flagellum elongation was not as dramatic as the inhibition on new FAZ elongation.

**Table 1:** Flagellar length, FAZ length, kinetoplast and nucleus migration in binucleated cells.

Cells	Cell type	Flagellar length (µm)		FAZ length (µm)		Kinetoplast migration (µm)	Nucleus migration (µm)
		Old	New	Old	New		
Uninduced control	2K2N	15.5±2.3	12.1±1.7	14.3±2.5	10.6±1.7	4.4±0.9	4.8±0.8
TbLRRP1-RNAi	1K2N	17.4±2.5	10.9±2.7*	15.8±2.8	6.3±1.7*	0	4.8±1.0

For each measurement, only cells with completely divided nuclei (no detectable connection between daughter nuclei) were counted. 100 cells were measured in each of 3 independent experiments. Results are shown as average length or distance ± SD. For TbLRRP-RNAi, 1K2N cells, if only one flagellum and/or one FAZ were found in the cell, the single flagellum/FAZ was measured as the old structure.

*For cells completely lacking a new flagellum/FAZ, the length measurement (0 µm) was not taken into consideration in the average length calculations.

The morphological details of the flagellum and the FAZ were further examined in TbLRRP1-RNAi cells by electron microscopy (Fig. S2). No obvious morphological defects could be detected in the flagellar axoneme or the paraflagellar rod structures. The FAZ structure, however, was difficult to find in cells with detached flagellum.

### The effects of TbLRRP1-RNAi on FPC

TbBILBO1 was previously characterized as a protein component of the FPC, which forms an adhesion zone at the flagellar pocket. Depletion of TbBILBO1 leads to defects in flagellar pocket biogenesis, Golgi segregation as well as FAZ formation [4]. The presence of TbBILBO1 in the flagellar complex proteome prompted for a closer examination of possible interaction between the bi-lobe and the FPC.

**Figure 8:**
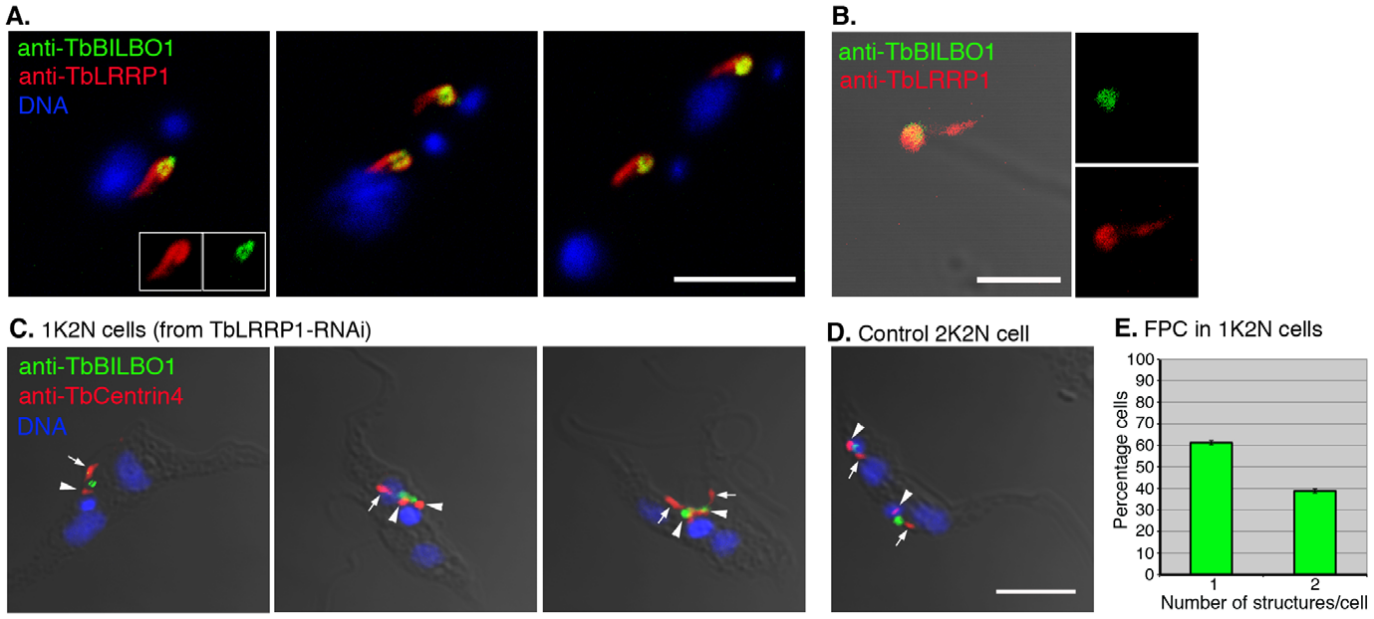
TbLRRP1-RNAi inhibits FPC duplication. (A) Methanol fixed YTat1.1 cells were labeled with anti-TbLRRP1 (red) and anti-TbBILBO1 (green) and images acquired by confocal microscope. Bar, 5 µm. (B) Extracted flagellar complex as described in Fig. 1A were labeled with anti-TbLRRP1 (red) and anti-TbBILBO1 (green) antibodies. The association between the TbLRRP1-labeled bi-lobe and TbBILBO1-labeled FPC maintained after detergent/salt extractions. Bars, 2 µm. (C, D) TbLRRP1-RNAi (48 h post-induction)(C) and control cells (D) were fixed and labeled with DAPI for DNA (blue), anti-TbBILBO1 for FPC (green) and anti-TbCentrin4 (red) basal bodies (arrowheads) and bi-lobed structures (arrows). (E) The number of FPC in each cell was counted for the 1K2N cells in the TbLRRP1-RNAi population. Results were presented as means ± SD. 200 cells were counted in each of 3 independent experiments.

YTat 1.1 cells were fixed and labeled with anti TbLRRP1 and anti-TbBILBO1 (Fig. 8A). The ring- or horseshoe-shaped FPC structure labeled with anti-TbBILBO1 was found to duplicate and segregate together with the bi-lobe, with the FPC often nested in the looped, posterior end of the TbLRRP1-labeled bi-lobed structure (Fig. 8A, inset). This association maintained even after detergent/salt extractions used to prepare the flagellar complexes (Fig. 8B). To further investigate the interaction between the bi-lobe and the FPC, anti-TbBILBO1 was also examined in TbLRRP1-RNAi cells. ∼60% 1K2N cells contained only one FPC, while ∼40% contained two FPCs, suggesting an inhibition of new FPC formation in TbLRRP1-RNAi cells (Fig. 8C–E). Large FPC staining was observed in some cells. However, it was not possible to distinguish whether it contained duplicated FPCs that were very close to each other (Fig. 8C, middle panel). In cells with two FPCs, the duplicated FPCs remained unsegregated (Fig. 8C, right panel; Fig. S2B). Transmission electron microscopy, however, did not reveal any additional morphological defects in the flagellar pocket region in TbLRRP1-RNAi cells (Fig. S2).

## Discussion

A comparative proteomics approach was used to identify new components of the bi-lobed structure. While the ultrastructural details of the bi-lobe remain to be understood [6], [36], it contains *T. brucei* homologs to centrins and Polo-like Kinase, all of which are important for organelle duplication and/or subsequent cell division in yeast and mammalian cells. The observation that the bi-lobed structure was tightly associated with the flagellum, even after detergent extraction and high-salt wash, suggests that it is a core component of the flagellar complex, which also includes the basal bodies that seed flagellum growth and the FPC that is required for flagellar pocket biogenesis. Using a modified cell fractionation protocol that was first developed by da Cunha e Silva *et al.* [19], flagella detached from cell bodies and therefore free of associated basal bodies or bi-lobes were purified from *T. brucei*. The flagellar complex and the purified flagella samples were then analyzed by iTRAQ, which allowed the relative abundance of each protein to be calculated leading to the identification of proteins present only in the flagellar complex but not in the detached flagella.

A total of 223 proteins were identified by the iTRAQ analysis, 67.7% of which were previously found in the flagellar proteome [18]. Based on a cutoff abundance ratio at 1 (or −1), 42 proteins were more abundant in the flagellar complex and among them were TbMORN1 and TbBILBO1, both found in structures tightly associated with the flagellar complex [4], [12]. Further bioinformatics screening and localization studies by YFP-tagging revealed one new bi-lobe protein. The protein was renamed TbLRRP1 due to a leucine-rich repeats-containing domain in its N-terminal region. It was also known as TbHERTS and was first characterized in the flagellar proteome [18]. In the same studies, RNAi depletion in procyclic *T. brucei* resulted in a motility defect while flagellum formation and cytokinesis both appeared normal [18].

The bi-lobe localization of TbLRRP1 was verified using a mouse monoclonal antibody raised specifically against the TbLRRP1 protein. TbLRRP1 was present on the Golgi-adjacent bi-lobe together with TbCentrin4 and TbMORN1. In addition to the Golgi, the TbLRRP1-labeled bi-lobe was adjacent to the TbBILBO1-labeled FPC on the posterior side of the bi-lobe and the FAZ on the anterior side. Consistent with pervious report [18], depletion of TbLRRP1 led to cell motility defects by sedimentation and tracking assays, and no obvious defects were observed in flagellum structure by transmission electron microscopy. In our experiments, however, TbLRRP1-RNAi generated a cell division phenotype, where kinetoplast division and cell division were both inhibited, leading to accumulation of 1K2N cells at 48 hours post-induction and multinucleated cells at later times. It is not clear why cell division defects were not observed in the previous report [18]. Though different RNAi fragments were used and different cultivation conditions may result in phenotypic differences, it seems likely that the late inhibition of cell duplication (>48 hour post-induction) and even later emergence of multinucleated cells at 72 hour post-induction prevented detection of cell division phenotypes at earlier time points, as was done in the previous study [18].

The effects of TbLRRP1-RNAi on organelle duplication and cell division were then evaluated for basal bodies, flagellum, Golgi, FPC and FAZ in the 1K2N cells where cell division defects were first observed. Both the basal bodies and the flagellum appeared to duplicate normally in ∼70% of the 1K2N cells, though the duplicated organelles remained close together without segregation. In 1K2N cells containing two flagella, the new flagella were slightly shorter than those in control cells (10.9±2.7 µm versus 12.1±1.7 µm), and no obvious structural defects in axoneme or PFR could be observed by electron microscopy. On the other hand, duplication of the Golgi, FPC and FAZ were all inhibited, with >60% of 1K2N cells having only one Golgi, one FPC, one FAZ and one bi-lobe. Even in 1K2N cells that contained two FAZ, the new FAZ structure was much shorter compared to new FAZ in control cells (6.3±1.7 µm versus 10.6±1.7 µm). Due to the lack of a functional new FAZ, the newly formed flagellum was not attached to the cell body, which perhaps resulted in the motility defects observed. The lack of new FAZ formation may also account for the inhibited basal body segregation and kinetoplast division in the same cells. Relative sliding movement of the new flagellum attached to the old has been proposed as a mechanism for basal body segregation, which in turn divides the kinetoplast DNA [37]. The cytokinetic defects observed in TbLRRP1-RNAi cells may also be attributed to inhibition of new FAZ formation. FAZ has long been observed as a structure important for proper cell division in *T. brucei* though its exact role in this process is not yet clear. The bi-lobed structure appears to partially overlap with the posterior tip of the FAZ [10], [12] and depletion of bi-lobe proteins TbCentrin2 and TbLRRP1 both inhibited FAZ formation, flagellar attachment and cell division [10 and this study], suggesting a role of the bi-lobe in FAZ function.

Interestingly, the duplication of the late Golgi fragments also seemed to be inhibited. The function of these late Golgi is still not clear. They often appear late in the cell cycle at the time of cell division, and then mostly disappear in newly formed daughter cells [3], [6], [31]. Unlike the main Golgi, the late Golgi are not adjacent to the bi-lobed structure, and are possibly not a core component of duplication.

Five proteins have so far been found on the bi-lobed structure: TbCentrin2 [6], TbCentrin4 [10], TbPLK1 [11], TbMORN1 [12] and TbLRRP1 (this paper). While TbPLK1 is only transiently associated with the bi-lobe right before its duplication, the other proteins are found on the bi-lobe at all times during the cell cycle. Depletion of the bi-lobe proteins has a common effect on organelle duplication and/or cell division, though in the case of TbMORN1, such an effect is only moderate in procyclic cells (the insect form parasites used in these studies). Most interestingly, the Golgi, FPC and FAZ have all been shown to be adjacent or partially overlapped with the bi-lobed structure [6, 10, 12 and this study]. Whether or not they are directly linked remained to be answered but these observations raise the possibility that the bi-lobe may be a core structure mediating the co-ordinated duplication and segregation of these single-copied organelles important for cell propagation.

TbLRRP1 contains several leucine rich repeats in its N-terminal region and a coiled coil motif in its C-terminal region. While leucine-rich repeats have been implicated in protein-protein interactions [27], coiled coils are thought to mediate protein oligomerizations [38]. Further examination of TbLRRP1 and its binding proteins may help to understand the molecular mechanism of the bi-lobed structure and how it interacts with other single-copied organelles.

## Supporting Information

Figure S1 Characterization of TbLRRP1 antibody. Total cell lysates containing equal amounts of protein from control YTat1.1 cells or cells stably expressing YFP-TbLRRP1 were fractionated by SDS-PAGE and immuno-blotted using anti-GFP and anti-TbLRRP1 antibodies, respectively. The anti-TbLRRP1 antibody detected only a single band in YTat1.1 lysate at ∼75 kDa, which was close to the estimated size of the 713aa-TbLRRP1 protein. In the YFP-TbLRRP1 lysates, two bands were detected, one at ∼75 kDa for the wild type protein, the other at ∼100 KDa for the YFP-TbLRRP1 fusion. The latter was also detected by anti-GFP. ** indicates a non-specific band just below 75 KDa by the anti-GFP antibody, which was present in both YTat1.1 and YFP-TbLRRP1 lysates.
(1.64 MB TIF)

Figure S2 Electron microscopic analyses of TbLRRP1-RNAi mutants. TbLRRP1-RNAi (48 h post-induction) (A) and control un-induced (B) cells were processed for transmission electron microscopy to examine if any morphological differences in flagellum, flagellar pocket and FAZ in the RNAi cells. No obvious defects were detected in the flagellar axoneme or paraflagellar rod structures. The shape and size of the flagellar pockets also appeared normal, though segregation of the flagellar pockets was inhibited (compare the distance between the duplicated flagellar pockets in cells shown in the right panels of A and B, which were at approximately the same cell cycle stage based on the v-shaped kinetoplasts). FAZ (double arrowheads) was more difficult to find in TbLRRP1-RNAi cells than in control cells. This, however, could also be due to the lack of attached flagellum to provide any positional cue. K, kinetoplast; FP, flagellar pocket.
(8.51 MB TIF)

Table S1 List of all 223 proteins identified by iTRAQ-based proteomic approach and comparison with previous flagellar proteomes.
(0.57 MB DOC)

Table S2 Summary of candidate flagellar complex proteins identified in this study.
(0.06 MB DOC)

Text S1 Summaries of iTRAQ-labeled proteins that have only a single peptide match.
(4.65 MB DOC)

Movie S1 Control cells were diluted with fresh medium to 100,000 cell/ml. 10 µl of diluted culture was loaded into a hemocytometer chamber and imaged every second for 1 minute.
(7.21 MB MOV)

Movie S2 TbLRRP1-RNAi cells (24 h post induction) were diluted with fresh medium to 100,000 cell/ml. 10 µl of diluted culture was loaded into a hemocytometer chamber and imaged every second for 1 minute.
(6.95 MB MOV)

Movie S3 TbLRRP1-RNAi cells (48 h post induction) were diluted with fresh medium to 100,000 cell/ml. 10 µl of diluted culture was loaded into a hemocytometer chamber and imaged every second for 1 minute.
(7.08 MB MOV)

Movie S4 TbLRRP1-RNAi cells (72 h post induction) were diluted with fresh medium to 100,000 cell/ml. 10 µl of diluted culture was loaded into a hemocytometer chamber and imaged every second for 1 minute.
(7.71 MB MOV)

Movie S5 TbLRRP1-RNAi cells (96 h post induction) were diluted with fresh medium to 100,000 cell/ml. 10 µl of diluted culture was loaded into a hemocytometer chamber and imaged every second for 1 minute.
(6.38 MB MOV)
