# Supplementary figures and images for: A Comparative Proteomic Analysis Reveals a New Bi-Lobe Protein Required for Bi-Lobe Duplication and Cell Division in Trypanosoma brucei
Source: PLoS One. 2010 Mar 15;5(3):e9660. doi: 10.1371/journal.pone.0009660 (PMC2837748; doi:10.1371/journal.pone.0009660)

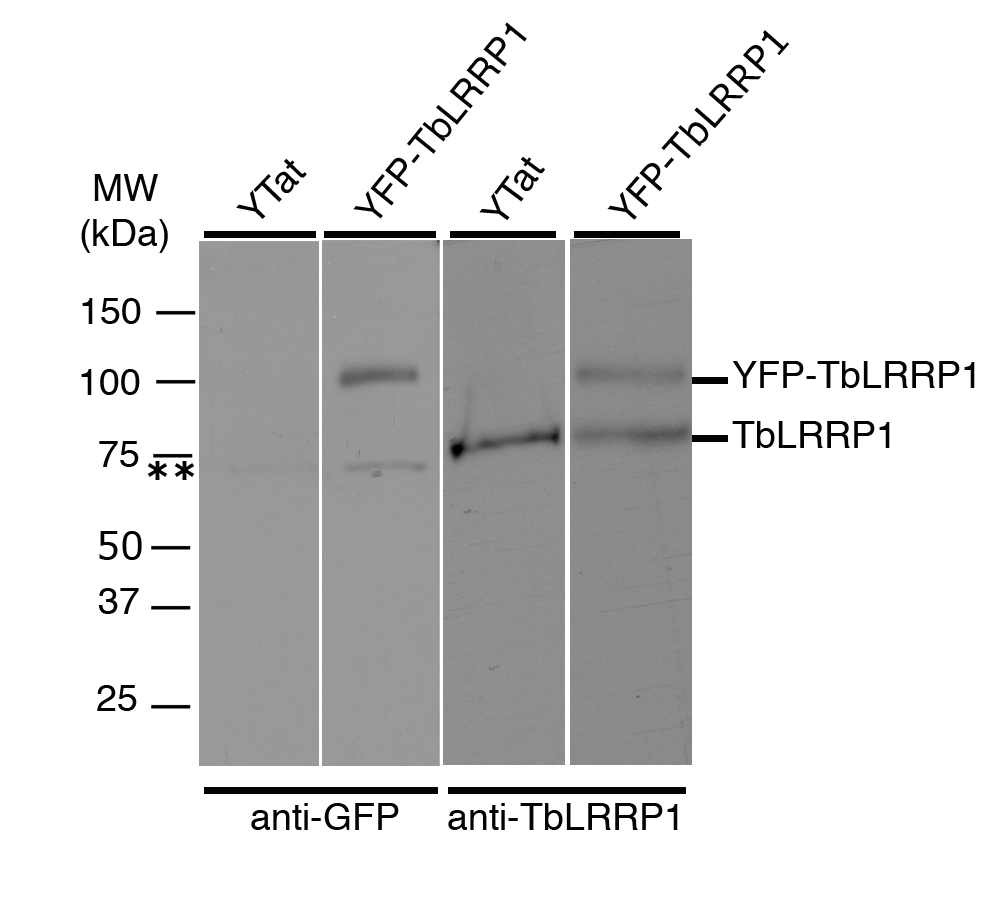

Supplement: Figure S1 — Characterization of TbLRRP1 antibody. Total cell lysates containing equal amounts of protein from control YTat1.1 cells or cells stably expressing YFP-TbLRRP1 were fractionated by SDS-PAGE and immuno-blotted using anti-GFP and anti-TbLRRP1 antibodies, respectively. The anti-TbLRRP1 antibody detected only a single band in YTat1.1 lysate at ∼75 kDa, which was close to the estimated size of the 713aa-TbLRRP1 protein. In the YFP-TbLRRP1 lysates, two bands were detected, one at ∼75 kDa for the wild type protein, the other at ∼100 KDa for the YFP-TbLRRP1 fusion. The latter was also detected by anti-GFP. ** indicates a non-specific band just below 75 KDa by the anti-GFP antibody, which was present in both YTat1.1 and YFP-TbLRRP1 lysates. (1.64 MB TIF) [file pone.0009660.s001.tif]

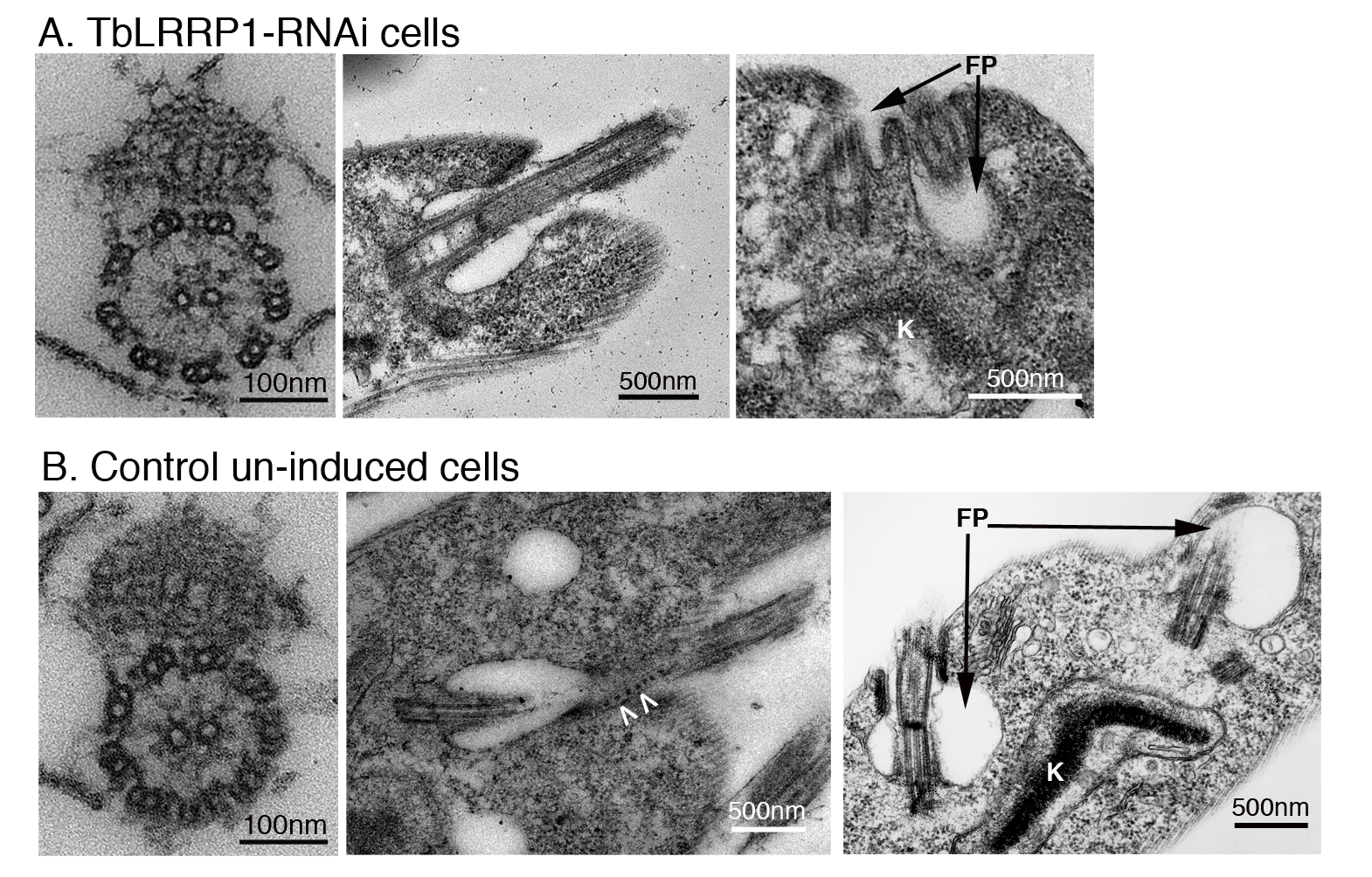

Supplement: Figure S2 — Electron microscopic analyses of TbLRRP1-RNAi mutants. TbLRRP1-RNAi (48 h post-induction) (A) and control un-induced (B) cells were processed for transmission electron microscopy to examine if any morphological differences in flagellum, flagellar pocket and FAZ in the RNAi cells. No obvious defects were detected in the flagellar axoneme or paraflagellar rod structures. The shape and size of the flagellar pockets also appeared normal, though segregation of the flagellar pockets was inhibited (compare the distance between the duplicated flagellar pockets in cells shown in the right panels of A and B, which were at approximately the same cell cycle stage based on the v-shaped kinetoplasts). FAZ (double arrowheads) was more difficult to find in TbLRRP1-RNAi cells than in control cells. This, however, could also be due to the lack of attached flagellum to provide any positional cue. K, kinetoplast; FP, flagellar pocket. (8.51 MB TIF) [file pone.0009660.s002.tif]
