# Supplementary material for: A Comparative Proteomic Analysis Reveals a New Bi-Lobe Protein Required for Bi-Lobe Duplication and Cell Division in Trypanosoma brucei
Source: PLoS One. 2010 Mar 15;5(3):e9660. doi: 10.1371/journal.pone.0009660 (PMC2837748; doi:10.1371/journal.pone.0009660)
Supplement: Table S1 — List of all 223 proteins identified by iTRAQ-based proteomic approach and comparison with previous flagellar proteomes. (0.57 MB DOC) [file pone.0009660.s003.doc]

Table S1. List of all 223 proteins identified by iTRAQ-based proteomic approach and comparison with previous flagellar proteomes.

Total ion score and best ion score were based on MS/MS Mascot search, the total ion score C.I. (confidence interval) and best ion score C.I. were calculated by GPS explorer. The protein with best ion score C.I. (%) >= 95 were considered statistically to be real protein identifications at 95% confidence interval. Mean values and standard deviation (SD) of iTRAQ ratios were calculated based on all peptides iTRAQ ratios of the same protein. The p values were determined based on t-test analysis of the iTRAQ ratio of peptides matched to the same protein. Standard deviation and p values are not available for proteins identified by single peptide. NA means not available. ‘+’ denotes that this protein was also identified by the corresponding proteomic studies.

| **No.** | **Accession No.** | **Protein Name** | **Molecular weight (kDa**) | **pI** | **Unique peptide count** | **Sequence tag** | **Total ion score** | **Total ion score C.I. (%)** | **Best ion score** | **Best ion score C.I. (%)** | **Average iTRAQ ratio**  **(115/114) ± SD** | **P value** | **Average iTRAQ ratio**  **(117/116) ± SD** | **P value** | **Abundance Ratio Log_2_(115/114 + 117/116)/2** | **Broadhead et al., 2006** | **Portman et al., 2009** |
| --- | --- | --- | --- | --- | --- | --- | --- | --- | --- | --- | --- | --- | --- | --- | --- | --- | --- |
| 1 | Tb11.02.4320 | hypothetical protein, conserved | 107.3 | 6.9 | 4 | HTEADGGSANYLAQTR | 309.56 | 100 | 103.15 | 100 | 0.13 ± 0.03 | 0.00 | 0.16 ± 0.06 | 0.00 | -2.84 | + |  |
| 2 | Tb927.4.4040 | hypothetical protein, conserved | 30.2 | 4.5 | 1 | LHAFLIPLEDGEQFAR | 81.87 | 99.99 | 81.87 | 99.99 | 0.12 ± 0 | NA | 0.19 ± 0 | NA | -2.74 |  |  |
| 3 | Tb10.6k15.0140 | PFC19 | 37 | 5.2 | 5 | VILHGVVCDPENPLFTPEGCAAR | 360.02 | 100 | 101.91 | 100 | 0.17 ± 0.08 | 0.00 | 0.18 ± 0.11 | 0.00 | -2.47 |  | + |
| 4 | Tb10.70.7330 | ADKB | 29.7 | 4.8 | 2 | FGWLLDGFPR | 107.46 | 100 | 57.41 | 99.98 | 0.18 ± 0.11 | 0.03 | 0.19 ± 0.11 | 0.03 | -2.4 | + | + |
| 5 | Tb09.160.1660 | hypothetical protein, conserved | 9.2 | 9.3 | 1 | IGVGPLLEDFVADAVR | 76.43 | 99.99 | 76.43 | 99.99 | 0.23 ± 0 | NA | 0.19 ± 0 | NA | -2.25 |  |  |
| 6 | Tb927.3.3690 | flagellar radial spoke protein-like, putative | 60.9 | 4.1 | 2 | WASSFGTALVPPKPR | 134.52 | 100 | 72.3 | 99.99 | 0.24 ± 0.05 | 0.01 | 0.24 ± 0.00 | 0.00 | -2.06 | + |  |
| 7 | Tb10.6k15.0810 | PFC14 | 118.6 | 4.9 | 12 | ISNYGMQQMSK | 687.25 | 100 | 100.35 | 100 | 0.26 ± 0.17 | 0.00 | 0.29 ± 0.16 | 0.00 | -1.89 | + | + |
| 8 | Tb927.7.4460 | hypothetical protein, conserved | 91.1 | 6.1 | 1 | GGFELFCPLFR | 36.85 | 97.59 | 36.85 | 97.59 | 0.26 ± 0 | NA | 0.29 ± 0 | NA | -1.89 | + |  |
| 9 | Tb927.8.4970 | PFR2 | 69.5 | 6.8 | 25 | TACLSNEEYVQDLHVSEWSETQK | 1983.06 | 100 | 188.99 | 100 | 0.24 ± 0.09 | 0.00 | 0.31 ± 0.08 | 0.00 | -1.84 | + | + |
| 10 | Tb11.01.1625 | hypothetical protein, conserved | 11.8 | 4.4 | 4 | HLTVEEIENNIGGLAITDNK | 366.39 | 100 | 116.48 | 100 | 0.24 ± 0.08 | 0.00 | 0.32 ± 0.16 | 0.00 | -1.84 |  |  |
| 11 | Tb927.3.4290 | PFR1 | 68.6 | 5.9 | 27 | TACLENEELVQELHVSDWSETQR | 1967.01 | 100 | 192.14 | 100 | 0.25 ± 0.08 | 0.00 | 0.33 ± 0.37 | 0.00 | -1.79 | + | + |
| 12 | Tb10.61.1550 | hypothetical protein, conserved | 48 | 7.3 | 2 | TLQGNWSEER | 68.3 | 99.99 | 45.86 | 99.7 | 0.27 ± 0.13 | 0.04 | 0.33 ± 0.07 | 0.02 | -1.74 | + |  |
| 13 | Tb11.01.6740 | Tb5.20 | 127.9 | 3.8 | 8 | WLFDMYGVR | 332.2 | 100 | 57.7 | 99.98 | 0.27 ± 0.17 | 0.00 | 0.36 ± 0.27 | 0.00 | -1.69 | + | + |
| 14 | Tb10.389.1320 | hypothetical protein, conserved | 24.6 | 5.1 | 1 | DSGDNITWQNHELLTSAGFDLR | 113.4 | 100 | 113.4 | 100 | 0.29 ± 0 | NA | 0.35 ± 0 | NA | -1.64 | + |  |
| 15 | Tb11.01.4623 | calmodulin | 16.8 | 3.9 | 3 | EADVDGDGQINYEEFVK | 195.63 | 100 | 132.81 | 100 | 0.30 ± 0.08 | 0.00 | 0.35 ± 0.04 | 0.00 | -1.6 | + | + |
| 16 | Tb927.3.3770 | PFC6 | 31.6 | 4.4 | 4 | VGVVDDGKPPLLVR | 211.09 | 100 | 62.27 | 99.99 | 0.31 ± 0.03 | 0.00 | 0.35 ± 0.04 | 0.00 | -1.6 | + | + |
| 17 | Tb927.6.4140 | PFC4 | 13 | 6.5 | 2 | PNLVVTATFSPPAFSIVGSPLR | 138.9 | 100 | 80.64 | 99.99 | 0.27 ± 0.02 | 0.01 | 0.40 ± 0.03 | 0.01 | -1.6 | + | + |
| 18 | Tb10.26.0680 | PFC16 | 14.3 | 5.6 | 5 | LVSSFVTKEPSGTGSK | 411.25 | 100 | 128.9 | 100 | 0.28 ± 0.09 | 0.00 | 0.39 ± 0.15 | 0.00 | -1.56 | + | + |
| 19 | Tb11.02.5550 | hypothetical protein, conserved | 67.6 | 6.5 | 3 | NITWWDSVDCGAIR | 134.25 | 100 | 63.17 | 99.99 | 0.32 ± 0.06 | 0.00 | 0.36 ± 0.14 | 0.01 | -1.56 | + |  |
| 20 | Tb927.3.3750 | PFC7 | 19.8 | 5.3 | 4 | DLYEVFDVHQSGGVER | 307.15 | 100 | 96.18 | 100 | 0.31 ± 0.07 | 0.00 | 0.40 ± 0.11 | 0.00 | -1.47 | + | + |
| 21 | Tb11.01.6780 | chaperone protein DNAJ, putative | 35.3 | 5.2 | 4 | APSIEVQLPVTLEDVYYGAVR | 223.88 | 100 | 69.17 | 99.99 | 0.30 ± 0.09 | 0.00 | 0.46 ± 0.22 | 0.01 | -1.4 | + |  |
| 22 | Tb927.6.4520 | hypothetical protein, conserved | 52.5 | 8.1 | 2 | AQLLEEYVPTLGQHVPAR | 150.34 | 100 | 88.93 | 100 | 0.33 ± 0.10 | 0.03 | 0.43 ± 0.11 | 0.04 | -1.4 | + |  |
| 23 | Tb09.160.3590 | PDEB1 | 103.6 | 5.7 | 4 | SDVILDVVTVLSNTDIR | 314.15 | 100 | 120.53 | 100 | 0.34 ± 0.11 | 0.00 | 0.46 ± 0.08 | 0.00 | -1.32 | + |  |
| 24 | Tb927.7.6910 | hypothetical protein, conserved | 92.2 | 4.6 | 8 | YLNDMDDEEELER | 439.48 | 100 | 84.99 | 100 | 0.38 ± 0.12 | 0.00 | 0.43 ± 0.19 | 0.00 | -1.32 | + |  |
| 25 | Tb10.70.4840 | hypothetical protein, conserved | 38.5 | 4.7 | 1 | IALLQEQR | 46.74 | 99.75 | 46.74 | 99.75 | 0.48 ± 0 | NA | 0.33 ± 0 | NA | -1.29 |  |  |
| 26 | Tb11.01.3000 | PFC17 | 41.9 | 5.2 | 2 | QVEDEWASESMPFLEMCR | 174.16 | 100 | 101.05 | 100 | 0.43 ± 0.01 | 0.00 | 0.39 ± 0.05 | 0.02 | -1.29 |  | + |
| 27 | Tb927.8.6660 | PFC1 | 69 | 5.2 | 15 | SIDVSNNPLVTVCSASPIIR | 878.38 | 100 | 113.5 | 100 | 0.37 ± 0.10 | 0.00 | 0.46 ± 0.14 | 0.00 | -1.29 | + | + |
| 28 | Tb11.01.6510 | PFC9 | 41 | 3.8 | 7 | ETESEEVVKEEVLR | 406.38 | 100 | 100.69 | 100 | 0.43 ± 0.06 | 0.00 | 0.42 ± 0.08 | 0.00 | -1.25 | + | + |
| 29 | Tb927.4.1040 | hypothetical protein, conserved | 19.3 | 9.7 | 1 | EIQEYVGR | 34.69 | 96.03 | 34.69 | 96.03 | 0.40 ± 0 | NA | 0.48 ± 0 | NA | -1.18 | + |  |
| 30 | Tb10.70.5560 | hypothetical protein, conserved | 34.5 | 7.1 | 2 | LFLPIAQQQQQQQQQQQQQQHMQK | 262.96 | 100 | 141.76 | 100 | 0.41 ± 0.05 | 0.02 | 0.47 ± 0.07 | 0.03 | -1.18 | + |  |
| 31 | Tb11.02.4230 | hypothetical protein, conserved | 95.7 | 4.9 | 1 | FAGDEGLYAAIR | 42.67 | 99.37 | 42.67 | 99.37 | 0.42 ± 0 | NA | 0.47 ± 0 | NA | -1.15 | + |  |
| 32 | Tb927.8.810 | radial spoke protein RSP9, putative | 37.5 | 4.3 | 4 | VLETLSEDQVAFCDQLR | 283.87 | 100 | 84.31 | 100 | 0.43 ± 0.09 | 0.00 | 0.48 ± 0.19 | 0.01 | -1.15 | + |  |
| 33 | Tb927.8.3790 | PFC2 | 25.1 | 5.8 | 3 | GTLEILHIDNNYSLGSLPSAIGELGR | 181.97 | 100 | 139.67 | 100 | 0.41 ± 0.08 | 0.00 | 0.50 ± 0.15 | 0.01 | -1.12 | + | + |
| 34 | Tb927.5.1880 | inhibitor of serine peptidase (ISP), putative | 17.8 | 5 | 1 | VMEDGLATGTVSGVVR | 85.31 | 100 | 85.31 | 100 | 0.38 ± 0 | NA | 0.54 ± 0 | NA | -1.12 | + |  |
| 35 | Tb11.1220 | hypothetical protein | 57.7 | 10.4 | 10 | VGEKFETAEDSEGLTGGFQFR | 523.5 | 100 | 88.21 | 100 | 0.46 ± 0.14 | 0.00 | 0.48 ± 0.06 | 0.00 | -1.09 |  |  |
| 36 | Tb11.47.0006 | hypothetical protein, conserved | 86.8 | 5.9 | 15 | YAPEMLPTATQTQK | 844.96 | 100 | 79.11 | 99.99 | 0.42 ± 0.15 | 0.00 | 0.53 ± 0.14 | 0.00 | -1.06 | + |  |
| 37 | Tb11.02.2060 | radial spoke protein RSP4/6, putative | 66.9 | 4 | 2 | IAAWTVR | 58.11 | 99.98 | 35.61 | 96.79 | 0.51 ± 0.03 | 0.01 | 0.45 ± 0.05 | 0.02 | -1.06 | + |  |
| 38 | Tb11.01.5100 | Par1 | 68.3 | 5.2 | 21 | GVTGVINALNATQDAGEQLFLSVEK | 1522.05 | 100 | 198.07 | 100 | 0.44 ± 0.08 | 0.00 | 0.54 ± 0.16 | 0.00 | -1.03 | + | + |
| 39 | Tb11.01.3550 | 2-oxoglutarate dehydrogenase E2 component, putative | 41.1 | 8.1 | 1 | ASPTEAAPKPAPAAAPVTSR | 97.91 | 100 | 97.91 | 100 | 0.40 ± 0 | NA | 0.58 ± 0 | NA | -1.03 |  |  |
| 40 | Tb927.8.1550 | PFC3 | 88.1 | 5.5 | 14 | DGPATEGAPSTMNTVDAK | 1178.28 | 100 | 155.93 | 100 | 0.46 ± 0.16 | 0.00 | 0.53 ± 0.08 | 0.00 | -1.03 | + | + |
| 41 | Tb927.6.1400 | PPIase, putative | 28.7 | 4.8 | 1 | LVFELFDDVVPR | 47.89 | 99.81 | 47.89 | 99.81 | 0.40 ± 0 | NA | 0.59 ± 0 | NA | -1 | + |  |
| 42 | Tb927.1.2400 | alpha tubulin | 49.7 | 4.7 | 17 | AYHEQLSVSEISNAVFEPASMMTK | 1682.13 | 100 | 217.76 | 100 | 0.42 ± 0.11 | 0.00 | 0.57 ± 0.26 | 0.00 | -1 | + |  |
| 43 | Tb10.6k15.1510 | PFC18 | 80 | 5 | 3 | QFDLMQYGMVLDEVER | 196.92 | 100 | 121.39 | 100 | 0.48 ± 0.07 | 0.00 | 0.53 ± 0.15 | 0.02 | -1 | + | + |
| 44 | Tb05.5K5.40 | hypothetical protein, conserved | 15 | 5.2 | 4 | GTLMAVEQEPEPNHEADGGK | 271.05 | 100 | 108.17 | 100 | 0.50 ± 0.14 | 0.00 | 0.51 ± 0.07 | 0.00 | -1 |  |  |
| 45 | Tb927.1.2670 | axoneme central apparatus protein | 56.1 | 6.6 | 4 | QIIQVFEEYQR | 163.14 | 100 | 61.33 | 99.99 | 0.40 ± 0.05 | 0.00 | 0.61 ± 0.21 | 0.02 | -1 | + |  |
| 46 | Tb11.18.0003 | dynein intermediate chain, putative | 90.4 | 5.9 | 1 | IGGEDFPWVPLLR | 36.49 | 97.38 | 36.49 | 97.38 | 0.51 ± 0 | NA | 0.50 ± 0 | NA | -1 | + |  |
| 47 | Tb927.2.5760 | hypothetical protein, conserved | 330 | 6.3 | 1 | EVFIQDVAEALR | 58.5 | 99.98 | 58.5 | 99.98 | 0.37 ± 0 | NA | 0.64 ± 0 | NA | -0.97 |  |  |
| 48 | Tb11.01.2800 | hypothetical protein, conserved | 41.6 | 10 | 11 | AYDSALLATNAEFAR | 628.13 | 100 | 100.51 | 100 | 0.45 ± 0.10 | 0.00 | 0.58 ± 0.07 | 0.00 | -0.94 | + |  |
| 49 | Tb927.2.2160 | PFC11 | 37.7 | 5 | 7 | SSSTYVPDAVFENLIER | 304.49 | 100 | 84.85 | 100 | 0.46 ± 0.14 | 0.00 | 0.57 ± 0.17 | 0.00 | -0.94 |  | + |
| 50 | Tb11.02.2210 | PKA-R | 56.7 | 5.1 | 3 | LYNTDLNEDSVR | 72.04 | 99.99 | 37.73 | 98.03 | 0.44 ± 0.07 | 0.00 | 0.6 ± 0.11 | 0.01 | -0.94 |  |  |
| 51 | Tb11.01.7750 | dynein docking complex 2 (ODA1) protein, putative | 72.1 | 5.3 | 1 | TALSEQKEEYDTAVR | 87.13 | 100 | 87.13 | 100 | 0.46 ± 0 | NA | 0.6 ± 0 | NA | -0.92 | + |  |
| 52 | Tb927.5.1230 | hypothetical protein, conserved | 58.5 | 10 | 3 | EMSEFNEQLR | 104.09 | 100 | 62.79 | 99.99 | 0.51 ± 0.18 | 0.02 | 0.59 ± 0.16 | 0.02 | -0.86 | + |  |
| 53 | Tb927.8.5440 | Tb-24 | 25.4 | 4.4 | 1 | GVGEEDLVEFLEFR | 94.67 | 100 | 94.67 | 100 | 0.42 ± 0 | NA | 0.69 ± 0 | NA | -0.84 |  |  |
| 54 | Tb11.01.6870 | calpain-like cysteine peptidase, putative | 33.1 | 4.8 | 1 | ALLGNWFEEEAYMR | 83.16 | 99.99 | 83.16 | 99.99 | 0.52 ± 0 | NA | 0.6 ± 0 | NA | -0.84 | + |  |
| 55 | Tb927.3.1900 | hypothetical protein, conserved | 95.7 | 4.4 | 1 | ETLNSDSSRPATPQK | 80.81 | 99.99 | 80.81 | 99.99 | 0.57 ± 0 | NA | 0.57 ± 0 | NA | -0.81 | + |  |
| 56 | Tb927.3.2310 | PACRGA | 33.7 | 9.9 | 3 | QEKPSIPIEGPVAVQGVR | 223 | 100 | 101.03 | 100 | 0.52 ± 0.08 | 0.00 | 0.63 ± 0.12 | 0.02 | -0.79 | + |  |
| 57 | Tb927.7.3740 | hypothetical protein, conserved | 93.2 | 4.5 | 7 | ILSEEHDGGGVADVLMGFR | 379.34 | 100 | 111.08 | 100 | 0.47 ± 0.30 | 0.00 | 0.7 ± 0.26 | 0.01 | -0.79 | + |  |
| 58 | Tb10.70.2280 | hypothetical protein, conserved | 55.2 | 9 | 1 | LIAEMLDTFAR | 43.93 | 99.53 | 43.93 | 99.53 | 0.67 ± 0 | NA | 0.51 ± 0 | NA | -0.76 |  |  |
| 59 | Tb927.5.2850 | radial spoke protein RSP2, putative | 57.5 | 4.4 | 1 | AFNPVGSYQR | 58.14 | 99.98 | 58.14 | 99.98 | 0.55 ± 0 | NA | 0.63 ± 0 | NA | -0.76 | + |  |
| 60 | Tb10.406.0550 | hypothetical protein, conserved | 20.1 | 10.1 | 1 | LSKPVNQPTVDDAMVDR | 84.71 | 100 | 84.71 | 100 | 0.53 ± 0 | NA | 0.65 ± 0 | NA | -0.76 |  |  |
| 61 | Tb10.6k15.1830 | TbCentrin3 | 18.8 | 4.5 | 1 | SGVSSNLILPEFEAILR | 94.47 | 100 | 94.47 | 100 | 0.49 ± 0 | NA | 0.69 ± 0 | NA | -0.76 |  |  |
| 62 | Tb09.211.0775 | hypothetical protein, conserved | 9.5 | 4.8 | 1 | QVIDTLTR | 34.91 | 96.23 | 34.91 | 96.23 | 0.61 ± 0 | NA | 0.58 ± 0 | NA | -0.76 |  |  |
| 63 | Tb10.70.0480 | trypanin | 53.9 | 6.8 | 2 | TLELIATEVDEWLQR | 93.21 | 100 | 51.36 | 99.91 | 0.46 ± 0.06 | 0.01 | 0.73 ± 0.07 | 0.06 | -0.74 | + |  |
| 64 | Tb09.211.2540 | calmodulin-like protein, putative | 15.8 | 4.6 | 1 | AAGMNPSEEK | 77.3 | 99.99 | 77.3 | 99.99 | 0.53 ± 0 | NA | 0.69 ± 0 | NA | -0.71 | + |  |
| 65 | Tb09.211.1370 | glyceraldehyde-3-phosphate dehydrogenase, putative | 38.8 | 5.7 | 3 | IQPIICSGAPLAVALAPFIR | 65.87 | 99.99 | 43.09 | 99.43 | 0.54 ± 0.27 | 0.04 | 0.7 ± 0.07 | 0.01 | -0.69 | + |  |
| 66 | Tb09.211.1790 | TAX-1 | 40.6 | 6.7 | 1 | VGAVIEQLLTR | 55.98 | 99.97 | 55.98 | 99.97 | 0.62 ± 0 | NA | 0.63 ± 0 | NA | -0.69 | + |  |
| 67 | Tb927.8.2630 | kinesin, putative | 85.1 | 9.5 | 1 | AEVTGGYFQER | 34.24 | 95.6 | 34.24 | 95.6 | 0.53 ± 0 | NA | 0.73 ± 0 | NA | -0.67 | + |  |
| 68 | Tb927.5.4480 | paraflagellar rod component Par4, putative | 68.3 | 4.9 | 13 | NVALMQMLNAQIEENKR | 788.92 | 100 | 129.58 | 100 | 0.61 ± 0.18 | 0.00 | 0.66 ± 0.28 | 0.00 | -0.64 | + |  |
| 69 | Tb11.02.1260 | hypothetical protein, conserved | 28.9 | 5.7 | 1 | MLVHDVPNR | 38.25 | 98.25 | 38.25 | 98.25 | 0.61 ± 0 | NA | 0.67 ± 0 | NA | -0.64 | + |  |
| 70 | Tb09.211.0890 | hypothetical protein, conserved | 19.8 | 7.7 | 1 | LANIFQIAALR | 68.62 | 99.99 | 68.62 | 99.99 | 0.64 ± 0 | NA | 0.64 ± 0 | NA | -0.64 | + |  |
| 71 | Tb09.211.4513 | KMP-11 | 11 | 6.5 | 5 | FFADKPDEATLSPEMK | 324.33 | 100 | 127.92 | 100 | 0.57 ± 0.04 | 0.00 | 0.72 ± 0.09 | 0.00 | -0.64 |  | + |
| 72 | Tb11.01.2670 | MENG | 99.7 | 6.8 | 1 | LLTELTNILAR | 67.04 | 99.99 | 68.46 | 99.99 | 0.58 ± 0 | NA | 0.71 ± 0 | NA | -0.62 | + |  |
| 73 | Tb10.70.7560 | TAX-2 | 25.5 | 4.7 | 3 | VLDITPLIQMYQGTR | 81 | 99.99 | 51.31 | 99.91 | 0.60 ± 0.18 | 0.03 | 0.69 ± 0.13 | 0.03 | -0.62 | + |  |
| 74 | Tb11.01.8650 | hypothetical protein, conserved | 228.8 | 11 | 3 | EATARPGDVGSQGK | 125.25 | 100 | 66.67 | 99.99 | 0.61 ± 0.15 | 0.02 | 0.69 ± 0.05 | 0.00 | -0.62 |  |  |
| 75 | Tb927.7.4570 | nucleoside hydrolase, putative | 39.3 | 4.5 | 2 | LLDLYDADIPFFR | 137.23 | 100 | 81.72 | 99.99 | 0.62 ± 0.26 | 0.14 | 0.68 ± 0.27 | 0.17 | -0.62 |  |  |
| 76 | Tb10.70.5350 | hypothetical protein, conserved | 118.7 | 5.8 | 1 | LQHPGLANISLTR | 41.13 | 99.1 | 41.13 | 99.1 | 0.59 ± 0 | NA | 0.71 ± 0 | NA | -0.62 | + |  |
| 77 | Tb11.02.0990 | hypothetical protein, conserved | 115.1 | 4.8 | 5 | ISGETGSTEEK | 124.47 | 100 | 68.45 | 99.99 | 0.60 ± 0.14 | 0.00 | 0.72 ± 0.18 | 0.01 | -0.6 | + |  |
| 78 | Tb927.5.1690 | hypothetical protein, conserved | 51.6 | 7.7 | 1 | LHPDATGLETETSDGQGGQR | 127.38 | 100 | 127.38 | 100 | 0.65 ± 0 | NA | 0.67 ± 0 | NA | -0.6 |  |  |
| 79 | Tb11.57.0008 | calpain-like protein, putative | 715.2 | 4.7 | 1 | FSTMEEER | 29.58 | 87.12 | 38 | 98.15 | 0.61 ± 0 | NA | 0.72 ± 0 | NA | -0.6 |  |  |
| 80 | Tb09.160.5560 | adenylosuccinate lyase, putative | 53.1 | 7.1 | 1 | ELSSNWAVVAEGIQTVLR | 61.38 | 99.99 | 61.38 | 99.99 | 0.51 ± 0 | NA | 0.82 ± 0 | NA | -0.6 |  |  |
| 81 | Tb11.02.2130 | hypothetical protein, conserved | 35.6 | 10 | 1 | AHEIEETIAQGR | 58.57 | 99.98 | 58.57 | 99.98 | 0.52 ± 0 | NA | 0.81 ± 0 | NA | -0.58 | + |  |
| 82 | Tb11.01.4390 | leucine-rich repeat protein (LRRP), putative | 70 | 5.1 | 1 | GLEIIATTLPQLIR | 44.72 | 99.61 | 44.72 | 99.61 | 0.57 ± 0 | NA | 0.77 ± 0 | NA | -0.58 | + |  |
| 83 | Tb927.5.1900 | hypothetical protein, conserved | 77.3 | 7.1 | 4 | VQEGLGQLLMDLGAGVQR | 283.71 | 100 | 104.75 | 100 | 0.55 ± 0.17 | 0.00 | 0.79 ± 0.32 | 0.14 | -0.58 | + |  |
| 84 | Tb10.6k15.0710 | hypothetical protein, conserved | 39.2 | 7.8 | 1 | ELELHQNIVR | 39.52 | 98.69 | 39.52 | 98.69 | 0.58 ± 0 | NA | 0.76 ± 0 | NA | -0.58 | + |  |
| 85 | Tb927.8.6240 | hypothetical protein, conserved | 30 | 8.9 | 8 | QEAWEPLEGK | 539.68 | 100 | 124.89 | 100 | 0.62 ± 0.06 | 0.00 | 0.73 ± 0.09 | 0.00 | -0.56 | + |  |
| 86 | Tb927.2.4330 | PFR5 | 87.1 | 6.4 | 2 | VPGTHGAGGEEPDR | 100.91 | 100 | 72.97 | 99.99 | 0.54 ± 0.02 | 0.00 | 0.82 ± 0.04 | 0.05 | -0.56 | + | + |
| 87 | Tb927.8.4640 | flagellar protofilament ribbon protein, putative | 46.8 | 9 | 5 | IYDIEQGIAEQR | 238.24 | 100 | 67.56 | 99.99 | 0.63 ± 0.03 | 0.00 | 0.74 ± 0.13 | 0.01 | -0.56 | + |  |
| 88 | Tb927.1.2390 | beta tubulin | 49.7 | 4.5 | 18 | FWEVISDEHGVDPTGTYQGDSDLQLER | 1442.47 | 100 | 185.97 | 100 | 0.59 ± 0.14 | 0.00 | 0.79 ± 0.12 | 0.00 | -0.54 | + |  |
| 89 | Tb09.211.3955 | hypothetical protein, conserved | 9.2 | 4.4 | 1 | ELTTFVQGLLQNMQTR | 87.94 | 100 | 87.94 | 100 | 0.64 ± 0 | NA | 0.74 ± 0 | NA | -0.54 |  |  |
| 90 | Tb927.8.4870 | DIGIT | 136.4 | 6.9 | 1 | SFGEFQSR | 36.4 | 97.32 | 36.4 | 97.32 | 0.64 ± 0 | NA | 0.74 ± 0 | NA | -0.54 | + |  |
| 91 | Tb11.01.3010 | dynein heavy chain, putative | 480.2 | 6 | 4 | FVAEDLPLFR | 63.02 | 99.99 | 37.2 | 97.77 | 0.69 ± 0.16 | 0.02 | 0.71 ± 0.06 | 0.00 | -0.51 | + |  |
| 92 | Tb927.4.4690 | hypothetical protein, conserved | 31.3 | 9.3 | 4 | QAMAASEGQLK | 176.07 | 100 | 72.76 | 99.99 | 0.63 ± 0.08 | 0.00 | 0.77 ± 0.17 | 0.04 | -0.51 | + |  |
| 93 | Tb11.01.0390 | dynein heavy chain, putative | 473.8 | 5.4 | 1 | YLPAEGIYEFMNSDFR | 63.37 | 99.99 | 64.79 | 99.99 | 0.63 ± 0 | NA | 0.77 ± 0 | NA | -0.51 | + |  |
| 94 | Tb927.7.6890 | hypothetical protein, conserved | 28.7 | 5.6 | 1 | YANPDYWEER | 43.72 | 99.5 | 43.72 | 99.5 | 0.73 ± 0 | NA | 0.68 ± 0 | NA | -0.51 | + |  |
| 95 | Tb10.70.4610 | hypothetical protein, conserved | 206.8 | 7.5 | 1 | GSAPQEYPHTTSASPTR | 55.89 | 99.97 | 55.89 | 99.97 | 0.65 ± 0 | NA | 0.76 ± 0 | NA | -0.49 | + |  |
| 96 | Tb10.6k15.3460 | hypothetical protein, conserved | 284.7 | 5.2 | 15 | DLWCGTGDFAPDPLVR | 232.59 | 100 | 60.53 | 99.99 | 0.59 ± 0.19 | 0.00 | 0.83 ± 0.29 | 0.02 | -0.49 |  |  |
| 97 | Tb09.211.1470 | PACRGB | 35.3 | 10 | 4 | GDPATKPASADVQK | 169.09 | 100 | 89.72 | 100 | 0.67 ± 0.26 | 0.04 | 0.75 ± 0.18 | 0.03 | -0.49 | + |  |
| 98 | Tb11.01.6840 | hypothetical protein, conserved | 77.3 | 6 | 1 | YSNLEQDAAVR | 33.73 | 95.05 | 33.73 | 95.05 | 0.61 ± 0 | NA | 0.81 ± 0 | NA | -0.49 | + |  |
| 99 | Tb10.6k15.1760 | hypothetical protein, conserved | 244.8 | 4.6 | 1 | ECTDADAPISLATLAALVR | 120.49 | 100 | 120.49 | 100 | 0.67 ± 0 | NA | 0.76 ± 0 | NA | -0.47 |  |  |
| 100 | Tb10.70.7320 | hypothetical protein, conserved | 195.4 | 5.9 | 1 | EEQQSTKPPGPR | 66.67 | 99.99 | 66.67 | 99.99 | 0.68 ± 0 | NA | 0.75 ± 0 | NA | -0.47 |  |  |
| 101 | Tb927.3.1200 | hypothetical protein, conserved | 83.7 | 8.6 | 3 | DTLTVDLPLFLR | 99.52 | 100 | 68.3 | 99.99 | 0.62 ± 0.02 | 0.00 | 0.84 ± 0.04 | 0.01 | -0.45 |  |  |
| 102 | Tb927.8.3250 | dynein heavy chain, putative | 537.3 | 6.2 | 1 | VAQDQTDDADAGKR | 84.6 | 100 | 84.6 | 100 | 0.65 ± 0 | NA | 0.83 ± 0 | NA | -0.43 | + |  |
| 103 | Tb927.4.870 | dynein heavy chain, putative | 510.1 | 6 | 8 | GLIGDLFPGLDPTR | 180.84 | 100 | 85.05 | 100 | 0.70 ± 0.19 | 0.00 | 0.79 ± 0.10 | 0.00 | -0.42 | + |  |
| 104 | Tb927.5.2950 | hypothetical protein, conserved | 88.2 | 6.5 | 5 | SVQGKPQQLEEEFR | 159.52 | 100 | 74.07 | 99.99 | 0.73 ± 0.19 | 0.02 | 0.77 ± 0.24 | 0.05 | -0.42 | + |  |
| 105 | Tb10.61.2210 | hypothetical protein, conserved | 37 | 7.6 | 5 | DNAAALGAVLHR | 250.5 | 100 | 84.97 | 100 | 0.71 ± 0.09 | 0.00 | 0.79 ± 0.07 | 0.00 | -0.42 | + |  |
| 106 | Tb09.211.2250 | hypothetical protein, conserved | 31.5 | 4.7 | 1 | SDTSLLSIDQYMDVLR | 97.7 | 100 | 97.7 | 100 | 0.72 ± 0 | NA | 0.79 ± 0 | NA | -0.42 | + |  |
| 107 | Tb10.61.2220 | hypothetical protein, conserved | 46.2 | 7.7 | 13 | LHDQSVGTDGQELVVEGK | 830.32 | 100 | 148.38 | 100 | 0.67 ± 0.28 | 0.00 | 0.85 ± 0.15 | 0.00 | -0.4 | + |  |
| 108 | Tb11.02.1500 | hypothetical protein, conserved | 62.9 | 9.8 | 5 | LLPLVALAQLR | 96.18 | 100 | 44.34 | 99.57 | 0.73 ± 0.12 | 0.00 | 0.79 ± 0.14 | 0.01 | -0.4 |  |  |
| 109 | Tb927.7.6970 | paraflagellar rod protein, putative | 77.2 | 6.3 | 1 | GGDSTGGEAGGTAAGPAQEAIR | 73.3 | 99.99 | 73.3 | 99.99 | 0.70 ± 0 | NA | 0.83 ± 0 | NA | -0.4 | + |  |
| 110 | Tb927.6.4710 | calmodulin, putative | 74.2 | 4.3 | 1 | ISEWAANQR | 37.56 | 97.95 | 37.56 | 97.95 | 0.63 ± 0 | NA | 0.90 ± 0 | NA | -0.38 | + |  |
| 111 | Tb927.8.6230 | hypothetical protein, conserved | 37.2 | 9.6 | 3 | KPPVNQDESPANDQR | 192.46 | 100 | 90.12 | 100 | 0.76 ± 0.06 | 0.01 | 0.77 ± 0.03 | 0.00 | -0.38 | + |  |
| 112 | Tb11.02.0760 | dynein heavy chain, putative | 531.1 | 5.2 | 10 | SIVNSFIANVGAGAGLIKPLVDGR | 236.55 | 100 | 117.43 | 100 | 0.69 ± 0.23 | 0.00 | 0.84 ± 0.19 | 0.01 | -0.38 | + |  |
| 113 | Tb10.6k15.2670 | hypothetical protein, conserved | 76.8 | 5.3 | 2 | EVESTYEQER | 101.23 | 100 | 50.87 | 99.9 | 0.72 ± 0.29 | 0.07 | 0.83 ± 0.32 | 0.29 | -0.38 | + |  |
| 114 | Tb10.26.0070 | 33 kDa inner dynein arm light chain, axonemal, putative | 42 | 7.9 | 3 | VTDVETLLYTLLPPQR | 193.24 | 100 | 107.97 | 100 | 0.69 ± 0.10 | 0.02 | 0.86 ± 0.04 | 0.01 | -0.36 | + |  |
| 115 | Tb09.160.2070 | cyclophilin type peptidyl-prolyl cis-trans isomerase, putative | 35.7 | 5.3 | 1 | YFPNESYAIPHDR | 37.53 | 97.94 | 37.53 | 97.94 | 0.70 ± 0 | NA | 0.85 ± 0 | NA | -0.36 | + |  |
| 116 | Tb10.6k15.2920 | hypothetical protein, conserved | 82.8 | 6.8 | 1 | FAATWEQR | 37.21 | 97.78 | 37.21 | 97.78 | 0.77 ± 0 | NA | 0.79 ± 0 | NA | -0.36 | + |  |
| 117 | Tb09.244.2800 | trypanin-related protein, putative | 54.1 | 9.4 | 3 | AIAEQQLSFER | 131.53 | 100 | 57.36 | 99.98 | 0.68 ± 0.14 | 0.03 | 0.89 ± 0.22 | 0.24 | -0.36 | + |  |
| 118 | Tb927.4.4700 | hypothetical protein, conserved | 30.7 | 9.1 | 6 | EAQLSEDHEEPK | 282.04 | 100 | 77.46 | 99.99 | 0.72 ± 0.12 | 0.00 | 0.85 ± 0 | NA | -0.36 | + |  |
| 119 | Tb11.02.1190 | hypothetical protein, conserved | 61.1 | 7.3 | 5 | AMLIELSGQHQEEER | 241.28 | 100 | 101.14 | 100 | 0.73 ± 0.16 | 0.01 | 0.85 ± 0.13 | 0.03 | -0.34 | + |  |
| 120 | Tb09.211.0170 | hypothetical protein, conserved | 28.8 | 7.3 | 2 | GQMDFTSTTVLR | 91.2 | 100 | 64.3 | 99.99 | 0.84 ± 0.17 | 0.21 | 0.74 ± 0.06 | 0.05 | -0.34 | + |  |
| 121 | Tb09.160.4520 | calmodulin, putative | 17.8 | 4.5 | 3 | QLLSEVHTNEDGR | 128.09 | 100 | 73 | 99.99 | 0.66 ± 0.11 | 0.02 | 0.92 ± 0.19 | 0.27 | -0.34 | + |  |
| 122 | Tb927.7.6280 | hypothetical protein, conserved | 72 | 7.8 | 1 | SQVPAFASQPPEEK | 37.85 | 98.08 | 37.85 | 98.08 | 0.67 ± 0 | NA | 0.92 ± 0 | NA | -0.34 | + |  |
| 123 | Tb11.01.1210 | hypothetical protein, conserved | 61.7 | 6.8 | 2 | YSPGVLVGNWYEDMR | 110.96 | 100 | 75.09 | 99.99 | 0.61 ± 0.03 | 0.02 | 1.00 ± 0.08 | 0.5 | -0.32 | + |  |
| 124 | Tb927.6.5030 | hypothetical protein, conserved | 64.8 | 6.2 | 2 | EHLMPIDEEDQR | 73.02 | 99.99 | 43.36 | 99.46 | 0.79 ± 0.03 | 0.03 | 0.83 ± 0.03 | 0.04 | -0.3 | + |  |
| 125 | Tb11.0845 | dynein light chain, putative | 10.4 | 6.7 | 2 | NADMPEDMQSDAVEVALQALEK | 215.73 | 100 | 166.05 | 100 | 0.74 ± 0.12 | 0.10 | 0.88 ± 0.03 | 0.06 | -0.3 | + |  |
| 126 | Tb927.6.570 | hypothetical protein, conserved | 11.8 | 5.8 | 1 | YTFTASEHPSTPGVYR | 38.44 | 98.33 | 38.44 | 98.33 | 0.68 ± 0 | NA | 0.94 ± 0 | NA | -0.3 | + |  |
| 127 | Tb927.1.990 | hypothetical protein, conserved | 42.2 | 10 | 4 | TQGAMEPADQQQK | 124.05 | 100 | 102.5 | 100 | 0.78 ± 0.11 | 0.01 | 0.85 ± 0.11 | 0.04 | -0.29 |  |  |
| 128 | Tb11.01.2310 | hypothetical protein, conserved | 99.4 | 5.5 | 6 | AYEDLENQFNEAK | 92.95 | 100 | 50.79 | 99.9 | 0.89 ± 0.04 | 0.00 | 0.74 ± 0.05 | 0.00 | -0.29 | + |  |
| 129 | Tb11.47.0034 | radial spoke protein RSP3, putative | 39.3 | 6 | 2 | GNTYAAVPMSTYAR | 127.81 | 100 | 74.82 | 99.99 | 0.76 ± 0.02 | 0.02 | 0.88 ± 0.01 | 0.02 | -0.29 | + |  |
| 130 | Tb927.2.4810 | hypothetical protein, conserved | 150.5 | 5.8 | 2 | LEAGRPEPR | 64.86 | 99.99 | 36.47 | 97.36 | 0.80 ± 0.15 | 0.16 | 0.86 ± 0.06 | 0.03 | -0.27 |  |  |
| 131 | Tb927.8.4580 | hypothetical protein, conserved | 58 | 7.5 | 9 | TLLDEQYDEVK | 389.42 | 100 | 63.61 | 99.99 | 0.73 ± 0.20 | 0.00 | 0.92 ± 0.23 | 0.16 | -0.27 | + |  |
| 132 | Tb09.160.3930 | hypothetical protein, conserved | 34.1 | 7.4 | 2 | GPVTGPIQMVDFLR | 107.3 | 100 | 67.69 | 99.99 | 0.79 ± 0.39 | 0.29 | 0.88 ± 0.42 | 0.38 | -0.25 | + |  |
| 133 | Tb927.8.8200 | hypothetical protein, conserved | 99.8 | 7.2 | 3 | QTQMQAEQADVQER | 140.26 | 100 | 69.45 | 99.99 | 0.72 ± 0.12 | 0.03 | 0.96 ± 0.10 | 0.28 | -0.25 | + |  |
| 134 | Tb927.3.930 | dynein heavy chain, putative | 531.4 | 6.4 | 10 | VTAEVDRYNQQLSEENAK | 175.98 | 100 | 65.68 | 99.99 | 0.75 ± 0.23 | 0.00 | 0.93 ± 0.20 | 0.14 | -0.25 | + |  |
| 135 | Tb927.7.4910 | hypothetical protein, conserved | 40.4 | 9.3 | 3 | IQFSCGSFESFVR | 146.11 | 100 | 72.56 | 99.99 | 0.79 ± 0.15 | 0.07 | 0.93 ± 0.11 | 0.19 | -0.22 | + |  |
| 136 | Tb927.7.3310 | hypothetical protein, conserved | 38.5 | 6.5 | 2 | EVMPVLPLQALLGGER | 91.1 | 100 | 68.06 | 99.99 | 0.86 ± 0.38 | 0.35 | 0.88 ± 0.51 | 0.4 | -0.2 |  |  |
| 137 | Tb11.02.2530 | hypothetical protein, conserved | 30.7 | 9.7 | 5 | VATEAYTEAFR | 71.87 | 99.99 | 40.59 | 98.98 | 0.81 ± 0.04 | 0.00 | 0.93 ± 0.03 | 0.00 | -0.2 | + |  |
| 138 | Tb927.8.5830 | hypothetical protein, conserved | 36.5 | 4 | 1 | STFYVGNVFAQLQGGMGPR | 72.15 | 99.99 | 72.15 | 99.99 | 0.62 ± 0 | NA | 1.13 ± 0 | NA | -0.2 |  |  |
| 139 | Tb927.4.2600 | hypothetical protein, conserved | 181.3 | 6.7 | 3 | NTEKPEASATNTSVR | 124.14 | 100 | 57.47 | 99.98 | 0.87 ± 0.19 | 0.10 | 0.88 ± 0.20 | 0.20 | -0.18 | + |  |
| 140 | Tb11.02.0170 | hypothetical protein, conserved | 63.1 | 6.2 | 1 | LEDDFQGAR | 38.72 | 98.43 | 40.14 | 98.87 | 0.81 ± 0 | NA | 0.96 ± 0 | NA | -0.17 |  |  |
| 141 | Tb11.01.4400 | hypothetical protein | 83.7 | 4.8 | 4 | GNPMANTEHDR | 128.45 | 100 | 44.22 | 99.56 | 0.87 ± 0.18 | 0.12 | 0.91 ± 0.34 | 0.32 | -0.17 | + |  |
| 142 | Tb11.01.8640 | hypothetical protein, conserved | 70.6 | 8.8 | 2 | GLAALVDCFR | 74.89 | 99.99 | 51.77 | 99.92 | 0.98 ± 0.39 | 0.48 | 0.82 ± 0.30 | 0.28 | -0.15 | + |  |
| 143 | Tb927.6.2720 | calcium-binding protein, putative | 41.6 | 5.1 | 1 | GFYMDEAR | 36.32 | 97.27 | 36.32 | 97.27 | 0.99 ± 0 | NA | 0.82 ± 0 | NA | -0.15 | + |  |
| 144 | Tb927.7.4510 | hypothetical protein, conserved | 48.1 | 8.7 | 1 | EEWQQQR | 37.78 | 98.05 | 37.78 | 98.05 | 0.80 ± 0 | NA | 1.02 ± 0 | NA | -0.14 | + |  |
| 145 | Tb11.02.4380 | hypothetical protein, conserved | 45.8 | 9.1 | 3 | SIGGEPQLYAGAK | 91.89 | 100 | 44.35 | 99.57 | 0.95 ± 0.21 | 0.36 | 0.88 ± 0.13 | 0.13 | -0.12 | + |  |
| 146 | Tb09.160.0650 | hypothetical protein, conserved | 502.8 | 5.1 | 3 | TLAPMMMHVDEIGEDFVR | 195.71 | 100 | 90.02 | 100 | 0.86 ± 0.29 | 0.25 | 0.97 ± 0.13 | 0.36 | -0.12 |  |  |
| 147 | Tb927.4.3950 | CAP5.5 | 94.6 | 4.1 | 3 | VAGEFDNEHPNLVLMIR | 213.78 | 100 | 105.38 | 100 | 0.87 ± 0.13 | 0.11 | 0.98 ± 0.20 | 0.44 | -0.1 | + |  |
| 148 | Tb927.3.1670 | hypothetical protein, conserved | 112.5 | 5.3 | 1 | ELLGAVPFSQVPNLFR | 48.66 | 99.84 | 48.66 | 99.84 | 0.76 ± 0 | NA | 1.10 ± 0 | NA | -0.1 | + |  |
| 149 | Tb11.02.0860 | hypothetical protein, conserved | 102.8 | 9.6 | 2 | MEGKDDQGMLQAADVSGSR | 75.21 | 99.99 | 38.34 | 98.29 | 0.91 ± 0.05 | 0.12 | 0.97 ± 0.02 | 0.14 | -0.09 | + |  |
| 150 | Tb927.3.5310 | paraflagellar rod protein | 242.7 | 4.1 | 1 | VSSHNNPPVVR | 36.7 | 97.5 | 36.7 | 97.5 | 0.83 ± 0 | NA | 1.05 ± 0 | NA | -0.09 | + |  |
| 151 | Tb11.01.4030 | hypothetical protein, conserved | 32.4 | 10 | 3 | TQAPFSAAVK | 163.27 | 100 | 62.77 | 99.99 | 0.81 ± 0.16 | 0.09 | 1.08 ± 0.13 | 0.20 | -0.07 | + |  |
| 152 | Tb11.02.4150 | PPDK | 100.4 | 8.8 | 1 | TPQQINHSLSLR | 45.3 | 99.66 | 45.3 | 99.66 | 0.86 ± 0 | NA | 1.06 ± 0 | NA | -0.06 | + |  |
| 153 | Tb927.8.3530 | glycerol-3-phosphate dehydrogenase [NAD+], glycosomal | 37.8 | 8.8 | 8 | NVPVVMCSK | 427.2 | 100 | 64.23 | 99.99 | 0.90 ± 0.09 | 0.01 | 1.07 ± 0.11 | 0.06 | -0.01 | + |  |
| 154 | Tb11.01.3110 | heat shock protein 70 | 75.3 | 6.3 | 2 | FEELCGDLFR | 99.56 | 100 | 67.65 | 99.99 | 0.85 ± 0.26 | 0.28 | 1.19 ± 0.30 | 0.27 | 0.03 | + |  |
| 155 | Tb11.02.0352 | hypothetical protein, conserved | 31.5 | 9.4 | 1 | TDFQPPEQGDYEGIDR | 65.84 | 99.99 | 65.84 | 99.99 | 0.82 ± 0 | NA | 1.26 ± 0 | NA | 0.06 |  |  |
| 156 | Tb927.7.3330 | hypothetical protein, conserved | 502.6 | 4.2 | 4 | LHEMEEQLAELR | 249.15 | 100 | 99 | 100 | 0.84 ± 0.55 | 0.30 | 1.29 ± 0.36 | 0.10 | 0.1 |  |  |
| 157 | Tb927.3.3760 | TRYP1, tryparedoxin | 15.9 | 4.8 | 1 | MPWLALPFDQR | 36.88 | 97.6 | 36.88 | 97.6 | 1.02 ± 0 | NA | 1.16 ± 0 | NA | 0.12 |  |  |
| 158 | Tb10.70.4990 | hypothetical protein, conserved | 13.7 | 5.1 | 1 | AALAEENPEAEGR | 52.59 | 99.94 | 52.59 | 99.94 | 1.13 ± 0 | NA | 1.20 ± 0 | NA | 0.21 | + |  |
| 159 | Tb927.1.2100 | calpain-like cysteine peptidase, putative | 126.1 | 4.5 | 2 | GALSEQAEDGHR | 118.45 | 100 | 75.22 | 99.99 | 1.07 ± 0.05 | 0.15 | 1.29 ± 0.50 | 0.28 | 0.24 | + |  |
| 160 | Tb10.6k15.3080 | dihydrolipoamide acetyltransferase E2 subunit, putative | 48.1 | 7.3 | 1 | NWQAAGSSPAATQSK | 53.44 | 99.95 | 53.44 | 99.95 | 1.12 ± 0 | NA | 1.29 ± 0 | NA | 0.26 |  |  |
| 161 | Tb09.160.4280 | TRYP1, tryparedoxin peroxidase | 22.4 | 6.4 | 9 | AFQFVEK | 463.27 | 100 | 121.96 | 100 | 1.12 ± 0.15 | 0.02 | 1.31 ± 0.23 | 0.00 | 0.29 | + |  |
| 162 | Tb10.26.1080 | heat shock protein 83 | 80.7 | 4.9 | 1 | YQSLTNQSVLGDEPHLR | 83.15 | 99.99 | 83.15 | 99.99 | 1.08 ± 0 | NA | 1.36 ± 0 | NA | 0.29 |  |  |
| 163 | Tb09.160.1160 | NOP86 | 85.9 | 4.4 | 5 | QQMVELDQVR | 286.23 | 100 | 99.87 | 100 | 1.17 ± 0.15 | 0.03 | 1.29 ± 0.18 | 0.01 | 0.3 | + |  |
| 164 | Tb927.3.2110 | TFIIF-stimulated CTD phosphatase, putative | 47.2 | 6.6 | 1 | WSGPEAEVPESAARPR | 41.94 | 99.25 | 41.94 | 99.25 | 1.27 ± 0 | NA | 1.20 ± 0 | NA | 0.31 |  |  |
| 165 | Tb11.01.8770 | hypothetical protein, conserved | 110.1 | 4.3 | 3 | ELTQANYATEYLYLR | 263.22 | 100 | 99.23 | 100 | 1.02 ± 0.17 | 0.43 | 1.48 ± 0.04 | 0.00 | 0.32 | + |  |
| 166 | Tb09.160.1200 | GB4 | 928.3 | 4.3 | 4 | ALGVSPQQVVIIDCR | 189.99 | 100 | 81.53 | 99.99 | 1.37 ± 0.34 | 0.06 | 1.19 ± 0.31 | 0.15 | 0.36 |  |  |
| 167 | Tb927.2.4210 | glycosomal phosphoenolpyruvate carboxykinase | 58.5 | 8.7 | 1 | NLIGDDEHVWTDR | 33.86 | 95.19 | 33.86 | 95.19 | 1.29 ± 0 | NA | 1.51 ± 0 | NA | 0.49 |  |  |
| 168 | Tb10.6k15.3580 | hypothetical protein, conserved | 37.2 | 9.7 | 1 | DYIPTAQPNEYR | 33.69 | 95 | 35.11 | 96.4 | 1.53 ± 0 | NA | 1.27 ± 0 | NA | 0.49 | + |  |
| 169 | Tb927.4.2080 | hypothetical protein, conserved | 104.8 | 5.7 | 8 | HLTNDGLGIVQGEPMR | 525.43 | 100 | 87.26 | 100 | 1.32 ± 0.48 | 0.05 | 1.56 ± 0.44 | 0.00 | 0.53 | + |  |
| 170 | Tb927.5.295b | retrotransposon hotspot (RHS) protein 1 | 95.1 | 8.6 | 1 | EEVDISHTPETMNEPLPR | 39.11 | 98.57 | 39.11 | 98.57 | 1.21 ± 0 | NA | 1.7 ± 0 | NA | 0.55 |  |  |
| 171 | Tb10.70.2650 | elongation factor 2 | 94.3 | 6 | 1 | AYLPVAESFGFTADLR | 66.7 | 99.99 | 66.7 | 99.99 | 1.59 ± 0 | NA | 1.35 ± 0 | NA | 0.56 |  |  |
| 172 | Tb927.7.2980 | hypothetical protein, conserved | 21.2 | 7.3 | 3 | ECPSAFNSQSSR | 136.39 | 100 | 49.26 | 99.86 | 1.30 ± 0.19 | 0.05 | 1.67 ± 0.15 | 0.01 | 0.57 |  |  |
| 173 | Tb927.1.4310 | hypothetical protein, conserved | 183.7 | 9.7 | 10 | SFVADAVQSEIDTLISDEMAAR | 568.39 | 100 | 141.26 | 100 | 1.32 ± 0.44 | 0.02 | 1.66 ± 0.31 | 0.00 | 0.57 | + |  |
| 174 | Tb927.3.4500 | fumarate hydratase, putative | 62.2 | 6.6 | 1 | YGGFYLGSIGGPAAILAR | 59.95 | 99.99 | 61.37 | 99.99 | 1.47 ± 0 | NA | 1.64 ± 0 | NA | 0.63 |  |  |
| 175 | Tb10.6k15.1500 | hypothetical protein, conserved | 45.3 | 9.2 | 13 | SNESVDVLNLGQYSSEQLNEVER | 1050.84 | 100 | 194.85 | 100 | 1.36 ± 0.38 | 0.00 | 1.75 ± 0.51 | 0.00 | 0.63 | + |  |
| 176 | Tb11.02.4170 | 40S ribosomal protein S5, putative | 21.3 | 11 | 1 | TPTYVPHSAGR | 40.24 | 98.9 | 40.24 | 98.89 | 1.60 ± 0 | NA | 1.85 ± 0 | NA | 0.79 |  |  |
| 177 | Tb10.v4.0052 | microtubule-associated protein 2 | NA | NA | 7 | STTAESYAPIDPAAYK | 445.15 | 100 | 122.32 | 100 | 1.68 ± 0.40 | 0.00 | 1.79 ± 0.29 | 0.00 | 0.79 | + |  |
| 178 | Tb10.70.0800 | universal minicircle sequence binding protein (UMSBP), putative | 14.5 | 8.2 | 1 | ECPNAPADAAAGGR | 65.61 | 99.99 | 65.61 | 99.99 | 1.88 ± 0 | NA | 1.61 ± 0 | NA | 0.8 |  |  |
| 179 | Tb927.7.3440 | I/6 autoantigen | 27 | 4.4 | 2 | GYVSVDEFMDALYGEEGR | 158.75 | 100 | 101.24 | 100 | 1.60 ± 0.00 | 0 | 1.89 ± 0.24 | 0.06 | 0.81 | + |  |
| 180 | Tb09.211.3540 | glk1, glycosomal | 27 | 4.4 | 3 | EAMIAEWR | 98.22 | 100 | 43.29 | 99.45 | 1.66 ± 0.14 | 0.01 | 1.95 ± 0.27 | 0.01 | 0.85 |  |  |
| 181 | Tb927.4.2070 | antigenic protein, putative | 511.3 | 4.1 | 5 | AVNEQYETLQR | 288.35 | 100 | 73.67 | 99.99 | 1.75 ± 0.14 | 0.00 | 1.95 ± 0.31 | 0.00 | 0.89 |  |  |
| 182 | Tb927.5.1610 | 60S ribosomal protein L13a, putative | 25.7 | 12 | 5 | SPSDVFIR | 63.04 | 99.99 | 41.82 | 99.23 | 1.67 ± 2.11 | 0.26 | 2.4 ± 1.45 | 0.05 | 1.02 | + |  |
| 183 | Tb10.26.0370 | RPS3,40S ribosomal protein S3, putative | 24.2 | 10.6 | 3 | ELADDGFAGVEHR | 98.1 | 100 | 53.49 | 99.95 | 1.60 ± 0.17 | 0.01 | 2.53 ± 0.02 | 0.00 | 1.05 | + |  |
| 184 | Tb10.389.0690 | mitochondrial carrier protein, putative | 33.1 | 10.6 | 3 | QAVYAPAR | 131.49 | 100 | 51.16 | 99.91 | 1.73 ± 0.17 | 0.01 | 2.41 ± 0.07 | 0.00 | 1.05 | + |  |
| 185 | Tb10.61.1630 | ZC3H40, RNA binding protein, putative | 46.1 | 7.5 | 1 | AAPPDTSPQEATGGER | 65.57 | 99.99 | 65.57 | 99.99 | 2.11 ± 0 | NA | 2.08 ± 0 | NA | 1.06 |  |  |
| 186 | Tb10.70.4780 | hypothetical protein, conserved | 84.3 | 7.4 | 2 | VSVSDGGHQQGEENQR | 126.11 | 100 | 94.32 | 100 | 1.72 ± 0.90 | 0.23 | 2.49 ± 1.49 | 0.20 | 1.07 | + |  |
| 187 | Tb10.70.7695 | 40S ribosomal proteins S11, putative | 20 | 11.5 | 2 | TPTVDLTVQHEK | 98.37 | 100 | 49.8 | 99.88 | 2.17 ± 0.26 | 0.05 | 2.11 ± 0.33 | 0.07 | 1.1 |  |  |
| 188 | Tb10.61.2190 | hypothetical protein, conserved | 51.5 | 9.9 | 2 | GTIHQQAAMAEQQR | 150.48 | 100 | 99.51 | 100 | 2.06 ± 0.39 | 0.02 | 2.29 ± 0.37 | 0.01 | 1.12 |  |  |
| 189 | Tb11.01.5720 | ribosomal protein L18, putative | 20.9 | 11.2 | 5 | NYSVDIAYYSQR | 190.52 | 100 | 78.67 | 99.99 | 1.90 ± 0.76 | 0.03 | 2.49 ± 0.82 | 0.01 | 1.13 |  |  |
| 190 | Tb927.6.4300 | GAPDH, glycosomal | 43.8 | 9.6 | 11 | NILGYTDEELVSADFISDSR | 920.5 | 100 | 161.45 | 100 | 1.97 ± 0.30 | 0.00 | 2.54 ± 0.36 | 0.00 | 1.176 |  |  |
| 191 | Tb11.01.0680 | TbLRRP1 | 78.9 | 8.7 | 7 | DQSYVVPPHTTSTDR | 198.5 | 100 | 53.96 | 99.95 | 2.32 ± 2.04 | 0.07 | 2.33 ± 1.33 | 0.02 | 1.22 | + |  |
| 192 | Tb10.389.0910 | 60S ribosomal protein L34, putative | 19.3 | 12.5 | 1 | AFLIEEQR | 48.13 | 99.82 | 48.13 | 99.82 | 1.71 ± 0 | NA | 3.08 ± 0 | NA | 1.26 | + |  |
| 193 | Tb927.7.2680 | hypothetical protein, conserved | 76.1 | 6.9 | 1 | TVGPQQPTEEQR | 61.13 | 99.99 | 61.13 | 99.99 | 2.29 ± 0 | NA | 2.61 ± 0 | NA | 1.29 |  |  |
| 194 | Tb09.211.0110 | QM, 60S ribosomal protein L10, putative | 24.7 | 11.2 | 6 | YWGFTNILR | 113.48 | 100 | 55.19 | 99.96 | 2.16 ± 0.53 | 0.00 | 2.79 ± 1.26 | 0.01 | 1.3 | + |  |
| 195 | Tb09.211.0350 | adenylate kinase, putative | 22.4 | 7.3 | 1 | NALNTHPSPR | 37.94 | 98.12 | 37.94 | 98.12 | 2.29 ± 0 | NA | 2.67 ± 0 | NA | 1.31 | + |  |
| 196 | Tb10.61.0560 | hypothetical protein, conserved | 197.7 | 7.3 | 9 | VEVATWDSEQQLR | 254.83 | 100 | 87.82 | 100 | 2.22 ± 1.32 | 0.01 | 2.76 ± 1.21 | 0.00 | 1.32 |  |  |
| 197 | Tb927.6.2790 | L-threonine 3-dehydrogenase, putative | 36.9 | 6.2 | 7 | VLVTGALGQIGTDLSLALR | 328.64 | 100 | 96.71 | 100 | 2.41 ± 2.00 | 0.06 | 2.84 ± 1.78 | 0.02 | 1.4 |  |  |
| 198 | Tb927.6.5040 | ribosomal protein L15, putative | 24.4 | 12.5 | 1 | GITYGKPNTAGVLGR | 67.93 | 99.99 | 67.93 | 99.99 | 2.39 ± 0 | NA | 2.91 ± 0 | NA | 1.41 |  |  |
| 199 | Tb10.70.1370 | ALD, glycosomal | 41 | 9 | 3 | FAGIGLSNTAEHR | 131.74 | 100 | 54.41 | 99.96 | 2.57 ± 0.43 | 0.01 | 2.82 ± 0.89 | 0.04 | 1.43 |  |  |
| 200 | Tb10.70.5650 | TEF1 | 49.1 | 9.4 | 9 | VETGVMKPGDVVTFAPANVTTEVK | 642.91 | 100 | 191.28 | 100 | 2.33 ± 0.85 | 0.00 | 3.18 ± 1.24 | 0.00 | 1.46 | + |  |
| 201 | Tb09.160.2550 | 1L12.1, 60ribosomal protein S7, putative | 23.8 | 11.9 | 3 | ANPSQEEESVAR | 119.81 | 100 | 57.64 | 99.98 | 2.36 ± 0.57 | 0.03 | 3.24 ± 0.53 | 0.01 | 1.49 | + |  |
| 202 | Tb09.211.2150 | poly(A)-binding protein 1 | 62.1 | 10.1 | 1 | LTAIGLATDEKGESR | 38.79 | 98.46 | 38.79 | 98.46 | 2.41 ± 0 | NA | 3.25 ± 0 | NA | 1.5 |  |  |
| 203 | Tb927.3.5050 | 60S ribosomal protein L4 | 41.8 | 12.1 | 2 | FAVVSALAASSLPALVMSR | 139.71 | 100 | 112.85 | 100 | 2.80 ± 2.28 | 0.15 | 2.91 ± 1.05 | 0.04 | 1.51 |  |  |
| 204 | Tb11.02.4040 | protein transport protein Sec31, putative | 131.1 | 6.3 | 5 | LVQEVLLLEEVTGR | 137.51 | 100 | 91.33 | 100 | 2.85 ± 1.80 | 0.04 | 2.88 ± 2.31 | 0.07 | 1.52 |  |  |
| 205 | Tb11.02.2490 | hypothetical protein, conserved | 30.2 | 10 | 1 | GVPIVDATK | 39.48 | 98.68 | 39.48 | 98.68 | 3.18 ± 0 | NA | 2.81 ± 0 | NA | 1.58 | + |  |
| 206 | Tb10.61.2090 | 60S ribosomal protein L17, putative | 19.1 | 11.6 | 1 | HVQVDQAPR | 48.5 | 99.83 | 48.5 | 99.83 | 2.50 ± 0 | NA | 3.51 ± 0 | NA | 1.59 |  |  |
| 207 | Tb11.01.7960 | 60S ribosomal protein L2, putative | 28.3 | 11.4 | 1 | AIEHEPGR | 40.79 | 99.03 | 40.79 | 99.03 | 2.76 ± 0 | NA | 3.61 ± 0 | NA | 1.67 | + |  |
| 208 | Tb10.70.0280 | HSP60, mitochondrial precursor | 59.5 | 5.1 | 4 | SIEFKDPFENMGAQLVR | 256.35 | 100 | 123.11 | 100 | 2.47 ± 2.58 | 0.19 | 3.98 ± 6.34 | 0.21 | 1.69 | + |  |
| 209 | Tb09.v4.0013 | retrotransposon hot spot (RHS) protein, putative | 94.5 | 6 | 1 | EFVGPVAR | 38.21 | 98.23 | 38.21 | 98.23 | 2.67 ± 0 | NA | 3.9 ± 0 | NA | 1.71 |  |  |
| 210 | Tb927.4.1790 | ribosomal protein L3, putative | 54.4 | 11.7 | 9 | AVSMEPNQATTAYDLTAK | 536.02 | 100 | 153.01 | 100 | 2.95 ± 1.91 | 0.01 | 3.61 ± 2.04 | 0.00 | 1.71 |  |  |
| 211 | Tb11.42.0003 | t-complex protein 1, beta subunit, putative | 58 | 6.4 | 1 | SSVLGYAAEAAEMILR | 81.07 | 99.99 | 81.07 | 99.99 | 2.72 ± 0 | NA | 3.86 ± 0 | NA | 1.72 |  |  |
| 212 | Tb09.160.0350 | hypothetical protein, conserved | 59.8 | 6.3 | 1 | GTGNHALYATEQPQVLLSPTSNR | 106.63 | 100 | 106.63 | 100 | 2.88 ± 0 | NA | 3.71 ± 0 | NA | 1.72 |  |  |
| 213 | Tb10.70.1540 | 60S ribosomal protein L24, putative | 14.5 | 12 | 2 | AIVGAELSYIQEVR | 113.15 | 100 | 84.93 | 100 | 2.51 ± 0.14 | 0.02 | 4.38 ± 0.19 | 0.01 | 1.78 | + |  |
| 214 | Tb11.01.3960 | BILBO1 | 67.3 | 5.7 | 15 | AFSLAGEASAADDKVR | 923.86 | 100 | 110.57 | 100 | 3.53 ± 2.21 | 0.00 | 3.78 ± 1.71 | 0.00 | 1.87 | + |  |
| 215 | Tb927.6.4670 | TbMORN1 | 40.6 | 5.1 | 6 | GVVVYAAPDGCVSEKYDGEWNEGR | 397.24 | 100 | 144.87 | 100 | 3.71 ± 3.00 | 0.07 | 4.02 ± 3.05 | 0.03 | 1.95 | + |  |
| 216 | Tb927.2.4230 | NUP-1, putative | 406.8 | 4.7 | 5 | NADTDLGTQLASALVALER | 214.86 | 100 | 167.65 | 100 | 4.03 ± 2.73 | 0.03 | 4.17 ± 3.62 | 0.06 | 2.04 | + |  |
| 217 | Tb927.4.1860 | ribosomal protein S19, putative | 18.8 | 11.3 | 1 | GSRPEITTR | 34.52 | 95.87 | 34.52 | 95.87 | 4.44 ± 0 | NA | 5.28 ± 0 | NA | 2.28 |  |  |
| 218 | Tb10.70.6570 | hypothetical protein, conserved | 329.6 | 4.3 | 1 | FLEELEACR | 46.3 | 99.73 | 51.85 | 99.92 | 4.73 ± 0 | NA | 5.10 ± 0 | NA | 2.3 |  |  |
| 219 | Tb927.5.4250 | histone H4, putative | 11.1 | 11.6 | 5 | DATAYTEYSR | 355.61 | 100 | 73.91 | 99.99 | 10.77 ± 7.12 | 0.02 | 8.07 ± 4.14 | 0.01 | 3.24 | + |  |
| 220 | Tb927.7.2900 | histone H2A, putative | 14.2 | 11.8 | 4 | HDDDLGALLR | 232.57 | 100 | 68.55 | 99.99 | 9.83 ± 7.41 | 0.05 | 9.29 ± 2.34 | 0.00 | 3.26 | + |  |
| 221 | Tb927.1.2510 | histone H3, putative | 14.7 | 11.6 | 4 | FQSSAILAAQEATESYIVSLLADTNR | 311.03 | 100 | 173.39 | 100 | 8.66 ± 13.37 | 0.17 | 11.84 ± 12.09 | 0.09 | 3.36 | + |  |
| 222 | Tb927.3.3180 | hypothetical protein, conserved | 98.1 | 6.9 | 1 | NPFSFGASAGNASASGEKDNAPR | 30.16 | 88.73 | 35.84 | 96.95 | 12.94 ± 0 | NA | 10.12 ± 0 | NA | 3.53 |  |  |
| 223 | Tb10.406.0410 | histone H2B, putative | 12.5 | 12.2 | 6 | IVNSFVNDLFER | 347.51 | 100 | 79.6 | 99.99 | 12.98 ± 8.56 | 0.01 | 11.26 ± 3.44 | 0.00 | 3.6 | + |  |
