# Supplementary material for: A Comparative Proteomic Analysis Reveals a New Bi-Lobe Protein Required for Bi-Lobe Duplication and Cell Division in Trypanosoma brucei
Source: PLoS One. 2010 Mar 15;5(3):e9660. doi: 10.1371/journal.pone.0009660 (PMC2837748; doi:10.1371/journal.pone.0009660)
Supplement: Table S2 — Summary of candidate flagellar complex proteins identified in this study. (0.06 MB DOC) [file pone.0009660.s004.doc]

**Table S2**. Summary of candidate flagellar complex proteins identified in this study. Blast analyses were performed as described in Materials and Methods. “+” indicates a homologue in this species (with E-value <10^-5^) or this protein was previously found in the indicated flagellar proteome; “x“ denotes no homologue in this species or this protein was not found in the flagellar proteome.

| **Accession No.** | **Protein name** | **Length of gene (bp)** | **Matching peptides** | **Abundance ratio** | ***T. brucei*** | ***T. cruzi*** | ***L. major*** | ***P. falciparum*** | ***C. reinhardtii*** | ***S. pombe*** | ***A. thaliana*** | ***C. elegans*** | ***D. melanogaster*** | ***H. sapiens*** | ***T. thermophila*** | **Broadhead et al., 2006** | **Putative subcellular**  **localization**  **by YFP reporter** |
| --- | --- | --- | --- | --- | --- | --- | --- | --- | --- | --- | --- | --- | --- | --- | --- | --- | --- |
|  |
| Tb10.61.0560 | Hypothetical protein | 5247 | 9 | 1.32 | + | + | + | x | x | x | x | x | x | x | x | x | Basal body |
| Tb10.61.2190 | Hypothetical protein | 1401 | 2 | 1.12 | + | + | + | x | x | x | x | x | x | x | x | x | Basal body |
| Tb10.70.4780 | Hypothetical protein | 2343 | 2 | 1.07 | + | + | + | x | + | x | x | x | + | + | + | + | Basal body |
| Tb927.6.2790 | L-threonine-3-dehydrogenase | 999 | 7 | 1.4 | + | + | x | x | x | x | x | + | + | + | x | x | Basal body |
| Tb11.02.2490 | Hypothetical protein | 825 | 1 | 1.58 | + | + | + | x | + | x | x | x | x | x | + | + | Flagellum or FAZ |
| Tb09.160.0350 | Hypothetical protein | 1614 | 1 | 1.72 | + | + | + | x | x | x | x | x | x | x | x | x | Flagellar pocket |
| Tb11.01.0680 | HERTS, renamed TbLRRP1 | 2142 | 7 | 1.22 | + | + | + | x | + | x | + | x | + | + | + | + | Bilobed structure |
| Tb10.61.1630 | ZC3H40 | 1299 | 1 | 1.06 | + | + | + | x | x | x | x | x | x | x | x | x | Nucleus |
| Tb11.02.4040 | Sec31 | 3696 | 5 | 1.52 | + | + | + | + | + | + | + | x | + | + | + | x | ER exit site |
| Tb09.211.0350 | Adenylate kinase | 603 | 1 | 1.31 | + | + | + | x | + | + | + | + | + | + | + | + | Not clear |
| Tb11.42.0003 | TCP1, subunit beta | 1590 | 1 | 1.72 | + | + | + | + | + | + | + | + | + | + | + | x | Not clear |
| Tb927.3.3180 | Hypothetical protein | 2979 | 1 | 3.53 | + | + | x | x | x | x | x | x | x | x | x | x | Not clear |
| Tb927.7.2680 | Hypothetical protein | 2127 | 1 | 1.29 | + | + | + | x | x | x | x | x | x | x | x | x | Not clear |
| Tb927.3.4500 | Fumarate hydratase | 1698 | 1 | 0.63 | + | + | + | + | + | x | x | x | x | x | x | x | Not clear |
| Tb927.4.2080 | Hypothetical protein | 2721 | 8 | 0.53 | + | + | + | x | x | x | x | x | x | x | x | + | Flagellum or FAZ |
| Tb927.7.3440 | I/6 autoantigen | 741 | 2 | 0.81 | + | + | + | x | x | x | x | x | x | x | x | + | Not clear |
