# Supplementary material for: A Comparative Proteomic Analysis Reveals a New Bi-Lobe Protein Required for Bi-Lobe Duplication and Cell Division in Trypanosoma brucei
Source: PLoS One. 2010 Mar 15;5(3):e9660. doi: 10.1371/journal.pone.0009660 (PMC2837748; doi:10.1371/journal.pone.0009660)
Supplement: Text S1 — Summaries of iTRAQ-labeled proteins that have only a single peptide match. (4.65 MB DOC) [file pone.0009660.s005.doc]

Text S1

Summaries of iTRAQ-labeled proteins that have only a single peptide match. The matching peptides with ion score < 33 are shown in bold blue. Only those peptides with ion score >= 33 (the confidence interval is greater than 95%) are accepted as true peptide identifications.

1. Tb927.5.1690

Match to: **Tb927.5.1690** Score: **127**

**hypothetical protein, conserved; Trypanosoma bruceichr 5Manual**

Nominal mass (M_r_): **56950**; Calculated pI value: **7.56**

NCBI BLAST search of [Tb927.5.1690](http://www.ncbi.nlm.nih.gov/blast/Blast.cgi?ALIGNMENTS=50&ALIGNMENT_VIEW=Pairwise&AUTO_FORMAT=Semiauto&CDD_SEARCH=on&CLIENT=web&COMPOSITION_BASED_STATISTICS=on&DATABASE=nr&DESCRIPTIONS=100&ENTREZ_QUERY=(none)&EXPECT=10&FILTER=L&FORMAT_BLOCK_ON_RESPAGE=None&FORMAT_OBJECT=Alignment&FORMAT_TYPE=HTML&GAPCOSTS=11+1&I_THRESH=0.001&LAYOUT=TwoWindows&MATRIX_NAME=BLOSUM62&NCBI_GI=on&PAGE=Proteins&PROGRAM=blastp&QUERY=MESYKDVILSQPPAMYQRLPQPSNVAVENYKGILLCACPVNIPNGASMELRGNNATAPTGPVFVPAGGSNTPLGLGPSAEERATMERNHRQRVENLKSQRANVCAVVSQHKRWLRSFAKQMRQMKEEEVVREVERARRVDQMRRKWAQKASEATAQEQQERGAALDADRGGQGGQQQQQQQQRLSEGAMGNVPSAPEAKEKKKVGKKKKKPKWALTEDEALEDEIAEADDLLEFAKNLDYDKFISDYEVAGALAIMRDRVEELTRENNWTKESVERAAKENADDEDEHECDYEGEAEKKGAYDAEARGQRRKELQQQLSSTAVARKAAPAQVAAHDKEWSNSTSIAGALRRAIMRDALQLAERILASSESMQRIHTKFSLARILQYCAVCGEDPREAMQKPSIGGKKGLEKEPQIVKLHPDATGLETETSDGQGGQRRVLLDLQRSKERTQGLPYLYRCPAI&SERVICE=plain&SET_DEFAULTS.x=9&SET_DEFAULTS.y=5&SHOW_OVERVIEW=on&WORD_SIZE=3&END_OF_HTTPGET=Yes) against nr

Unformatted [sequence string](../../../../D:%5CProteomic%20data%5C2010-1-8%5Ccgi%5Cgetseq.pl%3FTBA927_IPI+Tb927%2E5%2E1690+seq) for pasting into other applications

Fixed modifications: MMTS (C),(N-TERM)_iTRAQ,Lysine(K)_iTRAQ

Variable modifications: Oxidation (M)

Cleavage by Trypsin: cuts C-term side of KR unless next residue is P

Sequence Coverage: **9%**

Matched peptides shown in **Bold Red**

**1** MESYKDVILS QPPAMYQRLP QPSNVAVENY KGILLCACPV NIPNGASMEL

**51** RGNNATAPTG PVFVPAGGSN TPLGLGPSAE ERATMERNHR QRVENLKSQR

**101** ANVCAVVSQH KRWLRSFAKQ MRQMKEEEVV REVERARRVD QMRRKWAQKA

**151** SEATAQEQQE RGAALDADRG GQGGQQQQQQ QQRLSEGAMG NVPSAPEAKE

**201** KKKVGKKKKK PKWALTEDEA LEDEIAEADD LLEFAKNLDY DKFISDYEVA

**251** GALAIMRDRV EELTRENNWT KESVERAAKE NADDEDEHEC DYEGEAEKKG

**301** AYDAEARGQR RK**ELQQQLSS TAVAR**KAAPA QVAAHDKEWS NSTSIAGALR

**351** RAIMRDALQL AER**ILASSES MQR**IHTKFSL ARILQYCAVC GEDPREAMQK

**401** PSIGGKKGLE KEPQIVK**LHP DATGLETETS DGQGGQR**RVL LDLQRSKERT

**451** QGLPYLYRCP AI

**Start - End Observed Mr(expt) Mr(calc) Delta Miss Sequence**

**313 - 325 1574.81 1573.81 1573.85 -0.05 0 K.ELQQQLSSTAVAR.K**  ([Ions score 25](../../../../D:%5CProteomic%20data%5C2010-1-8%5CZQ%5C833.htm))

**364 - 373 1265.67 1264.66 1264.66 0.01 0 R.ILASSESMQR.I**  ([Ions score 16](../../../../D:%5CProteomic%20data%5C2010-1-8%5CZQ%5C832.htm))

**418 - 437 2213.05 2212.04 2212.05 -0.01 0 K.LHPDATGLETETSDGQGGQR.R**  ([Ions score 127](../../../../D:%5CProteomic%20data%5C2010-1-8%5CZQ%5C834.htm))

MS/MS Fragmentation of **LHPDATGLETETSDGQGGQR**
Found in **Tb927.5.1690**, hypothetical protein, conserved; Trypanosoma bruceichr 5Manual


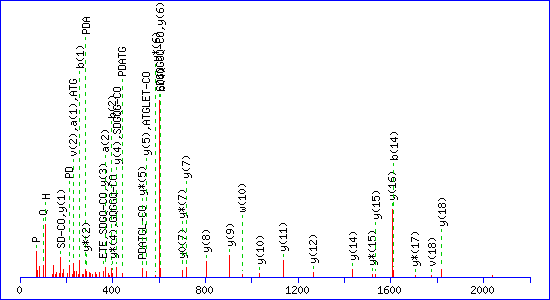


**MONOISOTOPIC mass of neutral peptide Mr(calc):** 2212.05

**Fixed modifications:** MMTS (C),(N-TERM)_iTRAQ,Lysine(K)_iTRAQ

**Ions Score:** 127 **Expect:** 1.9e-011

**Matches (Bold Red):** 48/346 fragment ions using 41 most intense peaks

| **#** | **Immon.** | **a** | **a*** | **a^0^** | **b** | **b*** | **b^0^** | **Seq.** | **v** | **w** | **w'** | **y** | **y*** | **y^0^** | **#** |
| --- | --- | --- | --- | --- | --- | --- | --- | --- | --- | --- | --- | --- | --- | --- | --- |
| **1** | 86.10 | **230.20** |  |  | **258.19** |  |  | **L** |  |  |  |  |  |  | **20** |
| **2** | **110.07** | **367.26** |  |  | **395.25** |  |  | **H** | 1873.82 |  |  | 1955.87 | 1938.84 | 1937.86 | **19** |
| **3** | **70.07** | 464.31 |  |  | 492.31 |  |  | **P** | **1776.76** | 1775.77 |  | **1818.81** | 1801.78 | 1800.80 | **18** |
| **4** | 88.04 | 579.34 |  | 561.33 | **607.33** |  | 589.32 | **D** | 1661.74 | 1660.74 |  | 1721.76 | **1704.73** | 1703.75 | **17** |
| **5** | 44.05 | 650.37 |  | 632.36 | 678.37 |  | 660.36 | **A** | 1590.70 |  |  | **1606.73** | 1589.70 | 1588.72 | **16** |
| **6** | 74.06 | 751.42 |  | 733.41 | 779.42 |  | 761.41 | **T** | 1489.65 | 1502.67 | 1504.65 | **1535.69** | **1518.67** | 1517.68 | **15** |
| **7** | 30.03 | 808.44 |  | 790.43 | 836.44 |  | 818.43 | **G** |  |  |  | **1434.65** | 1417.62 | 1416.63 | **14** |
| **8** | 86.10 | 921.53 |  | 903.52 | 949.52 |  | 931.51 | **L** | 1319.55 | 1318.55 |  | 1377.62 | 1360.60 | 1359.61 | **13** |
| **9** | 102.05 | 1050.57 |  | 1032.56 | 1078.57 |  | 1060.55 | **E** | 1190.50 | 1189.51 |  | **1264.54** | 1247.51 | 1246.53 | **12** |
| **10** | 74.06 | 1151.62 |  | 1133.61 | 1179.61 |  | 1161.60 | **T** | 1089.46 | 1102.48 | 1104.46 | **1135.50** | 1118.47 | 1117.49 | **11** |
| **11** | 102.05 | 1280.66 |  | 1262.65 | 1308.66 |  | 1290.64 | **E** | 960.41 | **959.42** |  | **1034.45** | 1017.42 | 1016.44 | **10** |
| **12** | 74.06 | 1381.71 |  | 1363.70 | 1409.70 |  | 1391.69 | **T** | 859.37 | 872.39 | 874.36 | **905.41** | 888.38 | 887.40 | **9** |
| **13** | 60.04 | 1468.74 |  | 1450.73 | 1496.74 |  | 1478.72 | **S** | 772.33 | 771.34 |  | **804.36** | 787.33 | 786.35 | **8** |
| **14** | 88.04 | 1583.77 |  | 1565.76 | **1611.76** |  | 1593.75 | **D** | 657.31 | 656.31 |  | **717.33** | **700.30** | **699.32** | **7** |
| **15** | 30.03 | 1640.79 |  | 1622.78 | 1668.78 |  | 1650.77 | **G** |  |  |  | **602.30** | **585.27** |  | **6** |
| **16** | **101.07** | 1768.85 | 1751.82 | 1750.84 | 1796.84 | 1779.82 | 1778.83 | **Q** | 472.23 | 471.23 |  | **545.28** | **528.25** |  | **5** |
| **17** | 30.03 | 1825.87 | 1808.84 | 1807.86 | 1853.86 | 1836.84 | 1835.85 | **G** |  |  |  | **417.22** | **400.19** |  | **4** |
| **18** | 30.03 | 1882.89 | 1865.86 | 1864.88 | 1910.89 | 1893.86 | 1892.87 | **G** |  |  |  | **360.20** | 343.17 |  | **3** |
| **19** | **101.07** | 2010.95 | 1993.92 | 1992.94 | 2038.94 | 2021.92 | 2020.93 | **Q** | **230.12** | 229.13 |  | 303.18 | **286.15** |  | **2** |
| **20** | 129.11 |  |  |  |  |  |  | **R** | 74.02 | 73.03 |  | **175.12** | 158.09 |  | **1** |

| **Seq** | **ya** | **yb** | **Seq** | **ya** | **yb** | **Seq** | **ya** | **yb** |
| --- | --- | --- | --- | --- | --- | --- | --- | --- |
| **HP** | 207.12 | 235.12 | **HPD** | 322.15 | 350.15 | **HPDA** | 393.19 | 421.18 |
| **HPDAT** | 494.24 | 522.23 | **HPDATG** | 551.26 | 579.25 | **HPDATGL** | 664.34 | 692.34 |
| **PD** | 185.09 | **213.09** | **PDA** | 256.13 | **284.12** | **PDAT** | 357.18 | 385.17 |
| **PDATG** | 414.20 | **442.19** | **PDATGL** | **527.28** | 555.28 | **PDATGLE** | 656.32 | 684.32 |
| **DA** | 159.08 | 187.07 | **DAT** | 260.12 | 288.12 | **DATG** | 317.15 | 345.14 |
| **DATGL** | 430.23 | 458.22 | **DATGLE** | 559.27 | 587.27 | **DATGLET** | 660.32 | 688.31 |
| **AT** | 145.10 | 173.09 | **ATG** | 202.12 | **230.11** | **ATGL** | 315.20 | 343.20 |
| **ATGLE** | 444.25 | 472.24 | **ATGLET** | **545.29** | 573.29 | **ATGLETE** | 674.34 | 702.33 |
| **TG** | 131.08 | 159.08 | **TGL** | 244.17 | 272.16 | **TGLE** | 373.21 | 401.20 |
| **TGLET** | 474.26 | 502.25 | **TGLETE** | 603.30 | 631.29 | **GL** | 143.12 | 171.11 |
| **GLE** | 272.16 | 300.16 | **GLET** | 373.21 | 401.20 | **GLETE** | 502.25 | 530.25 |
| **GLETET** | 603.30 | 631.29 | **GLETETS** | 690.33 | 718.33 | **LE** | 215.14 | 243.13 |
| **LET** | 316.19 | 344.18 | **LETE** | 445.23 | 473.22 | **LETET** | 546.28 | 574.27 |
| **LETETS** | 633.31 | 661.30 | **ET** | 203.10 | 231.10 | **ETE** | 332.15 | **360.14** |
| **ETET** | 433.19 | 461.19 | **ETETS** | 520.22 | 548.22 | **ETETSD** | 635.25 | 663.25 |
| **ETETSDG** | 692.27 | 720.27 | **TE** | 203.10 | 231.10 | **TET** | 304.15 | 332.15 |
| **TETS** | 391.18 | 419.18 | **TETSD** | 506.21 | 534.20 | **TETSDG** | 563.23 | 591.23 |
| **TETSDGQ** | 691.29 | 719.28 | **ET** | 203.10 | 231.10 | **ETS** | 290.13 | 318.13 |
| **ETSD** | 405.16 | 433.16 | **ETSDG** | 462.18 | 490.18 | **ETSDGQ** | 590.24 | 618.24 |
| **ETSDGQG** | 647.26 | 675.26 | **TS** | 161.09 | 189.09 | **TSD** | 276.12 | 304.11 |
| **TSDG** | 333.14 | 361.14 | **TSDGQ** | 461.20 | 489.19 | **TSDGQG** | 518.22 | 546.22 |
| **TSDGQGG** | 575.24 | 603.24 | **SD** | **175.07** | 203.07 | **SDG** | 232.09 | 260.09 |
| **SDGQ** | **360.15** | 388.15 | **SDGQG** | **417.17** | 445.17 | **SDGQGG** | 474.19 | 502.19 |
| **SDGQGGQ** | **602.25** | 630.25 | **DG** | 145.06 | 173.06 | **DGQ** | 273.12 | 301.11 |
| **DGQG** | 330.14 | 358.14 | **DGQGG** | 387.16 | 415.16 | **DGQGGQ** | 515.22 | 543.22 |
| **GQ** | 158.09 | 186.09 | **GQG** | 215.11 | 243.11 | **GQGG** | 272.14 | 300.13 |
| **GQGGQ** | **400.19** | 428.19 | **QG** | 158.09 | 186.09 | **QGG** | 215.11 | 243.11 |
| **QGGQ** | 343.17 | 371.17 | **GG** | 87.06 | 115.05 | **GGQ** | 215.11 | 243.11 |
| **GQ** | 158.09 | 186.09 |  |  |  |  |  |  |

2. Tb10.6k15.1760

Match to: **Tb10.6k15.1760** Score: **120**

**hypothetical protein, conserved; Trypanosoma bruceichr 10Manual**

Nominal mass (M_r_): **263271**; Calculated pI value: **4.78**

NCBI BLAST search of [Tb10.6k15.1760](http://www.ncbi.nlm.nih.gov/blast/Blast.cgi?ALIGNMENTS=50&ALIGNMENT_VIEW=Pairwise&AUTO_FORMAT=Semiauto&CDD_SEARCH=on&CLIENT=web&COMPOSITION_BASED_STATISTICS=on&DATABASE=nr&DESCRIPTIONS=100&ENTREZ_QUERY=(none)&EXPECT=10&FILTER=L&FORMAT_BLOCK_ON_RESPAGE=None&FORMAT_OBJECT=Alignment&FORMAT_TYPE=HTML&GAPCOSTS=11+1&I_THRESH=0.001&LAYOUT=TwoWindows&MATRIX_NAME=BLOSUM62&NCBI_GI=on&PAGE=Proteins&PROGRAM=blastp&QUERY=Tb10.6k15.1760&SERVICE=plain&SET_DEFAULTS.x=21&SET_DEFAULTS.y=7&SHOW_OVERVIEW=on&WORD_SIZE=3&END_OF_HTTPGET=Yes) against nr

Unformatted [sequence string](../../../../D:%5CProteomic%20data%5C2010-1-8%5Ccgi%5Cgetseq.pl%3FTBA927_IPI+Tb10%2E6k15%2E1760+seq) for pasting into other applications

Fixed modifications: MMTS (C),(N-TERM)_iTRAQ,Lysine(K)_iTRAQ

Variable modifications: Oxidation (M)

Cleavage by Trypsin: cuts C-term side of KR unless next residue is P

Sequence Coverage: **0%**

Matched peptides shown in **Bold Red**

**1** MKAAQPVVVD VEETFDSVAE KLWCALPSVA KFHATFEKTP ILGAESGNVG

**51** VVPQQSSVED TSEAAAAKLD PPVTIRDAVV PVDEMQHLAG CVYTACHTNP

**101** VDADVEAAVA AILDKGDEEN SVEYSWGDFR EFLERVESQW MEWADMDQKW

**151** LAVTERQHIQ VLKDLLESIT YDVEPFGLLP RDKVLTMRST IVHTKQLRTL

**201** FKTFVDFVSP PEYPAVYDKL WGLLIASQNG CEVAEVSPET PLELNSFTKF

**251** VLWFCLSIFV NATEAGLVVA AKTLWLRHLN ERGVMLKKQF VDVCRYMCKF

**301** YSVGGSDVEY FEKCCARSEE ALQGSDELPP FALFQQYMQP TEGRMLVDPN

**351** DLYIPEELER SKWMEELYRM NCSSNRIIVH GRRGVGKSHL AASLAKRLGC

**401** VHLDAGELAL EAEAAAPADP LGAQLR**ECTD ADAPISLATL AALVR**KKICS

**451** SETRYRGYVF SDIPFFSSEN DAEKISFFTD CGLLDELVPT TFVLVDCENE

**501** FHPERLEATL AARESDHQEE LSLLKEEKEE EASYEALAQK IEELKATLSQ

**551** ITTKQEVEGG AEAPEGEEAN APELDPEEVE KDLKQLLEDQ TVQEERKIEQ

**601** ADSRIARTKK YRELRLRRLV AQTLSEGYES GNEMSLTALP CFETALCRAR

**651** LMGRCLTVDC ASVAEEAVTY IVDTLSLQPC VRPWALVDKV ERDPEREGSV

**701** FDDPDIERLT EEFATNFGVM TSSRWKRFCP VTFAEHGVLV EGSIAFGCVF

**751** RQQLFYLASE EKLSMFRANP CLYLGTLPLS REPILLLSLV EPQNDAQLSP

**801** TDMQILVRKL HEQLDLTPMA FSEFTTLWDS HRLLKGKRAE VLSNRTKYEV

**851** VERKQRADRL KKRLAQEKRK KKSQKSPGKG KGKKAKSVVE EVTVEEYKGW

**901** EKKAEAPETI ATRIAKNLEE RLERQNTLVP VLVHALDDNP LSGFDQLFSE

**951** KVVPRTVVVL QYEKPTKAED SAVLEPSAAE GVEENSGEVP KVLLPQEVVL

**1001** DKLSDLPDGG VDIVAEVSPH DVAIHRIVVN DKDVHALVTE IMQAVCPGMD

**1051** PVGVGVVDDA VGEDDEEDAQ EFDDDENEED DVPTAVNPAI KPGKTFLNQF

**1101** GTTLEFCPVT LCERRLLVRG QSDHCLQYRG YVYTFASLEA KTKFEFNPLR

**1151** YMRSSYSLPP CRMWIVGQSK SGKKTLAQSL HEAYDVPYFQ YNRKLFDQCV

**1201** EVAMTPTGGV ISNIFIPPQT FDNPYLALAA GILKEVQEFD PEQERRMKLR

**1251** EEAERELERR EEAANNGDEE EDELDEEAEA RLQEHLAFEP ETEHDRQLRL

**1301** SEAYLKVAGC VTHIEPFASK GYIMVCPPFS DGDIEVLSSV DAIPEVTVNM

**1351** EVSDEIYIKR NKEAMSSVSY SEMSSGGIPE VREEPVVNEE AKLLRRLEYE

**1401** DRRKEREVAR WRRRHIGADD PESDIDEELG DEGAAGGGND ESQEDEANER

**1451** PFDQDLIVEK EAVGEFAEAV EERSIPMIVL NGDLSRNAVF RRAVRRLSRF

**1501** LENRRSLLHA PQIVRYEDAT RMLESGEATL SCFGSTDPVT LYDLRHGSRR

**1551** VCKWRPDGAY LEEEPLIEPD SSSSDAQGTA VPEGSEGKDE SEDTLDSGDR

**1601** KPEPPQLDTS STEGDTESGD DEEMSELDSE LLDEITEKFA NRRRREWLRT

**1651** CQRVALLHGR LFFFESDETL LRYMQNPLLF IQQPPPQPPL RSMPVITFYD

**1701** DDGAYPPENS GPSRKRCTAE HVAFNLNWIY LSLPKLLSWS AVNASLLSLS

**1751** RKAIDAVLSG SVDDALVARL LGHRLNAADA KQNGVVLHNL PRTPDQYRLL

**1801** LAWGLKVDKI FQFDDNYRDV ATLMKSTATI EKQLTTARVS VAGLSEICDC

**1851** IDGFVVNEGR AILSHLTGFP IDIDNSYHTV ADIETHLSPY RWFCPYSWCL

**1901** GENLVDQEKA DCRFAALYDG QYYFFSSEEY LERFLLCPSQ VTLPPGFKAL

**1951** PTPLPVRVQP STEYAFELEG CCPVLLYDTR ENRGLRGVLE PVARKGNPSC

**2001** IVEYGGCYYA LLDEEAVRRF LMRPWQYVDG AKLPPSRKVP LPEGKTMSTI

**2051** DEEEFIRRIL YDPVAHALIA VAEVRPKYYG LSLEESALKY IALHMKCFNP

**2101** KNSEIQAKQY KKKFEIFSKQ STLYKTITMH SNSITQNEKF AELCDEWENS

**2151** KYGREKELSI HCGCETEPV

MS/MS Fragmentation of **ECTDADAPISLATLAALVR**
Found in **Tb10.6k15.1760**, hypothetical protein, conserved; Trypanosoma bruceichr 10Manual


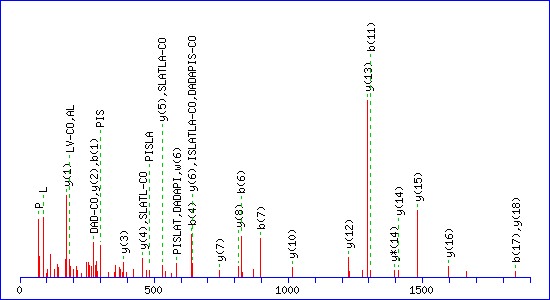


**MONOISOTOPIC mass of neutral peptide Mr(calc):** 2119.08

**Fixed modifications:** MMTS (C),(N-TERM)_iTRAQ,Lysine(K)_iTRAQ

**Ions Score:** 120 **Expect:** 9e-011

**Matches (Bold Red):** 43/334 fragment ions using 28 most intense peaks

| **#** | **Immon.** | **a** | **a^0^** | **b** | **b^0^** | **Seq.** | **v** | **w** | **w'** | **y** | **y*** | **y^0^** | **#** |
| --- | --- | --- | --- | --- | --- | --- | --- | --- | --- | --- | --- | --- | --- |
| **1** | 102.05 | 246.16 | 228.15 | **274.15** | 256.14 | **E** |  |  |  |  |  |  | **19** |
| **2** | 122.01 | 395.15 | 377.14 | 423.15 | 405.14 | **C** | 1752.95 | 1751.95 |  | **1846.94** | 1829.91 | 1828.93 | **18** |
| **3** | 74.06 | 496.20 | 478.19 | 524.20 | 506.19 | **T** | 1651.90 | 1664.92 | 1666.90 | 1697.94 | 1680.92 | 1679.93 | **17** |
| **4** | 88.04 | 611.23 | 593.22 | **639.22** | 621.21 | **D** | 1536.87 | 1535.88 |  | **1596.90** | 1579.87 | 1578.88 | **16** |
| **5** | 44.05 | 682.27 | 664.26 | 710.26 | 692.25 | **A** | 1465.84 |  |  | **1481.87** | 1464.84 | 1463.86 | **15** |
| **6** | 88.04 | 797.29 | 779.28 | **825.29** | 807.28 | **D** | 1350.81 | 1349.82 |  | **1410.83** | **1393.80** | 1392.82 | **14** |
| **7** | 44.05 | 868.33 | 850.32 | **896.32** | 878.31 | **A** | 1279.77 |  |  | **1295.80** | 1278.78 | 1277.79 | **13** |
| **8** | **70.07** | 965.38 | 947.37 | 993.38 | 975.37 | **P** | 1182.72 | 1181.73 |  | **1224.77** | 1207.74 | 1206.76 | **12** |
| **9** | **86.10** | 1078.47 | 1060.46 | 1106.46 | 1088.45 | **I** | 1069.64 | 1082.66 | 1096.67 | 1127.71 | 1110.69 | 1109.70 | **11** |
| **10** | 60.04 | 1165.50 | 1147.49 | 1193.49 | 1175.48 | **S** | 982.60 | 981.61 |  | **1014.63** | 997.60 | 996.62 | **10** |
| **11** | **86.10** | 1278.58 | 1260.57 | **1306.58** | 1288.57 | **L** | 869.52 | 868.53 |  | 927.60 | 910.57 | 909.59 | **9** |
| **12** | 44.05 | 1349.62 | 1331.61 | 1377.62 | 1359.60 | **A** | 798.48 |  |  | **814.51** | 797.49 | 796.50 | **8** |
| **13** | 74.06 | 1450.67 | 1432.66 | 1478.66 | 1460.65 | **T** | 697.44 | 710.46 | 712.44 | **743.48** | 726.45 | 725.47 | **7** |
| **14** | **86.10** | 1563.75 | 1545.74 | 1591.75 | 1573.74 | **L** | 584.35 | **583.36** |  | **642.43** | 625.40 |  | **6** |
| **15** | 44.05 | 1634.79 | 1616.78 | 1662.78 | 1644.77 | **A** | 513.31 |  |  | **529.35** | 512.32 |  | **5** |
| **16** | 44.05 | 1705.83 | 1687.82 | 1733.82 | 1715.81 | **A** | 442.28 |  |  | **458.31** | 441.28 |  | **4** |
| **17** | **86.10** | 1818.91 | 1800.90 | **1846.91** | 1828.89 | **L** | 329.19 | 328.20 |  | **387.27** | 370.24 |  | **3** |
| **18** | 72.08 | 1917.98 | 1899.97 | 1945.97 | 1927.96 | **V** | 230.12 | 243.15 |  | **274.19** | 257.16 |  | **2** |
| **19** | 129.11 |  |  |  |  | **R** | 74.02 | 73.03 |  | **175.12** | 158.09 |  | **1** |

| **Seq** | **ya** | **yb** | **Seq** | **ya** | **yb** | **Seq** | **ya** | **yb** |
| --- | --- | --- | --- | --- | --- | --- | --- | --- |
| **CT** | 223.06 | 251.05 | **CTD** | 338.08 | 366.08 | **CTDA** | 409.12 | 437.12 |
| **CTDAD** | 524.15 | 552.14 | **CTDADA** | 595.19 | 623.18 | **CTDADAP** | 692.24 | 720.23 |
| **TD** | 189.09 | 217.08 | **TDA** | 260.12 | 288.12 | **TDAD** | 375.15 | 403.15 |
| **TDADA** | 446.19 | 474.18 | **TDADAP** | 543.24 | 571.24 | **TDADAPI** | 656.32 | 684.32 |
| **DA** | 159.08 | 187.07 | **DAD** | **274.10** | 302.10 | **DADA** | 345.14 | 373.14 |
| **DADAP** | 442.19 | 470.19 | **DADAPI** | 555.28 | **583.27** | **DADAPIS** | **642.31** | 670.30 |
| **AD** | 159.08 | 187.07 | **ADA** | 230.11 | 258.11 | **ADAP** | 327.17 | 355.16 |
| **ADAPI** | 440.25 | 468.25 | **ADAPIS** | 527.28 | 555.28 | **ADAPISL** | 640.37 | 668.36 |
| **DA** | 159.08 | 187.07 | **DAP** | 256.13 | 284.12 | **DAPI** | 369.21 | 397.21 |
| **DAPIS** | 456.25 | 484.24 | **DAPISL** | 569.33 | 597.32 | **DAPISLA** | 640.37 | 668.36 |
| **AP** | 141.10 | 169.10 | **API** | 254.19 | 282.18 | **APIS** | 341.22 | 369.21 |
| **APISL** | 454.30 | **482.30** | **APISLA** | 525.34 | 553.33 | **APISLAT** | 626.39 | 654.38 |
| **PI** | 183.15 | 211.14 | **PIS** | 270.18 | **298.18** | **PISL** | 383.27 | 411.26 |
| **PISLA** | 454.30 | **482.30** | **PISLAT** | 555.35 | **583.34** | **PISLATL** | 668.43 | 696.43 |
| **IS** | 173.13 | 201.12 | **ISL** | 286.21 | 314.21 | **ISLA** | 357.25 | 385.24 |
| **ISLAT** | **458.30** | 486.29 | **ISLATL** | 571.38 | 599.38 | **ISLATLA** | **642.42** | 670.41 |
| **SL** | 173.13 | 201.12 | **SLA** | 244.17 | 272.16 | **SLAT** | 345.21 | 373.21 |
| **SLATL** | **458.30** | 486.29 | **SLATLA** | **529.33** | 557.33 | **SLATLAA** | 600.37 | 628.37 |
| **LA** | 157.13 | **185.13** | **LAT** | 258.18 | 286.18 | **LATL** | 371.27 | 399.26 |
| **LATLA** | 442.30 | 470.30 | **LATLAA** | 513.34 | 541.33 | **LATLAAL** | 626.42 | 654.42 |
| **AT** | 145.10 | 173.09 | **ATL** | 258.18 | 286.18 | **ATLA** | 329.22 | 357.21 |
| **ATLAA** | 400.26 | 428.25 | **ATLAAL** | 513.34 | 541.33 | **ATLAALV** | 612.41 | 640.40 |
| **TL** | 187.14 | 215.14 | **TLA** | 258.18 | 286.18 | **TLAA** | 329.22 | 357.21 |
| **TLAAL** | 442.30 | 470.30 | **TLAALV** | 541.37 | 569.37 | **LA** | 157.13 | **185.13** |
| **LAA** | 228.17 | 256.17 | **LAAL** | 341.25 | 369.25 | **LAALV** | 440.32 | 468.32 |
| **AA** | 115.09 | 143.08 | **AAL** | 228.17 | 256.17 | **AALV** | 327.24 | 355.23 |
| **AL** | 157.13 | **185.13** | **ALV** | 256.20 | 284.20 | **LV** | **185.16** | 213.16 |

3. Tb10.389.1320

Match to: **Tb10.389.1320** Score: **113**

**hypothetical protein, conserved; Trypanosoma bruceichr 10Manual**

Nominal mass (M_r_): **26602**; Calculated pI value: **5.28**

NCBI BLAST search of [Tb10.389.1320](http://www.ncbi.nlm.nih.gov/blast/Blast.cgi?ALIGNMENTS=50&ALIGNMENT_VIEW=Pairwise&AUTO_FORMAT=Semiauto&CDD_SEARCH=on&CLIENT=web&COMPOSITION_BASED_STATISTICS=on&DATABASE=nr&DESCRIPTIONS=100&ENTREZ_QUERY=(none)&EXPECT=10&FILTER=L&FORMAT_BLOCK_ON_RESPAGE=None&FORMAT_OBJECT=Alignment&FORMAT_TYPE=HTML&GAPCOSTS=11+1&I_THRESH=0.001&LAYOUT=TwoWindows&MATRIX_NAME=BLOSUM62&NCBI_GI=on&PAGE=Proteins&PROGRAM=blastp&QUERY=MEVKIGKDVALLTAHKTYRDDGEDMVKAVATYVPFMNYVAACESSGLVPSAFLVRNVRHIARRVVGVIMDVECNTPTGKIVQPVEMSDFSPAILLPVVIVETVRYALLLQRRCVAVGCGLTTEAFCGAKDSGDNITWQNHELLTSAGFDLRDVRKLGFGEYSVGNEGLPPYTLHTIKKGMSSEEFEQLQKISAGTADASLFAVRLEDVMSSVNDAKAGLAASVLLLES&SERVICE=plain&SET_DEFAULTS.x=9&SET_DEFAULTS.y=5&SHOW_OVERVIEW=on&WORD_SIZE=3&END_OF_HTTPGET=Yes) against nr

Unformatted [sequence string](../../../../D:%5CProteomic%20data%5C2010-1-8%5Ccgi%5Cgetseq.pl%3FTBA927_IPI+Tb10%2E389%2E1320+seq) for pasting into other applications

Fixed modifications: MMTS (C),(N-TERM)_iTRAQ,Lysine(K)_iTRAQ

Variable modifications: Oxidation (M)

Cleavage by Trypsin: cuts C-term side of KR unless next residue is P

Sequence Coverage: **9%**

Matched peptides shown in **Bold Red**

**1** MEVKIGKDVA LLTAHKTYRD DGEDMVKAVA TYVPFMNYVA ACESSGLVPS

**51** AFLVRNVRHI ARRVVGVIMD VECNTPTGKI VQPVEMSDFS PAILLPVVIV

**101** ETVRYALLLQ RRCVAVGCGL TTEAFCGAK**D SGDNITWQNH ELLTSAGFDL**

**151 R**DVRKLGFGE YSVGNEGLPP YTLHTIKKGM SSEEFEQLQK ISAGTADASL

**201** FAVRLEDVMS SVNDAKAGLA ASVLLLES

MS/MS Fragmentation of **DSGDNITWQNHELLTSAGFDLR**
Found in **Tb10.389.1320**, hypothetical protein, conserved; Trypanosoma bruceichr 10Manual


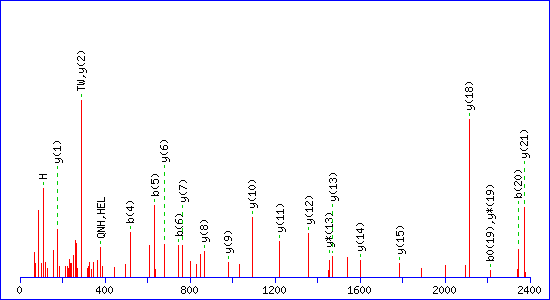


**MONOISOTOPIC mass of neutral peptide Mr(calc):** 2632.26

**Fixed modifications:** MMTS (C),(N-TERM)_iTRAQ,Lysine(K)_iTRAQ

**Ions Score:** 113 **Expect:** 3.5e-010

**Matches (Bold Red):** 25/403 fragment ions using 27 most intense peaks

| **#** | **Immon.** | **a** | **a*** | **a^0^** | **b** | **b*** | **b^0^** | **Seq.** | **v** | **w** | **w'** | **y** | **y*** | **y^0^** | **#** |
| --- | --- | --- | --- | --- | --- | --- | --- | --- | --- | --- | --- | --- | --- | --- | --- |
| **1** | 88.04 | 232.14 |  | 214.13 | 260.14 |  | 242.13 | **D** |  |  |  |  |  |  | **22** |
| **2** | 60.04 | 319.17 |  | 301.16 | 347.17 |  | 329.16 | **S** | 2342.12 | 2341.12 |  | **2374.14** | 2357.12 | 2356.13 | **21** |
| **3** | 30.03 | 376.20 |  | 358.18 | 404.19 |  | 386.18 | **G** |  |  |  | 2287.11 | 2270.08 | 2269.10 | **20** |
| **4** | 88.04 | 491.22 |  | 473.21 | **519.22** |  | 501.21 | **D** | 2170.07 | 2169.07 |  | 2230.09 | **2213.06** | 2212.08 | **19** |
| **5** | 87.06 | 605.27 | 588.24 | 587.25 | **633.26** | 616.23 | 615.25 | **N** | 2056.02 | 2055.03 |  | **2115.06** | 2098.04 | 2097.05 | **18** |
| **6** | 86.10 | 718.35 | 701.32 | 700.34 | **746.34** | 729.32 | 728.33 | **I** | 1942.94 | 1955.96 | 1969.98 | 2001.02 | 1983.99 | 1983.01 | **17** |
| **7** | 74.06 | 819.40 | 802.37 | 801.39 | 847.39 | 830.37 | 829.38 | **T** | 1841.89 | 1854.91 | 1856.89 | 1887.93 | 1870.91 | 1869.92 | **16** |
| **8** | 159.09 | 1005.48 | 988.45 | 987.47 | 1033.47 | 1016.44 | 1015.46 | **W** | 1655.81 |  |  | **1786.89** | 1769.86 | 1768.88 | **15** |
| **9** | 101.07 | 1133.53 | 1116.51 | 1115.52 | 1161.53 | 1144.50 | 1143.52 | **Q** | 1527.76 | 1526.76 |  | **1600.81** | 1583.78 | 1582.80 | **14** |
| **10** | 87.06 | 1247.58 | 1230.55 | 1229.57 | 1275.57 | 1258.55 | 1257.56 | **N** | 1413.71 | 1412.72 |  | **1472.75** | **1455.72** | 1454.74 | **13** |
| **11** | **110.07** | 1384.64 | 1367.61 | 1366.63 | 1412.63 | 1395.60 | 1394.62 | **H** | 1276.65 |  |  | **1358.71** | 1341.68 | 1340.70 | **12** |
| **12** | 102.05 | 1513.68 | 1496.65 | 1495.67 | 1541.67 | 1524.65 | 1523.66 | **E** | 1147.61 | 1146.62 |  | **1221.65** | 1204.62 | 1203.64 | **11** |
| **13** | 86.10 | 1626.76 | 1609.74 | 1608.75 | 1654.76 | 1637.73 | 1636.75 | **L** | 1034.53 | 1033.53 |  | **1092.60** | 1075.58 | 1074.59 | **10** |
| **14** | 86.10 | 1739.85 | 1722.82 | 1721.84 | 1767.84 | 1750.82 | 1749.83 | **L** | 921.44 | 920.45 |  | **979.52** | 962.49 | 961.51 | **9** |
| **15** | 74.06 | 1840.89 | 1823.87 | 1822.88 | 1868.89 | 1851.86 | 1850.88 | **T** | 820.39 | 833.42 | 835.39 | **866.44** | 849.41 | 848.43 | **8** |
| **16** | 60.04 | 1927.93 | 1910.90 | 1909.92 | 1955.92 | 1938.90 | 1937.91 | **S** | 733.36 | 732.37 |  | **765.39** | 748.36 | 747.38 | **7** |
| **17** | 44.05 | 1998.96 | 1981.94 | 1980.95 | 2026.96 | 2009.93 | 2008.95 | **A** | 662.33 |  |  | **678.36** | 661.33 | 660.35 | **6** |
| **18** | 30.03 | 2055.99 | 2038.96 | 2037.97 | 2083.98 | 2066.95 | 2065.97 | **G** |  |  |  | 607.32 | 590.29 | 589.31 | **5** |
| **19** | 120.08 | 2203.05 | 2186.03 | 2185.04 | 2231.05 | 2214.02 | **2213.04** | **F** | 458.24 |  |  | 550.30 | 533.27 | 532.29 | **4** |
| **20** | 88.04 | 2318.08 | 2301.05 | 2300.07 | **2346.08** | 2329.05 | 2328.07 | **D** | 343.21 | 342.21 |  | 403.23 | 386.20 | 385.22 | **3** |
| **21** | 86.10 | 2431.16 | 2414.14 | 2413.15 | 2459.16 | 2442.13 | 2441.15 | **L** | 230.12 | 229.13 |  | **288.20** | 271.18 |  | **2** |
| **22** | 129.11 |  |  |  |  |  |  | **R** | 74.02 | 73.03 |  | **175.12** | 158.09 |  | **1** |

| **Seq** | **ya** | **yb** | **Seq** | **ya** | **yb** | **Seq** | **ya** | **yb** |
| --- | --- | --- | --- | --- | --- | --- | --- | --- |
| **SG** | 117.07 | 145.06 | **SGD** | 232.09 | 260.09 | **SGDN** | 346.14 | 374.13 |
| **SGDNI** | 459.22 | 487.21 | **SGDNIT** | 560.27 | 588.26 | **GD** | 145.06 | 173.06 |
| **GDN** | 259.10 | 287.10 | **GDNI** | 372.19 | 400.18 | **GDNIT** | 473.24 | 501.23 |
| **GDNITW** | 659.31 | 687.31 | **DN** | 202.08 | 230.08 | **DNI** | 315.17 | 343.16 |
| **DNIT** | 416.21 | 444.21 | **DNITW** | 602.29 | 630.29 | **NI** | 200.14 | 228.13 |
| **NIT** | 301.19 | 329.18 | **NITW** | 487.27 | 515.26 | **NITWQ** | 615.32 | 643.32 |
| **IT** | 187.14 | 215.14 | **ITW** | 373.22 | 401.22 | **ITWQ** | 501.28 | 529.28 |
| **ITWQN** | 615.32 | 643.32 | **TW** | 260.14 | **288.13** | **TWQ** | 388.20 | 416.19 |
| **TWQN** | 502.24 | 530.24 | **TWQNH** | 639.30 | 667.29 | **WQ** | 287.15 | 315.15 |
| **WQN** | 401.19 | 429.19 | **WQNH** | 538.25 | 566.25 | **WQNHE** | 667.29 | 695.29 |
| **QN** | 215.11 | 243.11 | **QNH** | 352.17 | **380.17** | **QNHE** | 481.22 | 509.21 |
| **QNHEL** | 594.30 | 622.29 | **NH** | 224.11 | 252.11 | **NHE** | 353.16 | 381.15 |
| **NHEL** | 466.24 | 494.24 | **NHELL** | 579.32 | 607.32 | **NHELLT** | 680.37 | 708.37 |
| **HE** | 239.11 | 267.11 | **HEL** | 352.20 | **380.19** | **HELL** | 465.28 | 493.28 |
| **HELLT** | 566.33 | 594.32 | **HELLTS** | 653.36 | 681.36 | **EL** | 215.14 | 243.13 |
| **ELL** | 328.22 | 356.22 | **ELLT** | 429.27 | 457.27 | **ELLTS** | 516.30 | 544.30 |
| **ELLTSA** | 587.34 | 615.33 | **ELLTSAG** | 644.36 | 672.36 | **LL** | 199.18 | 227.18 |
| **LLT** | 300.23 | 328.22 | **LLTS** | 387.26 | 415.26 | **LLTSA** | 458.30 | 486.29 |
| **LLTSAG** | 515.32 | 543.31 | **LLTSAGF** | 662.39 | 690.38 | **LT** | 187.14 | 215.14 |
| **LTS** | 274.18 | 302.17 | **LTSA** | 345.21 | 373.21 | **LTSAG** | 402.23 | 430.23 |
| **LTSAGF** | 549.30 | 577.30 | **LTSAGFD** | 664.33 | 692.32 | **TS** | 161.09 | 189.09 |
| **TSA** | 232.13 | 260.12 | **TSAG** | 289.15 | 317.15 | **TSAGF** | 436.22 | 464.21 |
| **TSAGFD** | 551.25 | 579.24 | **TSAGFDL** | 664.33 | 692.32 | **SA** | 131.08 | 159.08 |
| **SAG** | 188.10 | 216.10 | **SAGF** | 335.17 | 363.17 | **SAGFD** | 450.20 | 478.19 |
| **SAGFDL** | 563.28 | 591.28 | **AG** | 101.07 | 129.07 | **AGF** | 248.14 | 276.13 |
| **AGFD** | 363.17 | 391.16 | **AGFDL** | 476.25 | 504.25 | **GF** | 177.10 | 205.10 |
| **GFD** | 292.13 | 320.12 | **GFDL** | 405.21 | 433.21 | **FD** | 235.11 | 263.10 |
| **FDL** | 348.19 | 376.19 | **DL** | 201.12 | 229.12 |  |  |  |

4. Tb09.160.0350

Match to: **Tb09.160.0350** Score: **107**

**hypothetical protein, conserved; Trypanosoma bruceichr 9Manual**

Nominal mass (M_r_): **64183**; Calculated pI value: **6.02**

NCBI BLAST search of [Tb09.160.0350](http://www.ncbi.nlm.nih.gov/blast/Blast.cgi?ALIGNMENTS=50&ALIGNMENT_VIEW=Pairwise&AUTO_FORMAT=Semiauto&CDD_SEARCH=on&CLIENT=web&COMPOSITION_BASED_STATISTICS=on&DATABASE=nr&DESCRIPTIONS=100&ENTREZ_QUERY=(none)&EXPECT=10&FILTER=L&FORMAT_BLOCK_ON_RESPAGE=None&FORMAT_OBJECT=Alignment&FORMAT_TYPE=HTML&GAPCOSTS=11+1&I_THRESH=0.001&LAYOUT=TwoWindows&MATRIX_NAME=BLOSUM62&NCBI_GI=on&PAGE=Proteins&PROGRAM=blastp&QUERY=MVFRDASQPRRMLVIEDIVTENRKRMELMGINANNESLCRLLLQMMSGVGELSNLAEQGEGALSLSRFEQEEVRTLIGRLAVSLFTVADICEVNLGQTAMDFISDQIQRKNSAPSLSFKEASKRSAPVPIVGDKGTGNHALYATEQPQVLLSPTSNRDFFSQLDALLTHNREDFEKADLKWVAPLPDGTVHELIENGSTINVSYDDIPMYLEKIQRYRNARVTEISTMARHCDVNTRHGSRPKGAAVAVCASDAKKERFDQLFSPPSTADSDLLNSNIGAVKKYPSPSEIMLQQPNPVDATLIGVVVSEAEFQAKVEAIKNGHIFGPAIAQQNLTFSVPRGGKLVELVPNGIHMKVTSANVSEFLRLLNDKSAALGGRVRRLESIKKLEVAGVGSQDLSREKGKFSPTHFSKDIFAPYDERTSFFVDTDASEEILPLYLRDQEELQRRADTYYVHVIRQGDIKTWNAIFEQVQADPSMLKKYGVTFCVPSSFSSHGSENEQRPRIHELITRGSTTPVEESQLWLFTKMVKQVMHPSA&SERVICE=plain&SET_DEFAULTS.x=9&SET_DEFAULTS.y=5&SHOW_OVERVIEW=on&WORD_SIZE=3&END_OF_HTTPGET=Yes) against nr

Unformatted [sequence string](../../../../D:%5CProteomic%20data%5C2010-1-8%5Ccgi%5Cgetseq.pl%3FTBA927_IPI+Tb09%2E160%2E0350+seq) for pasting into other applications

Fixed modifications: MMTS (C),(N-TERM)_iTRAQ,Lysine(K)_iTRAQ

Variable modifications: Oxidation (M)

Cleavage by Trypsin: cuts C-term side of KR unless next residue is P

Sequence Coverage: **4%**

Matched peptides shown in **Bold Red**

**1** MVFRDASQPR RMLVIEDIVT ENRKRMELMG INANNESLCR LLLQMMSGVG

**51** ELSNLAEQGE GALSLSRFEQ EEVRTLIGRL AVSLFTVADI CEVNLGQTAM

**101** DFISDQIQRK NSAPSLSFKE ASKRSAPVPI VGDK**GTGNHA LYATEQPQVL**

**151 LSPTSNR**DFF SQLDALLTHN REDFEKADLK WVAPLPDGTV HELIENGSTI

**201** NVSYDDIPMY LEKIQRYRNA RVTEISTMAR HCDVNTRHGS RPKGAAVAVC

**251** ASDAKKERFD QLFSPPSTAD SDLLNSNIGA VKKYPSPSEI MLQQPNPVDA

**301** TLIGVVVSEA EFQAKVEAIK NGHIFGPAIA QQNLTFSVPR GGKLVELVPN

**351** GIHMKVTSAN VSEFLRLLND KSAALGGRVR RLESIKKLEV AGVGSQDLSR

**401** EKGKFSPTHF SKDIFAPYDE RTSFFVDTDA SEEILPLYLR DQEELQRRAD

**451** TYYVHVIRQG DIKTWNAIFE QVQADPSMLK KYGVTFCVPS SFSSHGSENE

**501** QRPRIHELIT RGSTTPVEES QLWLFTKMVK QVMHPSA

MS/MS Fragmentation of **GTGNHALYATEQPQVLLSPTSNR**
Found in **Tb09.160.0350**, hypothetical protein, conserved; Trypanosoma bruceichr 9Manual


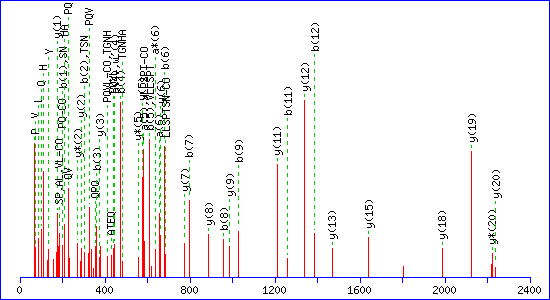


**MONOISOTOPIC mass of neutral peptide Mr(calc):** 2597.33

**Fixed modifications:** MMTS (C),(N-TERM)_iTRAQ,Lysine(K)_iTRAQ

**Ions Score:** 107 **Expect:** 1.5e-009

**Matches (Bold Red):** 65/435 fragment ions using 59 most intense peaks

| **#** | **Immon.** | **a** | **a*** | **a^0^** | **b** | **b*** | **b^0^** | **Seq.** | **v** | **w** | **w'** | **y** | **y*** | **y^0^** | **#** |
| --- | --- | --- | --- | --- | --- | --- | --- | --- | --- | --- | --- | --- | --- | --- | --- |
| **1** | 30.03 | 174.14 |  |  | **202.13** |  |  | **G** |  |  |  |  |  |  | **23** |
| **2** | 74.06 | 275.18 |  | 257.17 | **303.18** |  | 285.17 | **T** | 2351.17 | 2364.19 | 2366.17 | 2397.22 | 2380.19 | 2379.21 | **22** |
| **3** | 30.03 | 332.21 |  | 314.19 | **360.20** |  | 342.19 | **G** |  |  |  | 2296.17 | 2279.14 | 2278.16 | **21** |
| **4** | 87.06 | **446.25** | 429.22 | 428.24 | **474.24** | 457.22 | 456.23 | **N** | 2180.11 | 2179.11 |  | **2239.15** | **2222.12** | 2221.14 | **20** |
| **5** | **110.07** | **583.31** | 566.28 | 565.30 | **611.30** | 594.28 | 593.29 | **H** | 2043.05 |  |  | **2125.10** | 2108.08 | 2107.09 | **19** |
| **6** | 44.05 | **654.34** | **637.32** | 636.33 | **682.34** | 665.31 | 664.33 | **A** | 1972.01 |  |  | **1988.04** | 1971.02 | 1970.03 | **18** |
| **7** | **86.10** | 767.43 | 750.40 | 749.42 | **795.42** | 778.40 | 777.41 | **L** | 1858.93 | 1857.93 |  | 1917.01 | 1899.98 | 1899.00 | **17** |
| **8** | **136.08** | 930.49 | 913.47 | 912.48 | **958.49** | 941.46 | 940.48 | **Y** | 1695.87 |  |  | 1803.92 | 1786.90 | 1785.91 | **16** |
| **9** | 44.05 | 1001.53 | 984.50 | 983.52 | **1029.52** | 1012.50 | 1011.51 | **A** | 1624.83 |  |  | **1640.86** | 1623.83 | 1622.85 | **15** |
| **10** | 74.06 | 1102.58 | 1085.55 | 1084.57 | 1130.57 | 1113.54 | 1112.56 | **T** | 1523.78 | 1536.80 | 1538.78 | 1569.82 | 1552.80 | 1551.81 | **14** |
| **11** | 102.05 | 1231.62 | 1214.59 | 1213.61 | **1259.61** | 1242.59 | 1241.60 | **E** | 1394.74 | 1393.74 |  | **1468.78** | 1451.75 | 1450.76 | **13** |
| **12** | **101.07** | 1359.68 | 1342.65 | 1341.67 | **1387.67** | 1370.65 | 1369.66 | **Q** | 1266.68 | 1265.68 |  | **1339.73** | 1322.71 | 1321.72 | **12** |
| **13** | **70.07** | 1456.73 | 1439.70 | 1438.72 | 1484.73 | 1467.70 | 1466.71 | **P** | 1169.63 | 1168.63 |  | **1211.67** | 1194.65 | 1193.66 | **11** |
| **14** | **101.07** | 1584.79 | 1567.76 | 1566.78 | 1612.78 | 1595.76 | 1594.77 | **Q** | 1041.57 | 1040.57 |  | 1114.62 | 1097.59 | 1096.61 | **10** |
| **15** | **72.08** | 1683.86 | 1666.83 | 1665.85 | 1711.85 | 1694.83 | 1693.84 | **V** | 942.50 | 955.52 |  | **986.56** | 969.54 | 968.55 | **9** |
| **16** | **86.10** | 1796.94 | 1779.91 | 1778.93 | 1824.94 | 1807.91 | 1806.93 | **L** | 829.42 | 828.42 |  | **887.49** | 870.47 | 869.48 | **8** |
| **17** | **86.10** | 1910.03 | 1893.00 | 1892.02 | 1938.02 | 1920.99 | 1920.01 | **L** | 716.33 | 715.34 |  | **774.41** | 757.38 | 756.40 | **7** |
| **18** | 60.04 | 1997.06 | 1980.03 | 1979.05 | 2025.05 | 2008.03 | 2007.04 | **S** | 629.30 | 628.30 |  | **661.33** | 644.30 | 643.32 | **6** |
| **19** | **70.07** | 2094.11 | 2077.08 | 2076.10 | 2122.11 | 2105.08 | 2104.09 | **P** | 532.25 | 531.25 |  | **574.29** | **557.27** | 556.28 | **5** |
| **20** | 74.06 | 2195.16 | 2178.13 | 2177.15 | 2223.15 | 2206.13 | 2205.14 | **T** | 431.20 | 444.22 | **446.20** | 477.24 | 460.22 | 459.23 | **4** |
| **21** | 60.04 | 2282.19 | 2265.16 | 2264.18 | 2310.18 | 2293.16 | 2292.17 | **S** | 344.17 | 343.17 |  | **376.19** | 359.17 | 358.18 | **3** |
| **22** | 87.06 | 2396.23 | 2379.21 | 2378.22 | 2424.23 | 2407.20 | 2406.22 | **N** | 230.12 | 229.13 |  | **289.16** | **272.14** |  | **2** |
| **23** | 129.11 |  |  |  |  |  |  | **R** | 74.02 | 73.03 |  | **175.12** | 158.09 |  | **1** |

| **Seq** | **ya** | **yb** | **Seq** | **ya** | **yb** | **Seq** | **ya** | **yb** |
| --- | --- | --- | --- | --- | --- | --- | --- | --- |
| **TG** | 131.08 | 159.08 | **TGN** | 245.12 | 273.12 | **TGNH** | 382.18 | **410.18** |
| **TGNHA** | 453.22 | **481.22** | **TGNHAL** | 566.30 | 594.30 | **GN** | 144.08 | 172.07 |
| **GNH** | 281.14 | 309.13 | **GNHA** | 352.17 | 380.17 | **GNHAL** | 465.26 | 493.25 |
| **GNHALY** | 628.32 | 656.32 | **GNHALYA** | 699.36 | 727.35 | **NH** | 224.11 | 252.11 |
| **NHA** | 295.15 | 323.15 | **NHAL** | 408.24 | 436.23 | **NHALY** | 571.30 | 599.29 |
| **NHALYA** | 642.34 | 670.33 | **HA** | 181.11 | **209.10** | **HAL** | 294.19 | 322.19 |
| **HALY** | 457.26 | 485.25 | **HALYA** | 528.29 | 556.29 | **HALYAT** | 629.34 | 657.34 |
| **AL** | 157.13 | **185.13** | **ALY** | 320.20 | 348.19 | **ALYA** | 391.23 | 419.23 |
| **ALYAT** | 492.28 | 520.28 | **ALYATE** | 621.32 | 649.32 | **LY** | 249.16 | 277.15 |
| **LYA** | 320.20 | 348.19 | **LYAT** | 421.24 | 449.24 | **LYATE** | 550.29 | 578.28 |
| **LYATEQ** | 678.35 | 706.34 | **YA** | 207.11 | 235.11 | **YAT** | 308.16 | 336.16 |
| **YATE** | 437.20 | 465.20 | **YATEQ** | 565.26 | 593.26 | **YATEQP** | 662.31 | 690.31 |
| **AT** | 145.10 | 173.09 | **ATE** | 274.14 | 302.13 | **ATEQ** | 402.20 | **430.19** |
| **ATEQP** | 499.25 | 527.25 | **ATEQPQ** | 627.31 | 655.30 | **TE** | 203.10 | 231.10 |
| **TEQ** | 331.16 | 359.16 | **TEQP** | 428.21 | 456.21 | **TEQPQ** | 556.27 | 584.27 |
| **TEQPQV** | 655.34 | 683.34 | **EQ** | 230.11 | 258.11 | **EQP** | 327.17 | 355.16 |
| **EQPQ** | 455.22 | 483.22 | **EQPQV** | 554.29 | 582.29 | **EQPQVL** | 667.38 | 695.37 |
| **QP** | **198.12** | **226.12** | **QPQ** | 326.18 | **354.18** | **QPQV** | 425.25 | 453.25 |
| **QPQVL** | 538.33 | 566.33 | **QPQVLL** | 651.42 | 679.41 | **PQ** | **198.12** | **226.12** |
| **PQV** | 297.19 | **325.19** | **PQVL** | **410.28** | **438.27** | **PQVLL** | 523.36 | 551.36 |
| **PQVLLS** | 610.39 | 638.39 | **QV** | 200.14 | **228.13** | **QVL** | 313.22 | 341.22 |
| **QVLL** | 426.31 | 454.30 | **QVLLS** | 513.34 | 541.33 | **QVLLSP** | 610.39 | 638.39 |
| **VL** | **185.16** | 213.16 | **VLL** | 298.25 | 326.24 | **VLLS** | 385.28 | 413.28 |
| **VLLSP** | 482.33 | 510.33 | **VLLSPT** | **583.38** | **611.38** | **VLLSPTS** | 670.41 | 698.41 |
| **LL** | 199.18 | 227.18 | **LLS** | 286.21 | 314.21 | **LLSP** | 383.27 | 411.26 |
| **LLSPT** | 484.31 | 512.31 | **LLSPTS** | 571.34 | 599.34 | **LLSPTSN** | **685.39** | 713.38 |
| **LS** | 173.13 | 201.12 | **LSP** | 270.18 | 298.18 | **LSPT** | 371.23 | 399.22 |
| **LSPTS** | 458.26 | 486.26 | **LSPTSN** | 572.30 | 600.30 | **SP** | 157.10 | **185.09** |
| **SPT** | 258.14 | 286.14 | **SPTS** | 345.18 | 373.17 | **SPTSN** | 459.22 | 487.21 |
| **PT** | 171.11 | 199.11 | **PTS** | 258.14 | 286.14 | **PTSN** | 372.19 | 400.18 |
| **TS** | 161.09 | 189.09 | **TSN** | 275.14 | **303.13** | **SN** | 174.09 | **202.08** |

5. Tb11.01.3550

Match to: **Tb11.01.3550** Score: **98**

**2-oxoglutarate dehydrogenase E2 component, putative; Trypanosoma bruceichr 11Manual**

Nominal mass (M_r_): **45328**; Calculated pI value: **8.37**

NCBI BLAST search of [Tb11.01.3550](http://www.ncbi.nlm.nih.gov/blast/Blast.cgi?ALIGNMENTS=50&ALIGNMENT_VIEW=Pairwise&AUTO_FORMAT=Semiauto&CDD_SEARCH=on&CLIENT=web&COMPOSITION_BASED_STATISTICS=on&DATABASE=nr&DESCRIPTIONS=100&ENTREZ_QUERY=(none)&EXPECT=10&FILTER=L&FORMAT_BLOCK_ON_RESPAGE=None&FORMAT_OBJECT=Alignment&FORMAT_TYPE=HTML&GAPCOSTS=11+1&I_THRESH=0.001&LAYOUT=TwoWindows&MATRIX_NAME=BLOSUM62&NCBI_GI=on&PAGE=Proteins&PROGRAM=blastp&QUERY=MLRRLATHGLQATCLTSEKLAYRYCLSICVPTIAESISSGKVVGWTKKVGDAVAEDEIICQIESDKLNVDVRAPAAGVITKINFEEGTVVDVGAELSTMKEGEAPAAKAETADKPKQNAPAAAAPPKASPTEAAPKPAPAAAPVTSRGADPRVRSVRISSMRQRIADRLKASQNTCAMLTTFNEIDMTPLIELRNRYKDDFFKKNGVKLGFMSPFVKACAIALQDVPIVNASFGTDCIEYHDYVDISVAVSTPKGLVVPVLRDVQNSNFAQIEKQIADFGERARSNKLTMAEMTGGTFTISNGGVFGSWMGTPIVNPPQSAILGMHATKKKPWVVGNSVVPRDIMAVALTYDHRLIDGSDAVTFLVKVKNLIEDPARIVLDLA&SERVICE=plain&SET_DEFAULTS.x=9&SET_DEFAULTS.y=5&SHOW_OVERVIEW=on&WORD_SIZE=3&END_OF_HTTPGET=Yes) against nr

Unformatted [sequence string](../../../../D:%5CProteomic%20data%5C2010-1-8%5Ccgi%5Cgetseq.pl%3FTBA927_IPI+Tb11%2E01%2E3550+seq) for pasting into other applications

Fixed modifications: MMTS (C),(N-TERM)_iTRAQ,Lysine(K)_iTRAQ

Variable modifications: Oxidation (M)

Cleavage by Trypsin: cuts C-term side of KR unless next residue is P

Sequence Coverage: **7%**

Matched peptides shown in **Bold Red**

**1** MLRRLATHGL QATCLTSEKL AYRYCLSICV PTIAESISSG KVVGWTKKVG

**51** DAVAEDEIIC QIESDKLNVD VRAPAAGVIT KINFEEGTVV DVGAELSTMK

**101** EGEAPAAKAE TADKPKQNAP AAAAPPK**ASP TEAAPKPAPA AAPVTSR**GAD

**151** PRVRSVRISS MRQRIADRLK ASQNTCAMLT TFNEIDMTPL IELRNRYKDD

**201** FFKKNGVKLG FMSPFVKACA IALQDVPIVN ASFGTDCIEY HDYVDISVAV

**251** STPKGLVVPV LRDVQNSNFA QIEK**QIADFG ER**ARSNKLTM AEMTGGTFTI

**301** SNGGVFGSWM GTPIVNPPQS AILGMHATKK KPWVVGNSVV PRDIMAVALT

**351** YDHRLIDGSD AVTFLVKVKN LIEDPARIVL DLA

**Start - End Observed Mr(expt) Mr(calc) Delta Miss Sequence**

**128 - 147 2178.21 2177.20 2177.12 0.08 0 K.ASPTEAAPKPAPAAAPVTSR.G**  ([Ions score 98](../../../../D:%5CProteomic%20data%5C2010-1-8%5CZQ%5C947.htm))

**275 - 282 1079.58 1078.57 1078.55 0.02 0 K.QIADFGER.A**  ([Ions score 9](../../../../D:%5CProteomic%20data%5C2010-1-8%5CZQ%5C946.htm))

MS/MS Fragmentation of **ASPTEAAPKPAPAAAPVTSR**
Found in **Tb11.01.3550**, 2-oxoglutarate dehydrogenase E2 component, putative; Trypanosoma bruceichr 11Manual


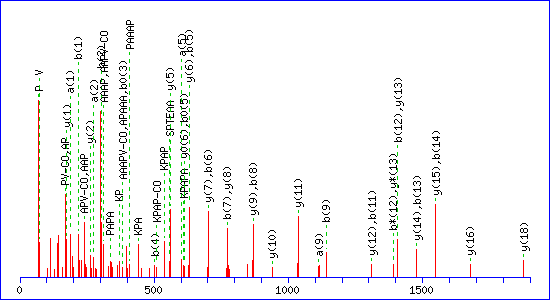


**MONOISOTOPIC mass of neutral peptide Mr(calc):** 2177.12

**Fixed modifications:** MMTS (C),(N-TERM)_iTRAQ,Lysine(K)_iTRAQ

**Ions Score:** 98 **Expect:** 1.3e-008

**Matches (Bold Red):** 76/366 fragment ions using 53 most intense peaks

| **#** | **Immon.** | **a** | **a*** | **a^0^** | **b** | **b*** | **b^0^** | **Seq.** | **v** | **w** | **w'** | **y** | **y*** | **y^0^** | **#** |
| --- | --- | --- | --- | --- | --- | --- | --- | --- | --- | --- | --- | --- | --- | --- | --- |
| **1** | 44.05 | **188.15** |  |  | **216.15** |  |  | **A** |  |  |  |  |  |  | **20** |
| **2** | 60.04 | **275.18** |  | 257.17 | **303.18** |  | 285.17 | **S** | 1930.96 | 1929.96 |  | 1962.98 | 1945.96 | 1944.97 | **19** |
| **3** | **70.07** | 372.24 |  | 354.23 | 400.23 |  | **382.22** | **P** | 1833.90 | 1832.91 |  | **1875.95** | 1858.92 | 1857.94 | **18** |
| **4** | 74.06 | 473.28 |  | 455.27 | **501.28** |  | 483.27 | **T** | 1732.86 | 1745.88 | 1747.86 | 1778.90 | 1761.87 | 1760.89 | **17** |
| **5** | 102.05 | **602.33** |  | 584.32 | **630.32** |  | **612.31** | **E** | 1603.81 | 1602.82 |  | **1677.85** | 1660.82 | 1659.84 | **16** |
| **6** | 44.05 | 673.36 |  | 655.35 | **701.36** |  | 683.35 | **A** | 1532.78 |  |  | **1548.81** | 1531.78 | 1530.80 | **15** |
| **7** | 44.05 | 744.40 |  | 726.39 | **772.40** |  | 754.39 | **A** | 1461.74 |  |  | **1477.77** | 1460.74 | 1459.76 | **14** |
| **8** | **70.07** | 841.45 |  | 823.44 | **869.45** |  | 851.44 | **P** | 1364.69 | 1363.69 |  | **1406.73** | **1389.71** | 1388.72 | **13** |
| **9** | 245.12 | **1113.56** | 1096.53 | 1095.55 | **1141.56** | 1124.53 | 1123.55 | **K** | 1092.58 | 1091.58 |  | **1309.68** | 1292.65 | 1291.67 | **12** |
| **10** | **70.07** | 1210.61 | 1193.59 | 1192.60 | 1238.61 | 1221.58 | 1220.60 | **P** | 995.53 | 994.53 |  | **1037.57** | 1020.55 | 1019.56 | **11** |
| **11** | 44.05 | 1281.65 | 1264.62 | 1263.64 | **1309.65** | 1292.62 | 1291.64 | **A** | 924.49 |  |  | **940.52** | 923.49 | 922.51 | **10** |
| **12** | **70.07** | 1378.70 | 1361.68 | 1360.69 | **1406.70** | **1389.67** | 1388.69 | **P** | 827.44 | 826.44 |  | **869.48** | 852.46 | 851.47 | **9** |
| **13** | 44.05 | 1449.74 | 1432.71 | 1431.73 | **1477.74** | 1460.71 | 1459.73 | **A** | 756.40 |  |  | **772.43** | 755.40 | 754.42 | **8** |
| **14** | 44.05 | 1520.78 | 1503.75 | 1502.77 | **1548.77** | 1531.75 | 1530.76 | **A** | 685.36 |  |  | **701.39** | 684.37 | 683.38 | **7** |
| **15** | 44.05 | 1591.82 | 1574.79 | 1573.80 | 1619.81 | 1602.78 | 1601.80 | **A** | 614.33 |  |  | **630.36** | 613.33 | **612.35** | **6** |
| **16** | **70.07** | 1688.87 | 1671.84 | 1670.86 | 1716.86 | 1699.84 | 1698.85 | **P** | 517.27 | 516.28 |  | **559.32** | 542.29 | 541.31 | **5** |
| **17** | **72.08** | 1787.94 | 1770.91 | 1769.93 | 1815.93 | 1798.90 | 1797.92 | **V** | 418.20 | 431.22 |  | 462.27 | 445.24 | 444.26 | **4** |
| **18** | 74.06 | 1888.98 | 1871.96 | 1870.97 | 1916.98 | 1899.95 | 1898.97 | **T** | 317.16 | 330.18 | 332.16 | 363.20 | 346.17 | 345.19 | **3** |
| **19** | 60.04 | 1976.02 | 1958.99 | 1958.01 | 2004.01 | 1986.98 | 1986.00 | **S** | 230.12 | 229.13 |  | **262.15** | 245.12 | 244.14 | **2** |
| **20** | 129.11 |  |  |  |  |  |  | **R** | 74.02 | 73.03 |  | **175.12** | 158.09 |  | **1** |

| **Seq** | **ya** | **yb** | **Seq** | **ya** | **yb** | **Seq** | **ya** | **yb** |
| --- | --- | --- | --- | --- | --- | --- | --- | --- |
| **SP** | 157.10 | 185.09 | **SPT** | 258.14 | 286.14 | **SPTE** | 387.19 | 415.18 |
| **SPTEA** | 458.22 | 486.22 | **SPTEAA** | 529.26 | **557.26** | **SPTEAAP** | 626.31 | 654.31 |
| **PT** | 171.11 | 199.11 | **PTE** | 300.16 | 328.15 | **PTEA** | 371.19 | 399.19 |
| **PTEAA** | 442.23 | 470.22 | **PTEAAP** | 539.28 | 567.28 | **TE** | 203.10 | 231.10 |
| **TEA** | 274.14 | 302.13 | **TEAA** | 345.18 | 373.17 | **TEAAP** | 442.23 | 470.22 |
| **EA** | 173.09 | 201.09 | **EAA** | 244.13 | 272.12 | **EAAP** | 341.18 | 369.18 |
| **EAAPK** | 613.29 | 641.28 | **AA** | 115.09 | 143.08 | **AAP** | 212.14 | **240.13** |
| **AAPK** | 484.25 | 512.24 | **AAPKP** | 581.30 | **609.29** | **AAPKPA** | 652.34 | 680.33 |
| **AP** | 141.10 | **169.10** | **APK** | 413.21 | **441.20** | **APKP** | **510.26** | **538.26** |
| **APKPA** | 581.30 | **609.29** | **APKPAP** | 678.35 | 706.35 | **PK** | 342.17 | **370.17** |
| **PKP** | 439.23 | 467.22 | **PKPA** | **510.26** | **538.26** | **PKPAP** | 607.32 | 635.31 |
| **PKPAPA** | 678.35 | 706.35 | **KP** | 342.17 | **370.17** | **KPA** | 413.21 | **441.20** |
| **KPAP** | **510.26** | **538.26** | **KPAPA** | 581.30 | **609.29** | **KPAPAA** | 652.34 | 680.33 |
| **PA** | 141.10 | **169.10** | **PAP** | 238.15 | 266.15 | **PAPA** | 309.19 | **337.19** |
| **PAPAA** | 380.23 | **408.22** | **PAPAAA** | 451.27 | 479.26 | **PAPAAAP** | 548.32 | 576.31 |
| **PAPAAAPV** | 647.39 | 675.38 | **AP** | 141.10 | **169.10** | **APA** | 212.14 | **240.13** |
| **APAA** | 283.18 | **311.17** | **APAAA** | 354.21 | **382.21** | **APAAAP** | 451.27 | 479.26 |
| **APAAAPV** | 550.33 | 578.33 | **APAAAPVT** | 651.38 | 679.38 | **PA** | 141.10 | **169.10** |
| **PAA** | 212.14 | **240.13** | **PAAA** | 283.18 | **311.17** | **PAAAP** | 380.23 | **408.22** |
| **PAAAPV** | 479.30 | 507.29 | **PAAAPVT** | 580.35 | 608.34 | **PAAAPVTS** | 667.38 | 695.37 |
| **AA** | 115.09 | 143.08 | **AAA** | 186.12 | 214.12 | **AAAP** | 283.18 | **311.17** |
| **AAAPV** | **382.24** | 410.24 | **AAAPVT** | 483.29 | 511.29 | **AAAPVTS** | 570.32 | 598.32 |
| **AA** | 115.09 | 143.08 | **AAP** | 212.14 | **240.13** | **AAPV** | **311.21** | 339.20 |
| **AAPVT** | 412.26 | 440.25 | **AAPVTS** | 499.29 | 527.28 | **AP** | 141.10 | **169.10** |
| **APV** | **240.17** | 268.17 | **APVT** | 341.22 | 369.21 | **APVTS** | 428.25 | 456.25 |
| **PV** | **169.13** | 197.13 | **PVT** | 270.18 | 298.18 | **PVTS** | 357.21 | 385.21 |
| **VT** | 173.13 | 201.12 | **VTS** | 260.16 | 288.16 | **TS** | 161.09 | 189.09 |

6. Tb09.211.2250

Match to: **Tb09.211.2250** Score: **98**

**hypothetical protein, conserved; Trypanosoma bruceichr 9Manual**

Nominal mass (M_r_): **32937**; Calculated pI value: **4.93**

NCBI BLAST search of [Tb09.211.2250](http://www.ncbi.nlm.nih.gov/blast/Blast.cgi?ALIGNMENTS=50&ALIGNMENT_VIEW=Pairwise&AUTO_FORMAT=Semiauto&CDD_SEARCH=on&CLIENT=web&COMPOSITION_BASED_STATISTICS=on&DATABASE=nr&DESCRIPTIONS=100&ENTREZ_QUERY=(none)&EXPECT=10&FILTER=L&FORMAT_BLOCK_ON_RESPAGE=None&FORMAT_OBJECT=Alignment&FORMAT_TYPE=HTML&GAPCOSTS=11+1&I_THRESH=0.001&LAYOUT=TwoWindows&MATRIX_NAME=BLOSUM62&NCBI_GI=on&PAGE=Proteins&PROGRAM=blastp&QUERY=METFRERTLANRYQPRQHLPPEFPGVLKEYAREVLREQPEDILQWSANYFKRLAREMDAKPAGEHRMTAPALPHLESRPNTREEEEEEYGERLQQYIDMFVEHDSENNGVLPVKAIKEALLKSYGLTLPQALYVLTATQLVEKEPVNYAEFACESFPALRFVWSTEHNFQVSNREDTTVHGLSRIDVQQEFLKLLRFADRSDTSLLSIDQYMDVLRCAPYHLTTRDLRILRVEAELNERHEVNYEEELLHIFDRLLLAEQFAQLDSDD&SERVICE=plain&SET_DEFAULTS.x=9&SET_DEFAULTS.y=5&SHOW_OVERVIEW=on&WORD_SIZE=3&END_OF_HTTPGET=Yes) against nr

Unformatted [sequence string](../../../../D:%5CProteomic%20data%5C2010-1-8%5Ccgi%5Cgetseq.pl%3FTBA927_IPI+Tb09%2E211%2E2250+seq) for pasting into other applications

Fixed modifications: MMTS (C),(N-TERM)_iTRAQ,Lysine(K)_iTRAQ

Variable modifications: Oxidation (M)

Cleavage by Trypsin: cuts C-term side of KR unless next residue is P

Sequence Coverage: **9%**

Matched peptides shown in **Bold Red**

**1** METFRERTLA NRYQPRQHLP PEFPGVLKEY AREVLREQPE DILQWSANYF

**51** KRLAREMDAK PAGEHRMTAP ALPHLESRPN TREEEEEEYG ERLQQYIDMF

**101** VEHDSENNGV LPVKAIKEAL LKSYGLTLPQ ALYVLTATQL VEKEPVNYAE

**151** FACESFPALR FVWSTEHNFQ VSNR**EDTTVH GLSR**IDVQQE FLKLLRFADR

**201** **SDTSLLSIDQ YMDVLR**CAPY HLTTRDLRIL RVEAELNERH EVNYEEELLH

**251** IFDRLLLAEQ FAQLDSDD

**Start - End Observed Mr(expt) Mr(calc) Delta Miss Sequence**

**175 - 184 1258.62 1257.61 1257.64 -0.03 0 R.EDTTVHGLSR.I**  ([Ions score 8](../../../../D:%5CProteomic%20data%5C2010-1-8%5CZQ%5C949.htm))

**201 - 216 2000.00 1999.00 1999.01 -0.01 0 R.SDTSLLSIDQYMDVLR.C**  ([Ions score 98](../../../../D:%5CProteomic%20data%5C2010-1-8%5CZQ%5C950.htm))

MS/MS Fragmentation of **SDTSLLSIDQYMDVLR**
Found in **Tb09.211.2250**, hypothetical protein, conserved; Trypanosoma bruceichr 9Manual


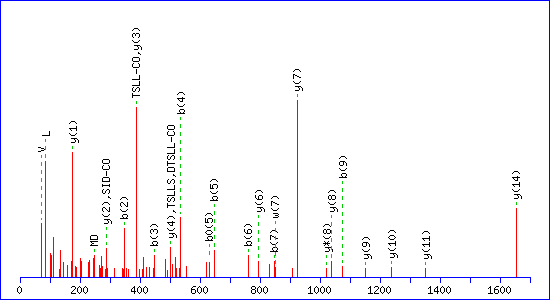


**MONOISOTOPIC mass of neutral peptide Mr(calc):** 1999.01

**Fixed modifications:** MMTS (C),(N-TERM)_iTRAQ,Lysine(K)_iTRAQ

**Ions Score:** 98 **Expect:** 1.8e-008

**Matches (Bold Red):** 31/262 fragment ions using 25 most intense peaks

| **#** | **Immon.** | **a** | **a*** | **a^0^** | **b** | **b*** | **b^0^** | **Seq.** | **v** | **w** | **w'** | **y** | **y*** | **y^0^** | **#** |
| --- | --- | --- | --- | --- | --- | --- | --- | --- | --- | --- | --- | --- | --- | --- | --- |
| **1** | 60.04 | 204.15 |  | 186.14 | 232.14 |  | 214.13 | **S** |  |  |  |  |  |  | **16** |
| **2** | 88.04 | 319.17 |  | 301.16 | **347.17** |  | 329.16 | **D** | 1708.86 | 1707.86 |  | 1768.88 | 1751.85 | 1750.87 | **15** |
| **3** | 74.06 | 420.22 |  | 402.21 | **448.22** |  | 430.21 | **T** | 1607.81 | 1620.83 | 1622.81 | **1653.85** | 1636.83 | 1635.84 | **14** |
| **4** | 60.04 | 507.25 |  | 489.24 | **535.25** |  | 517.24 | **S** | 1520.78 | 1519.78 |  | 1552.80 | 1535.78 | 1534.79 | **13** |
| **5** | **86.10** | 620.34 |  | 602.33 | **648.33** |  | **630.32** | **L** | 1407.69 | 1406.70 |  | 1465.77 | 1448.75 | 1447.76 | **12** |
| **6** | **86.10** | 733.42 |  | 715.41 | **761.42** |  | 743.41 | **L** | 1294.61 | 1293.61 |  | **1352.69** | 1335.66 | 1334.68 | **11** |
| **7** | 60.04 | 820.45 |  | 802.44 | **848.45** |  | 830.44 | **S** | 1207.58 | 1206.58 |  | **1239.60** | 1222.58 | 1221.59 | **10** |
| **8** | **86.10** | 933.54 |  | 915.53 | 961.53 |  | 943.52 | **I** | 1094.49 | 1107.51 | 1121.53 | **1152.57** | 1135.55 | 1134.56 | **9** |
| **9** | 88.04 | 1048.56 |  | 1030.55 | **1076.56** |  | 1058.55 | **D** | 979.47 | 978.47 |  | **1039.49** | **1022.46** | 1021.48 | **8** |
| **10** | 101.07 | 1176.62 | 1159.60 | 1158.61 | 1204.62 | 1187.59 | 1186.61 | **Q** | 851.41 | **850.41** |  | **924.46** | 907.43 | 906.45 | **7** |
| **11** | 136.08 | 1339.69 | 1322.66 | 1321.68 | 1367.68 | 1350.65 | 1349.67 | **Y** | 688.34 |  |  | **796.40** | 779.38 | 778.39 | **6** |
| **12** | 104.05 | 1470.73 | 1453.70 | 1452.72 | 1498.72 | 1481.70 | 1480.71 | **M** | 557.30 | 556.31 |  | 633.34 | 616.31 | 615.33 | **5** |
| **13** | 88.04 | 1585.75 | 1568.73 | 1567.74 | 1613.75 | 1596.72 | 1595.74 | **D** | 442.28 | 441.28 |  | **502.30** | 485.27 | 484.29 | **4** |
| **14** | **72.08** | 1684.82 | 1667.80 | 1666.81 | 1712.82 | 1695.79 | 1694.81 | **V** | 343.21 | 356.23 |  | **387.27** | 370.24 |  | **3** |
| **15** | **86.10** | 1797.91 | 1780.88 | 1779.90 | 1825.90 | 1808.87 | 1807.89 | **L** | 230.12 | 229.13 |  | **288.20** | 271.18 |  | **2** |
| **16** | 129.11 |  |  |  |  |  |  | **R** | 74.02 | 73.03 |  | **175.12** | 158.09 |  | **1** |

| **Seq** | **ya** | **yb** | **Seq** | **ya** | **yb** | **Seq** | **ya** | **yb** |
| --- | --- | --- | --- | --- | --- | --- | --- | --- |
| **DT** | 189.09 | 217.08 | **DTS** | 276.12 | 304.11 | **DTSL** | 389.20 | 417.20 |
| **DTSLL** | **502.29** | 530.28 | **DTSLLS** | 589.32 | 617.31 | **TS** | 161.09 | 189.09 |
| **TSL** | 274.18 | 302.17 | **TSLL** | **387.26** | 415.26 | **TSLLS** | 474.29 | **502.29** |
| **TSLLSI** | 587.38 | 615.37 | **SL** | 173.13 | 201.12 | **SLL** | 286.21 | 314.21 |
| **SLLS** | 373.24 | 401.24 | **SLLSI** | 486.33 | 514.32 | **SLLSID** | 601.36 | 629.35 |
| **LL** | 199.18 | 227.18 | **LLS** | 286.21 | 314.21 | **LLSI** | 399.30 | 427.29 |
| **LLSID** | 514.32 | 542.32 | **LLSIDQ** | 642.38 | 670.38 | **LS** | 173.13 | 201.12 |
| **LSI** | 286.21 | 314.21 | **LSID** | 401.24 | 429.23 | **LSIDQ** | 529.30 | 557.29 |
| **LSIDQY** | 692.36 | 720.36 | **SI** | 173.13 | 201.12 | **SID** | **288.16** | 316.15 |
| **SIDQ** | 416.21 | 444.21 | **SIDQY** | 579.28 | 607.27 | **ID** | 201.12 | 229.12 |
| **IDQ** | 329.18 | 357.18 | **IDQY** | 492.25 | 520.24 | **IDQYM** | 623.29 | 651.28 |
| **DQ** | 216.10 | 244.09 | **DQY** | 379.16 | 407.16 | **DQYM** | 510.20 | 538.20 |
| **DQYMD** | 625.23 | 653.22 | **QY** | 264.13 | 292.13 | **QYM** | 395.17 | 423.17 |
| **QYMD** | 510.20 | 538.20 | **QYMDV** | 609.27 | 637.27 | **YM** | 267.12 | 295.11 |
| **YMD** | 382.14 | 410.14 | **YMDV** | 481.21 | 509.21 | **YMDVL** | 594.30 | 622.29 |
| **MD** | 219.08 | **247.07** | **MDV** | 318.15 | 346.14 | **MDVL** | 431.23 | 459.23 |
| **DV** | 187.11 | 215.10 | **DVL** | 300.19 | 328.19 | **VL** | 185.16 | 213.16 |

7. Tb927.8.5440

Match to: **Tb927.8.5440** Score: **95**

**Tb-24flagellar calcium-binding protein; Trypanosoma bruceichr 8Manual**

Nominal mass (M_r_): **27682**; Calculated pI value: **4.75**

NCBI BLAST search of [Tb927.8.5440](http://www.ncbi.nlm.nih.gov/blast/Blast.cgi?ALIGNMENTS=50&ALIGNMENT_VIEW=Pairwise&AUTO_FORMAT=Semiauto&CDD_SEARCH=on&CLIENT=web&COMPOSITION_BASED_STATISTICS=on&DATABASE=nr&DESCRIPTIONS=100&ENTREZ_QUERY=(none)&EXPECT=10&FILTER=L&FORMAT_BLOCK_ON_RESPAGE=None&FORMAT_OBJECT=Alignment&FORMAT_TYPE=HTML&GAPCOSTS=11+1&I_THRESH=0.001&LAYOUT=TwoWindows&MATRIX_NAME=BLOSUM62&NCBI_GI=on&PAGE=Proteins&PROGRAM=blastp&QUERY=MGCSGSKNTTNSKDGAASKGGKDGKTTADRKVAWERIRCAIPRDKDAESKSRRIELFKQFDTNGTGKLGFREVLDGCYSVLKLDEFTTHLPDIVQRAFDKAKDLGNKVKGVGEEDLVEFLEFRLMLCYIYDIFELTVMFDTMDKDGSLLLELQEFKEALPKLKEWGVDITDATTVFNEIDTNGSGVVTFDEFSCWAVTKKLQVCGDPDGEENGANEGN&SERVICE=plain&SET_DEFAULTS.x=9&SET_DEFAULTS.y=5&SHOW_OVERVIEW=on&WORD_SIZE=3&END_OF_HTTPGET=Yes) against nr

Unformatted [sequence string](../../../../D:%5CProteomic%20data%5C2010-1-8%5Ccgi%5Cgetseq.pl%3FTBA927_IPI+Tb927%2E8%2E5440+seq) for pasting into other applications

Fixed modifications: MMTS (C),(N-TERM)_iTRAQ,Lysine(K)_iTRAQ

Variable modifications: Oxidation (M)

Cleavage by Trypsin: cuts C-term side of KR unless next residue is P

Sequence Coverage: **6%**

Matched peptides shown in **Bold Red**

**1** MGCSGSKNTT NSKDGAASKG GKDGKTTADR KVAWERIRCA IPRDKDAESK

**51** SRRIELFKQF DTNGTGKLGF REVLDGCYSV LKLDEFTTHL PDIVQRAFDK

**101** AKDLGNKVK**G VGEEDLVEFL EFR**LMLCYIY DIFELTVMFD TMDKDGSLLL

**151** ELQEFKEALP KLKEWGVDIT DATTVFNEID TNGSGVVTFD EFSCWAVTKK

**201** LQVCGDPDGE ENGANEGN

MS/MS Fragmentation of **GVGEEDLVEFLEFR**
Found in **Tb927.8.5440**, Tb-24flagellar calcium-binding protein; Trypanosoma bruceichr 8Manual


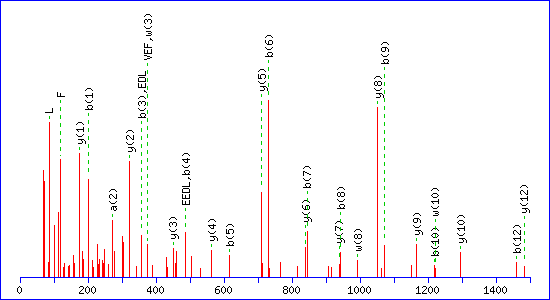


**MONOISOTOPIC mass of neutral peptide Mr(calc):** 1781.90

**Fixed modifications:** MMTS (C),(N-TERM)_iTRAQ,Lysine(K)_iTRAQ

**Ions Score:** 95 **Expect:** 4.2e-008

**Matches (Bold Red):** 32/201 fragment ions using 27 most intense peaks

| **#** | **Immon.** | **a** | **a^0^** | **b** | **b^0^** | **Seq.** | **v** | **w** | **y** | **y*** | **y^0^** | **#** |
| --- | --- | --- | --- | --- | --- | --- | --- | --- | --- | --- | --- | --- |
| **1** | 30.03 | 174.14 |  | **202.13** |  | **G** |  |  |  |  |  | **14** |
| **2** | 72.08 | **273.20** |  | 301.20 |  | **V** | 1537.72 | 1550.74 | 1581.78 | 1564.75 | 1563.77 | **13** |
| **3** | 30.03 | 330.23 |  | **358.22** |  | **G** |  |  | **1482.71** | 1465.68 | 1464.70 | **12** |
| **4** | 102.05 | 459.27 | 441.26 | **487.26** | 469.25 | **E** | 1351.65 | 1350.66 | 1425.69 | 1408.66 | 1407.68 | **11** |
| **5** | 102.05 | 588.31 | 570.30 | **616.31** | 598.30 | **E** | 1222.61 | **1221.61** | **1296.65** | 1279.62 | 1278.64 | **10** |
| **6** | 88.04 | 703.34 | 685.33 | **731.33** | 713.32 | **D** | 1107.58 | 1106.59 | **1167.60** | 1150.58 | 1149.59 | **9** |
| **7** | **86.10** | 816.42 | 798.41 | **844.42** | 826.41 | **L** | 994.50 | **993.50** | **1052.58** | 1035.55 | 1034.57 | **8** |
| **8** | 72.08 | 915.49 | 897.48 | **943.49** | 925.48 | **V** | 895.43 | 908.45 | **939.49** | 922.47 | 921.48 | **7** |
| **9** | 102.05 | 1044.53 | 1026.52 | **1072.53** | 1054.52 | **E** | 766.39 | 765.39 | **840.43** | 823.40 | 822.41 | **6** |
| **10** | **120.08** | 1191.60 | 1173.59 | **1219.60** | 1201.59 | **F** | 619.32 |  | **711.38** | 694.36 | 693.37 | **5** |
| **11** | **86.10** | 1304.69 | 1286.68 | 1332.68 | 1314.67 | **L** | 506.24 | 505.24 | **564.31** | 547.29 | 546.30 | **4** |
| **12** | 102.05 | 1433.73 | 1415.72 | **1461.72** | 1443.71 | **E** | 377.19 | **376.20** | **451.23** | 434.20 | 433.22 | **3** |
| **13** | **120.08** | 1580.80 | 1562.79 | 1608.79 | 1590.78 | **F** | 230.12 |  | **322.19** | 305.16 |  | **2** |
| **14** | 129.11 |  |  |  |  | **R** | 74.02 | 73.03 | **175.12** | 158.09 |  | **1** |

| **Seq** | **ya** | **yb** | **Seq** | **ya** | **yb** | **Seq** | **ya** | **yb** |
| --- | --- | --- | --- | --- | --- | --- | --- | --- |
| **VG** | 129.10 | 157.10 | **VGE** | 258.14 | 286.14 | **VGEE** | 387.19 | 415.18 |
| **VGEED** | 502.21 | 530.21 | **VGEEDL** | 615.30 | 643.29 | **GE** | 159.08 | 187.07 |
| **GEE** | 288.12 | 316.11 | **GEED** | 403.15 | 431.14 | **GEEDL** | 516.23 | 544.22 |
| **GEEDLV** | 615.30 | 643.29 | **EE** | 231.10 | 259.09 | **EED** | 346.12 | 374.12 |
| **EEDL** | 459.21 | **487.20** | **EEDLV** | 558.28 | 586.27 | **EEDLVE** | 687.32 | 715.31 |
| **ED** | 217.08 | 245.08 | **EDL** | 330.17 | **358.16** | **EDLV** | 429.23 | 457.23 |
| **EDLVE** | 558.28 | 586.27 | **DL** | 201.12 | 229.12 | **DLV** | 300.19 | 328.19 |
| **DLVE** | 429.23 | 457.23 | **DLVEF** | 576.30 | 604.30 | **DLVEFL** | 689.39 | 717.38 |
| **LV** | 185.16 | 213.16 | **LVE** | 314.21 | 342.20 | **LVEF** | 461.28 | 489.27 |
| **LVEFL** | 574.36 | 602.35 | **VE** | 201.12 | 229.12 | **VEF** | 348.19 | **376.19** |
| **VEFL** | 461.28 | 489.27 | **VEFLE** | 590.32 | 618.31 | **EF** | 249.12 | 277.12 |
| **EFL** | 362.21 | 390.20 | **EFLE** | 491.25 | 519.24 | **EFLEF** | 638.32 | 666.31 |
| **FL** | 233.16 | 261.16 | **FLE** | 362.21 | 390.20 | **FLEF** | 509.28 | 537.27 |
| **LE** | 215.14 | 243.13 | **LEF** | 362.21 | 390.20 | **EF** | 249.12 | 277.12 |

8. Tb10.6k15.1830

Match to: **Tb10.6k15.1830** Score: **94**

**centrin, putative; Trypanosoma bruceichr 10Manual**

Nominal mass (M_r_): **20662**; Calculated pI value: **4.67**

NCBI BLAST search of [Tb10.6k15.1830](http://www.ncbi.nlm.nih.gov/blast/Blast.cgi?ALIGNMENTS=50&ALIGNMENT_VIEW=Pairwise&AUTO_FORMAT=Semiauto&CDD_SEARCH=on&CLIENT=web&COMPOSITION_BASED_STATISTICS=on&DATABASE=nr&DESCRIPTIONS=100&ENTREZ_QUERY=(none)&EXPECT=10&FILTER=L&FORMAT_BLOCK_ON_RESPAGE=None&FORMAT_OBJECT=Alignment&FORMAT_TYPE=HTML&GAPCOSTS=11+1&I_THRESH=0.001&LAYOUT=TwoWindows&MATRIX_NAME=BLOSUM62&NCBI_GI=on&PAGE=Proteins&PROGRAM=blastp&QUERY=MDGDDGSRMGMPRLPPELTDAQRADIKEVFSILDVDGTETITPNDLKVALRALGYEPHKDTIKRLVAEMDRSGVSSNLILPEFEAILRAKLFTDDKEEVMLTFPHFTQGKSDYITLEDLKRVTQELGEDIPEDVLKRMIEEADVLDHDNRVSKEEFVRMLLTPKK&SERVICE=plain&SET_DEFAULTS.x=9&SET_DEFAULTS.y=5&SHOW_OVERVIEW=on&WORD_SIZE=3&END_OF_HTTPGET=Yes) against nr

Unformatted [sequence string](../../../../D:%5CProteomic%20data%5C2010-1-8%5Ccgi%5Cgetseq.pl%3FTBA927_IPI+Tb10%2E6k15%2E1830+seq) for pasting into other applications

Fixed modifications: MMTS (C),(N-TERM)_iTRAQ,Lysine(K)_iTRAQ

Variable modifications: Oxidation (M)

Cleavage by Trypsin: cuts C-term side of KR unless next residue is P

Sequence Coverage: **10%**

Matched peptides shown in **Bold Red**

**1** MDGDDGSRMG MPRLPPELTD AQRADIKEVF SILDVDGTET ITPNDLKVAL

**51** RALGYEPHKD TIKRLVAEMD R**SGVSSNLIL PEFEAILR**AK LFTDDKEEVM

**101** LTFPHFTQGK SDYITLEDLK RVTQELGEDI PEDVLKRMIE EADVLDHDNR

**151** VSKEEFVRML LTPKK

MS/MS Fragmentation of **SGVSSNLILPEFEAILR**
Found in **Tb10.6k15.1830**, centrin, putative; Trypanosoma bruceichr 10Manual


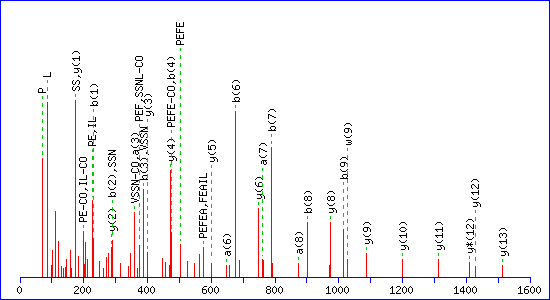


**MONOISOTOPIC mass of neutral peptide Mr(calc):** 1988.11

**Fixed modifications:** MMTS (C),(N-TERM)_iTRAQ,Lysine(K)_iTRAQ

**Ions Score:** 94 **Expect:** 2.9e-008

**Matches (Bold Red):** 50/297 fragment ions using 36 most intense peaks

| **#** | **Immon.** | **a** | **a*** | **a^0^** | **b** | **b*** | **b^0^** | **Seq.** | **v** | **w** | **w'** | **y** | **y*** | **y^0^** | **#** |
| --- | --- | --- | --- | --- | --- | --- | --- | --- | --- | --- | --- | --- | --- | --- | --- |
| **1** | 60.04 | 204.15 |  | 186.14 | **232.14** |  | 214.13 | **S** |  |  |  |  |  |  | **17** |
| **2** | 30.03 | 261.17 |  | 243.16 | **289.16** |  | 271.15 | **G** |  |  |  | 1757.98 | 1740.95 | 1739.97 | **16** |
| **3** | 72.08 | **360.24** |  | 342.23 | **388.23** |  | 370.22 | **V** | 1656.90 | 1669.92 |  | 1700.96 | 1683.93 | 1682.95 | **15** |
| **4** | 60.04 | 447.27 |  | 429.26 | **475.26** |  | 457.25 | **S** | 1569.86 | 1568.87 |  | 1601.89 | 1584.86 | 1583.88 | **14** |
| **5** | 60.04 | 534.30 |  | 516.29 | 562.30 |  | 544.29 | **S** | 1482.83 | 1481.84 |  | **1514.86** | 1497.83 | 1496.85 | **13** |
| **6** | 87.06 | **648.34** | 631.32 | 630.33 | **676.34** | 659.31 | 658.33 | **N** | 1368.79 | 1367.79 |  | **1427.83** | **1410.80** | 1409.82 | **12** |
| **7** | **86.10** | **761.43** | 744.40 | 743.42 | **789.42** | 772.40 | 771.41 | **L** | 1255.70 | 1254.71 |  | **1313.78** | 1296.76 | 1295.77 | **11** |
| **8** | **86.10** | **874.51** | 857.49 | 856.50 | **902.51** | 885.48 | 884.50 | **I** | 1142.62 | 1155.64 | 1169.66 | **1200.70** | 1183.67 | 1182.69 | **10** |
| **9** | **86.10** | 987.60 | 970.57 | 969.59 | **1015.59** | 998.56 | 997.58 | **L** | 1029.54 | **1028.54** |  | **1087.61** | 1070.59 | 1069.60 | **9** |
| **10** | **70.07** | 1084.65 | 1067.62 | 1066.64 | 1112.64 | 1095.62 | 1094.63 | **P** | 932.48 | 931.49 |  | **974.53** | 957.50 | 956.52 | **8** |
| **11** | 102.05 | 1213.69 | 1196.66 | 1195.68 | 1241.69 | 1224.66 | 1223.68 | **E** | 803.44 | 802.45 |  | 877.48 | 860.45 | 859.47 | **7** |
| **12** | 120.08 | 1360.76 | 1343.73 | 1342.75 | 1388.75 | 1371.73 | 1370.74 | **F** | 656.37 |  |  | **748.44** | 731.41 | 730.42 | **6** |
| **13** | 102.05 | 1489.80 | 1472.78 | 1471.79 | 1517.80 | 1500.77 | 1499.79 | **E** | 527.33 | 526.33 |  | **601.37** | 584.34 | 583.36 | **5** |
| **14** | 44.05 | 1560.84 | 1543.81 | 1542.83 | 1588.83 | 1571.81 | 1570.82 | **A** | 456.29 |  |  | **472.32** | 455.30 |  | **4** |
| **15** | **86.10** | 1673.92 | 1656.90 | 1655.91 | 1701.92 | 1684.89 | 1683.91 | **I** | 343.21 | 356.23 | 370.24 | **401.29** | 384.26 |  | **3** |
| **16** | **86.10** | 1787.01 | 1769.98 | 1769.00 | 1815.00 | 1797.98 | 1796.99 | **L** | 230.12 | 229.13 |  | **288.20** | 271.18 |  | **2** |
| **17** | 129.11 |  |  |  |  |  |  | **R** | 74.02 | 73.03 |  | **175.12** | 158.09 |  | **1** |

| **Seq** | **ya** | **yb** | **Seq** | **ya** | **yb** | **Seq** | **ya** | **yb** |
| --- | --- | --- | --- | --- | --- | --- | --- | --- |
| **GV** | 129.10 | 157.10 | **GVS** | 216.13 | 244.13 | **GVSS** | 303.17 | 331.16 |
| **GVSSN** | 417.21 | 445.20 | **GVSSNL** | 530.29 | 558.29 | **GVSSNLI** | 643.38 | 671.37 |
| **VS** | 159.11 | 187.11 | **VSS** | 246.14 | 274.14 | **VSSN** | **360.19** | **388.18** |
| **VSSNL** | 473.27 | 501.27 | **VSSNLI** | 586.36 | 614.35 | **VSSNLIL** | 699.44 | 727.43 |
| **SS** | 147.08 | **175.07** | **SSN** | 261.12 | **289.11** | **SSNL** | **374.20** | 402.20 |
| **SSNLI** | 487.29 | 515.28 | **SSNLIL** | 600.37 | 628.37 | **SSNLILP** | 697.42 | 725.42 |
| **SN** | 174.09 | 202.08 | **SNL** | 287.17 | 315.17 | **SNLI** | 400.26 | 428.25 |
| **SNLIL** | 513.34 | 541.33 | **SNLILP** | 610.39 | 638.39 | **NL** | 200.14 | 228.13 |
| **NLI** | 313.22 | 341.22 | **NLIL** | 426.31 | 454.30 | **NLILP** | 523.36 | 551.36 |
| **NLILPE** | 652.40 | 680.40 | **LI** | **199.18** | **227.18** | **LIL** | 312.26 | 340.26 |
| **LILP** | 409.32 | 437.31 | **LILPE** | 538.36 | 566.35 | **LILPEF** | 685.43 | 713.42 |
| **IL** | **199.18** | **227.18** | **ILP** | 296.23 | 324.23 | **ILPE** | 425.28 | 453.27 |
| **ILPEF** | 572.34 | 600.34 | **LP** | 183.15 | 211.14 | **LPE** | 312.19 | 340.19 |
| **LPEF** | 459.26 | 487.26 | **LPEFE** | 588.30 | 616.30 | **LPEFEA** | 659.34 | 687.33 |
| **PE** | **199.11** | **227.10** | **PEF** | 346.18 | **374.17** | **PEFE** | **475.22** | **503.21** |
| **PEFEA** | 546.26 | **574.25** | **PEFEAI** | 659.34 | 687.33 | **EF** | 249.12 | 277.12 |
| **EFE** | 378.17 | 406.16 | **EFEA** | 449.20 | 477.20 | **EFEAI** | 562.29 | 590.28 |
| **EFEAIL** | 675.37 | 703.37 | **FE** | 249.12 | 277.12 | **FEA** | 320.16 | 348.16 |
| **FEAI** | 433.24 | 461.24 | **FEAIL** | 546.33 | **574.32** | **EA** | 173.09 | 201.09 |
| **EAI** | 286.18 | 314.17 | **EAIL** | 399.26 | 427.26 | **AI** | 157.13 | 185.13 |
| **AIL** | 270.22 | 298.21 | **IL** | **199.18** | **227.18** |  |  |  |

9. Tb09.211.3955

Match to: **Tb09.211.3955** Score: **88**

**hypothetical protein, conserved; Trypanosoma bruceichr 9Manual**

Nominal mass (M_r_): **10396**; Calculated pI value: **4.65**

NCBI BLAST search of [Tb09.211.3955](http://www.ncbi.nlm.nih.gov/blast/Blast.cgi?ALIGNMENTS=50&ALIGNMENT_VIEW=Pairwise&AUTO_FORMAT=Semiauto&CDD_SEARCH=on&CLIENT=web&COMPOSITION_BASED_STATISTICS=on&DATABASE=nr&DESCRIPTIONS=100&ENTREZ_QUERY=(none)&EXPECT=10&FILTER=L&FORMAT_BLOCK_ON_RESPAGE=None&FORMAT_OBJECT=Alignment&FORMAT_TYPE=HTML&GAPCOSTS=11+1&I_THRESH=0.001&LAYOUT=TwoWindows&MATRIX_NAME=BLOSUM62&NCBI_GI=on&PAGE=Proteins&PROGRAM=blastp&QUERY=MDYEEPGQATRELTTFVQGLLQNMQTRFQEMSDTIITRIDEMGTRIDDLEKNIAELMQQAGPDEEAAQTASAPKDKKKSKGK&SERVICE=plain&SET_DEFAULTS.x=9&SET_DEFAULTS.y=5&SHOW_OVERVIEW=on&WORD_SIZE=3&END_OF_HTTPGET=Yes) against nr

Unformatted [sequence string](../../../../D:%5CProteomic%20data%5C2010-1-8%5Ccgi%5Cgetseq.pl%3FTBA927_IPI+Tb09%2E211%2E3955+seq) for pasting into other applications

Fixed modifications: MMTS (C),(N-TERM)_iTRAQ,Lysine(K)_iTRAQ

Variable modifications: Oxidation (M)

Cleavage by Trypsin: cuts C-term side of KR unless next residue is P

Sequence Coverage: **19%**

Matched peptides shown in **Bold Red**

**1** MDYEEPGQAT R**ELTTFVQGL LQNMQTR**FQE MSDTIITRID EMGTRIDDLE

**51** KNIAELMQQA GPDEEAAQTA SAPKDKKKSK GK

MS/MS Fragmentation of **ELTTFVQGLLQNMQTR**
Found in **Tb09.211.3955**, hypothetical protein, conserved; Trypanosoma bruceichr 9Manual


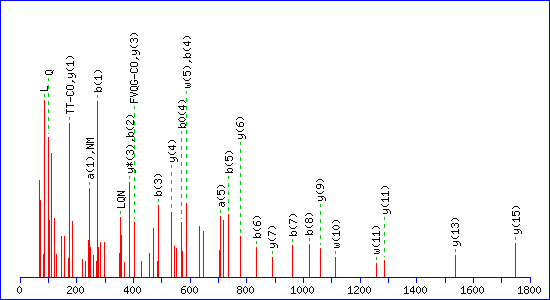


**MONOISOTOPIC mass of neutral peptide Mr(calc):** 2022.07

**Fixed modifications:** MMTS (C),(N-TERM)_iTRAQ,Lysine(K)_iTRAQ

**Ions Score:** 88 **Expect:** 1.7e-007

**Matches (Bold Red):** 34/275 fragment ions using 25 most intense peaks

| **#** | **Immon.** | **a** | **a*** | **a^0^** | **b** | **b*** | **b^0^** | **Seq.** | **v** | **w** | **w'** | **y** | **y*** | **y^0^** | **#** |
| --- | --- | --- | --- | --- | --- | --- | --- | --- | --- | --- | --- | --- | --- | --- | --- |
| **1** | 102.05 | **246.16** |  | 228.15 | **274.15** |  | 256.14 | **E** |  |  |  |  |  |  | **16** |
| **2** | **86.10** | 359.24 |  | 341.23 | **387.24** |  | 369.23 | **L** | 1691.85 | 1690.86 |  | **1749.93** | 1732.91 | 1731.92 | **15** |
| **3** | 74.06 | 460.29 |  | 442.28 | **488.28** |  | 470.27 | **T** | 1590.81 | 1603.83 | 1605.81 | 1636.85 | 1619.82 | 1618.84 | **14** |
| **4** | 74.06 | 561.34 |  | 543.33 | **589.33** |  | **571.32** | **T** | 1489.76 | 1502.78 | 1504.76 | **1535.80** | 1518.77 | 1517.79 | **13** |
| **5** | 120.08 | **708.41** |  | 690.39 | **736.40** |  | 718.39 | **F** | 1342.69 |  |  | 1434.75 | 1417.73 | 1416.74 | **12** |
| **6** | 72.08 | 807.47 |  | 789.46 | **835.47** |  | 817.46 | **V** | 1243.62 | **1256.64** |  | **1287.68** | 1270.66 | 1269.67 | **11** |
| **7** | **101.07** | 935.53 | 918.51 | 917.52 | **963.53** | 946.50 | 945.52 | **Q** | 1115.56 | **1114.57** |  | 1188.62 | 1171.59 | 1170.60 | **10** |
| **8** | 30.03 | 992.55 | 975.53 | 974.54 | **1020.55** | 1003.52 | 1002.54 | **G** |  |  |  | **1060.56** | 1043.53 | 1042.55 | **9** |
| **9** | **86.10** | 1105.64 | 1088.61 | 1087.63 | 1133.63 | 1116.61 | 1115.62 | **L** | 945.46 | 944.46 |  | 1003.54 | 986.51 | 985.52 | **8** |
| **10** | **86.10** | 1218.72 | 1201.70 | 1200.71 | 1246.72 | 1229.69 | 1228.71 | **L** | 832.37 | 831.38 |  | **890.45** | 873.42 | 872.44 | **7** |
| **11** | **101.07** | 1346.78 | 1329.75 | 1328.77 | 1374.78 | 1357.75 | 1356.76 | **Q** | 704.31 | 703.32 |  | **777.37** | 760.34 | 759.36 | **6** |
| **12** | 87.06 | 1460.82 | 1443.80 | 1442.81 | 1488.82 | 1471.79 | 1470.81 | **N** | 590.27 | **589.28** |  | 649.31 | 632.28 | 631.30 | **5** |
| **13** | 104.05 | 1591.86 | 1574.84 | 1573.85 | 1619.86 | 1602.83 | 1601.85 | **M** | 459.23 | 458.24 |  | **535.27** | 518.24 | 517.26 | **4** |
| **14** | **101.07** | 1719.92 | 1702.90 | 1701.91 | 1747.92 | 1730.89 | 1729.91 | **Q** | 331.17 | 330.18 |  | **404.23** | **387.20** | 386.21 | **3** |
| **15** | 74.06 | 1820.97 | 1803.94 | 1802.96 | 1848.96 | 1831.94 | 1830.95 | **T** | 230.12 | 243.15 | 245.12 | 276.17 | 259.14 | 258.16 | **2** |
| **16** | 129.11 |  |  |  |  |  |  | **R** | 74.02 | 73.03 |  | **175.12** | 158.09 |  | **1** |

| **Seq** | **ya** | **yb** | **Seq** | **ya** | **yb** | **Seq** | **ya** | **yb** |
| --- | --- | --- | --- | --- | --- | --- | --- | --- |
| **LT** | 187.14 | 215.14 | **LTT** | 288.19 | 316.19 | **LTTF** | 435.26 | 463.26 |
| **LTTFV** | 534.33 | 562.32 | **LTTFVQ** | 662.39 | 690.38 | **TT** | **175.11** | 203.10 |
| **TTF** | 322.18 | 350.17 | **TTFV** | 421.24 | 449.24 | **TTFVQ** | 549.30 | 577.30 |
| **TTFVQG** | 606.32 | 634.32 | **TF** | 221.13 | 249.12 | **TFV** | 320.20 | 348.19 |
| **TFVQ** | 448.26 | 476.25 | **TFVQG** | 505.28 | 533.27 | **TFVQGL** | 618.36 | 646.36 |
| **FV** | 219.15 | 247.14 | **FVQ** | 347.21 | 375.20 | **FVQG** | **404.23** | 432.22 |
| **FVQGL** | 517.31 | 545.31 | **FVQGLL** | 630.40 | 658.39 | **VQ** | 200.14 | 228.13 |
| **VQG** | 257.16 | 285.16 | **VQGL** | 370.24 | 398.24 | **VQGLL** | 483.33 | 511.32 |
| **VQGLLQ** | 611.39 | 639.38 | **QG** | 158.09 | 186.09 | **QGL** | 271.18 | 299.17 |
| **QGLL** | 384.26 | 412.26 | **QGLLQ** | 512.32 | 540.31 | **QGLLQN** | 626.36 | 654.36 |
| **GL** | 143.12 | 171.11 | **GLL** | 256.20 | 284.20 | **GLLQ** | 384.26 | 412.26 |
| **GLLQN** | 498.30 | 526.30 | **GLLQNM** | 629.34 | 657.34 | **LL** | 199.18 | 227.18 |
| **LLQ** | 327.24 | 355.23 | **LLQN** | 441.28 | 469.28 | **LLQNM** | 572.32 | 600.32 |
| **LQ** | 214.16 | 242.15 | **LQN** | 328.20 | **356.19** | **LQNM** | 459.24 | 487.23 |
| **LQNMQ** | 587.30 | 615.29 | **LQNMQT** | 688.34 | 716.34 | **QN** | 215.11 | 243.11 |
| **QNM** | 346.15 | 374.15 | **QNMQ** | 474.21 | 502.21 | **QNMQT** | 575.26 | 603.26 |
| **NM** | 218.10 | **246.09** | **NMQ** | 346.15 | 374.15 | **NMQT** | 447.20 | 475.20 |
| **MQ** | 232.11 | 260.11 | **MQT** | 333.16 | 361.15 | **QT** | 202.12 | 230.11 |

10. Tb11.01.7750

Match to: **Tb11.01.7750** Score: **87**

**dynein docking complex 2 (ODA1) protein, putative; Trypanosoma bruceichr 11Manual**

Nominal mass (M_r_): **78570**; Calculated pI value: **5.41**

NCBI BLAST search of [Tb11.01.7750](http://www.ncbi.nlm.nih.gov/blast/Blast.cgi?ALIGNMENTS=50&ALIGNMENT_VIEW=Pairwise&AUTO_FORMAT=Semiauto&CDD_SEARCH=on&CLIENT=web&COMPOSITION_BASED_STATISTICS=on&DATABASE=nr&DESCRIPTIONS=100&ENTREZ_QUERY=(none)&EXPECT=10&FILTER=L&FORMAT_BLOCK_ON_RESPAGE=None&FORMAT_OBJECT=Alignment&FORMAT_TYPE=HTML&GAPCOSTS=11+1&I_THRESH=0.001&LAYOUT=TwoWindows&MATRIX_NAME=BLOSUM62&NCBI_GI=on&PAGE=Proteins&PROGRAM=blastp&QUERY=MPPSQLLKDMDERRCLMASNIVAAQDEIMRQQEAVQKLTSENERLKKEIAVASGEQYDYVKADKYAALKTEVDSLEQRYQFEKMHLNELTKQYQLARIDLMQGSKLKGGVNAEQENVRAVQRQLEILENRLDQALARFNDAVSYNKELRDHIDIIRGERRVFQRVHKKMEDDLRSKKKIMSERIEQSNHDLDERDGYLQQVEQLRTALSEQKEEYDTAVRNLDVCMIDINRMRDELHRRQIEFEARSYMPNPTVDHSGVGKNLPVANTHAPTPFTEDGEDASDGMSSAEVGSEIMDIPAQLSMFAPDGDLQKLAQTYQVVGESNFSLYKRINELTTSREEMERDIHTLKKVIAEEHEHDVQQRRLIKEYEDRLAETELMLDRLNRSAEVHKEVLARIRETTEGVFKRIGCSAEEARRLVGSDHCTESTQLKFLGLIEERATRILCTYQLYKRTEAMLQEQKAAEEGADGSKRTLPIGAGDDQTYLPGKEKETDGDRGGNAAAAGGAENTRGIVSAVKEEGGEGSSGSKVDVRLLQALVEGTADFSPVLNLPVRKDGTAAKFVRCSVLPSAQLYGDESVEGKDDHDGDPVVSHEELRQQMQQRLLSKRLREEKGQRRKRDLKDQFADAPPILRRK&SERVICE=plain&SET_DEFAULTS.x=9&SET_DEFAULTS.y=5&SHOW_OVERVIEW=on&WORD_SIZE=3&END_OF_HTTPGET=Yes) against nr

Unformatted [sequence string](../../../../D:%5CProteomic%20data%5C2010-1-8%5Ccgi%5Cgetseq.pl%3FTBA927_IPI+Tb11%2E01%2E7750+seq) for pasting into other applications

Fixed modifications: MMTS (C),(N-TERM)_iTRAQ,Lysine(K)_iTRAQ

Variable modifications: Oxidation (M)

Cleavage by Trypsin: cuts C-term side of KR unless next residue is P

Sequence Coverage: **5%**

Matched peptides shown in **Bold Red**

**1** MPPSQLLKDM DERRCLMASN IVAAQDEIMR QQEAVQKLTS ENERLKKEIA

**51** VASGEQYDYV KADKYAALKT EVDSLEQRYQ FEKMHLNELT KQYQLARIDL

**101** MQGSKLKGGV NAEQENVRAV QRQLEILENR **LDQALAR**FND AVSYNKELRD

**151** HIDIIRGERR VFQRVHKKME DDLRSKKKIM SERIEQSNHD LDERDGYLQQ

**201** VEQLR**TALSE QKEEYDTAVR** NLDVCMIDIN RMRDELHRRQ IEFEARSYMP

**251** NPTVDHSGVG KNLPVANTHA PTPFTEDGED ASDGMSSAEV GSEIMDIPAQ

**301** LSMFAPDGDL QKLAQTYQVV GESNFSLYKR INELTTSREE MERDIHTLKK

**351** VIAEEHEHDV QQRRLIKEYE DRLAETELML DRLNRSAEVH KEVLARIRET

**401** TEGVFKRIGC SAEEARRLVG SDHCTESTQL KFLGLIEERA TRILCTYQLY

**451** KRTEAMLQEQ KAAEEGADGS KRTLPIGAGD DQTYLPGKEK ETDGDRGGNA

**501** AAAGGAENTR GIVSAVKEEG GEGSSGSKVD VRLLQALVEG TADFSPVLNL

**551** PVRKDGTAAK FVRCSVLPSA QLYGDESVEG KDDHDGDPVV SHEELRQQMQ

**601** QRLLSKRLRE EKGQRRKR**DL KDQFADAPPI LR**RK

**Start - End Observed Mr(expt) Mr(calc) Delta Miss Sequence**

**131 - 137 930.56 929.55 929.54 0.01 0 R.LDQALAR.F**  ([Ions score 25](../../../../D:%5CProteomic%20data%5C2010-1-8%5CZQ%5C988.htm))

**206 - 220 2028.05 2027.04 2026.95 0.09 1 R.TALSEQKEEYDTAVR.N**  ([Ions score 87](../../../../D:%5CProteomic%20data%5C2010-1-8%5CZQ%5C990.htm))

**619 - 632 1886.99 1885.98 1885.96 0.02 1 R.DLKDQFADAPPILR.R**  ([Ions score 18](../../../../D:%5CProteomic%20data%5C2010-1-8%5CZQ%5C989.htm))

MS/MS Fragmentation of **TALSEQKEEYDTAVR**
Found in **Tb11.01.7750**, dynein docking complex 2 (ODA1) protein, putative; Trypanosoma bruceichr 11Manual


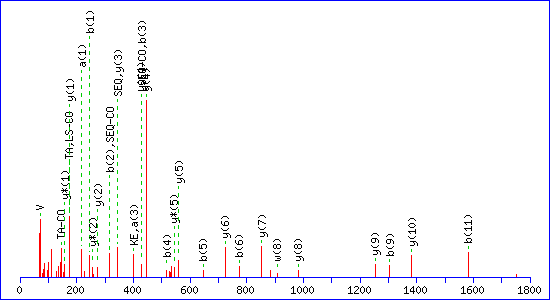


**MONOISOTOPIC mass of neutral peptide Mr(calc):** 2026.95

**Fixed modifications:** MMTS (C),(N-TERM)_iTRAQ,Lysine(K)_iTRAQ

**Ions Score:** 87 **Expect:** 2.1e-007

**Matches (Bold Red):** 33/231 fragment ions using 33 most intense peaks

| **#** | **Immon.** | **a** | **a*** | **a^0^** | **b** | **b*** | **b^0^** | **Seq.** | **v** | **w** | **w'** | **y** | **y*** | **y^0^** | **#** |
| --- | --- | --- | --- | --- | --- | --- | --- | --- | --- | --- | --- | --- | --- | --- | --- |
| **1** | 74.06 | **218.16** |  | 200.15 | **246.16** |  | 228.15 | **T** |  |  |  |  |  |  | **15** |
| **2** | 44.05 | 289.20 |  | 271.19 | **317.19** |  | 299.18 | **A** | 1766.78 |  |  | 1782.81 | 1765.78 | 1764.80 | **14** |
| **3** | 86.10 | **402.28** |  | 384.27 | **430.28** |  | 412.27 | **L** | 1653.69 | 1652.70 |  | 1711.77 | 1694.75 | 1693.76 | **13** |
| **4** | 60.04 | 489.32 |  | 471.31 | **517.31** |  | 499.30 | **S** | 1566.66 | 1565.67 |  | 1598.69 | 1581.66 | 1580.68 | **12** |
| **5** | 102.05 | 618.36 |  | 600.35 | **646.35** |  | 628.34 | **E** | 1437.62 | 1436.62 |  | 1511.66 | 1494.63 | 1493.65 | **11** |
| **6** | 101.07 | 746.42 | 729.39 | 728.41 | **774.41** | 757.39 | 756.40 | **Q** | 1309.56 | 1308.57 |  | **1382.61** | 1365.59 | 1364.60 | **10** |
| **7** | 245.12 | 1018.52 | 1001.50 | 1000.51 | 1046.52 | 1029.49 | 1028.51 | **K** | 1037.45 | 1036.46 |  | **1254.56** | 1237.53 | 1236.54 | **9** |
| **8** | 102.05 | 1147.57 | 1130.54 | 1129.56 | 1175.56 | 1158.54 | 1157.55 | **E** | 908.41 | **907.42** |  | **982.45** | 965.42 | 964.44 | **8** |
| **9** | 102.05 | 1276.61 | 1259.58 | 1258.60 | **1304.60** | 1287.58 | 1286.59 | **E** | 779.37 | 778.37 |  | **853.41** | 836.38 | 835.39 | **7** |
| **10** | 136.08 | 1439.67 | 1422.65 | 1421.66 | 1467.67 | 1450.64 | 1449.66 | **Y** | 616.30 |  |  | **724.36** | 707.34 | 706.35 | **6** |
| **11** | 88.04 | 1554.70 | 1537.67 | 1536.69 | **1582.69** | 1565.67 | 1564.68 | **D** | 501.28 | 500.28 |  | **561.30** | **544.27** | 543.29 | **5** |
| **12** | 74.06 | 1655.75 | 1638.72 | 1637.74 | 1683.74 | 1666.72 | 1665.73 | **T** | 400.23 | 413.25 | 415.23 | **446.27** | 429.25 | **428.26** | **4** |
| **13** | 44.05 | 1726.78 | 1709.76 | 1708.77 | 1754.78 | 1737.75 | 1736.77 | **A** | 329.19 |  |  | **345.22** | 328.20 |  | **3** |
| **14** | **72.08** | 1825.85 | 1808.83 | 1807.84 | 1853.85 | 1836.82 | 1835.84 | **V** | 230.12 | 243.15 |  | **274.19** | **257.16** |  | **2** |
| **15** | 129.11 |  |  |  |  |  |  | **R** | 74.02 | 73.03 |  | **175.12** | **158.09** |  | **1** |

| **Seq** | **ya** | **yb** | **Seq** | **ya** | **yb** | **Seq** | **ya** | **yb** |
| --- | --- | --- | --- | --- | --- | --- | --- | --- |
| **AL** | 157.13 | 185.13 | **ALS** | 244.17 | 272.16 | **ALSE** | 373.21 | 401.20 |
| **ALSEQ** | 501.27 | 529.26 | **LS** | **173.13** | 201.12 | **LSE** | 302.17 | 330.17 |
| **LSEQ** | **430.23** | 458.22 | **SE** | 189.09 | 217.08 | **SEQ** | **317.15** | **345.14** |
| **SEQK** | 589.25 | 617.25 | **EQ** | 230.11 | 258.11 | **EQK** | 502.22 | 530.22 |
| **EQKE** | 631.26 | 659.26 | **QK** | 373.18 | 401.17 | **QKE** | 502.22 | 530.22 |
| **QKEE** | 631.26 | 659.26 | **KE** | 374.16 | **402.16** | **KEE** | 503.20 | 531.20 |
| **KEEY** | 666.27 | 694.26 | **EE** | 231.10 | 259.09 | **EEY** | 394.16 | 422.16 |
| **EEYD** | 509.19 | 537.18 | **EEYDT** | 610.24 | 638.23 | **EEYDTA** | 681.27 | 709.27 |
| **EY** | 265.12 | 293.11 | **EYD** | 380.15 | 408.14 | **EYDT** | 481.19 | 509.19 |
| **EYDTA** | 552.23 | 580.22 | **EYDTAV** | 651.30 | 679.29 | **YD** | 251.10 | 279.10 |
| **YDT** | 352.15 | 380.15 | **YDTA** | 423.19 | 451.18 | **YDTAV** | 522.26 | 550.25 |
| **DT** | 189.09 | 217.08 | **DTA** | 260.12 | 288.12 | **DTAV** | 359.19 | 387.19 |
| **TA** | **145.10** | **173.09** | **TAV** | 244.17 | 272.16 | **AV** | 143.12 | 171.11 |

11.Tb927.5.1880

Match to: **Tb927.5.1880** Score: **85**

**inhibitor of serine peptidase (ISP), putative; Trypanosoma bruceichr 5Manual**

Nominal mass (M_r_): **18755**; Calculated pI value: **5.28**

NCBI BLAST search of [Tb927.5.1880](http://www.ncbi.nlm.nih.gov/blast/Blast.cgi?ALIGNMENTS=50&ALIGNMENT_VIEW=Pairwise&AUTO_FORMAT=Semiauto&CDD_SEARCH=on&CLIENT=web&COMPOSITION_BASED_STATISTICS=on&DATABASE=nr&DESCRIPTIONS=100&ENTREZ_QUERY=(none)&EXPECT=10&FILTER=L&FORMAT_BLOCK_ON_RESPAGE=None&FORMAT_OBJECT=Alignment&FORMAT_TYPE=HTML&GAPCOSTS=11+1&I_THRESH=0.001&LAYOUT=TwoWindows&MATRIX_NAME=BLOSUM62&NCBI_GI=on&PAGE=Proteins&PROGRAM=blastp&QUERY=MTDRPPTLADFKAPYPEPGPDQTCCIILLEEKPDEEENYRVELIPGRVMEDGLATGTVSGVVREEVIHGWGYSYYVVEMEPLVTTRRSIRSFHRPTRFVPVPTKHFIRYNSQLPVVVYLPHNTELRYRVWTPIDMQKVEPTEPEGLLKIEERAG&SERVICE=plain&SET_DEFAULTS.x=9&SET_DEFAULTS.y=5&SHOW_OVERVIEW=on&WORD_SIZE=3&END_OF_HTTPGET=Yes) against nr

Unformatted [sequence string](../../../../D:%5CProteomic%20data%5C2010-1-8%5Ccgi%5Cgetseq.pl%3FTBA927_IPI+Tb927%2E5%2E1880+seq) for pasting into other applications

Fixed modifications: MMTS (C),(N-TERM)_iTRAQ,Lysine(K)_iTRAQ

Variable modifications: Oxidation (M)

Cleavage by Trypsin: cuts C-term side of KR unless next residue is P

Sequence Coverage: **10%**

Matched peptides shown in **Bold Red**

**1** MTDRPPTLAD FKAPYPEPGP DQTCCIILLE EKPDEEENYR VELIPGR**VME**

**51 DGLATGTVSG VVR**EEVIHGW GYSYYVVEME PLVTTRRSIR SFHRPTRFVP

**101** VPTKHFIRYN SQLPVVVYLP HNTELRYRVW TPIDMQKVEP TEPEGLLKIE

**151** ERAG

MS/MS Fragmentation of **VMEDGLATGTVSGVVR**
Found in **Tb927.5.1880**, inhibitor of serine peptidase (ISP), putative; Trypanosoma bruceichr 5Manual


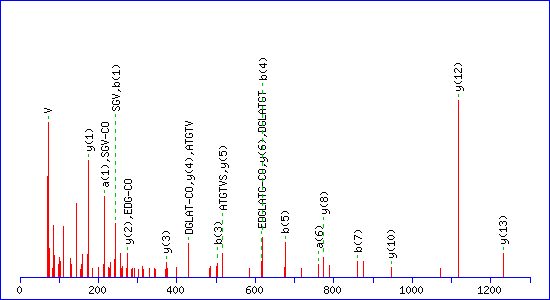


**MONOISOTOPIC mass of neutral peptide Mr(calc):** 1733.91

**Fixed modifications:** MMTS (C),(N-TERM)_iTRAQ,Lysine(K)_iTRAQ

**Ions Score:** 85 **Expect:** 3.8e-007

**Matches (Bold Red):** 31/271 fragment ions using 22 most intense peaks

| **#** | **Immon.** | **a** | **a^0^** | **b** | **b^0^** | **Seq.** | **v** | **w** | **w'** | **y** | **y*** | **y^0^** | **#** |
| --- | --- | --- | --- | --- | --- | --- | --- | --- | --- | --- | --- | --- | --- |
| **1** | **72.08** | **216.18** |  | **244.18** |  | **V** |  |  |  |  |  |  | **16** |
| **2** | 104.05 | 347.22 |  | 375.22 |  | **M** | 1415.71 | 1414.72 |  | 1491.75 | 1474.72 | 1473.74 | **15** |
| **3** | 102.05 | 476.27 | 458.26 | **504.26** | 486.25 | **E** | 1286.67 | 1285.67 |  | 1360.71 | 1343.68 | 1342.70 | **14** |
| **4** | 88.04 | 591.29 | 573.28 | **619.29** | 601.28 | **D** | 1171.64 | 1170.65 |  | **1231.66** | 1214.64 | 1213.65 | **13** |
| **5** | 30.03 | 648.31 | 630.30 | **676.31** | 658.30 | **G** |  |  |  | **1116.64** | 1099.61 | 1098.63 | **12** |
| **6** | 86.10 | **761.40** | 743.39 | 789.39 | 771.38 | **L** | 1001.54 | 1000.54 |  | 1059.62 | 1042.59 | 1041.61 | **11** |
| **7** | 44.05 | 832.44 | 814.43 | **860.43** | 842.42 | **A** | 930.50 |  |  | **946.53** | 929.51 | 928.52 | **10** |
| **8** | 74.06 | 933.48 | 915.47 | 961.48 | 943.47 | **T** | 829.45 | 842.47 | 844.45 | 875.49 | 858.47 | 857.48 | **9** |
| **9** | 30.03 | 990.50 | 972.49 | 1018.50 | 1000.49 | **G** |  |  |  | **774.45** | 757.42 | 756.44 | **8** |
| **10** | 74.06 | 1091.55 | 1073.54 | 1119.55 | 1101.54 | **T** | 671.38 | 684.40 | 686.38 | 717.43 | 700.40 | 699.41 | **7** |
| **11** | **72.08** | 1190.62 | 1172.61 | 1218.62 | 1200.61 | **V** | 572.32 | 585.34 |  | **616.38** | 599.35 | 598.37 | **6** |
| **12** | 60.04 | 1277.65 | 1259.64 | 1305.65 | 1287.64 | **S** | 485.28 | 484.29 |  | **517.31** | 500.28 | 499.30 | **5** |
| **13** | 30.03 | 1334.67 | 1316.66 | 1362.67 | 1344.66 | **G** |  |  |  | **430.28** | 413.25 |  | **4** |
| **14** | **72.08** | 1433.74 | 1415.73 | 1461.74 | 1443.73 | **V** | 329.19 | 342.21 |  | **373.26** | 356.23 |  | **3** |
| **15** | **72.08** | 1532.81 | 1514.80 | 1560.81 | 1542.80 | **V** | 230.12 | 243.15 |  | **274.19** | 257.16 |  | **2** |
| **16** | 129.11 |  |  |  |  | **R** | 74.02 | 73.03 |  | **175.12** | 158.09 |  | **1** |

| **Seq** | **ya** | **yb** | **Seq** | **ya** | **yb** | **Seq** | **ya** | **yb** |
| --- | --- | --- | --- | --- | --- | --- | --- | --- |
| **ME** | 233.10 | 261.09 | **MED** | 348.12 | 376.12 | **MEDG** | 405.14 | 433.14 |
| **MEDGL** | 518.23 | 546.22 | **MEDGLA** | 589.27 | 617.26 | **MEDGLAT** | 690.31 | 718.31 |
| **ED** | 217.08 | 245.08 | **EDG** | **274.10** | 302.10 | **EDGL** | 387.19 | 415.18 |
| **EDGLA** | 458.22 | 486.22 | **EDGLAT** | 559.27 | 587.27 | **EDGLATG** | **616.29** | 644.29 |
| **DG** | 145.06 | 173.06 | **DGL** | 258.14 | 286.14 | **DGLA** | 329.18 | 357.18 |
| **DGLAT** | **430.23** | 458.22 | **DGLATG** | 487.25 | 515.25 | **DGLATGT** | 588.30 | **616.29** |
| **DGLATGTV** | 687.37 | 715.36 | **GL** | 143.12 | 171.11 | **GLA** | 214.15 | 242.15 |
| **GLAT** | 315.20 | 343.20 | **GLATG** | 372.22 | 400.22 | **GLATGT** | 473.27 | 501.27 |
| **GLATGTV** | 572.34 | 600.34 | **GLATGTVS** | 659.37 | 687.37 | **LA** | 157.13 | 185.13 |
| **LAT** | 258.18 | 286.18 | **LATG** | 315.20 | 343.20 | **LATGT** | 416.25 | 444.25 |
| **LATGTV** | 515.32 | 543.31 | **LATGTVS** | 602.35 | 630.35 | **LATGTVSG** | 659.37 | 687.37 |
| **AT** | 145.10 | 173.09 | **ATG** | 202.12 | 230.11 | **ATGT** | 303.17 | 331.16 |
| **ATGTV** | 402.23 | **430.23** | **ATGTVS** | 489.27 | **517.26** | **ATGTVSG** | 546.29 | 574.28 |
| **ATGTVSGV** | 645.36 | 673.35 | **TG** | 131.08 | 159.08 | **TGT** | 232.13 | 260.12 |
| **TGTV** | 331.20 | 359.19 | **TGTVS** | 418.23 | 446.22 | **TGTVSG** | 475.25 | 503.25 |
| **TGTVSGV** | 574.32 | 602.31 | **TGTVSGVV** | 673.39 | 701.38 | **GT** | 131.08 | 159.08 |
| **GTV** | 230.15 | 258.14 | **GTVS** | 317.18 | 345.18 | **GTVSG** | 374.20 | 402.20 |
| **GTVSGV** | 473.27 | 501.27 | **GTVSGVV** | 572.34 | 600.34 | **TV** | 173.13 | 201.12 |
| **TVS** | 260.16 | 288.16 | **TVSG** | 317.18 | 345.18 | **TVSGV** | 416.25 | 444.25 |
| **TVSGVV** | 515.32 | 543.31 | **VS** | 159.11 | 187.11 | **VSG** | **216.13** | **244.13** |
| **VSGV** | 315.20 | 343.20 | **VSGVV** | 414.27 | 442.27 | **SG** | 117.07 | 145.06 |
| **SGV** | **216.13** | **244.13** | **SGVV** | 315.20 | 343.20 | **GV** | 129.10 | 157.10 |
| **GVV** | 228.17 | 256.17 | **VV** | 171.15 | 199.14 |  |  |  |

12. Tb10.406.0550

Match to: **Tb10.406.0550** Score: **85**

**hypothetical protein, conserved; Trypanosoma bruceichr 10Manual**

Nominal mass (M_r_): **22020**; Calculated pI value: **9.48**

NCBI BLAST search of [Tb10.406.0550](http://www.ncbi.nlm.nih.gov/blast/Blast.cgi?ALIGNMENTS=50&ALIGNMENT_VIEW=Pairwise&AUTO_FORMAT=Semiauto&CDD_SEARCH=on&CLIENT=web&COMPOSITION_BASED_STATISTICS=on&DATABASE=nr&DESCRIPTIONS=100&ENTREZ_QUERY=(none)&EXPECT=10&FILTER=L&FORMAT_BLOCK_ON_RESPAGE=None&FORMAT_OBJECT=Alignment&FORMAT_TYPE=HTML&GAPCOSTS=11+1&I_THRESH=0.001&LAYOUT=TwoWindows&MATRIX_NAME=BLOSUM62&NCBI_GI=on&PAGE=Proteins&PROGRAM=blastp&QUERY=MHTGDKRVLPCFDNHYNSGVLVDNWFDARINEAHGKFTIAVDGQSPLPSSVYKADYSTPCVTAVRPMLRQRGLGKSLIFGSGLPTVDEVVATSKDYGATQRYLIDSRYGKPKHHCKPLNTLSGVDRFALTGKLSKPVNQPTVDDAMVDRFRTTKSAMDVTILNHHVQRQLSAGRNCGARTQM&SERVICE=plain&SET_DEFAULTS.x=9&SET_DEFAULTS.y=5&SHOW_OVERVIEW=on&WORD_SIZE=3&END_OF_HTTPGET=Yes) against nr

Unformatted [sequence string](../../../../D:%5CProteomic%20data%5C2010-1-8%5Ccgi%5Cgetseq.pl%3FTBA927_IPI+Tb10%2E406%2E0550+seq) for pasting into other applications

Fixed modifications: MMTS (C),(N-TERM)_iTRAQ,Lysine(K)_iTRAQ

Variable modifications: Oxidation (M)

Cleavage by Trypsin: cuts C-term side of KR unless next residue is P

Sequence Coverage: **9%**

Matched peptides shown in **Bold Red**

**1** MHTGDKRVLP CFDNHYNSGV LVDNWFDARI NEAHGKFTIA VDGQSPLPSS

**51** VYKADYSTPC VTAVRPMLRQ RGLGKSLIFG SGLPTVDEVV ATSKDYGATQ

**101** RYLIDSRYGK PKHHCKPLNT LSGVDRFALT GK**LSKPVNQP TVDDAMVDR**F

**151** RTTKSAMDVT ILNHHVQRQL SAGRNCGART QM

MS/MS Fragmentation of **LSKPVNQPTVDDAMVDR**
Found in **Tb10.406.0550**, hypothetical protein, conserved; Trypanosoma bruceichr 10Manual


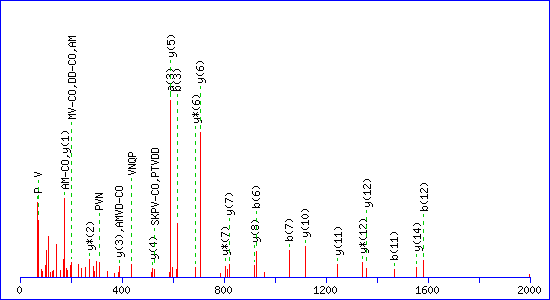


**MONOISOTOPIC mass of neutral peptide Mr(calc):** 2172.06

**Fixed modifications:** MMTS (C),(N-TERM)_iTRAQ,Lysine(K)_iTRAQ

**Ions Score:** 85 **Expect:** 2.9e-007

**Matches (Bold Red):** 37/302 fragment ions using 31 most intense peaks

| **#** | **Immon.** | **a** | **a*** | **a^0^** | **b** | **b*** | **b^0^** | **Seq.** | **v** | **w** | **w'** | **y** | **y*** | **y^0^** | **#** |
| --- | --- | --- | --- | --- | --- | --- | --- | --- | --- | --- | --- | --- | --- | --- | --- |
| **1** | 86.10 | 230.20 |  |  | 258.19 |  |  | **L** |  |  |  |  |  |  | **17** |
| **2** | 60.04 | 317.23 |  | 299.22 | 345.23 |  | 327.22 | **S** | 1883.85 | 1882.86 |  | 1915.88 | 1898.85 | 1897.87 | **16** |
| **3** | 245.12 | **589.34** | 572.31 | 571.33 | **617.33** | 600.31 | 599.32 | **K** | 1611.74 | 1610.75 |  | 1828.84 | 1811.82 | 1810.83 | **15** |
| **4** | **70.07** | 686.39 | 669.36 | 668.38 | 714.39 | 697.36 | 696.38 | **P** | 1514.69 | 1513.70 |  | **1556.74** | 1539.71 | 1538.73 | **14** |
| **5** | **72.08** | 785.46 | 768.43 | 767.45 | 813.45 | 796.43 | 795.44 | **V** | 1415.62 | 1428.64 |  | 1459.68 | 1442.66 | 1441.67 | **13** |
| **6** | 87.06 | 899.50 | 882.48 | 881.49 | **927.50** | 910.47 | 909.49 | **N** | 1301.58 | 1300.58 |  | **1360.62** | **1343.59** | 1342.61 | **12** |
| **7** | 101.07 | 1027.56 | 1010.53 | 1009.55 | **1055.56** | 1038.53 | 1037.55 | **Q** | 1173.52 | 1172.53 |  | **1246.57** | 1229.55 | 1228.56 | **11** |
| **8** | **70.07** | 1124.61 | 1107.59 | 1106.60 | 1152.61 | 1135.58 | 1134.60 | **P** | 1076.47 | 1075.47 |  | **1118.51** | 1101.49 | 1100.50 | **10** |
| **9** | 74.06 | 1225.66 | 1208.63 | 1207.65 | 1253.66 | 1236.63 | 1235.65 | **T** | 975.42 | 988.44 | 990.42 | 1021.46 | 1004.44 | 1003.45 | **9** |
| **10** | **72.08** | 1324.73 | 1307.70 | 1306.72 | 1352.72 | 1335.70 | 1334.71 | **V** | 876.35 | 889.37 |  | **920.41** | 903.39 | 902.40 | **8** |
| **11** | 88.04 | 1439.76 | 1422.73 | 1421.75 | **1467.75** | 1450.73 | 1449.74 | **D** | 761.32 | 760.33 |  | **821.35** | **804.32** | 803.34 | **7** |
| **12** | 88.04 | 1554.78 | 1537.76 | 1536.77 | **1582.78** | 1565.75 | 1564.77 | **D** | 646.30 | 645.30 |  | **706.32** | **689.29** | 688.31 | **6** |
| **13** | 44.05 | 1625.82 | 1608.79 | 1607.81 | 1653.82 | 1636.79 | 1635.81 | **A** | 575.26 |  |  | **591.29** | 574.27 | 573.28 | **5** |
| **14** | 104.05 | 1756.86 | 1739.83 | 1738.85 | 1784.86 | 1767.83 | 1766.85 | **M** | 444.22 | 443.22 |  | **520.25** | 503.23 | 502.24 | **4** |
| **15** | **72.08** | 1855.93 | 1838.90 | 1837.92 | 1883.92 | 1866.90 | 1865.91 | **V** | 345.15 | 358.17 |  | **389.21** | 372.19 | 371.20 | **3** |
| **16** | 88.04 | 1970.96 | 1953.93 | 1952.95 | 1998.95 | 1981.93 | 1980.94 | **D** | 230.12 | 229.13 |  | 290.15 | **273.12** | 272.14 | **2** |
| **17** | 129.11 |  |  |  |  |  |  | **R** | 74.02 | 73.03 |  | **175.12** | 158.09 |  | **1** |

| **Seq** | **ya** | **yb** | **Seq** | **ya** | **yb** | **Seq** | **ya** | **yb** |
| --- | --- | --- | --- | --- | --- | --- | --- | --- |
| **SK** | 332.15 | 360.15 | **SKP** | 429.20 | 457.20 | **SKPV** | **528.27** | 556.27 |
| **SKPVN** | 642.32 | 670.31 | **KP** | 342.17 | 370.17 | **KPV** | 441.24 | 469.24 |
| **KPVN** | 555.28 | 583.28 | **KPVNQ** | 683.34 | 711.34 | **PV** | 169.13 | 197.13 |
| **PVN** | 283.18 | **311.17** | **PVNQ** | 411.24 | **439.23** | **PVNQP** | 508.29 | 536.28 |
| **PVNQPT** | 609.34 | 637.33 | **VN** | 186.12 | 214.12 | **VNQ** | 314.18 | 342.18 |
| **VNQP** | 411.24 | **439.23** | **VNQPT** | 512.28 | 540.28 | **VNQPTV** | 611.35 | 639.35 |
| **NQ** | 215.11 | 243.11 | **NQP** | 312.17 | 340.16 | **NQPT** | 413.21 | 441.21 |
| **NQPTV** | 512.28 | 540.28 | **NQPTVD** | 627.31 | 655.30 | **QP** | 198.12 | 226.12 |
| **QPT** | 299.17 | 327.17 | **QPTV** | 398.24 | 426.23 | **QPTVD** | 513.27 | 541.26 |
| **QPTVDD** | 628.29 | 656.29 | **QPTVDDA** | 699.33 | 727.33 | **PT** | 171.11 | 199.11 |
| **PTV** | 270.18 | 298.18 | **PTVD** | 385.21 | 413.20 | **PTVDD** | 500.24 | **528.23** |
| **PTVDDA** | 571.27 | 599.27 | **TV** | 173.13 | 201.12 | **TVD** | 288.16 | 316.15 |
| **TVDD** | 403.18 | 431.18 | **TVDDA** | 474.22 | 502.21 | **TVDDAM** | 605.26 | 633.25 |
| **VD** | 187.11 | 215.10 | **VDD** | 302.13 | 330.13 | **VDDA** | 373.17 | 401.17 |
| **VDDAM** | 504.21 | 532.21 | **VDDAMV** | 603.28 | 631.28 | **DD** | **203.07** | 231.06 |
| **DDA** | 274.10 | 302.10 | **DDAM** | 405.14 | 433.14 | **DDAMV** | 504.21 | 532.21 |
| **DDAMVD** | 619.24 | 647.23 | **DA** | 159.08 | 187.07 | **DAM** | 290.12 | 318.11 |
| **DAMV** | **389.19** | 417.18 | **DAMVD** | 504.21 | 532.21 | **AM** | **175.09** | **203.08** |
| **AMV** | 274.16 | 302.15 | **AMVD** | **389.19** | 417.18 | **MV** | **203.12** | 231.12 |
| **MVD** | 318.15 | 346.14 | **VD** | 187.11 | 215.10 |  |  |  |

13. Tb927.8.3250

Match to: **Tb927.8.3250** Score: **85**

**dynein heavy chain, putative; Trypanosoma bruceichr 8Manual**

Nominal mass (M_r_): **579748**; Calculated pI value: **5.95**

NCBI BLAST search of [Tb927.8.3250](http://www.ncbi.nlm.nih.gov/blast/Blast.cgi?ALIGNMENTS=50&ALIGNMENT_VIEW=Pairwise&AUTO_FORMAT=Semiauto&CDD_SEARCH=on&CLIENT=web&COMPOSITION_BASED_STATISTICS=on&DATABASE=nr&DESCRIPTIONS=100&ENTREZ_QUERY=(none)&EXPECT=10&FILTER=L&FORMAT_BLOCK_ON_RESPAGE=None&FORMAT_OBJECT=Alignment&FORMAT_TYPE=HTML&GAPCOSTS=11+1&I_THRESH=0.001&LAYOUT=TwoWindows&MATRIX_NAME=BLOSUM62&NCBI_GI=on&PAGE=Proteins&PROGRAM=blastp&QUERY=Tb927.8.3250&SERVICE=plain&SET_DEFAULTS.x=21&SET_DEFAULTS.y=7&SHOW_OVERVIEW=on&WORD_SIZE=3&END_OF_HTTPGET=Yes) against nr

Unformatted [sequence string](../../../../D:%5CProteomic%20data%5C2010-1-8%5Ccgi%5Cgetseq.pl%3FTBA927_IPI+Tb927%2E8%2E3250+seq) for pasting into other applications

Fixed modifications: MMTS (C),(N-TERM)_iTRAQ,Lysine(K)_iTRAQ

Variable modifications: Oxidation (M)

Cleavage by Trypsin: cuts C-term side of KR unless next residue is P

Sequence Coverage: **1%**

Matched peptides shown in **Bold Red**

**1** MTSRVKFEVA EEGVIPECHP ECTVWMLQRI QAELQCKDEE FTNKHIRLIN

**51** DFLVGDEDLH ALFCYYSSVL VDVVSEDGEP SSESRSQQEM IAELRIVDGL

**101** PAVSRRDTMK **GMCLDVVWFA R**LDPEKLIVP ESVDTCIAWG VCRGGNLLEG

**151** FLRQLQYSIA PTLLQNRWPD SLEKDVRSAL HRFMAAVTEN VNRLKGQTVL

**201** YVPSDLFSKV DLAEAHQNRE LVQGFEAVVI HWTRQIKEVV GDKDAGLTGD

**251** GAGPLQEIAY WRSRARDLGN IRTQLNRSDV GGIVQVLKNA KSFYYLEPFL

**301** NLRADVEKGT DEAFDSLRFL NTLLEPCTRL SRAGPKEIPS LIPDVLIHAQ

**351** LILLYSKSYK KDRFFRLLRL ISNEIIFRCS QEIDVPAILN GDVERSMVAL

**401** RHSVAAGNAW IQECHKMLAA TRKRFKMERG EKLDVDDSFL NEIDGFVRHR

**451** CQNLCEICKA QLQFGFK**SVF QDSQISTDRT GGSR**HTRGVR NAAAVEKRQI

**501** AGGVAAYGAP LEVKDLVKTK GKEKDVFRGQ LPIFSGNKGP EIETQLLDIQ

**551** RAFKAKIDTL RRLDYDILDV KSTRWVDDFR ALKSDIDNLS MMLQQIITAA

**601** FDSFTTTEMG AEYIEAFFLV AETEELQLQL DRSKDRVFRM VHDRAMVVQG

**651** KLQRCFNKPP PIFYLHPPLA GHGMWAENNA HLLQMTTETL NHCYYLRESP

**701** ESTETIQLVD RLDRSLRDTM RQKFCEWRAN LPQNPGEYLE RFLISKRPNP

**751** RKHSLALYDV NFASELLLLF AEARYWHSLG ELLPVHIMDI VSKEERLRIY

**801** RESVAQAVRA RNSIALSLTR EECRLFSVRM NFLESKYMPG MTRLLWNSQG

**851** IVEYFVRECR QHVERVQHIV NEFKHGSEYV DHHCKAIADT IVVIFEKKKV

**901** YSIESFVEKQ EAHR**AATLEK** LQAIHRRLVD KLFELLSYFR DDYAEDDVVR

**951** TEWHRLISKV ELKVEEALRT MVKRTLQVVE R**MLPIEPSED RLEEK**VFKLD

**1001** VVVTVADDTR PHIEPVPSVR KLSHDVNGVC KAIIGIVKSI PRLEESLQAR

**1051** **VAQDQTDDAD AGKR**QPFQYS SSNTDSLALR GSYFEYMTSE QDAIYSLRHV

**1101** RESFDAIEEK VRDKLTQTWQ LHQSDTTDSL WTTQKQVRRI KQGWKLEDYR

**1151** IHMDHVAQRR EGINKQETFS DVLFLQLDFT KMKESFRKQC QLVITHYHSL

**1201** LYADAKSEVD AIYKNFVLTI QALTKEPQSL DELGDQIKRC AAATEALPEI

**1251** SAKFGPIADT FALITHDMYN FGSVRPEDVR RCEGLQEKFE VYSEQLVKAQ

**1301** QQLAKYKEQF RHDVETDIRA LSSNSYALRQ KVAEEGPRSH TLSTEDAFAK

**1351** LSSLGLRAKE LRTMESRLQQ GIEIFNLEKP QLDDLVAAEK ELEILRKIWN

**1401** LCDEWRRENS LWRTMYFIKL NSESMLDVCE RIRKDTLRLR NELQMTDVWV

**1451** NLKEEVELMK RLLPIVDDLR TPAIRPRHWE FLKVQLDATF NIDDESFCLN

**1501** DLMEARVETQ AEFVVNLATS AREEMKIETD LERIRTFWED SELMIEPYQG

**1551** YHKISGVDDI NNALAEHLAQ LSSMKMSRFV DSFRPKVIQW EQTLSIATDT

**1601** IEALLTVQTK WMYLENIFIG SDDIKRKLAA ESKKFDGVHS QWLAIITRFI

**1651** NDPNVVRGTR RDGLIDQLQN MNNSLEFIQK SLEGFLEDRR RVFPRFYFLS

**1701** NDDLLEILGH TKDPSKVQPH LRKCFEGLYQ LSLKTVRQRT VADAMLSSDG

**1751** ETVAFTPAVQ VGGLPVESWL RRVEVKMREM MQKRINATVD DLQKSVFETK

**1801** KSISRDSLKA WAERNEGQSI ITASCINWTL MTESAITEYG ELHSGGLGLQ

**1851** RRKASPLYKV YKRWKGMIKK YCQLVRQPQN RVQRSKLVAL ITIEVHSRDI

**1901** LRQVLAARVH QDDDFEWSRQ LRFYREEDES TDRPQEGHKI CLVRQTSATV

**1951** RYDYEYLGNS GRLVVTGLTD RAYMTLTTAL QLHRGGLPQG PAGTGKTETV

**2001** KDLGKAIGKY VMVFNCSDGL DYKSVGRMLS GIAQTGSWSC FDEFNRIEVE

**2051** VLSVVAQQIL SILTAVSERK DHFLFEGSDI PLNMNCGLFV TMNPGYAGRS

**2101** ELPDNLKALL RPISMMVPDF ALICEITLLS EGFEESETLS KKVSILYELM

**2151** EKQLSKQDHY DFSLRNIKAV LVQAGNLKRE GFPGTESQLC LKAMNDMNLP

**2201** KFVKDDVPLF VGMLNDLFPG VEPGDSGLGA LQEAAEKELD AEGLEVNAHI

**2251** VVKTLQLWDT LRTRHGVMVV GQTGSGKTVT WRNLSGALRL LKEQNLEPGL

**2301** YEPVRVSLLN PKSVTMDELY GSYNQATREW KDGILSDLMR QICRDITDTA

**2351** YKWMLFDGPV DTLWIESMNT VLDDNKMLTL NSGERITLNS TVRMMFEVQD

**2401** LSQASPATVS RCGMVYFNVE DLGWMPFFKT WLKSRWKFEI TMGAPRPDDT

**2451** ISELQEYVKN TVTRVLEYRA HECVELVPTT TLNVVRSFTR MLDALASVDA

**2501** EPFVPEAAHY ATSHAGENYL PQLRILATFC LMWSAGGSLT TESRQKLDAF

**2551** IRELDSSFPS TETIFEYFPD LGGLQWKNWN EHVDLQKTYM PATGTPYHKL

**2601** IVPTVDTVRY EYIVSQLVRS QVQLVLVGTT GTGKSLIARQ VLANLSNDVY

**2651** VTTQLNFSAQ TTAGNVQDII EGRMEHKSKK VCCPPGGRRM ICLVEDLNMP

**2701** AKEKFGAQPP LELLRQWLDN GYWYDRNTRG RRTVNDLQLL CCMTYGRPDI

**2751** TPRLMSKLNV FNITFPSESV ITKIFTSILM YRLEPYPELH KLVNSVVKAT

**2801** LQTYQKVSAD LLPTPSKSHY LFNLRDLSKV FQGIYGCHME YLQCKEHMVA

**2851** LWAHECFRVF SDRMNDPNDK AWFKNLICEK LADIFQTKWN NIIRARSRDS

**2901** RNQAVDEKEN PLFVDFWDGE YDEMAKYRLV PSLEALRDKV EEYLDAYNSE

**2951** PGARQMNLVF FTDALEHLCR IHRIVRQPRG NALLVGLGGS GRYSLTRLAT

**3001** YLAGYSIFSI ETHKKYDLDR FHEDLRSLYK GCGLKGQQRV FYFSDNQIMQ

**3051** PAFLEDLNNM LSTGEVPNLF PKDELQNIRD TVCKQAIASG YRDTPDEMYN

**3101** FFIDRARTNL HLVVAMSPAH KLFRARLRQF PALVSCTSID WFVEWPSEAL

**3151** REVGLRYLQE TRENKEDDEH LGIISDFFVY MHYTTSTLSR EMLEQVHRYN

**3201** YVTPSSYLDM VRGFRRMLTQ KRDEIIEQRD KLANGMAKLE ETKLAVSKMT

**3251** EELKVQDAKL QEKTEEVNRA TESIKVQQQN AEEQQSLLAS EKVKIEQTKR

**3301** SALADQAEAQ ADLDRAMPTL LEAQNALDKL EKNDINEIKS YKTPAAMIRT

**3351** VMYAVQTTLR RKLEWDEAKK SLSEPKFIDM LKHYHENNDM TDQRLLDKIE

**3401** KYVKRPDFTP AAASAVSKAA GGLCQWVIAI HKYGNIYKEV HPKIVKNENA

**3451** QQKVRAQEEM LRQKEEKLQR IMSEVKQLEL ALQQNVDEKM RLMQEAKETQ

**3501** MKLDRARIIV DGLEGEQDRW IESIARYEAA LGTLVGDALL VCGFLCYAGA

**3551** FTADYRQKLW LNWIKEIKRL QIAISKNFDF VEFLADPTEV RDWQQAGLPG

**3601** DDFSKENGAV VMRGTRWPLM IDPQLQAIKW IKRMEKDKGL KVIDQKQPDF

**3651** HKTVEYAVQF GCPLLLQDIL EEIDPLLDSV LSKAIVRKGA KPILKIGDNY

**3701** VEYNDNFKLY ITTRLPNPHY TPEICSKVCL LNFAVRETGL EEQLLKIVVE

**3751** KEKPELEQDN EQLILDTAEA RKETKRLEDE ILNLLSTSQV SLLENKKLVD

**3801** TLQSARVIAA NIKQQLKEAE ITAEKIHSAR EQYRECARRA SILFFALADL

**3851** GSIDAMYQFA LDSYIVLFQG SIQRSAQKIA THTLEERVRT LNDWHTSAVY

**3901** ANTCRGLFEK HKLLFTFHMT IRILQAEGLV NIEEYVFLMR GGQVLDKQGR

**3951** LPNPAPSWLS ERAWSHILEL DKLTNFHGVA ASFEQAQESW KHWFLQENPE

**4001** DAELPDDWQT RTADNYIQRM IFVRCLRPDR VIFMVYEFIE KQLGPQFVDP

**4051** PPFNLKDTFE ESTNVVPLVF VLSPGVDPTT QLAALAQREG RPLKTLALGQ

**4101** GQGENAKRAV QECSQVGGWV FLANCHLMVS WLVELEKIIE DLVEQRPHKE

**4151** FRLWLSSVPT TQFPIGILQR AIKMTTEPPT GIKANMLRLY NQFSEEQFAE

**4201** HTGSNPQIYC SLLFALCFFH SILLERRKFG NLGYNVVYDF TTSDFEVSEN

**4251** IIALYIGNMA TDRVEDIPFV TIRYLIAEAS YGGRVTDDWD RRVINTYISQ

**4301** FMCPAILTEE RYPLSAAEEY YIPSGISTLQ AYKDECSLLP ITDPPEAFGQ

**4351** HTNADIASRV AESTMLLDNL ISVNKTLARG GGSSGGASKG MSEEARCLEI

**4401** LASLEEPSKT AIPNPIDYDA VYESVKEDTN NALNTCLLQE IQRYNVLLRK

**4451** IIVQKRELRR AVKGEVLMTD ELEAVFNALL LSRVPPPWTS AYPSMKPLAS

**4501** WAVDLVERIE QMKQWGQRVP NVFWLSGFTY PTGFLKGLQQ QQARHDRISI

**4551** DQYTWEFVVL PSEERTIVNR AKKGAYVRGI FLEGAGWNEE MNTLCEPRPL

**4601** ELIVPMPIIH FKPKIRDTKP RPPTIYECPL YMYPLRTGTR ERPSFVVAVD

**4651** LESGEAVPEH YTKRGTALLL STDE

**Start - End Observed Mr(expt) Mr(calc) Delta Miss Sequence**

**111 - 121 1502.66 1501.65 1501.70 -0.05 0 K.GMCLDVVWFAR.L**  Oxidation (M) ([Ions score 5](../../../../D:%5CProteomic%20data%5C2010-1-8%5CZQ%5C1017.htm))

**468 - 484 1985.03 1984.03 1983.97 0.05 1 K.SVFQDSQISTDRTGGSR.H**  ([Ions score 3](../../../../D:%5CProteomic%20data%5C2010-1-8%5CZQ%5C1020.htm))

**915 - 920 920.51 919.50 919.47 0.03 0 R.AATLEK.L**  ([Ions score 9](../../../../D:%5CProteomic%20data%5C2010-1-8%5CZQ%5C1016.htm))

**982 - 995 1974.04 1973.03 1972.95 0.09 1 R.MLPIEPSEDRLEEK.V**  ([Ions score 7](../../../../D:%5CProteomic%20data%5C2010-1-8%5CZQ%5C1019.htm))

**1051 - 1064 1777.89 1776.88 1776.80 0.09 1 R.VAQDQTDDADAGKR.Q**  ([Ions score 85](../../../../D:%5CProteomic%20data%5C2010-1-8%5CZQ%5C1018.htm))

MS/MS Fragmentation of **VAQDQTDDADAGKR**
Found in **Tb927.8.3250**, dynein heavy chain, putative; Trypanosoma bruceichr 8Manual


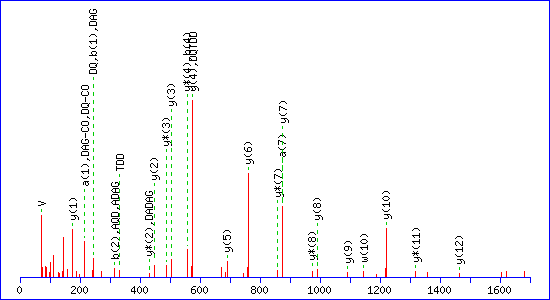


**MONOISOTOPIC mass of neutral peptide Mr(calc):** 1776.80

**Fixed modifications:** MMTS (C),(N-TERM)_iTRAQ,Lysine(K)_iTRAQ

**Ions Score:** 85 **Expect:** 4.7e-007

**Matches (Bold Red):** 35/230 fragment ions using 31 most intense peaks

| **#** | **Immon.** | **a** | **a*** | **a^0^** | **b** | **b*** | **b^0^** | **Seq.** | **v** | **w** | **w'** | **y** | **y*** | **y^0^** | **#** |
| --- | --- | --- | --- | --- | --- | --- | --- | --- | --- | --- | --- | --- | --- | --- | --- |
| **1** | **72.08** | **216.18** |  |  | **244.18** |  |  | **V** |  |  |  |  |  |  | **14** |
| **2** | 44.05 | 287.22 |  |  | **315.22** |  |  | **A** | 1518.60 |  |  | 1534.63 | 1517.61 | 1516.62 | **13** |
| **3** | 101.07 | 415.28 | 398.25 |  | 443.27 | 426.25 |  | **Q** | 1390.54 | 1389.55 |  | **1463.59** | 1446.57 | 1445.58 | **12** |
| **4** | 88.04 | 530.31 | 513.28 | 512.30 | **558.30** | 541.27 | 540.29 | **D** | 1275.51 | 1274.52 |  | 1335.54 | **1318.51** | 1317.53 | **11** |
| **5** | 101.07 | 658.36 | 641.34 | 640.35 | 686.36 | 669.33 | 668.35 | **Q** | 1147.46 | **1146.46** |  | **1220.51** | 1203.48 | 1202.50 | **10** |
| **6** | 74.06 | 759.41 | 742.39 | 741.40 | 787.41 | 770.38 | 769.40 | **T** | 1046.41 | 1059.43 | 1061.41 | **1092.45** | 1075.42 | 1074.44 | **9** |
| **7** | 88.04 | **874.44** | 857.41 | 856.43 | 902.43 | 885.41 | 884.42 | **D** | 931.38 | 930.39 |  | **991.40** | **974.38** | 973.39 | **8** |
| **8** | 88.04 | 989.47 | 972.44 | 971.46 | 1017.46 | 1000.43 | 999.45 | **D** | 816.35 | 815.36 |  | **876.38** | **859.35** | 858.37 | **7** |
| **9** | 44.05 | 1060.50 | 1043.48 | 1042.49 | 1088.50 | 1071.47 | 1070.49 | **A** | 745.32 |  |  | **761.35** | 744.32 | 743.34 | **6** |
| **10** | 88.04 | 1175.53 | 1158.50 | 1157.52 | 1203.52 | 1186.50 | 1185.51 | **D** | 630.29 | 629.30 |  | **690.31** | 673.29 | 672.30 | **5** |
| **11** | 44.05 | 1246.57 | 1229.54 | 1228.56 | 1274.56 | 1257.54 | 1256.55 | **A** | 559.25 |  |  | **575.28** | **558.26** |  | **4** |
| **12** | 30.03 | 1303.59 | 1286.56 | 1285.58 | 1331.58 | 1314.56 | 1313.57 | **G** |  |  |  | **504.25** | **487.22** |  | **3** |
| **13** | 245.12 | 1575.70 | 1558.67 | 1557.69 | 1603.69 | 1586.66 | 1585.68 | **K** | 230.12 | 229.13 |  | **447.23** | **430.20** |  | **2** |
| **14** | 129.11 |  |  |  |  |  |  | **R** | 74.02 | 73.03 |  | **175.12** | 158.09 |  | **1** |

| **Seq** | **ya** | **yb** | **Seq** | **ya** | **yb** | **Seq** | **ya** | **yb** |
| --- | --- | --- | --- | --- | --- | --- | --- | --- |
| **AQ** | 172.11 | 200.10 | **AQD** | 287.13 | **315.13** | **AQDQ** | 415.19 | 443.19 |
| **AQDQT** | 516.24 | 544.24 | **AQDQTD** | 631.27 | 659.26 | **QD** | **216.10** | **244.09** |
| **QDQ** | 344.16 | 372.15 | **QDQT** | 445.20 | 473.20 | **QDQTD** | 560.23 | 588.23 |
| **QDQTDD** | 675.26 | 703.25 | **DQ** | **216.10** | **244.09** | **DQT** | 317.15 | 345.14 |
| **DQTD** | 432.17 | 460.17 | **DQTDD** | 547.20 | **575.19** | **DQTDDA** | 618.24 | 646.23 |
| **QT** | 202.12 | 230.11 | **QTD** | 317.15 | 345.14 | **QTDD** | 432.17 | 460.17 |
| **QTDDA** | 503.21 | 531.20 | **QTDDAD** | 618.24 | 646.23 | **QTDDADA** | 689.27 | 717.27 |
| **TD** | 189.09 | 217.08 | **TDD** | 304.11 | **332.11** | **TDDA** | 375.15 | 403.15 |
| **TDDAD** | 490.18 | 518.17 | **TDDADA** | 561.22 | 589.21 | **TDDADAG** | 618.24 | 646.23 |
| **DD** | 203.07 | 231.06 | **DDA** | 274.10 | 302.10 | **DDAD** | 389.13 | 417.13 |
| **DDADA** | 460.17 | 488.16 | **DDADAG** | 517.19 | 545.18 | **DA** | 159.08 | 187.07 |
| **DAD** | 274.10 | 302.10 | **DADA** | 345.14 | 373.14 | **DADAG** | 402.16 | **430.16** |
| **DADAGK** | 674.27 | 702.26 | **AD** | 159.08 | 187.07 | **ADA** | 230.11 | 258.11 |
| **ADAG** | 287.13 | **315.13** | **ADAGK** | 559.24 | 587.24 | **DA** | 159.08 | 187.07 |
| **DAG** | **216.10** | **244.09** | **DAGK** | 488.21 | 516.20 | **AG** | 101.07 | 129.07 |
| **AGK** | 373.18 | 401.17 | **GK** | 302.14 | 330.14 |  |  |  |

14. Tb11.01.6870

Match to: **Tb11.01.6870** Score: **83**

**calpain-like cysteine peptidase, putative; Trypanosoma bruceichr 11Manual**

Nominal mass (M_r_): **35308**; Calculated pI value: **4.99**

NCBI BLAST search of [Tb11.01.6870](http://www.ncbi.nlm.nih.gov/blast/Blast.cgi?ALIGNMENTS=50&ALIGNMENT_VIEW=Pairwise&AUTO_FORMAT=Semiauto&CDD_SEARCH=on&CLIENT=web&COMPOSITION_BASED_STATISTICS=on&DATABASE=nr&DESCRIPTIONS=100&ENTREZ_QUERY=(none)&EXPECT=10&FILTER=L&FORMAT_BLOCK_ON_RESPAGE=None&FORMAT_OBJECT=Alignment&FORMAT_TYPE=HTML&GAPCOSTS=11+1&I_THRESH=0.001&LAYOUT=TwoWindows&MATRIX_NAME=BLOSUM62&NCBI_GI=on&PAGE=Proteins&PROGRAM=blastp&QUERY=MPRKALLGNWFEEEAYMRDRKRLLDSCDRGVVDAARETQRIIAKVKHHNSAYPMAEPHEDGYLHFYAPLMLQNAATLGFLSLDLEDRTLRPTGWHVACSTAPAAGPALRNCFVLVPAPTGPTDMIPAPPDEQDIVHYGQPFFIMTVPELCDNPLSLLSEPKGPLSASKVTGKHQDVFFSPDGASAEAMWVADFANPDHREDMRDLPIKADAVLVIRHNHTNTPLASSKAVFFNDFGPENEVCCGRFVNNPGTPCGPMKDENYWTFVHSENGEGDVEKGGETTTPVAENEVSVMTSTNGDKL&SERVICE=plain&SET_DEFAULTS.x=9&SET_DEFAULTS.y=5&SHOW_OVERVIEW=on&WORD_SIZE=3&END_OF_HTTPGET=Yes) against nr

Unformatted [sequence string](../../../../D:%5CProteomic%20data%5C2010-1-8%5Ccgi%5Cgetseq.pl%3FTBA927_IPI+Tb11%2E01%2E6870+seq) for pasting into other applications

Fixed modifications: MMTS (C),(N-TERM)_iTRAQ,Lysine(K)_iTRAQ

Variable modifications: Oxidation (M)

Cleavage by Trypsin: cuts C-term side of KR unless next residue is P

Sequence Coverage: **4%**

Matched peptides shown in **Bold Red**

**1** MPRK**ALLGNW FEEEAYMR**DR KRLLDSCDRG VVDAARETQR IIAKVKHHNS

**51** AYPMAEPHED GYLHFYAPLM LQNAATLGFL SLDLEDRTLR PTGWHVACST

**101** APAAGPALRN CFVLVPAPTG PTDMIPAPPD EQDIVHYGQP FFIMTVPELC

**151** DNPLSLLSEP KGPLSASKVT GKHQDVFFSP DGASAEAMWV ADFANPDHRE

**201** DMRDLPIKAD AVLVIRHNHT NTPLASSKAV FFNDFGPENE VCCGRFVNNP

**251** GTPCGPMKDE NYWTFVHSEN GEGDVEKGGE TTTPVAENEV SVMTSTNGDK

**301** L

MS/MS Fragmentation of **ALLGNWFEEEAYMR**
Found in **Tb11.01.6870**, calpain-like cysteine peptidase, putative; Trypanosoma bruceichr 11Manual


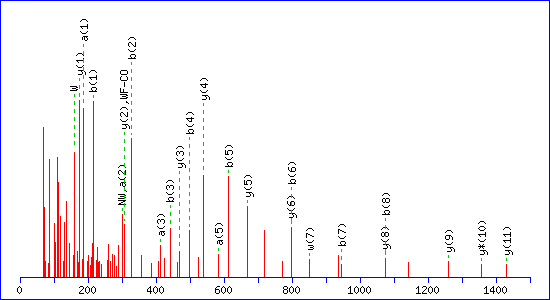


**MONOISOTOPIC mass of neutral peptide Mr(calc):** 1871.90

**Fixed modifications:** MMTS (C),(N-TERM)_iTRAQ,Lysine(K)_iTRAQ

**Ions Score:** 83 **Expect:** 5.3e-007

**Matches (Bold Red):** 26/199 fragment ions using 30 most intense peaks

| **#** | **Immon.** | **a** | **a*** | **a^0^** | **b** | **b*** | **b^0^** | **Seq.** | **v** | **w** | **y** | **y*** | **y^0^** | **#** |
| --- | --- | --- | --- | --- | --- | --- | --- | --- | --- | --- | --- | --- | --- | --- |
| **1** | 44.05 | **188.15** |  |  | **216.15** |  |  | **A** |  |  |  |  |  | **14** |
| **2** | 86.10 | **301.24** |  |  | **329.23** |  |  | **L** | 1599.69 | 1598.69 | 1657.77 | 1640.74 | 1639.76 | **13** |
| **3** | 86.10 | **414.32** |  |  | **442.31** |  |  | **L** | 1486.61 | 1485.61 | 1544.68 | 1527.66 | 1526.67 | **12** |
| **4** | 30.03 | 471.34 |  |  | **499.34** |  |  | **G** |  |  | **1431.60** | 1414.57 | 1413.59 | **11** |
| **5** | 87.06 | **585.38** | 568.36 |  | **613.38** | 596.35 |  | **N** | 1315.54 | 1314.55 | 1374.58 | **1357.55** | 1356.57 | **10** |
| **6** | **159.09** | 771.46 | 754.44 |  | **799.46** | 782.43 |  | **W** | 1129.46 |  | **1260.54** | 1243.51 | 1242.52 | **9** |
| **7** | 120.08 | 918.53 | 901.51 |  | **946.53** | 929.50 |  | **F** | 982.39 |  | **1074.46** | 1057.43 | 1056.45 | **8** |
| **8** | 102.05 | 1047.57 | 1030.55 | 1029.56 | **1075.57** | 1058.54 | 1057.56 | **E** | 853.35 | **852.36** | 927.39 | 910.36 | 909.38 | **7** |
| **9** | 102.05 | 1176.62 | 1159.59 | 1158.61 | 1204.61 | 1187.59 | 1186.60 | **E** | 724.31 | 723.31 | **798.35** | 781.32 | 780.33 | **6** |
| **10** | 102.05 | 1305.66 | 1288.63 | 1287.65 | 1333.65 | 1316.63 | 1315.64 | **E** | 595.27 | 594.27 | **669.30** | 652.28 | 651.29 | **5** |
| **11** | 44.05 | 1376.70 | 1359.67 | 1358.69 | 1404.69 | 1387.67 | 1386.68 | **A** | 524.23 |  | **540.26** | 523.23 |  | **4** |
| **12** | 136.08 | 1539.76 | 1522.73 | 1521.75 | 1567.76 | 1550.73 | 1549.74 | **Y** | 361.17 |  | **469.22** | 452.20 |  | **3** |
| **13** | 104.05 | 1670.80 | 1653.77 | 1652.79 | 1698.80 | 1681.77 | 1680.79 | **M** | 230.12 | 229.13 | **306.16** | 289.13 |  | **2** |
| **14** | 129.11 |  |  |  |  |  |  | **R** | 74.02 | 73.03 | **175.12** | 158.09 |  | **1** |

| **Seq** | **ya** | **yb** | **Seq** | **ya** | **yb** | **Seq** | **ya** | **yb** |
| --- | --- | --- | --- | --- | --- | --- | --- | --- |
| **LL** | 199.18 | 227.18 | **LLG** | 256.20 | 284.20 | **LLGN** | 370.24 | 398.24 |
| **LLGNW** | 556.32 | 584.32 | **LG** | 143.12 | 171.11 | **LGN** | 257.16 | 285.16 |
| **LGNW** | 443.24 | 471.24 | **LGNWF** | 590.31 | 618.30 | **GN** | 144.08 | 172.07 |
| **GNW** | 330.16 | 358.15 | **GNWF** | 477.22 | 505.22 | **GNWFE** | 606.27 | 634.26 |
| **NW** | 273.13 | **301.13** | **NWF** | 420.20 | 448.20 | **NWFE** | 549.25 | 577.24 |
| **NWFEE** | 678.29 | 706.28 | **WF** | **306.16** | 334.15 | **WFE** | 435.20 | 463.20 |
| **WFEE** | 564.25 | 592.24 | **WFEEE** | 693.29 | 721.28 | **FE** | 249.12 | 277.12 |
| **FEE** | 378.17 | 406.16 | **FEEE** | 507.21 | 535.20 | **FEEEA** | 578.25 | 606.24 |
| **EE** | 231.10 | 259.09 | **EEE** | 360.14 | 388.14 | **EEEA** | 431.18 | 459.17 |
| **EEEAY** | 594.24 | 622.24 | **EE** | 231.10 | 259.09 | **EEA** | 302.13 | 330.13 |
| **EEAY** | 465.20 | 493.19 | **EEAYM** | 596.24 | 624.23 | **EA** | 173.09 | 201.09 |
| **EAY** | 336.16 | 364.15 | **EAYM** | 467.20 | 495.19 | **AY** | 207.11 | 235.11 |
| **AYM** | 338.15 | 366.15 | **YM** | 267.12 | 295.11 |  |  |  |

15. Tb10.26.1080

Match to: **Tb10.26.1080** Score: **83**

**heat shock protein 83; Trypanosoma bruceichr 10Manual**

Nominal mass (M_r_): **91449**; Calculated pI value: **5.08**

NCBI BLAST search of [Tb10.26.1080](http://www.ncbi.nlm.nih.gov/blast/Blast.cgi?ALIGNMENTS=50&ALIGNMENT_VIEW=Pairwise&AUTO_FORMAT=Semiauto&CDD_SEARCH=on&CLIENT=web&COMPOSITION_BASED_STATISTICS=on&DATABASE=nr&DESCRIPTIONS=100&ENTREZ_QUERY=(none)&EXPECT=10&FILTER=L&FORMAT_BLOCK_ON_RESPAGE=None&FORMAT_OBJECT=Alignment&FORMAT_TYPE=HTML&GAPCOSTS=11+1&I_THRESH=0.001&LAYOUT=TwoWindows&MATRIX_NAME=BLOSUM62&NCBI_GI=on&PAGE=Proteins&PROGRAM=blastp&QUERY=MTETFAFQAEINQLMSLIINTFYSNKEIFLRELISNSSDACDKIRYQSLTNQSVLGDEPHLRIRVIPDRVNKTLTVEDSGIGMTKADLVNNLGTIARSGTKSFMEALEAGGDMSMIGQFGVGFYSAYLVADRVTVVSKNNEDDAYTWESSAGGTFTVTSTPDCDLKRGTRIVLHLKEDQQEYLEERRLKDLIKKHSEFIGYDIELMVENTTEKEVTDEDEDEEAAKKAEEGEEPKVEEVKDGDDADAKKKKTKKVKEVKQEFVVQNKHKPLWTRDPKDVTKEEYASFYKAISNDWEEQLSTKHFSVEGQLEFRAILFLPKRAPFDMFEPNKKRNNIKLYVRRVFIMDNCEDLCPEWLGFLRGVVDSEDLPLNISRENLQQNKILKVIRKNIVKKALELFEELAENKEDYKKFYEQFSKNVKLGIHEDSTNRKKLMELLRFHSSESGEEMTTLKDYVTRMKDGQKCIYYVTGDSKKKLETSPFIEQAKRRGMEVLFMTDPIDEYVMQQVKDFEDKKFACLTKEGVHFEETEEEKKQREEEKASYERLCKAMKEVLGDKVEKVVVSDRLATSPCILVTSEFGWSAHMEQIMRNQALRDSSMSAYMMSKKTMEINTTHAIVKELKRRVEADENDKAAKDLIFLLFDTSLLTSGFTLDDPTAYADRIHRMIKLGLSLDDDAEEEEAQAPVAAAAANSSTGASGMEEVD&SERVICE=plain&SET_DEFAULTS.x=9&SET_DEFAULTS.y=5&SHOW_OVERVIEW=on&WORD_SIZE=3&END_OF_HTTPGET=Yes) against nr

Unformatted [sequence string](../../../../D:%5CProteomic%20data%5C2010-1-8%5Ccgi%5Cgetseq.pl%3FTBA927_IPI+Tb10%2E26%2E1080+seq) for pasting into other applications

Fixed modifications: MMTS (C),(N-TERM)_iTRAQ,Lysine(K)_iTRAQ

Variable modifications: Oxidation (M)

Cleavage by Trypsin: cuts C-term side of KR unless next residue is P

Sequence Coverage: **5%**

Matched peptides shown in **Bold Red**

**1** MTETFAFQAE INQLMSLIIN TFYSNKEIFL RELISNSSDA CDKIR**YQSLT**

**51 NQSVLGDEPH LR**IRVIPDRV NK**TLTVEDSG IGMTKADLVN NLGTIAR**SGT

**101** KSFMEALEAG GDMSMIGQFG VGFYSAYLVA DRVTVVSKNN EDDAYTWESS

**151** AGGTFTVTST PDCDLKRGTR IVLHLKEDQQ EYLEERRLKD LIKKHSEFIG

**201** YDIELMVENT TEKEVTDEDE DEEAAKKAEE GEEPKVEEVK DGDDADAKKK

**251** KTKKVKEVKQ EFVVQNKHKP LWTRDPKDVT KEEYASFYKA ISNDWEEQLS

**301** TKHFSVEGQL EFRAILFLPK RAPFDMFEPN KKRNNIKLYV RRVFIMDNCE

**351** DLCPEWLGFL RGVVDSEDLP LNISRENLQQ NKILKVIRKN IVKKALELFE

**401** ELAENKEDYK KFYEQFSKNV KLGIHEDSTN RKKLMELLRF HSSESGEEMT

**451** TLKDYVTRMK DGQKCIYYVT GDSKKKLETS PFIEQAKRRG MEVLFMTDPI

**501** DEYVMQQVKD FEDKKFACLT KEGVHFEETE EEKKQREEEK ASYERLCKAM

**551** KEVLGDKVEK VVVSDRLATS PCILVTSEFG WSAHMEQIMR NQALRDSSMS

**601** AYMMSKKTME INTTHAIVKE LKRRVEADEN DKAAKDLIFL LFDTSLLTSG

**651** FTLDDPTAYA DRIHRMIKLG LSLDDDAEEE EAQAPVAAAA ANSSTGASGM

**701** EEVD

**Start - End Observed Mr(expt) Mr(calc) Delta Miss Sequence**

**46 - 62 2101.08 2100.07 2100.07 -0.00 0 R.YQSLTNQSVLGDEPHLR.I**  ([Ions score 83](../../../../D:%5CProteomic%20data%5C2010-1-8%5CZQ%5C1024.htm))

**73 - 97 2877.62 2876.61 2876.46 0.15 1 K.TLTVEDSGIGMTKADLVNNLGTIAR.S**  ([Ions score 1](../../../../D:%5CProteomic%20data%5C2010-1-8%5CZQ%5C1025.htm))

MS/MS Fragmentation of **YQSLTNQSVLGDEPHLR**
Found in **Tb10.26.1080**, heat shock protein 83; Trypanosoma bruceichr 10Manual


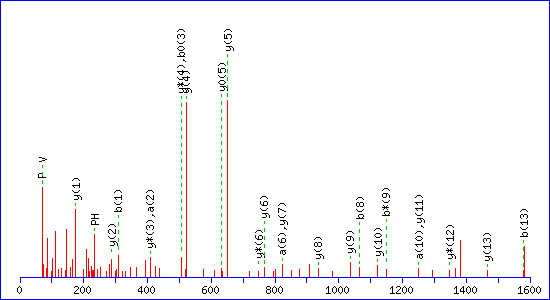


**MONOISOTOPIC mass of neutral peptide Mr(calc):** 2100.07

**Fixed modifications:** MMTS (C),(N-TERM)_iTRAQ,Lysine(K)_iTRAQ

**Ions Score:** 83 **Expect:** 5e-007

**Matches (Bold Red):** 27/305 fragment ions using 29 most intense peaks

| **#** | **Immon.** | **a** | **a*** | **a^0^** | **b** | **b*** | **b^0^** | **Seq.** | **v** | **w** | **w'** | **y** | **y*** | **y^0^** | **#** |
| --- | --- | --- | --- | --- | --- | --- | --- | --- | --- | --- | --- | --- | --- | --- | --- |
| **1** | 136.08 | 280.18 |  |  | **308.17** |  |  | **Y** |  |  |  |  |  |  | **17** |
| **2** | 101.07 | **408.24** | 391.21 |  | 436.23 | 419.21 |  | **Q** | 1720.86 | 1719.87 |  | 1793.91 | 1776.89 | 1775.90 | **16** |
| **3** | 60.04 | 495.27 | 478.24 | 477.26 | 523.26 | 506.24 | **505.25** | **S** | 1633.83 | 1632.83 |  | 1665.86 | 1648.83 | 1647.84 | **15** |
| **4** | 86.10 | 608.35 | 591.33 | 590.34 | 636.35 | 619.32 | 618.34 | **L** | 1520.75 | 1519.75 |  | 1578.82 | 1561.80 | 1560.81 | **14** |
| **5** | 74.06 | 709.40 | 692.37 | 691.39 | 737.40 | 720.37 | 719.38 | **T** | 1419.70 | 1432.72 | 1434.70 | **1465.74** | 1448.71 | 1447.73 | **13** |
| **6** | 87.06 | **823.44** | 806.42 | 805.43 | 851.44 | 834.41 | 833.43 | **N** | 1305.65 | 1304.66 |  | 1364.69 | **1347.67** | 1346.68 | **12** |
| **7** | 101.07 | 951.50 | 934.48 | 933.49 | 979.50 | 962.47 | 961.49 | **Q** | 1177.60 | 1176.60 |  | **1250.65** | 1233.62 | 1232.64 | **11** |
| **8** | 60.04 | 1038.53 | 1021.51 | 1020.52 | **1066.53** | 1049.50 | 1048.52 | **S** | 1090.56 | 1089.57 |  | **1122.59** | 1105.56 | 1104.58 | **10** |
| **9** | **72.08** | 1137.60 | 1120.58 | 1119.59 | 1165.60 | **1148.57** | 1147.59 | **V** | 991.50 | 1004.52 |  | **1035.56** | 1018.53 | 1017.55 | **9** |
| **10** | 86.10 | **1250.69** | 1233.66 | 1232.68 | 1278.68 | 1261.65 | 1260.67 | **L** | 878.41 | 877.42 |  | **936.49** | 919.46 | 918.48 | **8** |
| **11** | 30.03 | 1307.71 | 1290.68 | 1289.70 | 1335.70 | 1318.68 | 1317.69 | **G** |  |  |  | **823.41** | 806.38 | 805.40 | **7** |
| **12** | 88.04 | 1422.73 | 1405.71 | 1404.72 | 1450.73 | 1433.70 | 1432.72 | **D** | 706.36 | 705.37 |  | **766.38** | **749.36** | 748.37 | **6** |
| **13** | 102.05 | 1551.78 | 1534.75 | 1533.77 | **1579.77** | 1562.75 | 1561.76 | **E** | 577.32 | 576.33 |  | **651.36** | 634.33 | **633.35** | **5** |
| **14** | **70.07** | 1648.83 | 1631.80 | 1630.82 | 1676.83 | 1659.80 | 1658.81 | **P** | 480.27 | 479.27 |  | **522.31** | **505.29** |  | **4** |
| **15** | 110.07 | 1785.89 | 1768.86 | 1767.88 | 1813.88 | 1796.86 | 1795.87 | **H** | 343.21 |  |  | 425.26 | **408.24** |  | **3** |
| **16** | 86.10 | 1898.97 | 1881.95 | 1880.96 | 1926.97 | 1909.94 | 1908.96 | **L** | 230.12 | 229.13 |  | **288.20** | 271.18 |  | **2** |
| **17** | 129.11 |  |  |  |  |  |  | **R** | 74.02 | 73.03 |  | **175.12** | 158.09 |  | **1** |

| **Seq** | **ya** | **yb** | **Seq** | **ya** | **yb** | **Seq** | **ya** | **yb** |
| --- | --- | --- | --- | --- | --- | --- | --- | --- |
| **QS** | 188.10 | 216.10 | **QSL** | 301.19 | 329.18 | **QSLT** | 402.23 | 430.23 |
| **QSLTN** | 516.28 | 544.27 | **QSLTNQ** | 644.34 | 672.33 | **SL** | 173.13 | 201.12 |
| **SLT** | 274.18 | 302.17 | **SLTN** | 388.22 | 416.21 | **SLTNQ** | 516.28 | 544.27 |
| **SLTNQS** | 603.31 | 631.30 | **LT** | 187.14 | 215.14 | **LTN** | 301.19 | 329.18 |
| **LTNQ** | 429.25 | 457.24 | **LTNQS** | 516.28 | 544.27 | **LTNQSV** | 615.35 | 643.34 |
| **TN** | 188.10 | 216.10 | **TNQ** | 316.16 | 344.16 | **TNQS** | 403.19 | 431.19 |
| **TNQSV** | 502.26 | 530.26 | **TNQSVL** | 615.35 | 643.34 | **TNQSVLG** | 672.37 | 700.36 |
| **NQ** | 215.11 | 243.11 | **NQS** | 302.15 | 330.14 | **NQSV** | 401.21 | 429.21 |
| **NQSVL** | 514.30 | 542.29 | **NQSVLG** | 571.32 | 599.31 | **NQSVLGD** | 686.35 | 714.34 |
| **QS** | 188.10 | 216.10 | **QSV** | 287.17 | 315.17 | **QSVL** | 400.26 | 428.25 |
| **QSVLG** | 457.28 | 485.27 | **QSVLGD** | 572.30 | 600.30 | **SV** | 159.11 | 187.11 |
| **SVL** | 272.20 | 300.19 | **SVLG** | 329.22 | 357.21 | **SVLGD** | 444.25 | 472.24 |
| **SVLGDE** | 573.29 | 601.28 | **SVLGDEP** | 670.34 | 698.34 | **VL** | 185.16 | 213.16 |
| **VLG** | 242.19 | 270.18 | **VLGD** | 357.21 | 385.21 | **VLGDE** | 486.26 | 514.25 |
| **VLGDEP** | 583.31 | 611.30 | **LG** | 143.12 | 171.11 | **LGD** | 258.14 | 286.14 |
| **LGDE** | 387.19 | 415.18 | **LGDEP** | 484.24 | 512.24 | **LGDEPH** | 621.30 | 649.29 |
| **GD** | 145.06 | 173.06 | **GDE** | 274.10 | 302.10 | **GDEP** | 371.16 | 399.15 |
| **GDEPH** | 508.22 | 536.21 | **GDEPHL** | 621.30 | 649.29 | **DE** | 217.08 | 245.08 |
| **DEP** | 314.13 | 342.13 | **DEPH** | 451.19 | 479.19 | **DEPHL** | 564.28 | 592.27 |
| **EP** | 199.11 | 227.10 | **EPH** | 336.17 | 364.16 | **EPHL** | 449.25 | 477.25 |
| **PH** | 207.12 | **235.12** | **PHL** | 320.21 | 348.20 | **HL** | 223.16 | 251.15 |

16. Tb927.4.4040

Match to: **Tb927.4.4040** Score: **82**

**hypothetical protein, conserved; Trypanosoma bruceichr 4Manual**

Nominal mass (M_r_): **32311**; Calculated pI value: **4.79**

NCBI BLAST search of [Tb927.4.4040](http://www.ncbi.nlm.nih.gov/blast/Blast.cgi?ALIGNMENTS=50&ALIGNMENT_VIEW=Pairwise&AUTO_FORMAT=Semiauto&CDD_SEARCH=on&CLIENT=web&COMPOSITION_BASED_STATISTICS=on&DATABASE=nr&DESCRIPTIONS=100&ENTREZ_QUERY=(none)&EXPECT=10&FILTER=L&FORMAT_BLOCK_ON_RESPAGE=None&FORMAT_OBJECT=Alignment&FORMAT_TYPE=HTML&GAPCOSTS=11+1&I_THRESH=0.001&LAYOUT=TwoWindows&MATRIX_NAME=BLOSUM62&NCBI_GI=on&PAGE=Proteins&PROGRAM=blastp&QUERY=MDNTEETSRVTDQEERTLTETELPAPAEHVLEGDNSASEENRLHAFLIPLEDGEQFARANLQHRWYRGLTKMLWDEELERNGILIVERGESGRNNQSADEEEEEDDDGTDDCVAKMKREFRIKREQDKIVYEEDSTRTETLREEQNAISLIVTLMEEAMDRSILMNEAETMFALILDVLKPRVHYRYIYAWGVVTCCSFKPLKSEMDRLRPSDRHKPPYMRKSLPTCRVPLEPIYYLPDGGKKMRAMNELNDVRAIGWV&SERVICE=plain&SET_DEFAULTS.x=9&SET_DEFAULTS.y=5&SHOW_OVERVIEW=on&WORD_SIZE=3&END_OF_HTTPGET=Yes) against nr

Unformatted [sequence string](../../../../D:%5CProteomic%20data%5C2010-1-8%5Ccgi%5Cgetseq.pl%3FTBA927_IPI+Tb927%2E4%2E4040+seq) for pasting into other applications

Fixed modifications: MMTS (C),(N-TERM)_iTRAQ,Lysine(K)_iTRAQ

Variable modifications: Oxidation (M)

Cleavage by Trypsin: cuts C-term side of KR unless next residue is P

Sequence Coverage: **9%**

Matched peptides shown in **Bold Red**

**1** MDNTEETSRV TDQEERTLTE TELPAPAEHV LEGDNSASEE NR**LHAFLIPL**

**51 EDGEQFAR**AN LQHRWYRGLT KMLWDEELER NGILIVERGE SGRNNQSADE

**101** EEEEDDDGTD DCVAKMKREF RIKREQDKIV YEEDSTRTET LREEQNAISL

**151** IVTLMEEAMD RSILMNEAET MFALILDVLK PRVHYRYIYA WGVVTCCSFK

**201** PLKSEMDRLR PSDRHKPPYM RKSLPTCRVP LEPIYYLPDG GKKMR**AMNEL**

**251 NDVR**AIGWV

**Start - End Observed Mr(expt) Mr(calc) Delta Miss Sequence**

**43 - 58 2000.07 1999.06 1999.07 -0.01 0 R.LHAFLIPLEDGEQFAR.A**  ([Ions score 82](../../../../D:%5CProteomic%20data%5C2010-1-8%5CZQ%5C1036.htm))

**246 - 254 1205.62 1204.62 1204.60 0.02 0 R.AMNELNDVR.A**  ([Ions score 19](../../../../D:%5CProteomic%20data%5C2010-1-8%5CZQ%5C1035.htm))

MS/MS Fragmentation of **LHAFLIPLEDGEQFAR**
Found in **Tb927.4.4040**, hypothetical protein, conserved; Trypanosoma bruceichr 4Manual


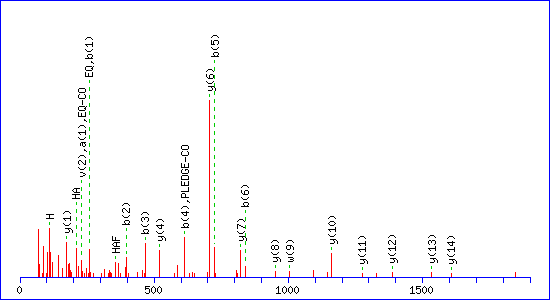


**MONOISOTOPIC mass of neutral peptide Mr(calc):** 1999.07

**Fixed modifications:** MMTS (C),(N-TERM)_iTRAQ,Lysine(K)_iTRAQ

**Ions Score:** 82 **Expect:** 6.3e-007

**Matches (Bold Red):** 25/239 fragment ions using 23 most intense peaks

| **#** | **Immon.** | **a** | **a*** | **a^0^** | **b** | **b*** | **b^0^** | **Seq.** | **v** | **w** | **w'** | **y** | **y*** | **y^0^** | **#** |
| --- | --- | --- | --- | --- | --- | --- | --- | --- | --- | --- | --- | --- | --- | --- | --- |
| **1** | 86.10 | **230.20** |  |  | **258.19** |  |  | **L** |  |  |  |  |  |  | **16** |
| **2** | **110.07** | 367.26 |  |  | **395.25** |  |  | **H** | 1660.83 |  |  | 1742.89 | 1725.86 | 1724.88 | **15** |
| **3** | 44.05 | 438.29 |  |  | **466.29** |  |  | **A** | 1589.80 |  |  | **1605.83** | 1588.80 | 1587.82 | **14** |
| **4** | 120.08 | 585.36 |  |  | **613.36** |  |  | **F** | 1442.73 |  |  | **1534.79** | 1517.76 | 1516.78 | **13** |
| **5** | 86.10 | 698.45 |  |  | **726.44** |  |  | **L** | 1329.64 | 1328.65 |  | **1387.72** | 1370.70 | 1369.71 | **12** |
| **6** | 86.10 | 811.53 |  |  | **839.53** |  |  | **I** | 1216.56 | 1229.58 | 1243.60 | **1274.64** | 1257.61 | 1256.63 | **11** |
| **7** | 70.07 | 908.58 |  |  | 936.58 |  |  | **P** | 1119.51 | 1118.51 |  | **1161.55** | 1144.53 | 1143.54 | **10** |
| **8** | 86.10 | 1021.67 |  |  | 1049.66 |  |  | **L** | 1006.42 | **1005.43** |  | 1064.50 | 1047.47 | 1046.49 | **9** |
| **9** | 102.05 | 1150.71 |  | 1132.70 | 1178.71 |  | 1160.70 | **E** | 877.38 | 876.38 |  | **951.42** | 934.39 | 933.41 | **8** |
| **10** | 88.04 | 1265.74 |  | 1247.73 | 1293.73 |  | 1275.72 | **D** | 762.35 | 761.36 |  | **822.37** | 805.35 | 804.36 | **7** |
| **11** | 30.03 | 1322.76 |  | 1304.75 | 1350.75 |  | 1332.74 | **G** |  |  |  | **707.35** | 690.32 | 689.34 | **6** |
| **12** | 102.05 | 1451.80 |  | 1433.79 | 1479.80 |  | 1461.79 | **E** | 576.29 | 575.29 |  | 650.33 | 633.30 | 632.32 | **5** |
| **13** | 101.07 | 1579.86 | 1562.83 | 1561.85 | 1607.86 | 1590.83 | 1589.84 | **Q** | 448.23 | 447.24 |  | **521.28** | 504.26 |  | **4** |
| **14** | 120.08 | 1726.93 | 1709.90 | 1708.92 | 1754.92 | 1737.90 | 1736.91 | **F** | 301.16 |  |  | 393.22 | 376.20 |  | **3** |
| **15** | 44.05 | 1797.97 | 1780.94 | 1779.96 | 1825.96 | 1808.93 | 1807.95 | **A** | **230.12** |  |  | 246.16 | 229.13 |  | **2** |
| **16** | 129.11 |  |  |  |  |  |  | **R** | 74.02 | 73.03 |  | **175.12** | 158.09 |  | **1** |

| **Seq** | **ya** | **yb** | **Seq** | **ya** | **yb** | **Seq** | **ya** | **yb** |
| --- | --- | --- | --- | --- | --- | --- | --- | --- |
| **HA** | 181.11 | **209.10** | **HAF** | 328.18 | **356.17** | **HAFL** | 441.26 | 469.26 |
| **HAFLI** | 554.34 | 582.34 | **HAFLIP** | 651.40 | 679.39 | **AF** | 191.12 | 219.11 |
| **AFL** | 304.20 | 332.20 | **AFLI** | 417.29 | 445.28 | **AFLIP** | 514.34 | 542.33 |
| **AFLIPL** | 627.42 | 655.42 | **FL** | 233.16 | 261.16 | **FLI** | 346.25 | 374.24 |
| **FLIP** | 443.30 | 471.30 | **FLIPL** | 556.39 | 584.38 | **FLIPLE** | 685.43 | 713.42 |
| **LI** | 199.18 | 227.18 | **LIP** | 296.23 | 324.23 | **LIPL** | 409.32 | 437.31 |
| **LIPLE** | 538.36 | 566.35 | **LIPLED** | 653.39 | 681.38 | **IP** | 183.15 | 211.14 |
| **IPL** | 296.23 | 324.23 | **IPLE** | 425.28 | 453.27 | **IPLED** | 540.30 | 568.30 |
| **IPLEDG** | 597.32 | 625.32 | **PL** | 183.15 | 211.14 | **PLE** | 312.19 | 340.19 |
| **PLED** | 427.22 | 455.21 | **PLEDG** | 484.24 | 512.24 | **PLEDGE** | **613.28** | 641.28 |
| **LE** | 215.14 | 243.13 | **LED** | 330.17 | 358.16 | **LEDG** | 387.19 | 415.18 |
| **LEDGE** | 516.23 | 544.22 | **LEDGEQ** | 644.29 | 672.28 | **ED** | 217.08 | 245.08 |
| **EDG** | 274.10 | 302.10 | **EDGE** | 403.15 | 431.14 | **EDGEQ** | 531.20 | 559.20 |
| **EDGEQF** | 678.27 | 706.27 | **DG** | 145.06 | 173.06 | **DGE** | 274.10 | 302.10 |
| **DGEQ** | 402.16 | 430.16 | **DGEQF** | 549.23 | 577.23 | **DGEQFA** | 620.27 | 648.26 |
| **GE** | 159.08 | 187.07 | **GEQ** | 287.13 | 315.13 | **GEQF** | 434.20 | 462.20 |
| **GEQFA** | 505.24 | 533.24 | **EQ** | **230.11** | **258.11** | **EQF** | 377.18 | 405.18 |
| **EQFA** | 448.22 | 476.21 | **QF** | 248.14 | 276.13 | **QFA** | 319.18 | 347.17 |
| **FA** | 191.12 | 219.11 |  |  |  |  |  |  |

17. Tb11.42.0003

Match to: **Tb11.42.0003** Score: **81**

**t-complex protein 1, beta subunit, putative; Trypanosoma bruceichr 11Manual**

Nominal mass (M_r_): **64202**; Calculated pI value: **6.09**

NCBI BLAST search of [Tb11.42.0003](http://www.ncbi.nlm.nih.gov/blast/Blast.cgi?ALIGNMENTS=50&ALIGNMENT_VIEW=Pairwise&AUTO_FORMAT=Semiauto&CDD_SEARCH=on&CLIENT=web&COMPOSITION_BASED_STATISTICS=on&DATABASE=nr&DESCRIPTIONS=100&ENTREZ_QUERY=(none)&EXPECT=10&FILTER=L&FORMAT_BLOCK_ON_RESPAGE=None&FORMAT_OBJECT=Alignment&FORMAT_TYPE=HTML&GAPCOSTS=11+1&I_THRESH=0.001&LAYOUT=TwoWindows&MATRIX_NAME=BLOSUM62&NCBI_GI=on&PAGE=Proteins&PROGRAM=blastp&QUERY=MLFANQAQQVLRDGASEEKGERARLMNIMGAVSVADIVKTTLGPKGMDKILQGMDRSQGVRVTNDGATILKSLFMDNPAGKILIDMSKTQDDEVGDGTTSVTVLAGELLRNAEKLLDQSIHPQTIIEGYRLATQVAREALVASAEDHGSDEKLFYDDLIRIAKTTLSSKIITVEKEHFAKLCVDAVLRLKGSGNLEMINIMKKLGGTLRDSYLEPGFLLDKKIGVGQPRFLEDAKILVANTPMDTDKIKIFGAKVRVESVSQLAEVEASEKEKMKNKCMKIIKHNINCFINRQLIYNYPEEIFAQHGIMAIEHADFEGIERLAKALGADVLSQFDESQNVKYGFAKRIDEIMIGESTVIRFSGLPKGEACTIVVRGMSQHILDEAERSIHDALCVISQTIGETRTVLGAGCSEFVMARAVEERAKTTPGKKQLAMIAFANALRMIPSIIADNAGLDSNDLITRLQAEHYQGRNTFGIDVVKGDVADVKALGITESFKVKSSVLGYAAEAAEMILRVDDILRAVPRKRTQ&SERVICE=plain&SET_DEFAULTS.x=9&SET_DEFAULTS.y=5&SHOW_OVERVIEW=on&WORD_SIZE=3&END_OF_HTTPGET=Yes) against nr

Unformatted [sequence string](../../../../D:%5CProteomic%20data%5C2010-1-8%5Ccgi%5Cgetseq.pl%3FTBA927_IPI+Tb11%2E42%2E0003+seq) for pasting into other applications

Fixed modifications: MMTS (C),(N-TERM)_iTRAQ,Lysine(K)_iTRAQ

Variable modifications: Oxidation (M)

Cleavage by Trypsin: cuts C-term side of KR unless next residue is P

Sequence Coverage: **4%**

Matched peptides shown in **Bold Red**

**1** MLFANQAQQV LRDGASEEKG ERARLMNIMG AVSVADIVKT TLGPKGMDKI

**51** LQGMDRSQGV RVTNDGATIL KSLFMDNPAG KILIDMSKTQ DDEVGDGTTS

**101** VTVLAGELLR NAEKLLDQSI HPQTIIEGYR LATQVAREAL VASAEDHGSD

**151** EKLFYDDLIR IAKTTLSSKI ITVEKEHFAK LCVDAVLRLK GSGNLEMINI

**201** MKKLGGTLRD SYLEPGFLLD KKIGVGQPRF LEDAKILVAN TPMDTDKIKI

**251** FGAKVRVESV SQLAEVEASE KEKMKNKCMK IIKHNINCFI NRQLIYNYPE

**301** EIFAQHGIMA IEHADFEGIE RLAKALGADV LSQFDESQNV KYGFAKRIDE

**351** IMIGESTVIR FSGLPK**GEAC TIVVR**GMSQH ILDEAERSIH DALCVISQTI

**401** GETRTVLGAG CSEFVMARAV EERAKTTPGK KQLAMIAFAN ALRMIPSIIA

**451** DNAGLDSNDL ITRLQAEHYQ GRNTFGIDVV KGDVADVKAL GITESFKVK**S**

**501 SVLGYAAEAA EMILR**VDDIL RAVPRKRTQ

**Start - End Observed Mr(expt) Mr(calc) Delta Miss Sequence**

**367 - 375 1137.61 1136.60 1136.58 0.02 0 K.GEACTIVVR.G**  ([Ions score 14](../../../../D:%5CProteomic%20data%5C2010-1-8%5CZQ%5C1038.htm))

**500 - 515 1824.95 1823.94 1823.96 -0.02 0 K.SSVLGYAAEAAEMILR.V**  ([Ions score 81](../../../../D:%5CProteomic%20data%5C2010-1-8%5CZQ%5C1039.htm))

MS/MS Fragmentation of **SSVLGYAAEAAEMILR**
Found in **Tb11.42.0003**, t-complex protein 1, beta subunit, putative; Trypanosoma bruceichr 11Manual


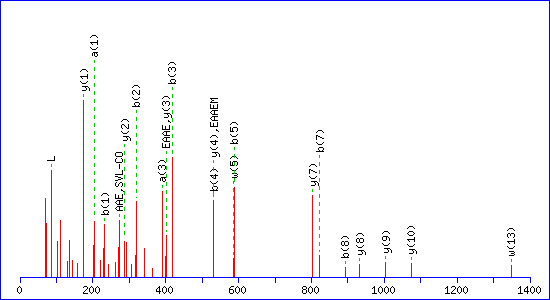


**MONOISOTOPIC mass of neutral peptide Mr(calc):** 1823.96

**Fixed modifications:** MMTS (C),(N-TERM)_iTRAQ,Lysine(K)_iTRAQ

**Ions Score:** 81 **Expect:** 9.4e-007

**Matches (Bold Red):** 29/262 fragment ions using 23 most intense peaks

| **#** | **Immon.** | **a** | **a^0^** | **b** | **b^0^** | **Seq.** | **v** | **w** | **w'** | **y** | **y*** | **y^0^** | **#** |
| --- | --- | --- | --- | --- | --- | --- | --- | --- | --- | --- | --- | --- | --- |
| **1** | 60.04 | **204.15** | 186.14 | **232.14** | 214.13 | **S** |  |  |  |  |  |  | **16** |
| **2** | 60.04 | 291.18 | 273.17 | **319.17** | 301.16 | **S** | 1561.80 | 1560.81 |  | 1593.83 | 1576.80 | 1575.82 | **15** |
| **3** | 72.08 | **390.25** | 372.24 | **418.24** | 400.23 | **V** | 1462.74 | 1475.76 |  | 1506.80 | 1489.77 | 1488.79 | **14** |
| **4** | **86.10** | 503.33 | 485.32 | **531.33** | 513.32 | **L** | 1349.65 | **1348.66** |  | 1407.73 | 1390.70 | 1389.72 | **13** |
| **5** | 30.03 | 560.35 | 542.34 | **588.35** | 570.34 | **G** |  |  |  | 1294.65 | 1277.62 | 1276.64 | **12** |
| **6** | 136.08 | 723.42 | 705.41 | 751.41 | 733.40 | **Y** | 1129.57 |  |  | 1237.62 | 1220.60 | 1219.61 | **11** |
| **7** | 44.05 | 794.45 | 776.44 | **822.45** | 804.44 | **A** | 1058.53 |  |  | **1074.56** | 1057.53 | 1056.55 | **10** |
| **8** | 44.05 | 865.49 | 847.48 | **893.49** | 875.47 | **A** | 987.49 |  |  | **1003.52** | 986.50 | 985.51 | **9** |
| **9** | 102.05 | 994.53 | 976.52 | 1022.53 | 1004.52 | **E** | 858.45 | 857.45 |  | **932.49** | 915.46 | 914.48 | **8** |
| **10** | 44.05 | 1065.57 | 1047.56 | 1093.56 | 1075.55 | **A** | 787.41 |  |  | **803.44** | 786.42 | 785.43 | **7** |
| **11** | 44.05 | 1136.61 | 1118.60 | 1164.60 | 1146.59 | **A** | 716.38 |  |  | 732.41 | 715.38 | 714.40 | **6** |
| **12** | 102.05 | 1265.65 | 1247.64 | 1293.64 | 1275.63 | **E** | 587.33 | **586.34** |  | 661.37 | 644.34 | 643.36 | **5** |
| **13** | 104.05 | 1396.69 | 1378.68 | 1424.69 | 1406.67 | **M** | 456.29 | 455.30 |  | **532.33** | 515.30 |  | **4** |
| **14** | **86.10** | 1509.77 | 1491.76 | 1537.77 | 1519.76 | **I** | 343.21 | 356.23 | 370.24 | **401.29** | 384.26 |  | **3** |
| **15** | **86.10** | 1622.86 | 1604.85 | 1650.85 | 1632.84 | **L** | 230.12 | 229.13 |  | **288.20** | 271.18 |  | **2** |
| **16** | 129.11 |  |  |  |  | **R** | 74.02 | 73.03 |  | **175.12** | 158.09 |  | **1** |

| **Seq** | **ya** | **yb** | **Seq** | **ya** | **yb** | **Seq** | **ya** | **yb** |
| --- | --- | --- | --- | --- | --- | --- | --- | --- |
| **SV** | 159.11 | 187.11 | **SVL** | **272.20** | 300.19 | **SVLG** | 329.22 | 357.21 |
| **SVLGY** | 492.28 | 520.28 | **SVLGYA** | 563.32 | 591.31 | **SVLGYAA** | 634.36 | 662.35 |
| **VL** | 185.16 | 213.16 | **VLG** | 242.19 | 270.18 | **VLGY** | 405.25 | 433.24 |
| **VLGYA** | 476.29 | 504.28 | **VLGYAA** | 547.32 | 575.32 | **VLGYAAE** | 676.37 | 704.36 |
| **LG** | 143.12 | 171.11 | **LGY** | 306.18 | 334.18 | **LGYA** | 377.22 | 405.21 |
| **LGYAA** | 448.26 | 476.25 | **LGYAAE** | 577.30 | 605.29 | **LGYAAEA** | 648.34 | 676.33 |
| **GY** | 193.10 | 221.09 | **GYA** | 264.13 | 292.13 | **GYAA** | 335.17 | 363.17 |
| **GYAAE** | 464.21 | 492.21 | **GYAAEA** | 535.25 | 563.25 | **GYAAEAA** | 606.29 | 634.28 |
| **YA** | 207.11 | 235.11 | **YAA** | 278.15 | 306.14 | **YAAE** | 407.19 | 435.19 |
| **YAAEA** | 478.23 | 506.22 | **YAAEAA** | 549.27 | 577.26 | **YAAEAAE** | 678.31 | 706.30 |
| **AA** | 115.09 | 143.08 | **AAE** | 244.13 | **272.12** | **AAEA** | 315.17 | 343.16 |
| **AAEAA** | 386.20 | 414.20 | **AAEAAE** | 515.25 | 543.24 | **AAEAAEM** | 646.29 | 674.28 |
| **AE** | 173.09 | 201.09 | **AEA** | 244.13 | **272.12** | **AEAA** | 315.17 | 343.16 |
| **AEAAE** | 444.21 | 472.20 | **AEAAEM** | 575.25 | 603.24 | **AEAAEMI** | 688.33 | 716.33 |
| **EA** | 173.09 | 201.09 | **EAA** | 244.13 | **272.12** | **EAAE** | 373.17 | **401.17** |
| **EAAEM** | 504.21 | **532.21** | **EAAEMI** | 617.30 | 645.29 | **AA** | 115.09 | 143.08 |
| **AAE** | 244.13 | **272.12** | **AAEM** | 375.17 | 403.16 | **AAEMI** | 488.25 | 516.25 |
| **AAEMIL** | 601.34 | 629.33 | **AE** | 173.09 | 201.09 | **AEM** | 304.13 | 332.13 |
| **AEMI** | 417.22 | 445.21 | **AEMIL** | 530.30 | 558.30 | **EM** | 233.10 | 261.09 |
| **EMI** | 346.18 | 374.17 | **EMIL** | 459.26 | 487.26 | **MI** | 217.14 | 245.13 |
| **MIL** | 330.22 | 358.22 | **IL** | 199.18 | 227.18 |  |  |  |

18. Tb927.3.1900

Match to: **Tb927.3.1900** Score: **81**

**hypothetical protein, conserved; Trypanosoma bruceichr 3Manual**

Nominal mass (M_r_): **108944**; Calculated pI value: **4.76**

NCBI BLAST search of [Tb927.3.1900](http://www.ncbi.nlm.nih.gov/blast/Blast.cgi?ALIGNMENTS=50&ALIGNMENT_VIEW=Pairwise&AUTO_FORMAT=Semiauto&CDD_SEARCH=on&CLIENT=web&COMPOSITION_BASED_STATISTICS=on&DATABASE=nr&DESCRIPTIONS=100&ENTREZ_QUERY=(none)&EXPECT=10&FILTER=L&FORMAT_BLOCK_ON_RESPAGE=None&FORMAT_OBJECT=Alignment&FORMAT_TYPE=HTML&GAPCOSTS=11+1&I_THRESH=0.001&LAYOUT=TwoWindows&MATRIX_NAME=BLOSUM62&NCBI_GI=on&PAGE=Proteins&PROGRAM=blastp&QUERY=MSKSLPPLESSSRGGEAARVGVYASHGPAATNALCVVTPRSTVNSNLRPQSAQRFLETEFTLGPLQDQSLPPYNDLEDPYLAPYWARREMLIRETAERREEMRRQKRLEQHRREVARRRFEERRQRELEELACRTGFATQRRVEAEELTSPKETTSGRKEVAKKRQRTVSSRATGSKKRAVATVPKPPKQKRPDTVGQRGGRPGACSSQGKGKTSSPPKNILLKTIRETLNSDSSRPATPQKGKGAASPVGGRKSGGGLTESSKHGAVTEDEAPISSSVAEDSATGKGSTSSVAQPSEAKRESRPSTASPQPASTEQSTSKEILKDGGNSRPASAARPSSASASASQKAMQQDTTDAEDGKDTQSDRSRPSSRKSERSGREGREKTDDEPTADTTKNDYGDEFEASESGKKDPDTEDQKDTESERSRSSSKKPEELTKDDADKTDDEPTADTTKNDYGDEFEASESGKKDPDTEDQKDTESERSRSSSKKPEDDEQKDEYTEELDKSQTSEKKDTEAEDQKDTESERSRSSSKKPEDDEQKDEYTEELDKSQTSEKKDTEAEDQKDTESERSRSSSKKPEDDEQKDEYTEELDKSQTSEKKDTESEQSRSSSKKPEDDEQKDEYTEELDKSQTSEKKDTEAEDQKDTESERSRSSSKKPEDDEQKDEYTEELDKSQTSEKKDTEAEDQKDTESERSRSSSKKPEDDEQKDEYTEELDKSQTSEKKDTESEQSRSSSKKSESSAKLEGDKFEYGDELERSPSSEKGEAVQQVVEDEHVNDQDVDELDSDAVKSEAVDEASGADAAKDSADGASSKESSSKRTSRSSSSVSKGRKNASSSSSSIGSEELSGNDVARASGTGEDEIEDEA&SERVICE=plain&SET_DEFAULTS.x=9&SET_DEFAULTS.y=5&SHOW_OVERVIEW=on&WORD_SIZE=3&END_OF_HTTPGET=Yes) against nr

Unformatted [sequence string](../../../../D:%5CProteomic%20data%5C2010-1-8%5Ccgi%5Cgetseq.pl%3FTBA927_IPI+Tb927%2E3%2E1900+seq) for pasting into other applications

Fixed modifications: MMTS (C),(N-TERM)_iTRAQ,Lysine(K)_iTRAQ

Variable modifications: Oxidation (M)

Cleavage by Trypsin: cuts C-term side of KR unless next residue is P

Sequence Coverage: **4%**

Matched peptides shown in **Bold Red**

**1** MSKSLPPLES SSRGGEAARV GVYASHGPAA TNALCVVTPR STVNSNLRPQ

**51** SAQRFLETEF TLGPLQDQSL PPYNDLEDPY LAPYWARREM LIRETAERRE

**101** EMRRQKRLEQ HRREVARRRF EERRQR**ELEE LACRTGFATQ R**RVEAEELTS

**151** PKETTSGRKE VAKKRQRTVS SRATGSKKRA VATVPKPPKQ KRPDTVGQRG

**201** GRPGACSSQG KGKTSSPPKN ILLKTIR**ETL NSDSSRPATP QKGKGAASPV**

**251 GGR**KSGGGLT ESSKHGAVTE DEAPISSSVA EDSATGKGST SSVAQPSEAK

**301** RESRPSTASP QPASTEQSTS KEILKDGGNS RPASAARPSS ASASASQKAM

**351** QQDTTDAEDG KDTQSDRSRP SSRKSERSGR EGREKTDDEP TADTTKNDYG

**401** DEFEASESGK KDPDTEDQKD TESERSRSSS KKPEELTKDD ADKTDDEPTA

**451** DTTKNDYGDE FEASESGKKD PDTEDQKDTE SERSRSSSKK PEDDEQKDEY

**501** TEELDKSQTS EKKDTEAEDQ KDTESERSRS SSKKPEDDEQ KDEYTEELDK

**551** SQTSEKKDTE AEDQKDTESE RSRSSSKKPE DDEQKDEYTE ELDKSQTSEK

**601** KDTESEQSRS SSKKPEDDEQ KDEYTEELDK SQTSEKKDTE AEDQKDTESE

**651** RSRSSSKKPE DDEQKDEYTE ELDKSQTSEK KDTEAEDQKD TESERSRSSS

**701** KKPEDDEQKD EYTEELDKSQ TSEKKDTESE QSRSSSKKSE SSAKLEGDKF

**751** EYGDELERSP SSEKGEAVQQ VVEDEHVNDQ DVDELDSDAV KSEAVDEASG

**801** ADAAKDSADG ASSKESSSKR TSRSSSSVSK GRKNASSSSS SIGSEELSGN

**851** DVARASGTGE DEIEDEA

**Start - End Observed Mr(expt) Mr(calc) Delta Miss Sequence**

**127 - 141 1914.05 1913.04 1912.93 0.12 1 R.ELEELACRTGFATQR.R**  ([Ions score 4](../../../../D:%5CProteomic%20data%5C2010-1-8%5CZQ%5C1042.htm))

**228 - 242 1918.99 1917.98 1917.91 0.07 0 R.ETLNSDSSRPATPQK.G**  ([Ions score 81](../../../../D:%5CProteomic%20data%5C2010-1-8%5CZQ%5C1043.htm))

**243 - 253 1244.60 1243.59 1243.63 -0.05 1 K.GKGAASPVGGR.K**  ([Ions score 11](../../../../D:%5CProteomic%20data%5C2010-1-8%5CZQ%5C1041.htm))

MS/MS Fragmentation of **ETLNSDSSRPATPQK**
Found in **Tb927.3.1900**, hypothetical protein, conserved; Trypanosoma bruceichr 3Manual


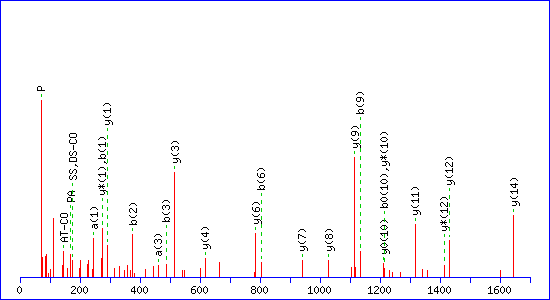


**MONOISOTOPIC mass of neutral peptide Mr(calc):** 1917.91

**Fixed modifications:** MMTS (C),(N-TERM)_iTRAQ,Lysine(K)_iTRAQ

**Ions Score:** 81 **Expect:** 9.1e-007

**Matches (Bold Red):** 29/260 fragment ions using 28 most intense peaks

| **#** | **Immon.** | **a** | **a*** | **a^0^** | **b** | **b*** | **b^0^** | **d** | **d'** | **Seq.** | **v** | **w** | **w'** | **y** | **y*** | **y^0^** | **#** |
| --- | --- | --- | --- | --- | --- | --- | --- | --- | --- | --- | --- | --- | --- | --- | --- | --- | --- |
| **1** | 102.05 | **246.16** |  | 228.15 | **274.15** |  | 256.14 |  |  | **E** |  |  |  |  |  |  | **15** |
| **2** | 74.06 | 347.21 |  | 329.19 | **375.20** |  | 357.19 |  |  | **T** | 1599.73 | 1612.75 | 1614.73 | **1645.77** | 1628.75 | 1627.76 | **14** |
| **3** | 86.10 | **460.29** |  | 442.28 | **488.28** |  | 470.27 |  |  | **L** | 1486.65 | 1485.65 |  | 1544.73 | 1527.70 | 1526.71 | **13** |
| **4** | 87.06 | 574.33 | 557.31 | 556.32 | 602.33 | 585.30 | 584.32 |  |  | **N** | 1372.60 | 1371.61 |  | **1431.64** | **1414.61** | 1413.63 | **12** |
| **5** | 60.04 | 661.36 | 644.34 | 643.35 | 689.36 | 672.33 | 671.35 |  |  | **S** | 1285.57 | 1284.58 |  | **1317.60** | 1300.57 | 1299.59 | **11** |
| **6** | 88.04 | 776.39 | 759.36 | 758.38 | **804.39** | 787.36 | 786.38 |  |  | **D** | 1170.55 | 1169.55 |  | 1230.57 | **1213.54** | **1212.56** | **10** |
| **7** | 60.04 | 863.42 | 846.40 | 845.41 | 891.42 | 874.39 | 873.41 |  |  | **S** | 1083.51 | 1082.52 |  | **1115.54** | 1098.51 | 1097.53 | **9** |
| **8** | 60.04 | 950.46 | 933.43 | 932.44 | 978.45 | 961.42 | 960.44 |  |  | **S** | 996.48 | 995.49 |  | **1028.51** | 1011.48 | 1010.50 | **8** |
| **9** | 129.11 | 1106.56 | 1089.53 | 1088.55 | **1134.55** | 1117.52 | 1116.54 | 1021.49 |  | **R** | 840.38 | 839.38 |  | **941.48** | 924.45 | 923.46 | **7** |
| **10** | **70.07** | 1203.61 | 1186.58 | 1185.60 | 1231.60 | 1214.58 | **1213.59** | 1177.59 |  | **P** |  |  |  | **785.37** | 768.35 | 767.36 | **6** |
| **11** | 44.05 | 1274.65 | 1257.62 | 1256.64 | 1302.64 | 1285.61 | 1284.63 |  |  | **A** |  |  |  | 688.32 | 671.29 | 670.31 | **5** |
| **12** | 74.06 | 1375.69 | 1358.67 | 1357.68 | 1403.69 | 1386.66 | 1385.68 | 1359.70 | 1361.68 | **T** |  |  |  | **617.28** | 600.26 | 599.27 | **4** |
| **13** | **70.07** | 1472.75 | 1455.72 | 1454.74 | 1500.74 | 1483.71 | 1482.73 | 1446.73 |  | **P** |  |  |  | **516.24** | 499.21 |  | **3** |
| **14** | 101.07 | 1600.81 | 1583.78 | 1582.79 | 1628.80 | 1611.77 | 1610.79 | 1543.78 |  | **Q** |  |  |  | 419.18 | 402.16 |  | **2** |
| **15** | 245.12 |  |  |  |  |  |  |  |  | **K** |  |  |  | **291.13** | **274.10** |  | **1** |

| **Seq** | **ya** | **yb** | **Seq** | **ya** | **yb** | **Seq** | **ya** | **yb** |
| --- | --- | --- | --- | --- | --- | --- | --- | --- |
| **TL** | 187.14 | 215.14 | **TLN** | 301.19 | 329.18 | **TLNS** | 388.22 | 416.21 |
| **TLNSD** | 503.25 | 531.24 | **TLNSDS** | 590.28 | 618.27 | **TLNSDSS** | 677.31 | 705.30 |
| **LN** | 200.14 | 228.13 | **LNS** | 287.17 | 315.17 | **LNSD** | 402.20 | 430.19 |
| **LNSDS** | 489.23 | 517.23 | **LNSDSS** | 576.26 | 604.26 | **NS** | 174.09 | 202.08 |
| **NSD** | 289.11 | 317.11 | **NSDS** | 376.15 | 404.14 | **NSDSS** | 463.18 | 491.17 |
| **NSDSSR** | 619.28 | 647.27 | **SD** | **175.07** | 203.07 | **SDS** | 262.10 | 290.10 |
| **SDSS** | 349.14 | 377.13 | **SDSSR** | 505.24 | 533.23 | **SDSSRP** | 602.29 | 630.28 |
| **SDSSRPA** | 673.33 | 701.32 | **DS** | **175.07** | 203.07 | **DSS** | 262.10 | 290.10 |
| **DSSR** | 418.20 | 446.20 | **DSSRP** | 515.26 | 543.25 | **DSSRPA** | 586.29 | 614.29 |
| **DSSRPAT** | 687.34 | 715.34 | **SS** | 147.08 | **175.07** | **SSR** | 303.18 | 331.17 |
| **SSRP** | 400.23 | 428.23 | **SSRPA** | 471.27 | 499.26 | **SSRPAT** | 572.32 | 600.31 |
| **SSRPATP** | 669.37 | 697.36 | **SR** | 216.15 | 244.14 | **SRP** | 313.20 | 341.19 |
| **SRPA** | 384.24 | 412.23 | **SRPAT** | 485.28 | 513.28 | **SRPATP** | 582.34 | 610.33 |
| **RP** | 226.17 | 254.16 | **RPA** | 297.20 | 325.20 | **RPAT** | 398.25 | 426.25 |
| **RPATP** | 495.30 | 523.30 | **RPATPQ** | 623.36 | 651.36 | **PA** | 141.10 | **169.10** |
| **PAT** | 242.15 | 270.14 | **PATP** | 339.20 | 367.20 | **PATPQ** | 467.26 | 495.26 |
| **AT** | **145.10** | 173.09 | **ATP** | 242.15 | 270.14 | **ATPQ** | 370.21 | 398.20 |
| **TP** | 171.11 | 199.11 | **TPQ** | 299.17 | 327.17 | **PQ** | 198.12 | 226.12 |

19. Tb09.211.2540

Match to: **Tb09.211.2540** Score: **77**

**calmodulin-like protein, putative; Trypanosoma bruceichr 9Manual**

Nominal mass (M_r_): **17624**; Calculated pI value: **4.78**

NCBI BLAST search of [Tb09.211.2540](http://www.ncbi.nlm.nih.gov/blast/Blast.cgi?ALIGNMENTS=50&ALIGNMENT_VIEW=Pairwise&AUTO_FORMAT=Semiauto&CDD_SEARCH=on&CLIENT=web&COMPOSITION_BASED_STATISTICS=on&DATABASE=nr&DESCRIPTIONS=100&ENTREZ_QUERY=(none)&EXPECT=10&FILTER=L&FORMAT_BLOCK_ON_RESPAGE=None&FORMAT_OBJECT=Alignment&FORMAT_TYPE=HTML&GAPCOSTS=11+1&I_THRESH=0.001&LAYOUT=TwoWindows&MATRIX_NAME=BLOSUM62&NCBI_GI=on&PAGE=Proteins&PROGRAM=blastp&QUERY=MSRASIMKEAFELLQRDGKIPKASIPTALRAAGMNPSEEKLKEIMATAVDIDMAGYESLVTEHYDKTDTVEAVKEAFRVFDKDHNGTVSVAEFRHIMTTMGEKYTEEEFCDLIQGFDANGVIPYEKFVEKMLAPFTEHESA&SERVICE=plain&SET_DEFAULTS.x=9&SET_DEFAULTS.y=5&SHOW_OVERVIEW=on&WORD_SIZE=3&END_OF_HTTPGET=Yes) against nr

Unformatted [sequence string](../../../../D:%5CProteomic%20data%5C2010-1-8%5Ccgi%5Cgetseq.pl%3FTBA927_IPI+Tb09%2E211%2E2540+seq) for pasting into other applications

Fixed modifications: MMTS (C),(N-TERM)_iTRAQ,Lysine(K)_iTRAQ

Variable modifications: Oxidation (M)

Cleavage by Trypsin: cuts C-term side of KR unless next residue is P

Sequence Coverage: **12%**

Matched peptides shown in **Bold Red**

**1** MSRASIMK**EA FELLQR**DGKI PKASIPTALR **AAGMNPSEEK** LKEIMATAVD

**51** IDMAGYESLV TEHYDKTDTV EAVKEAFRVF DKDHNGTVSV AEFRHIMTTM

**101** GEKYTEEEFC DLIQGFDANG VIPYEKFVEK MLAPFTEHES A

**Start - End Observed Mr(expt) Mr(calc) Delta Miss Sequence**

**9 - 16 1149.61 1148.61 1148.63 -0.02 0 K.EAFELLQR.D**  ([Ions score 9](../../../../D:%5CProteomic%20data%5C2010-1-8%5CZQ%5C1071.htm))

**31 - 40 1321.67 1320.66 1320.57 0.09 0 R.AAGMNPSEEK.L**  ([Ions score 77](../../../../D:%5CProteomic%20data%5C2010-1-8%5CZQ%5C1072.htm))

MS/MS Fragmentation of **AAGMNPSEEK**
Found in **Tb09.211.2540**, calmodulin-like protein, putative; Trypanosoma bruceichr 9Manual


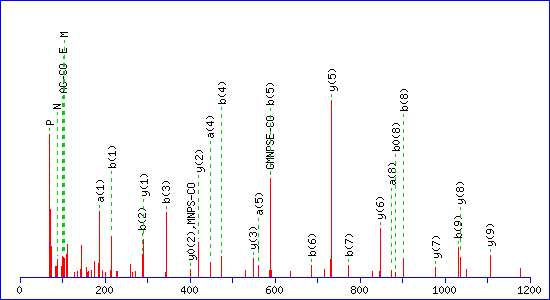


**MONOISOTOPIC mass of neutral peptide Mr(calc):** 1320.57

**Fixed modifications:** MMTS (C),(N-TERM)_iTRAQ,Lysine(K)_iTRAQ

**Ions Score:** 77 **Expect:** 3.2e-006

**Matches (Bold Red):** 31/122 fragment ions using 41 most intense peaks

| **#** | **Immon.** | **a** | **a*** | **a^0^** | **b** | **b*** | **b^0^** | **Seq.** | **y** | **y*** | **y^0^** | **#** |
| --- | --- | --- | --- | --- | --- | --- | --- | --- | --- | --- | --- | --- |
| **1** | 44.05 | **188.15** |  |  | **216.15** |  |  | **A** |  |  |  | **10** |
| **2** | 44.05 | 259.19 |  |  | **287.18** |  |  | **A** | **1106.44** | 1089.41 | 1088.43 | **9** |
| **3** | 30.03 | 316.21 |  |  | **344.21** |  |  | **G** | **1035.40** | 1018.37 | 1017.39 | **8** |
| **4** | **104.05** | **447.25** |  |  | **475.25** |  |  | **M** | **978.38** | 961.35 | 960.37 | **7** |
| **5** | **87.06** | **561.29** | 544.27 |  | **589.29** | 572.26 |  | **N** | **847.34** | 830.31 | 829.33 | **6** |
| **6** | **70.07** | 658.35 | 641.32 |  | **686.34** | 669.32 |  | **P** | **733.30** | 716.27 | 715.28 | **5** |
| **7** | 60.04 | 745.38 | 728.35 | 727.37 | **773.37** | 756.35 | 755.36 | **S** | 636.24 | 619.22 | 618.23 | **4** |
| **8** | **102.05** | **874.42** | 857.39 | 856.41 | **902.42** | 885.39 | **884.41** | **E** | **549.21** | 532.18 | 531.20 | **3** |
| **9** | **102.05** | 1003.46 | 986.44 | 985.45 | **1031.46** | 1014.43 | 1013.45 | **E** | **420.17** | 403.14 | **402.16** | **2** |
| **10** | 245.12 |  |  |  |  |  |  | **K** | **291.13** | 274.10 |  | **1** |

| **Seq** | **ya** | **yb** | **Seq** | **ya** | **yb** | **Seq** | **ya** | **yb** |
| --- | --- | --- | --- | --- | --- | --- | --- | --- |
| **AG** | **101.07** | 129.07 | **AGM** | 232.11 | 260.11 | **AGMN** | 346.15 | 374.15 |
| **AGMNP** | 443.21 | 471.20 | **AGMNPS** | 530.24 | 558.23 | **AGMNPSE** | 659.28 | 687.28 |
| **GM** | 161.07 | 189.07 | **GMN** | 275.12 | 303.11 | **GMNP** | 372.17 | 400.16 |
| **GMNPS** | 459.20 | 487.20 | **GMNPSE** | **588.24** | 616.24 | **MN** | 218.10 | 246.09 |
| **MNP** | 315.15 | 343.14 | **MNPS** | **402.18** | 430.18 | **MNPSE** | 531.22 | 559.22 |
| **MNPSEE** | 660.27 | 688.26 | **NP** | 184.11 | 212.10 | **NPS** | 271.14 | 299.13 |
| **NPSE** | 400.18 | 428.18 | **NPSEE** | 529.23 | 557.22 | **PS** | 157.10 | 185.09 |
| **PSE** | 286.14 | 314.13 | **PSEE** | 415.18 | 443.18 | **SE** | 189.09 | 217.08 |
| **SEE** | 318.13 | 346.12 | **EE** | 231.10 | 259.09 |  |  |  |

20. Tb09.160.1660

Match to: **Tb09.160.1660** Score: **76**

**3C4.305hypothetical protein, conserved; Trypanosoma bruceichr 9Manual**

Nominal mass (M_r_): **9887**; Calculated pI value: **8.93**

NCBI BLAST search of [Tb09.160.1660](http://www.ncbi.nlm.nih.gov/blast/Blast.cgi?ALIGNMENTS=50&ALIGNMENT_VIEW=Pairwise&AUTO_FORMAT=Semiauto&CDD_SEARCH=on&CLIENT=web&COMPOSITION_BASED_STATISTICS=on&DATABASE=nr&DESCRIPTIONS=100&ENTREZ_QUERY=(none)&EXPECT=10&FILTER=L&FORMAT_BLOCK_ON_RESPAGE=None&FORMAT_OBJECT=Alignment&FORMAT_TYPE=HTML&GAPCOSTS=11+1&I_THRESH=0.001&LAYOUT=TwoWindows&MATRIX_NAME=BLOSUM62&NCBI_GI=on&PAGE=Proteins&PROGRAM=blastp&QUERY=MSFGLEYSEGQRDYLERIGVGPLLEDFVADAVREKPNDVYEFLRQWATARCAKATAATHEKSARVIQRAFRNYRSRLTATA&SERVICE=plain&SET_DEFAULTS.x=9&SET_DEFAULTS.y=5&SHOW_OVERVIEW=on&WORD_SIZE=3&END_OF_HTTPGET=Yes) against nr

Unformatted [sequence string](../../../../D:%5CProteomic%20data%5C2010-1-8%5Ccgi%5Cgetseq.pl%3FTBA927_IPI+Tb09%2E160%2E1660+seq) for pasting into other applications

Fixed modifications: MMTS (C),(N-TERM)_iTRAQ,Lysine(K)_iTRAQ

Variable modifications: Oxidation (M)

Cleavage by Trypsin: cuts C-term side of KR unless next residue is P

Sequence Coverage: **19%**

Matched peptides shown in **Bold Red**

**1** MSFGLEYSEG QRDYLER**IGV GPLLEDFVAD AVR**EKPNDVY EFLRQWATAR

**51** CAKATAATHE KSARVIQRAF RNYRSRLTAT A

MS/MS Fragmentation of **IGVGPLLEDFVADAVR**
Found in **Tb09.160.1660**, 3C4.305hypothetical protein, conserved; Trypanosoma bruceichr 9Manual


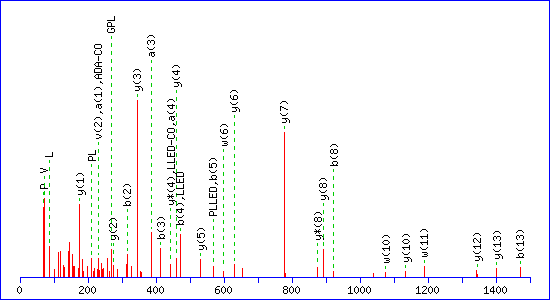


**MONOISOTOPIC mass of neutral peptide Mr(calc):** 1814.01

**Fixed modifications:** MMTS (C),(N-TERM)_iTRAQ,Lysine(K)_iTRAQ

**Ions Score:** 76 **Expect:** 2.2e-006

**Matches (Bold Red):** 39/239 fragment ions using 36 most intense peaks

| **#** | **Immon.** | **a** | **a^0^** | **b** | **b^0^** | **Seq.** | **v** | **w** | **y** | **y*** | **y^0^** | **#** |
| --- | --- | --- | --- | --- | --- | --- | --- | --- | --- | --- | --- | --- |
| **1** | **86.10** | **230.20** |  | 258.19 |  | **I** |  |  |  |  |  | **16** |
| **2** | 30.03 | 287.22 |  | **315.22** |  | **G** |  |  | 1557.83 | 1540.80 | 1539.82 | **15** |
| **3** | **72.08** | **386.29** |  | **414.28** |  | **V** | 1456.74 | 1469.76 | 1500.81 | 1483.78 | 1482.80 | **14** |
| **4** | 30.03 | **443.31** |  | **471.31** |  | **G** |  |  | **1401.74** | 1384.71 | 1383.73 | **13** |
| **5** | **70.07** | 540.36 |  | **568.36** |  | **P** | 1302.67 | 1301.67 | **1344.72** | 1327.69 | 1326.71 | **12** |
| **6** | **86.10** | 653.45 |  | 681.44 |  | **L** | 1189.58 | **1188.59** | 1247.66 | 1230.64 | 1229.65 | **11** |
| **7** | **86.10** | 766.53 |  | 794.53 |  | **L** | 1076.50 | **1075.51** | **1134.58** | 1117.55 | 1116.57 | **10** |
| **8** | 102.05 | 895.57 | 877.56 | **923.57** | 905.56 | **E** | 947.46 | 946.46 | 1021.49 | 1004.47 | 1003.48 | **9** |
| **9** | 88.04 | 1010.60 | 992.59 | 1038.60 | 1020.58 | **D** | 832.43 | 831.44 | **892.45** | **875.43** | 874.44 | **8** |
| **10** | 120.08 | 1157.67 | 1139.66 | 1185.66 | 1167.65 | **F** | 685.36 |  | **777.43** | 760.40 | 759.41 | **7** |
| **11** | **72.08** | 1256.74 | 1238.73 | 1284.73 | 1266.72 | **V** | 586.29 | **599.31** | **630.36** | 613.33 | 612.35 | **6** |
| **12** | 44.05 | 1327.77 | 1309.76 | 1355.77 | 1337.76 | **A** | 515.26 |  | **531.29** | 514.26 | 513.28 | **5** |
| **13** | 88.04 | 1442.80 | 1424.79 | **1470.80** | 1452.79 | **D** | 400.23 | 399.24 | **460.25** | **443.22** | 442.24 | **4** |
| **14** | 44.05 | 1513.84 | 1495.83 | 1541.83 | 1523.82 | **A** | 329.19 |  | **345.22** | 328.20 |  | **3** |
| **15** | **72.08** | 1612.91 | 1594.90 | 1640.90 | 1622.89 | **V** | **230.12** | 243.15 | **274.19** | 257.16 |  | **2** |
| **16** | 129.11 |  |  |  |  | **R** | 74.02 | 73.03 | **175.12** | 158.09 |  | **1** |

| **Seq** | **ya** | **yb** | **Seq** | **ya** | **yb** | **Seq** | **ya** | **yb** |
| --- | --- | --- | --- | --- | --- | --- | --- | --- |
| **GV** | 129.10 | 157.10 | **GVG** | 186.12 | 214.12 | **GVGP** | 283.18 | 311.17 |
| **GVGPL** | 396.26 | 424.26 | **GVGPLL** | 509.34 | 537.34 | **GVGPLLE** | 638.39 | 666.38 |
| **VG** | 129.10 | 157.10 | **VGP** | 226.15 | 254.15 | **VGPL** | 339.24 | 367.23 |
| **VGPLL** | 452.32 | 480.32 | **VGPLLE** | 581.37 | 609.36 | **VGPLLED** | 696.39 | 724.39 |
| **GP** | 127.09 | 155.08 | **GPL** | 240.17 | **268.17** | **GPLL** | 353.25 | 381.25 |
| **GPLLE** | 482.30 | 510.29 | **GPLLED** | 597.32 | 625.32 | **PL** | 183.15 | **211.14** |
| **PLL** | 296.23 | 324.23 | **PLLE** | 425.28 | 453.27 | **PLLED** | 540.30 | **568.30** |
| **PLLEDF** | 687.37 | 715.37 | **LL** | 199.18 | 227.18 | **LLE** | 328.22 | 356.22 |
| **LLED** | **443.25** | **471.24** | **LLEDF** | 590.32 | 618.31 | **LLEDFV** | 689.39 | 717.38 |
| **LE** | 215.14 | 243.13 | **LED** | 330.17 | 358.16 | **LEDF** | 477.23 | 505.23 |
| **LEDFV** | 576.30 | 604.30 | **LEDFVA** | 647.34 | 675.33 | **ED** | 217.08 | 245.08 |
| **EDF** | 364.15 | 392.15 | **EDFV** | 463.22 | 491.21 | **EDFVA** | 534.26 | 562.25 |
| **EDFVAD** | 649.28 | 677.28 | **DF** | 235.11 | 263.10 | **DFV** | 334.18 | 362.17 |
| **DFVA** | 405.21 | 433.21 | **DFVAD** | 520.24 | 548.24 | **DFVADA** | 591.28 | 619.27 |
| **DFVADAV** | 690.35 | 718.34 | **FV** | 219.15 | 247.14 | **FVA** | 290.19 | 318.18 |
| **FVAD** | 405.21 | 433.21 | **FVADA** | 476.25 | 504.25 | **FVADAV** | 575.32 | 603.31 |
| **VA** | 143.12 | 171.11 | **VAD** | 258.14 | 286.14 | **VADA** | 329.18 | 357.18 |
| **VADAV** | 428.25 | 456.25 | **AD** | 159.08 | 187.07 | **ADA** | **230.11** | 258.11 |
| **ADAV** | 329.18 | 357.18 | **DA** | 159.08 | 187.07 | **DAV** | 258.14 | 286.14 |
| **AV** | 143.12 | 171.11 |  |  |  |  |  |  |

21. Tb927.7.6970

Match to: **Tb927.7.6970** Score: **73**

**paraflagellar rod protein, putative; Trypanosoma bruceichr 7Manual**

Nominal mass (M_r_): **84880**; Calculated pI value: **6.07**

NCBI BLAST search of [Tb927.7.6970](http://www.ncbi.nlm.nih.gov/blast/Blast.cgi?ALIGNMENTS=50&ALIGNMENT_VIEW=Pairwise&AUTO_FORMAT=Semiauto&CDD_SEARCH=on&CLIENT=web&COMPOSITION_BASED_STATISTICS=on&DATABASE=nr&DESCRIPTIONS=100&ENTREZ_QUERY=(none)&EXPECT=10&FILTER=L&FORMAT_BLOCK_ON_RESPAGE=None&FORMAT_OBJECT=Alignment&FORMAT_TYPE=HTML&GAPCOSTS=11+1&I_THRESH=0.001&LAYOUT=TwoWindows&MATRIX_NAME=BLOSUM62&NCBI_GI=on&PAGE=Proteins&PROGRAM=blastp&QUERY=MTKKSGHEGGTSLNGNGSSSTTPSNTVPTSLTFAGSVAATRKTFLQREAVLDACAVTTNNVALAQARRTALRDSGARIVKLFEELAQTYAANMKTATASQEGSGTGTPSAKDGPITVEQLNMINCMAEAYAESFRKECGLELALGDMVMSDAPANTSKKENDNSGSRNHNSASKGGDSTGGEAGGTAAGPAQEAIRRRIAPLQRALASAKELRNIPTYNKVLSVAQRGNVGANSLTTLVKNLTETLEESLQELEGGYGSCSNSANDVAEWACWVQPLVRLTNECLQTKPLEKIMEGPRERLRRVAFDVEEKQREQEDAVTDGDMVRSEQLYFEKTALLESMRPIYDELEAAIEESKRASVDEPSKELRSLVKELSTKQAPKVLDREKQVRRRGKTDLERLLARREEIMAARTKQTSTFKVYLAEWDKMFRHNEQQQENCLRAIEELEQRLKYLSEERAILVEDRLEVAAQEQQRSEDAASFMLFAAQHEQKLRKTIENLDQSLSCGERVLEVVRSSYHHLGKYLQDMVQREADEQLLEVRKERLSHFRGLYLTLGELKYKKERHLEELDKRIEYYHVQQELAMDTFNPKAKEFSKAKKDLLEVKETMQQQIDLIGQKAVKQLEDFKPTEQLLLASGVKFVHPVKELEEMNQRRTQKLLEYHNLMSTIGDGRSREEAEANGKVAPKTSS&SERVICE=plain&SET_DEFAULTS.x=9&SET_DEFAULTS.y=5&SHOW_OVERVIEW=on&WORD_SIZE=3&END_OF_HTTPGET=Yes) against nr

Unformatted [sequence string](../../../../D:%5CProteomic%20data%5C2010-1-8%5Ccgi%5Cgetseq.pl%3FTBA927_IPI+Tb927%2E7%2E6970+seq) for pasting into other applications

Fixed modifications: MMTS (C),(N-TERM)_iTRAQ,Lysine(K)_iTRAQ

Variable modifications: Oxidation (M)

Cleavage by Trypsin: cuts C-term side of KR unless next residue is P

Sequence Coverage: **3%**

Matched peptides shown in **Bold Red**

**1** MTKKSGHEGG TSLNGNGSSS TTPSNTVPTS LTFAGSVAAT RKTFLQREAV

**51** LDACAVTTNN VALAQARRTA LRDSGARIVK LFEELAQTYA ANMKTATASQ

**101** EGSGTGTPSA KDGPITVEQL NMINCMAEAY AESFRKECGL ELALGDMVMS

**151** DAPANTSKKE NDNSGSRNHN SASK**GGDSTG GEAGGTAAGP AQEAIR**RRIA

**201** PLQRALASAK ELRNIPTYNK VLSVAQRGNV GANSLTTLVK NLTETLEESL

**251** QELEGGYGSC SNSANDVAEW ACWVQPLVRL TNECLQTKPL EKIMEGPRER

**301** LRRVAFDVEE KQREQEDAVT DGDMVRSEQL YFEKTALLES MRPIYDELEA

**351** AIEESKRASV DEPSKELRSL VKELSTKQAP KVLDREKQVR RRGKTDLERL

**401** LARREEIMAA RTKQTSTFKV YLAEWDKMFR HNEQQQENCL RAIEELEQRL

**451** KYLSEERAIL VEDRLEVAAQ EQQRSEDAAS FMLFAAQHEQ KLRKTIENLD

**501** QSLSCGERVL EVVRSSYHHL GKYLQDMVQR EADEQLLEVR KERLSHFRGL

**551** YLTLGELKYK KERHLEELDK RIEYYHVQQE LAMDTFNPKA KEFSKAKKDL

**601** LEVKETMQQQ IDLIGQKAVK QLEDFKPTEQ LLLASGVKFV HPVKELEEMN

**651** QRRTQKLLEY HNLMSTIGDG RSREEAEANG KVAPKTSS

MS/MS Fragmentation of **GGDSTGGEAGGTAAGPAQEAIR**
Found in **Tb927.7.6970**, paraflagellar rod protein, putative; Trypanosoma bruceichr 7Manual


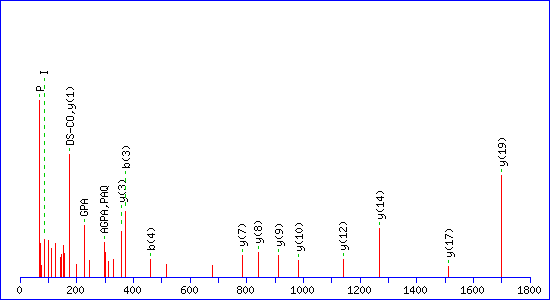


**MONOISOTOPIC mass of neutral peptide Mr(calc):** 2072.98

**Fixed modifications:** MMTS (C),(N-TERM)_iTRAQ,Lysine(K)_iTRAQ

**Ions Score:** 73 **Expect:** 5e-006

**Matches (Bold Red):** 20/429 fragment ions using 18 most intense peaks

| **#** | **Immon.** | **a** | **a*** | **a^0^** | **b** | **b*** | **b^0^** | **Seq.** | **v** | **w** | **w'** | **y** | **y*** | **y^0^** | **#** |
| --- | --- | --- | --- | --- | --- | --- | --- | --- | --- | --- | --- | --- | --- | --- | --- |
| **1** | 30.03 | 174.14 |  |  | 202.13 |  |  | **G** |  |  |  |  |  |  | **22** |
| **2** | 30.03 | 231.16 |  |  | 259.15 |  |  | **G** |  |  |  | 1872.87 | 1855.84 | 1854.86 | **21** |
| **3** | 88.04 | 346.18 |  | 328.17 | **374.18** |  | 356.17 | **D** | 1755.83 | 1754.83 |  | 1815.85 | 1798.82 | 1797.84 | **20** |
| **4** | 60.04 | 433.22 |  | 415.21 | **461.21** |  | 443.20 | **S** | 1668.79 | 1667.80 |  | **1700.82** | 1683.79 | 1682.81 | **19** |
| **5** | 74.06 | 534.26 |  | 516.25 | 562.26 |  | 544.25 | **T** | 1567.75 | 1580.77 | 1582.75 | 1613.79 | 1596.76 | 1595.78 | **18** |
| **6** | 30.03 | 591.29 |  | 573.28 | 619.28 |  | 601.27 | **G** |  |  |  | **1512.74** | 1495.71 | 1494.73 | **17** |
| **7** | 30.03 | 648.31 |  | 630.30 | 676.30 |  | 658.29 | **G** |  |  |  | 1455.72 | 1438.69 | 1437.71 | **16** |
| **8** | 102.05 | 777.35 |  | 759.34 | 805.34 |  | 787.33 | **E** | 1324.66 | 1323.67 |  | 1398.70 | 1381.67 | 1380.69 | **15** |
| **9** | 44.05 | 848.39 |  | 830.38 | 876.38 |  | 858.37 | **A** | 1253.62 |  |  | **1269.65** | 1252.63 | 1251.64 | **14** |
| **10** | 30.03 | 905.41 |  | 887.40 | 933.40 |  | 915.39 | **G** |  |  |  | 1198.62 | 1181.59 | 1180.61 | **13** |
| **11** | 30.03 | 962.43 |  | 944.42 | 990.42 |  | 972.41 | **G** |  |  |  | **1141.60** | 1124.57 | 1123.59 | **12** |
| **12** | 74.06 | 1063.48 |  | 1045.47 | 1091.47 |  | 1073.46 | **T** | 1038.53 | 1051.55 | 1053.53 | 1084.57 | 1067.55 | 1066.56 | **11** |
| **13** | 44.05 | 1134.51 |  | 1116.50 | 1162.51 |  | 1144.50 | **A** | 967.50 |  |  | **983.53** | 966.50 | 965.52 | **10** |
| **14** | 44.05 | 1205.55 |  | 1187.54 | 1233.55 |  | 1215.54 | **A** | 896.46 |  |  | **912.49** | 895.46 | 894.48 | **9** |
| **15** | 30.03 | 1262.57 |  | 1244.56 | 1290.57 |  | 1272.56 | **G** |  |  |  | **841.45** | 824.43 | 823.44 | **8** |
| **16** | **70.07** | 1359.63 |  | 1341.62 | 1387.62 |  | 1369.61 | **P** | 742.38 | 741.39 |  | **784.43** | 767.40 | 766.42 | **7** |
| **17** | 44.05 | 1430.66 |  | 1412.65 | 1458.66 |  | 1440.65 | **A** | 671.35 |  |  | 687.38 | 670.35 | 669.37 | **6** |
| **18** | 101.07 | 1558.72 | 1541.70 | 1540.71 | 1586.72 | 1569.69 | 1568.71 | **Q** | 543.29 | 542.29 |  | 616.34 | 599.31 | 598.33 | **5** |
| **19** | 102.05 | 1687.76 | 1670.74 | 1669.75 | 1715.76 | 1698.73 | 1697.75 | **E** | 414.25 | 413.25 |  | 488.28 | 471.26 | 470.27 | **4** |
| **20** | 44.05 | 1758.80 | 1741.77 | 1740.79 | 1786.80 | 1769.77 | 1768.79 | **A** | 343.21 |  |  | **359.24** | 342.21 |  | **3** |
| **21** | **86.10** | 1871.89 | 1854.86 | 1853.87 | 1899.88 | 1882.85 | 1881.87 | **I** | 230.12 | 243.15 | 257.16 | 288.20 | 271.18 |  | **2** |
| **22** | 129.11 |  |  |  |  |  |  | **R** | 74.02 | 73.03 |  | **175.12** | 158.09 |  | **1** |

| **Seq** | **ya** | **yb** | **Seq** | **ya** | **yb** | **Seq** | **ya** | **yb** |
| --- | --- | --- | --- | --- | --- | --- | --- | --- |
| **GD** | 145.06 | 173.06 | **GDS** | 232.09 | 260.09 | **GDST** | 333.14 | 361.14 |
| **GDSTG** | 390.16 | 418.16 | **GDSTGG** | 447.18 | 475.18 | **GDSTGGE** | 576.23 | 604.22 |
| **GDSTGGEA** | 647.26 | 675.26 | **DS** | **175.07** | 203.07 | **DST** | 276.12 | 304.11 |
| **DSTG** | 333.14 | 361.14 | **DSTGG** | 390.16 | 418.16 | **DSTGGE** | 519.20 | 547.20 |
| **DSTGGEA** | 590.24 | 618.24 | **DSTGGEAG** | 647.26 | 675.26 | **ST** | 161.09 | 189.09 |
| **STG** | 218.11 | 246.11 | **STGG** | 275.13 | 303.13 | **STGGE** | 404.18 | 432.17 |
| **STGGEA** | 475.21 | 503.21 | **STGGEAG** | 532.24 | 560.23 | **STGGEAGG** | 589.26 | 617.25 |
| **STGGEAGGT** | 690.31 | 718.30 | **TG** | 131.08 | 159.08 | **TGG** | 188.10 | 216.10 |
| **TGGE** | 317.15 | 345.14 | **TGGEA** | 388.18 | 416.18 | **TGGEAG** | 445.20 | 473.20 |
| **TGGEAGG** | 502.23 | 530.22 | **TGGEAGGT** | 603.27 | 631.27 | **TGGEAGGTA** | 674.31 | 702.31 |
| **GG** | 87.06 | 115.05 | **GGE** | 216.10 | 244.09 | **GGEA** | 287.13 | 315.13 |
| **GGEAG** | 344.16 | 372.15 | **GGEAGG** | 401.18 | 429.17 | **GGEAGGT** | 502.23 | 530.22 |
| **GGEAGGTA** | 573.26 | 601.26 | **GGEAGGTAA** | 644.30 | 672.29 | **GE** | 159.08 | 187.07 |
| **GEA** | 230.11 | 258.11 | **GEAG** | 287.13 | 315.13 | **GEAGG** | 344.16 | 372.15 |
| **GEAGGT** | 445.20 | 473.20 | **GEAGGTA** | 516.24 | 544.24 | **GEAGGTAA** | 587.28 | 615.27 |
| **GEAGGTAAG** | 644.30 | 672.29 | **EA** | 173.09 | 201.09 | **EAG** | 230.11 | 258.11 |
| **EAGG** | 287.13 | 315.13 | **EAGGT** | 388.18 | 416.18 | **EAGGTA** | 459.22 | 487.21 |
| **EAGGTAA** | 530.26 | 558.25 | **EAGGTAAG** | 587.28 | 615.27 | **EAGGTAAGP** | 684.33 | 712.33 |
| **AG** | 101.07 | 129.07 | **AGG** | 158.09 | 186.09 | **AGGT** | 259.14 | 287.13 |
| **AGGTA** | 330.18 | 358.17 | **AGGTAA** | 401.21 | 429.21 | **AGGTAAG** | 458.24 | 486.23 |
| **AGGTAAGP** | 555.29 | 583.28 | **AGGTAAGPA** | 626.33 | 654.32 | **GG** | 87.06 | 115.05 |
| **GGT** | 188.10 | 216.10 | **GGTA** | 259.14 | 287.13 | **GGTAA** | 330.18 | 358.17 |
| **GGTAAG** | 387.20 | 415.19 | **GGTAAGP** | 484.25 | 512.25 | **GGTAAGPA** | 555.29 | 583.28 |
| **GGTAAGPAQ** | 683.35 | 711.34 | **GT** | 131.08 | 159.08 | **GTA** | 202.12 | 230.11 |
| **GTAA** | 273.16 | 301.15 | **GTAAG** | 330.18 | 358.17 | **GTAAGP** | 427.23 | 455.22 |
| **GTAAGPA** | 498.27 | 526.26 | **GTAAGPAQ** | 626.33 | 654.32 | **TA** | 145.10 | 173.09 |
| **TAA** | 216.13 | 244.13 | **TAAG** | 273.16 | 301.15 | **TAAGP** | 370.21 | 398.20 |
| **TAAGPA** | 441.25 | 469.24 | **TAAGPAQ** | 569.30 | 597.30 | **TAAGPAQE** | 698.35 | 726.34 |
| **AA** | 115.09 | 143.08 | **AAG** | 172.11 | 200.10 | **AAGP** | 269.16 | **297.16** |
| **AAGPA** | 340.20 | 368.19 | **AAGPAQ** | 468.26 | 496.25 | **AAGPAQE** | 597.30 | 625.29 |
| **AAGPAQEA** | 668.34 | 696.33 | **AG** | 101.07 | 129.07 | **AGP** | 198.12 | **226.12** |
| **AGPA** | 269.16 | **297.16** | **AGPAQ** | 397.22 | 425.21 | **AGPAQE** | 526.26 | 554.26 |
| **AGPAQEA** | 597.30 | 625.29 | **GP** | 127.09 | 155.08 | **GPA** | 198.12 | **226.12** |
| **GPAQ** | 326.18 | 354.18 | **GPAQE** | 455.22 | 483.22 | **GPAQEA** | 526.26 | 554.26 |
| **GPAQEAI** | 639.35 | 667.34 | **PA** | 141.10 | 169.10 | **PAQ** | 269.16 | **297.16** |
| **PAQE** | 398.20 | 426.20 | **PAQEA** | 469.24 | 497.24 | **PAQEAI** | 582.32 | 610.32 |
| **AQ** | 172.11 | 200.10 | **AQE** | 301.15 | 329.15 | **AQEA** | 372.19 | 400.18 |
| **AQEAI** | 485.27 | 513.27 | **QE** | 230.11 | 258.11 | **QEA** | 301.15 | 329.15 |
| **QEAI** | 414.23 | 442.23 | **EA** | 173.09 | 201.09 | **EAI** | 286.18 | 314.17 |
| **AI** | 157.13 | 185.13 |  |  |  |  |  |  |

22. Tb927.8.5830

Match to: **Tb927.8.5830** Score: **72**

**hypothetical protein, conserved; Trypanosoma bruceichr 8Manual**

Nominal mass (M_r_): **37967**; Calculated pI value: **4.36**

NCBI BLAST search of [Tb927.8.5830](http://www.ncbi.nlm.nih.gov/blast/Blast.cgi?ALIGNMENTS=50&ALIGNMENT_VIEW=Pairwise&AUTO_FORMAT=Semiauto&CDD_SEARCH=on&CLIENT=web&COMPOSITION_BASED_STATISTICS=on&DATABASE=nr&DESCRIPTIONS=100&ENTREZ_QUERY=(none)&EXPECT=10&FILTER=L&FORMAT_BLOCK_ON_RESPAGE=None&FORMAT_OBJECT=Alignment&FORMAT_TYPE=HTML&GAPCOSTS=11+1&I_THRESH=0.001&LAYOUT=TwoWindows&MATRIX_NAME=BLOSUM62&NCBI_GI=on&PAGE=Proteins&PROGRAM=blastp&QUERY=MEFVAEAHGYLITGLALDSQGDLFAICSCSGELLRLNKEENTLVSIMATEASPYNIAIEPNSGSVFITDRSENAILKLEDAATVRAEQELREGKDQESQAAYTTVQYLNAFEGRSFLGPTAIAFSPSGELFFTDAGAEGDSSFSDPVGAVYRTTMNHEHLVPICTRGLIRPSAIAVAPDNSVYVCEQGTNRVLRFVQRSTFYVGNVFAQLQGGMGPRAIAVSPRDGSVFVAQYDISAVEAPTEEGESEKGNEDAGEGEGGDDGKVQNQSALESAGEGGIITVLGRDGDVRGIVRTMRPCITAIALDASGETLYVMEGDEANGSSKLYKLQVLPLDDQQCGGQAE&SERVICE=plain&SET_DEFAULTS.x=9&SET_DEFAULTS.y=5&SHOW_OVERVIEW=on&WORD_SIZE=3&END_OF_HTTPGET=Yes) against nr

Unformatted [sequence string](../../../../D:%5CProteomic%20data%5C2010-1-8%5Ccgi%5Cgetseq.pl%3FTBA927_IPI+Tb927%2E8%2E5830+seq) for pasting into other applications

Fixed modifications: MMTS (C),(N-TERM)_iTRAQ,Lysine(K)_iTRAQ

Variable modifications: Oxidation (M)

Cleavage by Trypsin: cuts C-term side of KR unless next residue is P

Sequence Coverage: **5%**

Matched peptides shown in **Bold Red**

**1** MEFVAEAHGY LITGLALDSQ GDLFAICSCS GELLRLNKEE NTLVSIMATE

**51** ASPYNIAIEP NSGSVFITDR SENAILKLED AATVRAEQEL REGKDQESQA

**101** AYTTVQYLNA FEGRSFLGPT AIAFSPSGEL FFTDAGAEGD SSFSDPVGAV

**151** YRTTMNHEHL VPICTRGLIR PSAIAVAPDN SVYVCEQGTN RVLRFVQR**ST**

**201 FYVGNVFAQL QGGMGPR**AIA VSPRDGSVFV AQYDISAVEA PTEEGESEKG

**251** NEDAGEGEGG DDGKVQNQSA LESAGEGGII TVLGRDGDVR GIVRTMRPCI

**301** TAIALDASGE TLYVMEGDEA NGSSKLYKLQ VLPLDDQQCG GQAE

MS/MS Fragmentation of **STFYVGNVFAQLQGGMGPR**
Found in **Tb927.8.5830**, hypothetical protein, conserved; Trypanosoma bruceichr 8Manual


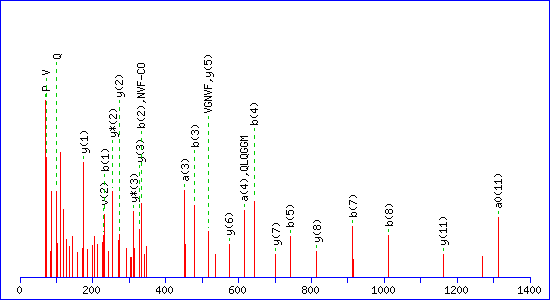


**MONOISOTOPIC mass of neutral peptide Mr(calc):** 2172.09

**Fixed modifications:** MMTS (C),(N-TERM)_iTRAQ,Lysine(K)_iTRAQ

**Ions Score:** 72 **Expect:** 5.8e-006

**Matches (Bold Red):** 29/325 fragment ions using 30 most intense peaks

| **#** | **Immon.** | **a** | **a*** | **a^0^** | **b** | **b*** | **b^0^** | **Seq.** | **v** | **w** | **w'** | **y** | **y*** | **y^0^** | **#** |
| --- | --- | --- | --- | --- | --- | --- | --- | --- | --- | --- | --- | --- | --- | --- | --- |
| **1** | 60.04 | 204.15 |  | 186.14 | **232.14** |  | 214.13 | **S** |  |  |  |  |  |  | **19** |
| **2** | 74.06 | 305.19 |  | 287.18 | **333.19** |  | 315.18 | **T** | 1895.92 | 1908.94 | 1910.92 | 1941.96 | 1924.94 | 1923.95 | **18** |
| **3** | 120.08 | **452.26** |  | 434.25 | **480.26** |  | 462.25 | **F** | 1748.85 |  |  | 1840.92 | 1823.89 |  | **17** |
| **4** | 136.08 | **615.33** |  | 597.32 | **643.32** |  | 625.31 | **Y** | 1585.79 |  |  | 1693.85 | 1676.82 |  | **16** |
| **5** | **72.08** | 714.39 |  | 696.38 | **742.39** |  | 724.38 | **V** | 1486.72 | 1499.74 |  | 1530.78 | 1513.76 |  | **15** |
| **6** | 30.03 | 771.42 |  | 753.41 | 799.41 |  | 781.40 | **G** |  |  |  | 1431.72 | 1414.69 |  | **14** |
| **7** | 87.06 | 885.46 | 868.43 | 867.45 | **913.45** | 896.43 | 895.44 | **N** | 1315.66 | 1314.66 |  | 1374.69 | 1357.67 |  | **13** |
| **8** | **72.08** | 984.53 | 967.50 | 966.52 | **1012.52** | 995.50 | 994.51 | **V** | 1216.59 | 1229.61 |  | 1260.65 | 1243.63 |  | **12** |
| **9** | 120.08 | 1131.60 | 1114.57 | 1113.59 | 1159.59 | 1142.56 | 1141.58 | **F** | 1069.52 |  |  | **1161.58** | 1144.56 |  | **11** |
| **10** | 44.05 | 1202.63 | 1185.61 | 1184.62 | 1230.63 | 1213.60 | 1212.62 | **A** | 998.48 |  |  | 1014.51 | 997.49 |  | **10** |
| **11** | **101.07** | 1330.69 | 1313.67 | **1312.68** | 1358.69 | 1341.66 | 1340.68 | **Q** | 870.43 | 869.43 |  | 943.48 | 926.45 |  | **9** |
| **12** | 86.10 | 1443.78 | 1426.75 | 1425.77 | 1471.77 | 1454.74 | 1453.76 | **L** | 757.34 | 756.35 |  | **815.42** | 798.39 |  | **8** |
| **13** | **101.07** | 1571.83 | 1554.81 | 1553.82 | 1599.83 | 1582.80 | 1581.82 | **Q** | 629.28 | 628.29 |  | **702.34** | 685.31 |  | **7** |
| **14** | 30.03 | 1628.86 | 1611.83 | 1610.85 | 1656.85 | 1639.82 | 1638.84 | **G** |  |  |  | **574.28** | 557.25 |  | **6** |
| **15** | 30.03 | 1685.88 | 1668.85 | 1667.87 | 1713.87 | 1696.85 | 1695.86 | **G** |  |  |  | **517.26** | 500.23 |  | **5** |
| **16** | 104.05 | 1816.92 | 1799.89 | 1798.91 | 1844.91 | 1827.89 | 1826.90 | **M** | 384.20 | 383.20 |  | 460.23 | 443.21 |  | **4** |
| **17** | 30.03 | 1873.94 | 1856.91 | 1855.93 | 1901.93 | 1884.91 | 1883.92 | **G** |  |  |  | **329.19** | **312.17** |  | **3** |
| **18** | **70.07** | 1970.99 | 1953.97 | 1952.98 | 1998.99 | 1981.96 | 1980.98 | **P** | **230.12** | 229.13 |  | **272.17** | **255.15** |  | **2** |
| **19** | 129.11 |  |  |  |  |  |  | **R** | 74.02 | 73.03 |  | **175.12** | 158.09 |  | **1** |

| **Seq** | **ya** | **yb** | **Seq** | **ya** | **yb** | **Seq** | **ya** | **yb** |
| --- | --- | --- | --- | --- | --- | --- | --- | --- |
| **TF** | 221.13 | 249.12 | **TFY** | 384.19 | 412.19 | **TFYV** | 483.26 | 511.26 |
| **TFYVG** | 540.28 | 568.28 | **TFYVGN** | 654.32 | 682.32 | **FY** | 283.14 | 311.14 |
| **FYV** | 382.21 | 410.21 | **FYVG** | 439.23 | 467.23 | **FYVGN** | 553.28 | 581.27 |
| **FYVGNV** | 652.35 | 680.34 | **YV** | 235.14 | 263.14 | **YVG** | 292.17 | 320.16 |
| **YVGN** | 406.21 | 434.20 | **YVGNV** | 505.28 | 533.27 | **YVGNVF** | 652.35 | 680.34 |
| **VG** | 129.10 | 157.10 | **VGN** | 243.15 | 271.14 | **VGNV** | 342.21 | 370.21 |
| **VGNVF** | 489.28 | **517.28** | **VGNVFA** | 560.32 | 588.31 | **VGNVFAQ** | 688.38 | 716.37 |
| **GN** | 144.08 | 172.07 | **GNV** | 243.15 | 271.14 | **GNVF** | 390.21 | 418.21 |
| **GNVFA** | 461.25 | 489.25 | **GNVFAQ** | 589.31 | 617.30 | **NV** | 186.12 | 214.12 |
| **NVF** | **333.19** | 361.19 | **NVFA** | 404.23 | 432.22 | **NVFAQ** | 532.29 | 560.28 |
| **NVFAQL** | 645.37 | 673.37 | **VF** | 219.15 | 247.14 | **VFA** | 290.19 | 318.18 |
| **VFAQ** | 418.24 | 446.24 | **VFAQL** | 531.33 | 559.32 | **VFAQLQ** | 659.39 | 687.38 |
| **FA** | 191.12 | 219.11 | **FAQ** | 319.18 | 347.17 | **FAQL** | 432.26 | 460.26 |
| **FAQLQ** | 560.32 | 588.31 | **FAQLQG** | 617.34 | 645.34 | **FAQLQGG** | 674.36 | 702.36 |
| **AQ** | 172.11 | 200.10 | **AQL** | 285.19 | 313.19 | **AQLQ** | 413.25 | 441.25 |
| **AQLQG** | 470.27 | 498.27 | **AQLQGG** | 527.29 | 555.29 | **AQLQGGM** | 658.33 | 686.33 |
| **QL** | 214.16 | 242.15 | **QLQ** | 342.21 | 370.21 | **QLQG** | 399.24 | 427.23 |
| **QLQGG** | 456.26 | 484.25 | **QLQGGM** | 587.30 | **615.29** | **QLQGGMG** | 644.32 | 672.31 |
| **LQ** | 214.16 | 242.15 | **LQG** | 271.18 | 299.17 | **LQGG** | 328.20 | 356.19 |
| **LQGGM** | 459.24 | 487.23 | **LQGGMG** | 516.26 | 544.25 | **LQGGMGP** | 613.31 | 641.31 |
| **QG** | 158.09 | 186.09 | **QGG** | 215.11 | 243.11 | **QGGM** | 346.15 | 374.15 |
| **QGGMG** | 403.18 | 431.17 | **QGGMGP** | 500.23 | 528.22 | **GG** | 87.06 | 115.05 |
| **GGM** | 218.10 | 246.09 | **GGMG** | 275.12 | 303.11 | **GGMGP** | 372.17 | 400.16 |
| **GM** | 161.07 | 189.07 | **GMG** | 218.10 | 246.09 | **GMGP** | 315.15 | 343.14 |
| **MG** | 161.07 | 189.07 | **MGP** | 258.13 | 286.12 | **GP** | 127.09 | 155.08 |

23. Tb09.211.0890

Match to: **Tb09.211.0890** Score: **69**

**hypothetical protein, conserved; Trypanosoma bruceichr 9Manual**

Nominal mass (M_r_): **22253**; Calculated pI value: **7.62**

NCBI BLAST search of [Tb09.211.0890](http://www.ncbi.nlm.nih.gov/blast/Blast.cgi?ALIGNMENTS=50&ALIGNMENT_VIEW=Pairwise&AUTO_FORMAT=Semiauto&CDD_SEARCH=on&CLIENT=web&COMPOSITION_BASED_STATISTICS=on&DATABASE=nr&DESCRIPTIONS=100&ENTREZ_QUERY=(none)&EXPECT=10&FILTER=L&FORMAT_BLOCK_ON_RESPAGE=None&FORMAT_OBJECT=Alignment&FORMAT_TYPE=HTML&GAPCOSTS=11+1&I_THRESH=0.001&LAYOUT=TwoWindows&MATRIX_NAME=BLOSUM62&NCBI_GI=on&PAGE=Proteins&PROGRAM=blastp&QUERY=MQELAVFKRPHLHACSEYVDVAVELAPLRRCESFTDFLQLLQGELEFIYGSAPKSFNNAILYSTHEAPCSFSCYFSEKQLEMLRNFDEACEKESQMRVSYENVVAEYDAKVEENKDRKMNRRRRMEMEKARKRVKVMDRDVKQAEYEVKKSAQKLANIFQIAALRVLLN&SERVICE=plain&SET_DEFAULTS.x=9&SET_DEFAULTS.y=5&SHOW_OVERVIEW=on&WORD_SIZE=3&END_OF_HTTPGET=Yes) against nr

Unformatted [sequence string](../../../../D:%5CProteomic%20data%5C2010-1-8%5Ccgi%5Cgetseq.pl%3FTBA927_IPI+Tb09%2E211%2E0890+seq) for pasting into other applications

Fixed modifications: MMTS (C),(N-TERM)_iTRAQ,Lysine(K)_iTRAQ

Variable modifications: Oxidation (M)

Cleavage by Trypsin: cuts C-term side of KR unless next residue is P

Sequence Coverage: **12%**

Matched peptides shown in **Bold Red**

**1** MQELAVFKRP HLHACSEYVD VAVELAPLRR CESFTDFLQL LQGELEFIYG

**51** SAPKSFNNAI LYSTHEAPCS FSCYFSEKQL EMLRNFDEAC EKESQMRVSY

**101** ENVVAEYDAK VEENKDRKMN RRRRMEMEKA RKRVKVMDR**D VKQAEYEVK**K

**151** SAQK**LANIFQ IAALR**VLLN

**Start - End Observed Mr(expt) Mr(calc) Delta Miss Sequence**

**140 - 149 1640.85 1639.85 1639.74 0.11 1 R.DVKQAEYEVK.K**  ([Ions score 5](../../../../D:%5CProteomic%20data%5C2010-1-8%5CZQ%5C1112.htm))

**143 - 149 1154.60 1153.59 1153.53 0.06 0 K.QAEYEVK.K**  ([Ions score 9](../../../../D:%5CProteomic%20data%5C2010-1-8%5CZQ%5C1110.htm))

**155 - 165 1373.84 1372.83 1372.83 -0.00 0 K.LANIFQIAALR.V**  ([Ions score 69](../../../../D:%5CProteomic%20data%5C2010-1-8%5CZQ%5C1111.htm))

MS/MS Fragmentation of **LANIFQIAALR**
Found in **Tb09.211.0890**, hypothetical protein, conserved; Trypanosoma bruceichr 9Manual


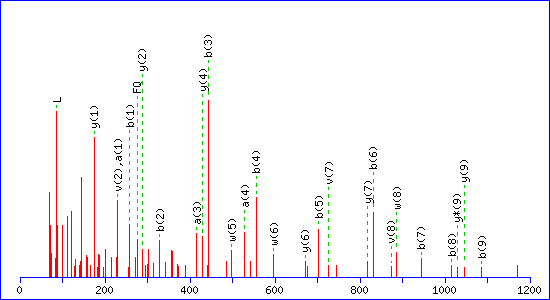


**MONOISOTOPIC mass of neutral peptide Mr(calc):** 1372.83

**Fixed modifications:** MMTS (C),(N-TERM)_iTRAQ,Lysine(K)_iTRAQ

**Ions Score:** 69 **Expect:** 1.2e-005

**Matches (Bold Red):** 30/145 fragment ions using 29 most intense peaks

| **#** | **Immon.** | **a** | **a*** | **b** | **b*** | **Seq.** | **v** | **w** | **w'** | **y** | **y*** | **#** |
| --- | --- | --- | --- | --- | --- | --- | --- | --- | --- | --- | --- | --- |
| **1** | **86.10** | **230.20** |  | **258.19** |  | **L** |  |  |  |  |  | **11** |
| **2** | 44.05 | 301.24 |  | **329.23** |  | **A** | 1100.62 |  |  | 1116.65 | 1099.63 | **10** |
| **3** | 87.06 | **415.28** | 398.25 | **443.27** | 426.25 | **N** | 986.58 | 985.58 |  | **1045.62** | **1028.59** | **9** |
| **4** | **86.10** | **528.36** | 511.34 | **556.36** | 539.33 | **I** | **873.49** | **886.51** | 900.53 | 931.57 | 914.55 | **8** |
| **5** | 120.08 | 675.43 | 658.40 | **703.43** | 686.40 | **F** | **726.43** |  |  | **818.49** | 801.46 | **7** |
| **6** | 101.07 | 803.49 | 786.46 | **831.48** | 814.46 | **Q** | 598.37 | **597.37** |  | **671.42** | 654.39 | **6** |
| **7** | **86.10** | 916.57 | 899.55 | **944.57** | 927.54 | **I** | 485.28 | **498.30** | 512.32 | 543.36 | 526.33 | **5** |
| **8** | 44.05 | 987.61 | 970.58 | **1015.61** | 998.58 | **A** | 414.25 |  |  | **430.28** | 413.25 | **4** |
| **9** | 44.05 | 1058.65 | 1041.62 | **1086.64** | 1069.62 | **A** | 343.21 |  |  | 359.24 | 342.21 | **3** |
| **10** | **86.10** | 1171.73 | 1154.71 | 1199.73 | 1182.70 | **L** | **230.12** | 229.13 |  | **288.20** | 271.18 | **2** |
| **11** | 129.11 |  |  |  |  | **R** | 74.02 | 73.03 |  | **175.12** | 158.09 | **1** |

| **Seq** | **ya** | **yb** | **Seq** | **ya** | **yb** | **Seq** | **ya** | **yb** |
| --- | --- | --- | --- | --- | --- | --- | --- | --- |
| **AN** | 158.09 | 186.09 | **ANI** | 271.18 | 299.17 | **ANIF** | 418.24 | 446.24 |
| **ANIFQ** | 546.30 | 574.30 | **ANIFQI** | 659.39 | 687.38 | **NI** | 200.14 | 228.13 |
| **NIF** | 347.21 | 375.20 | **NIFQ** | 475.27 | 503.26 | **NIFQI** | 588.35 | 616.35 |
| **NIFQIA** | 659.39 | 687.38 | **IF** | 233.16 | 261.16 | **IFQ** | 361.22 | 389.22 |
| **IFQI** | 474.31 | 502.30 | **IFQIA** | 545.34 | 573.34 | **IFQIAA** | 616.38 | 644.38 |
| **FQ** | 248.14 | **276.13** | **FQI** | 361.22 | 389.22 | **FQIA** | 432.26 | 460.26 |
| **FQIAA** | 503.30 | 531.29 | **FQIAAL** | 616.38 | 644.38 | **QI** | 214.16 | 242.15 |
| **QIA** | 285.19 | 313.19 | **QIAA** | 356.23 | 384.22 | **QIAAL** | 469.31 | 497.31 |
| **IA** | 157.13 | 185.13 | **IAA** | 228.17 | 256.17 | **IAAL** | 341.25 | 369.25 |
| **AA** | 115.09 | 143.08 | **AAL** | 228.17 | 256.17 | **AL** | 157.13 | 185.13 |

24. Tb11.01.2670

Match to: **Tb11.01.2670** Score: **68**

**MENG; Trypanosoma bruceichr 11Manual**

Nominal mass (M_r_): **112864**; Calculated pI value: **6.44**

NCBI BLAST search of [Tb11.01.2670](http://www.ncbi.nlm.nih.gov/blast/Blast.cgi?ALIGNMENTS=50&ALIGNMENT_VIEW=Pairwise&AUTO_FORMAT=Semiauto&CDD_SEARCH=on&CLIENT=web&COMPOSITION_BASED_STATISTICS=on&DATABASE=nr&DESCRIPTIONS=100&ENTREZ_QUERY=(none)&EXPECT=10&FILTER=L&FORMAT_BLOCK_ON_RESPAGE=None&FORMAT_OBJECT=Alignment&FORMAT_TYPE=HTML&GAPCOSTS=11+1&I_THRESH=0.001&LAYOUT=TwoWindows&MATRIX_NAME=BLOSUM62&NCBI_GI=on&PAGE=Proteins&PROGRAM=blastp&QUERY=MENGKNDEFTVSDEAVENLQKDFEEAMAALAEHESFDRFRMEYDVLYRALRKSHDSEKRLVKRCQQLTQELMSNAAKVQAALKLSQSDHTTIDALKKEIEKAWRMVDSANEKDAHAKETMKNLKEEVASLQEIMANGAELTSSQSATLEGLKLEKKRMEMEYGELVKQMDNLTKEIKELNTKSKELEVEIMNNQEEFKRVTDRETLIQQEYDKEIKARERADFQVKEQLHLAQQRAKELKTHEQLRINLTETVTKLRAQVQEDNEKRQLLEQKIETAEKQLYHTQQSYDDAVDTTEALNERHRAVCKEIAEAEKMAHDLLSEEERTRAVCDGDYKKLRRLIQQNDDVRQEYENLTRQQSNIQKRINTVKKERHAMNNAYEVLQKEQDTLKKYGEHERKKLQTIEGIIANEVESQKDVEAAIEREREISVRLSKTIAKLESEREKYTAEVLQAVEQHALVKEDLKVATITCNETQKAIEESEQRLKKQQGLYEQARAERNLYTKKLIESQDEVMELKQGFRMMDHQIRQLKEELAMKEKKFQDETSAQKIAKEKLAKVRRVVNERTIALDDTIRNCENVAQNIKQLVKVVNECDKQLSEQRQMFLSVSNERDMLGTQLIRRNDELALLYEKIRMQQEVLSRGYAACRARQEDMRLLRLKTEDLKRQAKIADRRAQDTKQLQEDIKQLVYDLTVQRAKVQALTEEAENPKSSLRWEKVDGRNPTAEELNRKIFRLQRRLITKSEECVEKDMELQEKQRLLTELTNILARQPGPEVVQRLNMCQKELHRTCSVMKQKASELNMTGTHFAELKYEAERLRREVNDTRRKYYEMRMSNDELTKAMEASRSIKS&SERVICE=plain&SET_DEFAULTS.x=9&SET_DEFAULTS.y=5&SHOW_OVERVIEW=on&WORD_SIZE=3&END_OF_HTTPGET=Yes) against nr

Unformatted [sequence string](../../../../D:%5CProteomic%20data%5C2010-1-8%5Ccgi%5Cgetseq.pl%3FTBA927_IPI+Tb11%2E01%2E2670+seq) for pasting into other applications

Fixed modifications: MMTS (C),(N-TERM)_iTRAQ,Lysine(K)_iTRAQ

Variable modifications: Oxidation (M)

Cleavage by Trypsin: cuts C-term side of KR unless next residue is P

Sequence Coverage: **1%**

Matched peptides shown in **Bold Red**

**1** MENGKNDEFT VSDEAVENLQ KDFEEAMAAL AEHESFDRFR MEYDVLYRAL

**51** RKSHDSEKRL VKRCQQLTQE LMSNAAKVQA ALKLSQSDHT TIDALKKEIE

**101** KAWRMVDSAN EKDAHAKETM KNLKEEVASL QEIMANGAEL TSSQSATLEG

**151** LKLEKKRMEM EYGELVKQMD NLTKEIKELN TKSKELEVEI MNNQEEFKRV

**201** TDRETLIQQE YDKEIKARER ADFQVKEQLH LAQQRAKELK THEQLRINLT

**251** ETVTKLRAQV QEDNEKRQLL EQKIETAEKQ LYHTQQSYDD AVDTTEALNE

**301** RHRAVCKEIA EAEKMAHDLL SEEERTRAVC DGDYKKLRRL IQQNDDVRQE

**351** YENLTRQQSN IQKRINTVKK ERHAMNNAYE VLQKEQDTLK KYGEHERKKL

**401** QTIEGIIANE VESQKDVEAA IEREREISVR LSKTIAKLES EREKYTAEVL

**451** QAVEQHALVK EDLKVATITC NETQKAIEES EQRLKKQQGL YEQARAERNL

**501** YTKKLIESQD EVMELKQGFR MMDHQIRQLK EELAMKEKKF QDETSAQKIA

**551** KEKLAKVRRV VNERTIALDD TIRNCENVAQ NIKQLVKVVN ECDKQLSEQR

**601** QMFLSVSNER DMLGTQLIRR NDELALLYEK IRMQQEVLSR GYAACRARQE

**651** DMRLLRLKTE DLKRQAKIAD RRAQDTKQLQ EDIKQLVYDL TVQRAKVQAL

**701** TEEAENPKSS LRWEKVDGRN PTAEELNRKI FRLQRRLITK SEECVEKDME

**751** LQEKQR**LLTE LTNILAR**QPG PEVVQRLNMC QKELHRTCSV MKQKASELNM

**801** TGTHFAELKY EAERLRREVN DTRRKYYEMR MSNDELTKAM EASRSIKS

MS/MS Fragmentation of **LLTELTNILAR**
Found in **Tb11.01.2670**, MENG; Trypanosoma bruceichr 11Manual


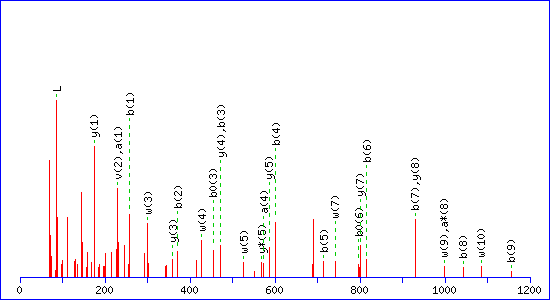


**MONOISOTOPIC mass of neutral peptide Mr(calc):** 1399.85

**Fixed modifications:** MMTS (C),(N-TERM)_iTRAQ,Lysine(K)_iTRAQ

**Ions Score:** 68 **Expect:** 9.6e-006

**Matches (Bold Red):** 33/162 fragment ions using 29 most intense peaks

| **#** | **Immon.** | **a** | **a*** | **a^0^** | **b** | **b*** | **b^0^** | **Seq.** | **v** | **w** | **w'** | **y** | **y*** | **y^0^** | **#** |
| --- | --- | --- | --- | --- | --- | --- | --- | --- | --- | --- | --- | --- | --- | --- | --- |
| **1** | **86.10** | **230.20** |  |  | **258.19** |  |  | **L** |  |  |  |  |  |  | **11** |
| **2** | **86.10** | 343.28 |  |  | **371.28** |  |  | **L** | 1085.59 | **1084.60** |  | 1143.67 | 1126.65 | 1125.66 | **10** |
| **3** | 74.06 | 444.33 |  | 426.32 | **472.33** |  | **454.31** | **T** | 984.55 | **997.57** | 999.55 | 1030.59 | 1013.56 | 1012.58 | **9** |
| **4** | 102.05 | **573.37** |  | 555.36 | **601.37** |  | 583.36 | **E** | 855.50 | 854.51 |  | **929.54** | 912.51 | 911.53 | **8** |
| **5** | **86.10** | 686.46 |  | 668.45 | **714.45** |  | 696.44 | **L** | 742.42 | **741.43** |  | **800.50** | 783.47 | 782.49 | **7** |
| **6** | 74.06 | 787.50 |  | 769.49 | **815.50** |  | **797.49** | **T** | 641.37 | 654.39 | 656.37 | 687.41 | 670.39 | 669.40 | **6** |
| **7** | 87.06 | 901.55 | 884.52 | 883.54 | **929.54** | 912.52 | 911.53 | **N** | 527.33 | **526.33** |  | **586.37** | **569.34** |  | **5** |
| **8** | **86.10** | 1014.63 | **997.61** | 996.62 | **1042.63** | 1025.60 | 1024.62 | **I** | 414.25 | **427.27** | 441.28 | **472.32** | 455.30 |  | **4** |
| **9** | **86.10** | 1127.72 | 1110.69 | 1109.71 | **1155.71** | 1138.68 | 1137.70 | **L** | 301.16 | **300.17** |  | **359.24** | 342.21 |  | **3** |
| **10** | 44.05 | 1198.75 | 1181.73 | 1180.74 | 1226.75 | 1209.72 | 1208.74 | **A** | **230.12** |  |  | 246.16 | 229.13 |  | **2** |
| **11** | 129.11 |  |  |  |  |  |  | **R** | 74.02 | 73.03 |  | **175.12** | 158.09 |  | **1** |

| **Seq** | **ya** | **yb** | **Seq** | **ya** | **yb** | **Seq** | **ya** | **yb** |
| --- | --- | --- | --- | --- | --- | --- | --- | --- |
| **LT** | 187.14 | 215.14 | **LTE** | 316.19 | 344.18 | **LTEL** | 429.27 | 457.27 |
| **LTELT** | 530.32 | 558.31 | **LTELTN** | 644.36 | 672.36 | **TE** | 203.10 | 231.10 |
| **TEL** | 316.19 | 344.18 | **TELT** | 417.23 | 445.23 | **TELTN** | 531.28 | 559.27 |
| **TELTNI** | 644.36 | 672.36 | **EL** | 215.14 | 243.13 | **ELT** | 316.19 | 344.18 |
| **ELTN** | 430.23 | 458.22 | **ELTNI** | 543.31 | 571.31 | **ELTNIL** | 656.40 | 684.39 |
| **LT** | 187.14 | 215.14 | **LTN** | 301.19 | 329.18 | **LTNI** | 414.27 | 442.27 |
| **LTNIL** | 527.36 | 555.35 | **LTNILA** | 598.39 | 626.39 | **TN** | 188.10 | 216.10 |
| **TNI** | 301.19 | 329.18 | **TNIL** | 414.27 | 442.27 | **TNILA** | 485.31 | 513.30 |
| **NI** | 200.14 | 228.13 | **NIL** | 313.22 | 341.22 | **NILA** | 384.26 | 412.26 |
| **IL** | 199.18 | 227.18 | **ILA** | 270.22 | 298.21 | **LA** | 157.13 | 185.13 |

25. Tb927.6.5040

Match to: **Tb927.6.5040** Score: **68**

**ribosomal protein L15, putative; Trypanosoma bruceichr 6Manual**

Nominal mass (M_r_): **27299**; Calculated pI value: **12.01**

NCBI BLAST search of [Tb927.6.5040](http://www.ncbi.nlm.nih.gov/blast/Blast.cgi?ALIGNMENTS=50&ALIGNMENT_VIEW=Pairwise&AUTO_FORMAT=Semiauto&CDD_SEARCH=on&CLIENT=web&COMPOSITION_BASED_STATISTICS=on&DATABASE=nr&DESCRIPTIONS=100&ENTREZ_QUERY=(none)&EXPECT=10&FILTER=L&FORMAT_BLOCK_ON_RESPAGE=None&FORMAT_OBJECT=Alignment&FORMAT_TYPE=HTML&GAPCOSTS=11+1&I_THRESH=0.001&LAYOUT=TwoWindows&MATRIX_NAME=BLOSUM62&NCBI_GI=on&PAGE=Proteins&PROGRAM=blastp&QUERY=MGAFMYMNELWKKKSSDVMRFIQRVRAWEFRHQHTIVRLRRPTRPEKARMVGYKTKQGYVVFRVRVRRGGRKRPVHKGITYGKPNTAGVLGRKLNKNNRVVAEQRLGKKYGNLRVLNSYWVNADSTFLWYEVVAVDPMHRTIRRDPRINWIVNAVHKHREQRGLTSAGRKHRGLRQKGHKASKLRPSRRAAWRRNNRMVFLRKR&SERVICE=plain&SET_DEFAULTS.x=9&SET_DEFAULTS.y=5&SHOW_OVERVIEW=on&WORD_SIZE=3&END_OF_HTTPGET=Yes) against nr

Unformatted [sequence string](../../../../D:%5CProteomic%20data%5C2010-1-8%5Ccgi%5Cgetseq.pl%3FTBA927_IPI+Tb927%2E6%2E5040+seq) for pasting into other applications

Fixed modifications: MMTS (C),(N-TERM)_iTRAQ,Lysine(K)_iTRAQ

Variable modifications: Oxidation (M)

Cleavage by Trypsin: cuts C-term side of KR unless next residue is P

Sequence Coverage: **7%**

Matched peptides shown in **Bold Red**

**1** MGAFMYMNEL WKKKSSDVMR FIQRVRAWEF RHQHTIVRLR RPTRPEKARM

**51** VGYKTKQGYV VFRVRVRRGG RKRPVHK**GIT YGKPNTAGVL GR**KLNKNNRV

**101** VAEQRLGKKY GNLRVLNSYW VNADSTFLWY EVVAVDPMHR TIRRDPRINW

**151** IVNAVHKHRE QRGLTSAGRK HRGLRQKGHK ASKLRPSRRA AWRRNNRMVF

**201** LRKR

MS/MS Fragmentation of **GITYGKPNTAGVLGR**
Found in **Tb927.6.5040**, ribosomal protein L15, putative; Trypanosoma bruceichr 6Manual


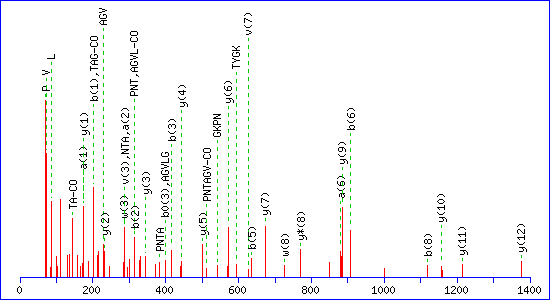


**MONOISOTOPIC mass of neutral peptide Mr(calc):** 1790.94

**Fixed modifications:** MMTS (C),(N-TERM)_iTRAQ,Lysine(K)_iTRAQ

**Ions Score:** 68 **Expect:** 1.3e-005

**Matches (Bold Red):** 41/239 fragment ions using 43 most intense peaks

| **#** | **Immon.** | **a** | **a*** | **a^0^** | **b** | **b*** | **b^0^** | **Seq.** | **v** | **w** | **w'** | **y** | **y*** | **y^0^** | **#** |
| --- | --- | --- | --- | --- | --- | --- | --- | --- | --- | --- | --- | --- | --- | --- | --- |
| **1** | 30.03 | **174.14** |  |  | **202.13** |  |  | **G** |  |  |  |  |  |  | **15** |
| **2** | **86.10** | **287.22** |  |  | **315.22** |  |  | **I** | 1532.74 | 1545.76 | 1559.78 | 1590.82 | 1573.79 | 1572.81 | **14** |
| **3** | 74.06 | 388.27 |  | 370.26 | **416.26** |  | **398.25** | **T** | 1431.69 | 1444.71 | 1446.69 | 1477.73 | 1460.71 | 1459.72 | **13** |
| **4** | 136.08 | 551.33 |  | 533.32 | 579.33 |  | 561.32 | **Y** | 1268.63 |  |  | **1376.69** | 1359.66 | 1358.68 | **12** |
| **5** | 30.03 | 608.35 |  | 590.34 | **636.35** |  | 618.34 | **G** |  |  |  | **1213.62** | 1196.60 | 1195.61 | **11** |
| **6** | 245.12 | **880.46** | 863.43 | 862.45 | **908.46** | 891.43 | 890.44 | **K** | 939.50 | 938.51 |  | **1156.60** | 1139.58 | 1138.59 | **10** |
| **7** | **70.07** | 977.51 | 960.49 | 959.50 | 1005.51 | 988.48 | 987.50 | **P** | 842.45 | 841.45 |  | **884.49** | 867.47 | 866.48 | **9** |
| **8** | 87.06 | 1091.56 | 1074.53 | 1073.55 | **1119.55** | 1102.52 | 1101.54 | **N** | 728.40 | **727.41** |  | 787.44 | **770.42** | 769.43 | **8** |
| **9** | 74.06 | 1192.60 | 1175.58 | 1174.59 | 1220.60 | 1203.57 | 1202.59 | **T** | **627.36** | 640.38 | 642.36 | **673.40** | 656.37 | 655.39 | **7** |
| **10** | 44.05 | 1263.64 | 1246.61 | 1245.63 | 1291.64 | 1274.61 | 1273.63 | **A** | 556.32 |  |  | **572.35** | 555.32 |  | **6** |
| **11** | 30.03 | 1320.66 | 1303.64 | 1302.65 | 1348.66 | 1331.63 | 1330.65 | **G** |  |  |  | **501.31** | 484.29 |  | **5** |
| **12** | **72.08** | 1419.73 | 1402.70 | 1401.72 | 1447.73 | 1430.70 | 1429.71 | **V** | 400.23 | 413.25 |  | **444.29** | 427.27 |  | **4** |
| **13** | **86.10** | 1532.81 | 1515.79 | 1514.80 | 1560.81 | 1543.78 | 1542.80 | **L** | **287.15** | **286.15** |  | **345.22** | 328.20 |  | **3** |
| **14** | 30.03 | 1589.84 | 1572.81 | 1571.83 | 1617.83 | 1600.80 | 1599.82 | **G** |  |  |  | **232.14** | 215.11 |  | **2** |
| **15** | 129.11 |  |  |  |  |  |  | **R** | 74.02 | 73.03 |  | **175.12** | 158.09 |  | **1** |

| **Seq** | **ya** | **yb** | **Seq** | **ya** | **yb** | **Seq** | **ya** | **yb** |
| --- | --- | --- | --- | --- | --- | --- | --- | --- |
| **IT** | 187.14 | 215.14 | **ITY** | 350.21 | 378.20 | **ITYG** | 407.23 | 435.22 |
| **ITYGK** | 679.34 | 707.33 | **TY** | 237.12 | 265.12 | **TYG** | 294.14 | 322.14 |
| **TYGK** | 566.25 | **594.25** | **TYGKP** | 663.30 | 691.30 | **YG** | 193.10 | 221.09 |
| **YGK** | 465.20 | 493.20 | **YGKP** | 562.26 | 590.25 | **YGKPN** | 676.30 | 704.30 |
| **GK** | 302.14 | 330.14 | **GKP** | 399.19 | 427.19 | **GKPN** | 513.24 | **541.23** |
| **GKPNT** | 614.28 | 642.28 | **GKPNTA** | 685.32 | 713.32 | **KP** | 342.17 | 370.17 |
| **KPN** | 456.22 | 484.21 | **KPNT** | 557.26 | 585.26 | **KPNTA** | 628.30 | 656.30 |
| **KPNTAG** | 685.32 | 713.32 | **PN** | 184.11 | 212.10 | **PNT** | 285.16 | **313.15** |
| **PNTA** | 356.19 | **384.19** | **PNTAG** | 413.21 | 441.21 | **PNTAGV** | **512.28** | 540.28 |
| **PNTAGVL** | 625.37 | 653.36 | **PNTAGVLG** | 682.39 | 710.38 | **NT** | 188.10 | 216.10 |
| **NTA** | 259.14 | **287.13** | **NTAG** | 316.16 | 344.16 | **NTAGV** | 415.23 | 443.22 |
| **NTAGVL** | 528.31 | 556.31 | **NTAGVLG** | 585.34 | 613.33 | **TA** | **145.10** | 173.09 |
| **TAG** | **202.12** | 230.11 | **TAGV** | 301.19 | 329.18 | **TAGVL** | 414.27 | 442.27 |
| **TAGVLG** | 471.29 | 499.29 | **AG** | 101.07 | 129.07 | **AGV** | 200.14 | **228.13** |
| **AGVL** | **313.22** | 341.22 | **AGVLG** | 370.24 | **398.24** | **GV** | 129.10 | 157.10 |
| **GVL** | 242.19 | 270.18 | **GVLG** | 299.21 | 327.20 | **VL** | 185.16 | 213.16 |
| **VLG** | 242.19 | 270.18 | **LG** | 143.12 | 171.11 |  |  |  |

26. Tb10.70.2650

Match to: **Tb10.70.2650** Score: **67**

**elongation factor 2; Trypanosoma bruceichr 10Manual**

Nominal mass (M_r_): **102734**; Calculated pI value: **5.83**

NCBI BLAST search of [Tb10.70.2650](http://www.ncbi.nlm.nih.gov/blast/Blast.cgi?ALIGNMENTS=50&ALIGNMENT_VIEW=Pairwise&AUTO_FORMAT=Semiauto&CDD_SEARCH=on&CLIENT=web&COMPOSITION_BASED_STATISTICS=on&DATABASE=nr&DESCRIPTIONS=100&ENTREZ_QUERY=(none)&EXPECT=10&FILTER=L&FORMAT_BLOCK_ON_RESPAGE=None&FORMAT_OBJECT=Alignment&FORMAT_TYPE=HTML&GAPCOSTS=11+1&I_THRESH=0.001&LAYOUT=TwoWindows&MATRIX_NAME=BLOSUM62&NCBI_GI=on&PAGE=Proteins&PROGRAM=blastp&QUERY=MVNFTVDEVRALMDYPEQIRNMSVIAHVDHGKSTLSDSLVGAAGIIKMEDAGDKRIMDTRADEIARGITIKSTAISMHYHVPPEIISDLPDDRRDFLINLIDSPGHVDFSSEVTAALRVTDGALVVVDCVEGVCVQTETVLRQALTERIRPVVFINKVDRAILELQLDPEEAYQGFVKTLQNVNVVIATYNDPVMGDVQVYPEKGTVAIGSGLQAWAFSVTRFAKMYASKFGVDESKMCERLWGDNFFDAKNKKWIKSETNAAGERVRRAFCQFCLDPIYQIFDAVMTEKAEKVEKMLKSLNINLTTEEREQVPKKLLKSIMMKFLPAAETLLQMIVAHLPSPKKAQSYRAEMLYSGESNPDEKYYMGIKNCDPNAPLMLYISKMVPTADRGRFFAFGRIFSGKVRCGQKVRIMGNNYIHGKKQDLYEDKPVQRTVLMMGRYQEAVEDMPCGNVVGLVGVDKYIVKSATITDDGESPHPLRDMKYSVSPVVRVAVEAKNPSDLPKLVEGLKRLAKSDPLVVCSIEESGEHIVAGAGELHLEICLKDLQEDFMNGAPLKISEPVVSFRETVTDVSSIQCLSKSANKHNRLFCRGAPLTEELCVEIEDGANAGSEADPKTRARFLADKFEWDVAEARKIWCYGPDNRGPNVVVDVTKGVQNMMEMKDSFVAAWQWATREGVLCDENMRGVRINVEDVTMHADAIHRGGGQIIPTARRVFYACCLTATPRLMEPMFQVDIQTVEHAMGGIYGVLTRRRGVIIGEENRPGTPIYNVRAYLPVAESFGFTADLRAGTGGQAFPQCVFDHWQQYPGDPLDPKSQANTLVLSIRQRKGLKPDIPGLDTFLDKL&SERVICE=plain&SET_DEFAULTS.x=9&SET_DEFAULTS.y=5&SHOW_OVERVIEW=on&WORD_SIZE=3&END_OF_HTTPGET=Yes) against nr

Unformatted [sequence string](../../../../D:%5CProteomic%20data%5C2010-1-8%5Ccgi%5Cgetseq.pl%3FTBA927_IPI+Tb10%2E70%2E2650+seq) for pasting into other applications

Fixed modifications: MMTS (C),(N-TERM)_iTRAQ,Lysine(K)_iTRAQ

Variable modifications: Oxidation (M)

Cleavage by Trypsin: cuts C-term side of KR unless next residue is P

Sequence Coverage: **8%**

Matched peptides shown in **Bold Red**

**1** MVNFTVDEVR ALMDYPEQIR NMSVIAHVDH GKSTLSDSLV GAAGIIKMED

**51** AGDKRIMDTR ADEIARGITI KSTAISMHYH VPPEIISDLP DDRRDFLINL

**101** IDSPGHVDFS SEVTAALRVT DGALVVVDCV EGVCVQTETV LRQALTERIR

**151** PVVFINKVDR AILELQLDPE EAYQGFVKTL QNVNVVIATY NDPVMGDVQV

**201** YPEKGTVAIG SGLQAWAFSV TRFAKMYASK FGVDESKMCE RLWGDNFFDA

**251** KNKKWIKSET NAAGERVRRA FCQFCLDPIY QIFDAVMTEK AEKVEKMLKS

**301** LNINLTTEER EQVPKKLLKS IMMKFLPAAE TLLQMIVAHL PSPKKAQSYR

**351** AEMLYSGESN PDEKYYMGIK NCDPNAPLML YISKMVPTAD RGRFFAFGRI

**401** FSGKVRCGQK VRIMGNNYIH GKKQDLYEDK PVQRTVLMMG RYQEAVEDMP

**451** CGNVVGLVGV DKYIVK**SATI TDDGESPHPL R**DMKYSVSPV VRVAVEAKNP

**501** SDLPKLVEGL KRLAKSDPLV VCSIEESGEH IVAGAGELHL EICLKDLQED

**551** FMNGAPLK**IS EPVVSFR**ETV TDVSSIQCLS KSANKHNRLF CRGAPLTEEL

**601** CVEIEDGANA GSEADPKTRA RFLADKFEWD VAEARKIWCY GPDNRGPNVV

**651** VDVTKGVQNM MEMKDSFVAA WQWATR**EGVL CDENMR**GVRI NVEDVTMHAD

**701** AIHRGGGQII PTARRVFYAC CLTATPRLME PMFQVDIQTV EHAMGGIYGV

**751** LTRRR**GVIIG EENRPGTPIY NVRAYLPVAE SFGFTADLR**A GTGGQAFPQC

**801** VFDHWQQYPG DPLDPKSQAN TLVLSIRQRK GLKPDIPGLD TFLDKL

**Start - End Observed Mr(expt) Mr(calc) Delta Miss Sequence**

**467 - 481 1739.86 1738.85 1738.86 -0.01 0 K.SATITDDGESPHPLR.D**  ([Ions score 2](../../../../D:%5CProteomic%20data%5C2010-1-8%5CZQ%5C1141.htm))

**559 - 567 1177.67 1176.66 1176.66 0.00 0 K.ISEPVVSFR.E**  ([Ions score 9](../../../../D:%5CProteomic%20data%5C2010-1-8%5CZQ%5C1139.htm))

**677 - 686 1371.72 1370.71 1370.58 0.13 0 R.EGVLCDENMR.G**  Oxidation (M) ([Ions score 2](../../../../D:%5CProteomic%20data%5C2010-1-8%5CZQ%5C1140.htm))

**756 - 773 2128.14 2127.14 2127.16 -0.02 0 R.GVIIGEENRPGTPIYNVR.A**  ([Ions score 17](../../../../D:%5CProteomic%20data%5C2010-1-8%5CZQ%5C1143.htm))

**774 - 789 1900.99 1899.98 1899.99 -0.00 0 R.AYLPVAESFGFTADLR.A**  ([Ions score 67](../../../../D:%5CProteomic%20data%5C2010-1-8%5CZQ%5C1142.htm))

MS/MS Fragmentation of **AYLPVAESFGFTADLR**
Found in **Tb10.70.2650**, elongation factor 2; Trypanosoma bruceichr 10Manual


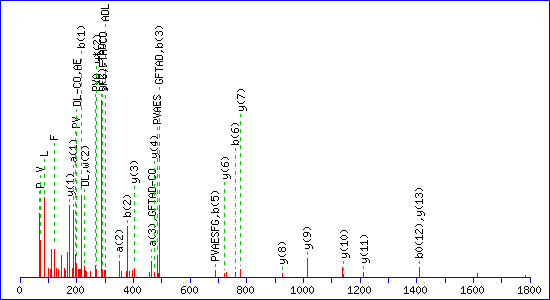


**MONOISOTOPIC mass of neutral peptide Mr(calc):** 1899.99

**Fixed modifications:** MMTS (C),(N-TERM)_iTRAQ,Lysine(K)_iTRAQ

**Ions Score:** 67 **Expect:** 2.4e-005

**Matches (Bold Red):** 45/244 fragment ions using 43 most intense peaks

| **#** | **Immon.** | **a** | **a^0^** | **b** | **b^0^** | **Seq.** | **v** | **w** | **w'** | **y** | **y*** | **y^0^** | **#** |
| --- | --- | --- | --- | --- | --- | --- | --- | --- | --- | --- | --- | --- | --- |
| **1** | 44.05 | **188.15** |  | **216.15** |  | **A** |  |  |  |  |  |  | **16** |
| **2** | 136.08 | **351.22** |  | **379.21** |  | **Y** | 1577.80 |  |  | 1685.85 | 1668.83 | 1667.84 | **15** |
| **3** | **86.10** | **464.30** |  | **492.29** |  | **L** | 1464.71 | 1463.72 |  | 1522.79 | 1505.76 | 1504.78 | **14** |
| **4** | **70.07** | 561.35 |  | 589.35 |  | **P** | 1367.66 | 1366.66 |  | **1409.71** | 1392.68 | 1391.70 | **13** |
| **5** | **72.08** | 660.42 |  | **688.42** |  | **V** | 1268.59 | 1281.61 |  | 1312.65 | 1295.63 | 1294.64 | **12** |
| **6** | 44.05 | 731.46 |  | **759.45** |  | **A** | 1197.55 |  |  | **1213.58** | 1196.56 | 1195.57 | **11** |
| **7** | 102.05 | 860.50 | 842.49 | 888.50 | 870.48 | **E** | 1068.51 | 1067.52 |  | **1142.55** | 1125.52 | 1124.54 | **10** |
| **8** | 60.04 | 947.53 | 929.52 | 975.53 | 957.52 | **S** | 981.48 | 980.48 |  | **1013.51** | 996.48 | 995.49 | **9** |
| **9** | **120.08** | 1094.60 | 1076.59 | 1122.60 | 1104.58 | **F** | 834.41 |  |  | **926.47** | 909.45 | 908.46 | **8** |
| **10** | 30.03 | 1151.62 | 1133.61 | 1179.62 | 1161.61 | **G** |  |  |  | **779.40** | 762.38 | 761.39 | **7** |
| **11** | **120.08** | 1298.69 | 1280.68 | 1326.69 | 1308.67 | **F** | 630.32 |  |  | **722.38** | 705.36 | 704.37 | **6** |
| **12** | 74.06 | 1399.74 | 1381.73 | 1427.73 | **1409.72** | **T** | 529.27 | 542.29 | 544.27 | 575.31 | 558.29 | 557.30 | **5** |
| **13** | 44.05 | 1470.78 | 1452.76 | 1498.77 | 1480.76 | **A** | 458.24 |  |  | **474.27** | 457.24 | 456.26 | **4** |
| **14** | 88.04 | 1585.80 | 1567.79 | 1613.80 | 1595.79 | **D** | 343.21 | 342.21 |  | **403.23** | 386.20 | 385.22 | **3** |
| **15** | **86.10** | 1698.89 | 1680.88 | 1726.88 | 1708.87 | **L** | 230.12 | **229.13** |  | **288.20** | **271.18** |  | **2** |
| **16** | 129.11 |  |  |  |  | **R** | 74.02 | 73.03 |  | **175.12** | 158.09 |  | **1** |

| **Seq** | **ya** | **yb** | **Seq** | **ya** | **yb** | **Seq** | **ya** | **yb** |
| --- | --- | --- | --- | --- | --- | --- | --- | --- |
| **YL** | 249.16 | 277.15 | **YLP** | 346.21 | 374.21 | **YLPV** | 445.28 | 473.28 |
| **YLPVA** | 516.32 | 544.31 | **YLPVAE** | 645.36 | 673.36 | **LP** | 183.15 | 211.14 |
| **LPV** | 282.22 | 310.21 | **LPVA** | 353.25 | 381.25 | **LPVAE** | 482.30 | 510.29 |
| **LPVAES** | 569.33 | 597.32 | **PV** | 169.13 | **197.13** | **PVA** | 240.17 | **268.17** |
| **PVAE** | 369.21 | 397.21 | **PVAES** | 456.25 | **484.24** | **PVAESF** | 603.31 | 631.31 |
| **PVAESFG** | 660.34 | **688.33** | **VA** | 143.12 | 171.11 | **VAE** | 272.16 | **300.16** |
| **VAES** | 359.19 | 387.19 | **VAESF** | 506.26 | 534.26 | **VAESFG** | 563.28 | 591.28 |
| **AE** | 173.09 | **201.09** | **AES** | 260.12 | **288.12** | **AESF** | 407.19 | 435.19 |
| **AESFG** | **464.21** | **492.21** | **AESFGF** | 611.28 | 639.28 | **ES** | 189.09 | 217.08 |
| **ESF** | 336.16 | 364.15 | **ESFG** | 393.18 | 421.17 | **ESFGF** | 540.25 | 568.24 |
| **ESFGFT** | 641.29 | 669.29 | **SF** | 207.11 | 235.11 | **SFG** | 264.13 | **292.13** |
| **SFGF** | 411.20 | 439.20 | **SFGFT** | 512.25 | 540.25 | **SFGFTA** | 583.29 | 611.28 |
| **SFGFTAD** | 698.31 | 726.31 | **FG** | 177.10 | 205.10 | **FGF** | 324.17 | 352.17 |
| **FGFT** | 425.22 | 453.21 | **FGFTA** | 496.26 | 524.25 | **FGFTAD** | 611.28 | 639.28 |
| **GF** | 177.10 | 205.10 | **GFT** | 278.15 | 306.14 | **GFTA** | 349.19 | 377.18 |
| **GFTAD** | **464.21** | **492.21** | **GFTADL** | 577.30 | 605.29 | **FT** | 221.13 | 249.12 |
| **FTA** | **292.17** | 320.16 | **FTAD** | 407.19 | 435.19 | **FTADL** | 520.28 | 548.27 |
| **TA** | 145.10 | 173.09 | **TAD** | 260.12 | **288.12** | **TADL** | 373.21 | 401.20 |
| **AD** | 159.08 | 187.07 | **ADL** | 272.16 | **300.16** | **DL** | **201.12** | **229.12** |

27. Tb10.70.7320

Match to: **Tb10.70.7320** Score: **67**

**hypothetical protein, conserved; Trypanosoma bruceichr 10Manual**

Nominal mass (M_r_): **213744**; Calculated pI value: **5.77**

NCBI BLAST search of [Tb10.70.7320](http://www.ncbi.nlm.nih.gov/blast/Blast.cgi?ALIGNMENTS=50&ALIGNMENT_VIEW=Pairwise&AUTO_FORMAT=Semiauto&CDD_SEARCH=on&CLIENT=web&COMPOSITION_BASED_STATISTICS=on&DATABASE=nr&DESCRIPTIONS=100&ENTREZ_QUERY=(none)&EXPECT=10&FILTER=L&FORMAT_BLOCK_ON_RESPAGE=None&FORMAT_OBJECT=Alignment&FORMAT_TYPE=HTML&GAPCOSTS=11+1&I_THRESH=0.001&LAYOUT=TwoWindows&MATRIX_NAME=BLOSUM62&NCBI_GI=on&PAGE=Proteins&PROGRAM=blastp&QUERY=Tb10.70.7320&SERVICE=plain&SET_DEFAULTS.x=21&SET_DEFAULTS.y=7&SHOW_OVERVIEW=on&WORD_SIZE=3&END_OF_HTTPGET=Yes) against nr

Unformatted [sequence string](../../../../D:%5CProteomic%20data%5C2010-1-8%5Ccgi%5Cgetseq.pl%3FTBA927_IPI+Tb10%2E70%2E7320+seq) for pasting into other applications

Fixed modifications: MMTS (C),(N-TERM)_iTRAQ,Lysine(K)_iTRAQ

Variable modifications: Oxidation (M)

Cleavage by Trypsin: cuts C-term side of KR unless next residue is P

Sequence Coverage: **2%**

Matched peptides shown in **Bold Red**

**1** MIGTRRSTKS TSFQHLYGSQ NRTLRGANGQ KGFSHQRNGT AR**EEQQSTKP**

**51 PGPR**NSVLIN RVVDVLPLFS INGTVRYIEA VGGRSAWTAE ADGCIRVRSV

**101** PKGTEVRTLE GREGCFCTCI LYVESTNAMW TTFSDGFIRV FDFNTHALLS

**151** EFIKHDKAIE CIWEVEGYVF TGGRDWQIYQ WKPDTYVCER RLSGHNNTVR

**201** CLCQYAGDTG AVLFSGSDDG TVRAWDPYLP ARKGKDGGPM MHIFTGHTQG

**251** VLSLELVTVS NQLWSGGEDT TIRVYDLQTL GCVSVLKHHR APVSSIKLIG

**301** IRVWSADKYG KTLLWDPKTL TPLKDLTTHM ATDPGSMLSI CKVK**QLVSWK**

**351** VWTACSGGKI YCWNADSIPI AFDGPTAGK**D AVDVSDAVQL LTENKGLR**GE

**401** IEKLRRQLMG EAEDGGSSAN RGSASVSPSR AKSVPSHPRH HESKMGRGTS

**451** PSATPPGKRR SPPAFSAEYA NDTPIHERAN TASREGDDAR GCTTCHERFF

**501** YGDAWEFIFR QDTEELCAVI RHEVATVLGI PADQVQITQM ELGSAGQGCV

**551** VKLNIQHSAS LPPSEIDTML SNYPFIDVYE LYGRYLAKKY GDPNEVHVSE

**601** SGSGTLGGQV HRSLCVDDSR GEEEMANGYD DPTPLQNMIN TLLKENEELR

**651** GQLMPKAETP PDENNAEVRT ALTNYEEERM KPRIAQLEKA LAERDETLHR

**701** KDERIRQLER ELEATKSDTG MKEVMKDLRK REKKLLTEVE ALTSQVEAME

**751** EDKRRAEDDA SFYRRENELL RNRISAMNEQ MAKASGSEAE EMQKVLTSYE

**801** EENVKPRIAR LEEVIAEKDE NLQSRDEKIK KLEDELEAAK QEAKFASGES

**851** VKSNGKAGSE TETSLLTEVQ VLRDQVEAME EDKRRVEDDA SFYRRENELL

**901** RNRISAMNEQ MAKASGSEAE EMQKVLTSYE EENVKPRIAR LEEAVSQRDE

**951** VLRSQDERIK ELTREIEENR REDKKGSYHV TDEAVVASKE EVQALKNQMK

**1001** AMKKEKEKLE NESKLYRKEN ESLKERLSET NDQLKKSSPL HEEEKQKVLS

**1051** RYEEENMKAR VARLEEAVTQ RDEALRAKSE RIRQLEKELR AAHREVKAAL

**1101** EESKKSSSRL HSDSTQTSAE ELRSLMTKAR EREKEKLKNE SKLYRKENES

**1151** LKERLSETDD QLKKSSSLDE EEKQKVLSRY EEEDVKPRVA RLEEAVTQRD

**1201** EALRAKDERI RQLEKELRAA HREAKSALED GRRNSSRLHS DSTQTSAEEL

**1251** RSLKTKMDEM ENDKRRLNEE IVLLRKENET LKRNLGDVMR RLKNPSSFIA

**1301** SEKQKLLSSY EEEHVKPRIT RLEHAVTLRD ERLQAKEDRI R**QLERELDAL**

**1351 R**YGGKNDVDN NAVEDGKTFR QLEEALLVEI QGVKDQMRAM AKSNQKMQEE

**1401** AVCLMRENQM LRDRLAEACE QLGKTPSIEA RETSRELVKC EDVSPNPRIA

**1451** QLEQAVTERD EILHTKDERI RQLERELEMF QHENRAICGN RDCRDAGRKL

**1501** AAENQELRNL VKYIENDKQK LEHEASFFKH ENDALIGRIA ELESTPRAPS

**1551** LSPTKTHTLS GDREALMSRL GTAIEQVHNV QKEREAAERK IAVLQEDCRD

**1601** LRMRLQHASD TPVRNEALFP SSTNCDGTYQ TGLHSGEDAH LIESARGAPH

**1651** SSSDAIVELL PDRLTALEDT MASLNTGLHD AIRRLSSVSD NSALLRTQIA

**1701** DMLSQRSSHN LITDPHERS

**Start - End Observed Mr(expt) Mr(calc) Delta Miss Sequence**

**43 - 54 1641.86 1640.86 1640.78 0.07 0 R.EEQQSTKPPGPR.N**  ([Ions score 67](../../../../D:%5CProteomic%20data%5C2010-1-8%5CZQ%5C1147.htm))

**345 - 350 1048.57 1047.56 1047.54 0.02 0 K.QLVSWK.V**  ([Ions score 9](../../../../D:%5CProteomic%20data%5C2010-1-8%5CZQ%5C1145.htm))

**380 - 398 2331.27 2330.26 2330.18 0.08 1 K.DAVDVSDAVQLLTENKGLR.G**  ([Ions score 0](../../../../D:%5CProteomic%20data%5C2010-1-8%5CZQ%5C1148.htm))

**1342 - 1351 1386.79 1385.78 1385.78 0.01 1 R.QLERELDALR.Y**  ([Ions score 9](../../../../D:%5CProteomic%20data%5C2010-1-8%5CZQ%5C1146.htm))

MS/MS Fragmentation of **EEQQSTKPPGPR**
Found in **Tb10.70.7320**, hypothetical protein, conserved; Trypanosoma bruceichr 10Manual


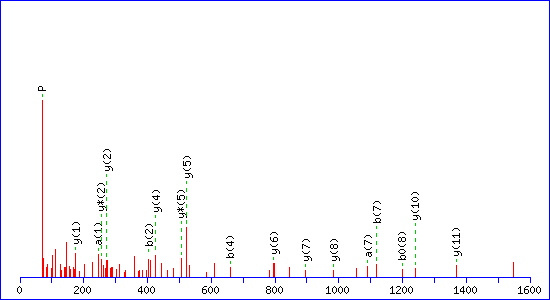


**MONOISOTOPIC mass of neutral peptide Mr(calc):** 1640.78

**Fixed modifications:** MMTS (C),(N-TERM)_iTRAQ,Lysine(K)_iTRAQ

**Ions Score:** 67 **Expect:** 2.7e-005

**Matches (Bold Red):** 20/183 fragment ions using 26 most intense peaks

| **#** | **Immon.** | **a** | **a*** | **a^0^** | **b** | **b*** | **b^0^** | **Seq.** | **v** | **w** | **w'** | **y** | **y*** | **y^0^** | **#** |
| --- | --- | --- | --- | --- | --- | --- | --- | --- | --- | --- | --- | --- | --- | --- | --- |
| **1** | 102.05 | **246.16** |  | 228.15 | 274.15 |  | 256.14 | **E** |  |  |  |  |  |  | **12** |
| **2** | 102.05 | 375.20 |  | 357.19 | **403.19** |  | 385.18 | **E** | 1294.61 | 1293.61 |  | **1368.65** | 1351.62 | 1350.63 | **11** |
| **3** | 101.07 | 503.26 | 486.23 | 485.25 | 531.25 | 514.23 | 513.24 | **Q** | 1166.55 | 1165.55 |  | **1239.60** | 1222.58 | 1221.59 | **10** |
| **4** | 101.07 | 631.32 | 614.29 | 613.31 | **659.31** | 642.29 | 641.30 | **Q** | 1038.49 | 1037.50 |  | 1111.54 | 1094.52 | 1093.53 | **9** |
| **5** | 60.04 | 718.35 | 701.32 | 700.34 | 746.34 | 729.32 | 728.33 | **S** | 951.46 | 950.46 |  | **983.49** | 966.46 | 965.48 | **8** |
| **6** | 74.06 | 819.40 | 802.37 | 801.39 | 847.39 | 830.37 | 829.38 | **T** | 850.41 | 863.43 | 865.41 | **896.45** | 879.43 | 878.44 | **7** |
| **7** | 245.12 | **1091.50** | 1074.48 | 1073.49 | **1119.50** | 1102.47 | 1101.49 | **K** | 578.30 | 577.31 |  | **795.41** | 778.38 |  | **6** |
| **8** | **70.07** | 1188.56 | 1171.53 | 1170.55 | 1216.55 | 1199.53 | **1198.54** | **P** | 481.25 | 480.26 |  | **523.30** | **506.27** |  | **5** |
| **9** | **70.07** | 1285.61 | 1268.58 | 1267.60 | 1313.60 | 1296.58 | 1295.59 | **P** | 384.20 | 383.20 |  | **426.25** | 409.22 |  | **4** |
| **10** | 30.03 | 1342.63 | 1325.60 | 1324.62 | 1370.63 | 1353.60 | 1352.62 | **G** |  |  |  | 329.19 | 312.17 |  | **3** |
| **11** | **70.07** | 1439.68 | 1422.66 | 1421.67 | 1467.68 | 1450.65 | 1449.67 | **P** | 230.12 | 229.13 |  | **272.17** | **255.15** |  | **2** |
| **12** | 129.11 |  |  |  |  |  |  | **R** | 74.02 | 73.03 |  | **175.12** | 158.09 |  | **1** |

| **Seq** | **ya** | **yb** | **Seq** | **ya** | **yb** | **Seq** | **ya** | **yb** |
| --- | --- | --- | --- | --- | --- | --- | --- | --- |
| **EQ** | 230.11 | 258.11 | **EQQ** | 358.17 | 386.17 | **EQQS** | 445.20 | 473.20 |
| **EQQST** | 546.25 | 574.25 | **QQ** | 229.13 | 257.12 | **QQS** | 316.16 | 344.16 |
| **QQST** | 417.21 | 445.20 | **QQSTK** | 689.32 | 717.31 | **QS** | 188.10 | 216.10 |
| **QST** | 289.15 | 317.15 | **QSTK** | 561.26 | 589.25 | **QSTKP** | 658.31 | 686.31 |
| **ST** | 161.09 | 189.09 | **STK** | 433.20 | 461.19 | **STKP** | 530.25 | 558.25 |
| **STKPP** | 627.30 | 655.30 | **STKPPG** | 684.33 | 712.32 | **TK** | 346.17 | 374.16 |
| **TKP** | 443.22 | 471.22 | **TKPP** | 540.27 | 568.27 | **TKPPG** | 597.29 | 625.29 |
| **TKPPGP** | 694.35 | 722.34 | **KP** | 342.17 | 370.17 | **KPP** | 439.23 | 467.22 |
| **KPPG** | 496.25 | 524.24 | **KPPGP** | 593.30 | 621.29 | **PP** | 167.12 | 195.11 |
| **PPG** | 224.14 | 252.13 | **PPGP** | 321.19 | 349.19 | **PG** | 127.09 | 155.08 |
| **PGP** | 224.14 | 252.13 | **GP** | 127.09 | 155.08 |  |  |  |

28. Tb11.02.0352

Match to: **Tb11.02.0352** Score: **66**

**hypothetical protein, conserved; Trypanosoma bruceichr 11Manual**

Nominal mass (M_r_): **34786**; Calculated pI value: **9.01**

NCBI BLAST search of [Tb11.02.0352](http://www.ncbi.nlm.nih.gov/blast/Blast.cgi?ALIGNMENTS=50&ALIGNMENT_VIEW=Pairwise&AUTO_FORMAT=Semiauto&CDD_SEARCH=on&CLIENT=web&COMPOSITION_BASED_STATISTICS=on&DATABASE=nr&DESCRIPTIONS=100&ENTREZ_QUERY=(none)&EXPECT=10&FILTER=L&FORMAT_BLOCK_ON_RESPAGE=None&FORMAT_OBJECT=Alignment&FORMAT_TYPE=HTML&GAPCOSTS=11+1&I_THRESH=0.001&LAYOUT=TwoWindows&MATRIX_NAME=BLOSUM62&NCBI_GI=on&PAGE=Proteins&PROGRAM=blastp&QUERY=MFSRRRKVAVVAERPSLTWHLMREEYHPIFVTDSSERIEYWYNKLATQHQRENFRRICKEISQQDPERGLPKMSALYSLQYCICEADSMIRHYGFMFSVLGKKLARLWIMHDASHRDKNTFTELFTGLQTDFIPKSVMKTDFQPPEQGDYEGIDRSHFLSSIDWFNPESKRNLPRVDIQKFGNKIASTRASLPVKKPKNQFDGVSPLAVLGYTGDLDTEIEKLREKQQKSEEAERYYVEDGCTVYVAGRKENRRAATLSKNRVIATFYGD&SERVICE=plain&SET_DEFAULTS.x=9&SET_DEFAULTS.y=5&SHOW_OVERVIEW=on&WORD_SIZE=3&END_OF_HTTPGET=Yes) against nr

Unformatted [sequence string](../../../../D:%5CProteomic%20data%5C2010-1-8%5Ccgi%5Cgetseq.pl%3FTBA927_IPI+Tb11%2E02%2E0352+seq) for pasting into other applications

Fixed modifications: MMTS (C),(N-TERM)_iTRAQ,Lysine(K)_iTRAQ

Variable modifications: Oxidation (M)

Cleavage by Trypsin: cuts C-term side of KR unless next residue is P

Sequence Coverage: **5%**

Matched peptides shown in **Bold Red**

**1** MFSRRRKVAV VAERPSLTWH LMREEYHPIF VTDSSERIEY WYNKLATQHQ

**51** RENFRRICKE ISQQDPERGL PKMSALYSLQ YCICEADSMI RHYGFMFSVL

**101** GKKLARLWIM HDASHRDKNT FTELFTGLQT DFIPKSVMK**T DFQPPEQGDY**

**151 EGIDR**SHFLS SIDWFNPESK RNLPRVDIQK FGNKIASTRA SLPVKKPKNQ

**201** FDGVSPLAVL GYTGDLDTEI EKLREKQQKS EEAERYYVED GCTVYVAGRK

**251** ENRRAATLSK NRVIATFYGD

MS/MS Fragmentation of **TDFQPPEQGDYEGIDR**
Found in **Tb11.02.0352**, hypothetical protein, conserved; Trypanosoma bruceichr 11Manual


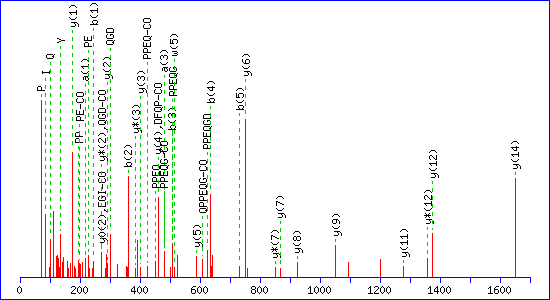


**MONOISOTOPIC mass of neutral peptide Mr(calc):** 2009.91

**Fixed modifications:** MMTS (C),(N-TERM)_iTRAQ,Lysine(K)_iTRAQ

**Ions Score:** 66 **Expect:** 2.5e-005

**Matches (Bold Red):** 46/276 fragment ions using 53 most intense peaks

| **#** | **Immon.** | **a** | **a*** | **a^0^** | **b** | **b*** | **b^0^** | **Seq.** | **v** | **w** | **w'** | **y** | **y*** | **y^0^** | **#** |
| --- | --- | --- | --- | --- | --- | --- | --- | --- | --- | --- | --- | --- | --- | --- | --- |
| **1** | 74.06 | **218.16** |  | 200.15 | **246.16** |  | 228.15 | **T** |  |  |  |  |  |  | **16** |
| **2** | 88.04 | 333.19 |  | 315.18 | **361.18** |  | 343.17 | **D** | 1705.75 | 1704.75 |  | 1765.77 | 1748.74 | 1747.76 | **15** |
| **3** | 120.08 | **480.26** |  | 462.25 | **508.25** |  | 490.24 | **F** | 1558.68 |  |  | **1650.74** | 1633.71 | 1632.73 | **14** |
| **4** | **101.07** | 608.32 | 591.29 | 590.31 | **636.31** | 619.28 | 618.30 | **Q** | 1430.62 | 1429.62 |  | 1503.67 | 1486.64 | 1485.66 | **13** |
| **5** | **70.07** | 705.37 | 688.34 | 687.36 | **733.36** | 716.34 | 715.35 | **P** | 1333.57 | 1332.57 |  | **1375.61** | **1358.59** | 1357.60 | **12** |
| **6** | **70.07** | 802.42 | 785.40 | 784.41 | 830.42 | 813.39 | 812.41 | **P** | 1236.51 | 1235.52 |  | **1278.56** | 1261.53 | 1260.55 | **11** |
| **7** | 102.05 | 931.46 | 914.44 | 913.45 | 959.46 | 942.43 | 941.45 | **E** | 1107.47 | 1106.47 |  | 1181.51 | 1164.48 | 1163.50 | **10** |
| **8** | **101.07** | 1059.52 | 1042.50 | 1041.51 | 1087.52 | 1070.49 | 1069.51 | **Q** | 979.41 | 978.42 |  | **1052.46** | 1035.44 | 1034.45 | **9** |
| **9** | 30.03 | 1116.54 | 1099.52 | 1098.53 | 1144.54 | 1127.51 | 1126.53 | **G** |  |  |  | **924.41** | 907.38 | 906.40 | **8** |
| **10** | 88.04 | 1231.57 | 1214.54 | 1213.56 | 1259.57 | 1242.54 | 1241.56 | **D** | 807.36 | 806.37 |  | **867.38** | **850.36** | 849.37 | **7** |
| **11** | **136.08** | 1394.63 | 1377.61 | 1376.62 | 1422.63 | 1405.60 | 1404.62 | **Y** | 644.30 |  |  | **752.36** | 735.33 | 734.35 | **6** |
| **12** | 102.05 | 1523.68 | 1506.65 | 1505.67 | 1551.67 | 1534.65 | 1533.66 | **E** | 515.26 | **514.26** |  | **589.29** | 572.27 | 571.28 | **5** |
| **13** | 30.03 | 1580.70 | 1563.67 | 1562.69 | 1608.69 | 1591.67 | 1590.68 | **G** |  |  |  | **460.25** | 443.22 | 442.24 | **4** |
| **14** | **86.10** | 1693.78 | 1676.76 | 1675.77 | 1721.78 | 1704.75 | 1703.77 | **I** | 345.15 | 358.17 | 372.19 | **403.23** | **386.20** | 385.22 | **3** |
| **15** | 88.04 | 1808.81 | 1791.78 | 1790.80 | 1836.80 | 1819.78 | 1818.79 | **D** | 230.12 | 229.13 |  | **290.15** | **273.12** | **272.14** | **2** |
| **16** | 129.11 |  |  |  |  |  |  | **R** | 74.02 | 73.03 |  | **175.12** | 158.09 |  | **1** |

| **Seq** | **ya** | **yb** | **Seq** | **ya** | **yb** | **Seq** | **ya** | **yb** |
| --- | --- | --- | --- | --- | --- | --- | --- | --- |
| **DF** | 235.11 | 263.10 | **DFQ** | 363.17 | 391.16 | **DFQP** | **460.22** | 488.21 |
| **DFQPP** | 557.27 | 585.27 | **DFQPPE** | 686.31 | 714.31 | **FQ** | 248.14 | 276.13 |
| **FQP** | 345.19 | 373.19 | **FQPP** | 442.24 | 470.24 | **FQPPE** | 571.29 | 599.28 |
| **FQPPEQ** | 699.35 | 727.34 | **QP** | 198.12 | 226.12 | **QPP** | 295.18 | 323.17 |
| **QPPE** | **424.22** | **452.21** | **QPPEQ** | 552.28 | 580.27 | **QPPEQG** | **609.30** | 637.29 |
| **PP** | 167.12 | **195.11** | **PPE** | 296.16 | 324.16 | **PPEQ** | **424.22** | **452.21** |
| **PPEQG** | **481.24** | **509.24** | **PPEQGD** | 596.27 | **624.26** | **PE** | **199.11** | **227.10** |
| **PEQ** | 327.17 | 355.16 | **PEQG** | 384.19 | 412.18 | **PEQGD** | 499.21 | 527.21 |
| **PEQGDY** | 662.28 | 690.27 | **EQ** | 230.11 | 258.11 | **EQG** | 287.13 | 315.13 |
| **EQGD** | 402.16 | 430.16 | **EQGDY** | 565.23 | 593.22 | **EQGDYE** | 694.27 | 722.26 |
| **QG** | 158.09 | 186.09 | **QGD** | **273.12** | **301.11** | **QGDY** | 436.18 | 464.18 |
| **QGDYE** | 565.23 | 593.22 | **QGDYEG** | 622.25 | 650.24 | **GD** | 145.06 | 173.06 |
| **GDY** | 308.12 | 336.12 | **GDYE** | 437.17 | 465.16 | **GDYEG** | 494.19 | 522.18 |
| **GDYEGI** | 607.27 | 635.27 | **DY** | 251.10 | 279.10 | **DYE** | 380.15 | 408.14 |
| **DYEG** | 437.17 | 465.16 | **DYEGI** | 550.25 | 578.25 | **DYEGID** | 665.28 | 693.27 |
| **YE** | 265.12 | 293.11 | **YEG** | 322.14 | 350.13 | **YEGI** | 435.22 | 463.22 |
| **YEGID** | 550.25 | 578.25 | **EG** | 159.08 | 187.07 | **EGI** | **272.16** | 300.16 |
| **EGID** | 387.19 | 415.18 | **GI** | 143.12 | 171.11 | **GID** | 258.14 | 286.14 |
| **ID** | 201.12 | 229.12 |  |  |  |  |  |  |

29. Tb10.70.0800

Match to: **Tb10.70.0800** Score: **66**

**ZFPuniversal minicircle sequence binding protein (UMSBP), putative; Trypanosoma bruceichr 10Manual**

Nominal mass (M_r_): **15534**; Calculated pI value: **8.79**

NCBI BLAST search of [Tb10.70.0800](http://www.ncbi.nlm.nih.gov/blast/Blast.cgi?ALIGNMENTS=50&ALIGNMENT_VIEW=Pairwise&AUTO_FORMAT=Semiauto&CDD_SEARCH=on&CLIENT=web&COMPOSITION_BASED_STATISTICS=on&DATABASE=nr&DESCRIPTIONS=100&ENTREZ_QUERY=(none)&EXPECT=10&FILTER=L&FORMAT_BLOCK_ON_RESPAGE=None&FORMAT_OBJECT=Alignment&FORMAT_TYPE=HTML&GAPCOSTS=11+1&I_THRESH=0.001&LAYOUT=TwoWindows&MATRIX_NAME=BLOSUM62&NCBI_GI=on&PAGE=Proteins&PROGRAM=blastp&QUERY=MADNMQMSNARTCYNCGQPGHMSRECPNARSGGNMGGGRSCYNCGQPDHISRDCPNARTGGNMGGGRSCYNCGRPGHISRDCPNARSGGNMGGGRACYHCQQEGHIARECPNAPADAAAGGRACFNCGQPGHLSRACPVK&SERVICE=plain&SET_DEFAULTS.x=9&SET_DEFAULTS.y=5&SHOW_OVERVIEW=on&WORD_SIZE=3&END_OF_HTTPGET=Yes) against nr

Unformatted [sequence string](../../../../D:%5CProteomic%20data%5C2010-1-8%5Ccgi%5Cgetseq.pl%3FTBA927_IPI+Tb10%2E70%2E0800+seq) for pasting into other applications

Fixed modifications: MMTS (C),(N-TERM)_iTRAQ,Lysine(K)_iTRAQ

Variable modifications: Oxidation (M)

Cleavage by Trypsin: cuts C-term side of KR unless next residue is P

Sequence Coverage: **10%**

Matched peptides shown in **Bold Red**

**1** MADNMQMSNA RTCYNCGQPG HMSRECPNAR SGGNMGGGRS CYNCGQPDHI

**51** SRDCPNARTG GNMGGGRSCY NCGRPGHISR DCPNARSGGN MGGGRACYHC

**101** QQEGHIAR**EC PNAPADAAAG GR**ACFNCGQP GHLSRACPVK

MS/MS Fragmentation of **ECPNAPADAAAGGR**
Found in **Tb10.70.0800**, ZFPuniversal minicircle sequence binding protein (UMSBP), putative; Trypanosoma bruceichr 10Manual


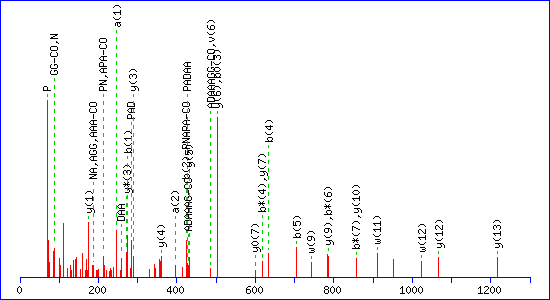


**MONOISOTOPIC mass of neutral peptide Mr(calc):** 1488.66

**Fixed modifications:** MMTS (C),(N-TERM)_iTRAQ,Lysine(K)_iTRAQ

**Ions Score:** 66 **Expect:** 3.4e-005

**Matches (Bold Red):** 43/246 fragment ions using 36 most intense peaks

| **#** | **Immon.** | **a** | **a*** | **a^0^** | **b** | **b*** | **b^0^** | **Seq.** | **v** | **w** | **y** | **y*** | **y^0^** | **#** |
| --- | --- | --- | --- | --- | --- | --- | --- | --- | --- | --- | --- | --- | --- | --- |
| **1** | 102.05 | **246.16** |  | 228.15 | **274.15** |  | 256.14 | **E** |  |  |  |  |  | **14** |
| **2** | 122.01 | **395.15** |  | 377.14 | **423.15** |  | 405.14 | **C** | 1122.53 | 1121.53 | **1216.52** | 1199.49 | 1198.51 | **13** |
| **3** | **70.07** | 492.21 |  | 474.20 | 520.20 |  | **502.19** | **P** | 1025.48 | **1024.48** | **1067.52** | 1050.50 | 1049.51 | **12** |
| **4** | **87.06** | 606.25 | 589.22 | 588.24 | **634.24** | **617.22** | 616.23 | **N** | 911.43 | **910.44** | 970.47 | 953.44 | 952.46 | **11** |
| **5** | 44.05 | 677.29 | 660.26 | 659.28 | **705.28** | 688.26 | 687.27 | **A** | 840.40 |  | **856.43** | 839.40 | 838.42 | **10** |
| **6** | **70.07** | 774.34 | 757.31 | 756.33 | 802.33 | **785.31** | 784.32 | **P** | 743.34 | **742.35** | **785.39** | 768.36 | 767.38 | **9** |
| **7** | 44.05 | 845.38 | 828.35 | 827.37 | 873.37 | **856.35** | 855.36 | **A** | 672.31 |  | 688.34 | 671.31 | 670.33 | **8** |
| **8** | 88.04 | 960.40 | 943.38 | 942.39 | 988.40 | 971.37 | 970.39 | **D** | 557.28 | 556.28 | **617.30** | 600.27 | **599.29** | **7** |
| **9** | 44.05 | 1031.44 | 1014.41 | 1013.43 | 1059.44 | 1042.41 | 1041.43 | **A** | **486.24** |  | **502.27** | 485.25 |  | **6** |
| **10** | 44.05 | 1102.48 | 1085.45 | 1084.47 | 1130.47 | 1113.45 | 1112.46 | **A** | 415.20 |  | **431.24** | 414.21 |  | **5** |
| **11** | 44.05 | 1173.52 | 1156.49 | 1155.50 | 1201.51 | 1184.48 | 1183.50 | **A** | 344.17 |  | **360.20** | 343.17 |  | **4** |
| **12** | 30.03 | 1230.54 | 1213.51 | 1212.53 | 1258.53 | 1241.51 | 1240.52 | **G** |  |  | **289.16** | **272.14** |  | **3** |
| **13** | 30.03 | 1287.56 | 1270.53 | 1269.55 | 1315.55 | 1298.53 | 1297.54 | **G** |  |  | 232.14 | 215.11 |  | **2** |
| **14** | 129.11 |  |  |  |  |  |  | **R** | 74.02 | 73.03 | **175.12** | 158.09 |  | **1** |

| **Seq** | **ya** | **yb** | **Seq** | **ya** | **yb** | **Seq** | **ya** | **yb** |
| --- | --- | --- | --- | --- | --- | --- | --- | --- |
| **CP** | 219.06 | 247.06 | **CPN** | 333.10 | 361.10 | **CPNA** | 404.14 | 432.14 |
| **CPNAP** | 501.19 | 529.19 | **CPNAPA** | 572.23 | 600.23 | **CPNAPAD** | 687.26 | 715.25 |
| **PN** | 184.11 | **212.10** | **PNA** | 255.15 | 283.14 | **PNAP** | 352.20 | 380.19 |
| **PNAPA** | **423.24** | 451.23 | **PNAPAD** | 538.26 | 566.26 | **PNAPADA** | 609.30 | 637.29 |
| **PNAPADAA** | 680.34 | 708.33 | **NA** | 158.09 | **186.09** | **NAP** | 255.15 | 283.14 |
| **NAPA** | 326.18 | 354.18 | **NAPAD** | 441.21 | 469.20 | **NAPADA** | 512.25 | 540.24 |
| **NAPADAA** | 583.28 | 611.28 | **NAPADAAA** | 654.32 | 682.32 | **AP** | 141.10 | 169.10 |
| **APA** | **212.14** | 240.13 | **APAD** | 327.17 | 355.16 | **APADA** | 398.20 | **426.20** |
| **APADAA** | 469.24 | 497.24 | **APADAAA** | 540.28 | 568.27 | **APADAAAG** | 597.30 | 625.29 |
| **APADAAAGG** | 654.32 | 682.32 | **PA** | 141.10 | 169.10 | **PAD** | 256.13 | **284.12** |
| **PADA** | 327.17 | 355.16 | **PADAA** | 398.20 | **426.20** | **PADAAA** | 469.24 | 497.24 |
| **PADAAAG** | 526.26 | 554.26 | **PADAAAGG** | 583.28 | 611.28 | **AD** | 159.08 | 187.07 |
| **ADA** | 230.11 | **258.11** | **ADAA** | 301.15 | 329.15 | **ADAAA** | 372.19 | 400.18 |
| **ADAAAG** | **429.21** | 457.20 | **ADAAAGG** | **486.23** | 514.23 | **DA** | 159.08 | 187.07 |
| **DAA** | 230.11 | **258.11** | **DAAA** | 301.15 | 329.15 | **DAAAG** | 358.17 | 386.17 |
| **DAAAGG** | 415.19 | 443.19 | **AA** | 115.09 | 143.08 | **AAA** | **186.12** | 214.12 |
| **AAAG** | 243.15 | 271.14 | **AAAGG** | 300.17 | 328.16 | **AA** | 115.09 | 143.08 |
| **AAG** | 172.11 | 200.10 | **AAGG** | 229.13 | 257.12 | **AG** | 101.07 | 129.07 |
| **AGG** | 158.09 | **186.09** | **GG** | **87.06** | 115.05 |  |  |  |

30. Tb10.61.1630

Match to: **Tb10.61.1630** Score: **66**

**ZC3H40RNA binding protein, putative; Trypanosoma bruceichr 10Manual**

Nominal mass (M_r_): **50082**; Calculated pI value: **7.52**

NCBI BLAST search of [Tb10.61.1630](http://www.ncbi.nlm.nih.gov/blast/Blast.cgi?ALIGNMENTS=50&ALIGNMENT_VIEW=Pairwise&AUTO_FORMAT=Semiauto&CDD_SEARCH=on&CLIENT=web&COMPOSITION_BASED_STATISTICS=on&DATABASE=nr&DESCRIPTIONS=100&ENTREZ_QUERY=(none)&EXPECT=10&FILTER=L&FORMAT_BLOCK_ON_RESPAGE=None&FORMAT_OBJECT=Alignment&FORMAT_TYPE=HTML&GAPCOSTS=11+1&I_THRESH=0.001&LAYOUT=TwoWindows&MATRIX_NAME=BLOSUM62&NCBI_GI=on&PAGE=Proteins&PROGRAM=blastp&QUERY=MYGNQSSGGPETPPLVWNVAKVLQDGFNNHRSSLPQLLGIAHNLEETLENTRLMYNKAVKERDELRNKLVAAQNSIVAVKRVVEQYATVNEPVVASDGFTYESELIRDYLRECASSQTKAYSQLTKEELLDVLVPNQTLSRLVKMLQQVCPMDVPPVSVRTPIPPFKPLQQGEVGSKGVGSNLNWAGDERGPGTSAIQASDVETAMVSAATTPGVSGVHSSNAQAVTSNPNGATARRWENRQAQQPQVSGFTGKNSNRKHPCLRVYGRCNFLEDCAFANYPYGACLNYIKGKCRFGQHCKELHVSSAYPRYGDQRGGGGINSGNNTANNSTANADIATPTATGRGGSGANPTTTPRSEEKGQSTSTKAKKGARESSKCSSVKTDACAGGDVEAEAVKELKAAPPDTSPQEATGGERTEREVMEAPEPSEGST&SERVICE=plain&SET_DEFAULTS.x=9&SET_DEFAULTS.y=5&SHOW_OVERVIEW=on&WORD_SIZE=3&END_OF_HTTPGET=Yes) against nr

Unformatted [sequence string](../../../../D:%5CProteomic%20data%5C2010-1-8%5Ccgi%5Cgetseq.pl%3FTBA927_IPI+Tb10%2E61%2E1630+seq) for pasting into other applications

Fixed modifications: MMTS (C),(N-TERM)_iTRAQ,Lysine(K)_iTRAQ

Variable modifications: Oxidation (M)

Cleavage by Trypsin: cuts C-term side of KR unless next residue is P

Sequence Coverage: **3%**

Matched peptides shown in **Bold Red**

**1** MYGNQSSGGP ETPPLVWNVA KVLQDGFNNH RSSLPQLLGI AHNLEETLEN

**51** TRLMYNKAVK ERDELRNKLV AAQNSIVAVK RVVEQYATVN EPVVASDGFT

**101** YESELIRDYL RECASSQTKA YSQLTKEELL DVLVPNQTLS RLVKMLQQVC

**151** PMDVPPVSVR TPIPPFKPLQ QGEVGSKGVG SNLNWAGDER GPGTSAIQAS

**201** DVETAMVSAA TTPGVSGVHS SNAQAVTSNP NGATARRWEN RQAQQPQVSG

**251** FTGKNSNRKH PCLRVYGRCN FLEDCAFANY PYGACLNYIK GKCRFGQHCK

**301** ELHVSSAYPR YGDQRGGGGI NSGNNTANNS TANADIATPT ATGRGGSGAN

**351** PTTTPRSEEK GQSTSTKAKK GARESSKCSS VKTDACAGGD VEAEAVKELK

**401** **AAPPDTSPQE ATGGER**TERE VMEAPEPSEG ST

MS/MS Fragmentation of **AAPPDTSPQEATGGER**
Found in **Tb10.61.1630**, ZC3H40RNA binding protein, putative; Trypanosoma bruceichr 10Manual


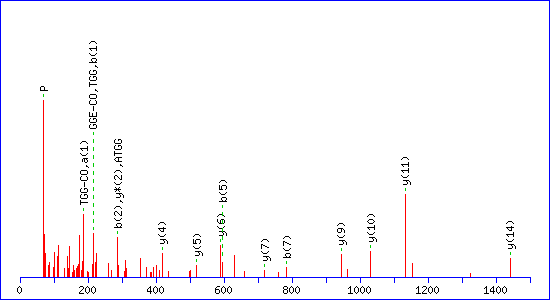


**MONOISOTOPIC mass of neutral peptide Mr(calc):** 1726.82

**Fixed modifications:** MMTS (C),(N-TERM)_iTRAQ,Lysine(K)_iTRAQ

**Ions Score:** 66 **Expect:** 3.5e-005

**Matches (Bold Red):** 21/272 fragment ions using 18 most intense peaks

| **#** | **Immon.** | **a** | **a*** | **a^0^** | **b** | **b*** | **b^0^** | **Seq.** | **v** | **w** | **w'** | **y** | **y*** | **y^0^** | **#** |
| --- | --- | --- | --- | --- | --- | --- | --- | --- | --- | --- | --- | --- | --- | --- | --- |
| **1** | 44.05 | **188.15** |  |  | **216.15** |  |  | **A** |  |  |  |  |  |  | **16** |
| **2** | 44.05 | 259.19 |  |  | **287.18** |  |  | **A** | 1496.66 |  |  | 1512.69 | 1495.67 | 1494.68 | **15** |
| **3** | **70.07** | 356.24 |  |  | 384.24 |  |  | **P** | 1399.61 | 1398.61 |  | **1441.66** | 1424.63 | 1423.64 | **14** |
| **4** | **70.07** | 453.29 |  |  | 481.29 |  |  | **P** | 1302.56 | 1301.56 |  | 1344.60 | 1327.58 | 1326.59 | **13** |
| **5** | 88.04 | 568.32 |  | 550.31 | **596.32** |  | 578.31 | **D** | 1187.53 | 1186.53 |  | 1247.55 | 1230.52 | 1229.54 | **12** |
| **6** | 74.06 | 669.37 |  | 651.36 | 697.36 |  | 679.35 | **T** | 1086.48 | 1099.50 | 1101.48 | **1132.52** | 1115.50 | 1114.51 | **11** |
| **7** | 60.04 | 756.40 |  | 738.39 | **784.40** |  | 766.39 | **S** | 999.45 | 998.45 |  | **1031.48** | 1014.45 | 1013.46 | **10** |
| **8** | **70.07** | 853.45 |  | 835.44 | 881.45 |  | 863.44 | **P** | 902.40 | 901.40 |  | **944.44** | 927.42 | 926.43 | **9** |
| **9** | 101.07 | 981.51 | 964.49 | 963.50 | 1009.51 | 992.48 | 991.50 | **Q** | 774.34 | 773.34 |  | 847.39 | 830.36 | 829.38 | **8** |
| **10** | 102.05 | 1110.56 | 1093.53 | 1092.54 | 1138.55 | 1121.52 | 1120.54 | **E** | 645.30 | 644.30 |  | **719.33** | 702.31 | 701.32 | **7** |
| **11** | 44.05 | 1181.59 | 1164.57 | 1163.58 | 1209.59 | 1192.56 | 1191.58 | **A** | 574.26 |  |  | **590.29** | 573.26 | 572.28 | **6** |
| **12** | 74.06 | 1282.64 | 1265.61 | 1264.63 | 1310.63 | 1293.61 | 1292.62 | **T** | 473.21 | 486.23 | 488.21 | **519.25** | 502.23 | 501.24 | **5** |
| **13** | 30.03 | 1339.66 | 1322.63 | 1321.65 | 1367.66 | 1350.63 | 1349.65 | **G** |  |  |  | **418.20** | 401.18 | 400.19 | **4** |
| **14** | 30.03 | 1396.68 | 1379.66 | 1378.67 | 1424.68 | 1407.65 | 1406.67 | **G** |  |  |  | 361.18 | 344.16 | 343.17 | **3** |
| **15** | 102.05 | 1525.73 | 1508.70 | 1507.71 | 1553.72 | 1536.69 | 1535.71 | **E** | 230.12 | 229.13 |  | 304.16 | **287.13** | 286.15 | **2** |
| **16** | 129.11 |  |  |  |  |  |  | **R** | 74.02 | 73.03 |  | 175.12 | 158.09 |  | **1** |

| **Seq** | **ya** | **yb** | **Seq** | **ya** | **yb** | **Seq** | **ya** | **yb** |
| --- | --- | --- | --- | --- | --- | --- | --- | --- |
| **AP** | 141.10 | 169.10 | **APP** | 238.15 | 266.15 | **APPD** | 353.18 | 381.18 |
| **APPDT** | 454.23 | 482.22 | **APPDTS** | 541.26 | 569.26 | **APPDTSP** | 638.31 | 666.31 |
| **PP** | 167.12 | 195.11 | **PPD** | 282.14 | 310.14 | **PPDT** | 383.19 | 411.19 |
| **PPDTS** | 470.22 | 498.22 | **PPDTSP** | 567.28 | 595.27 | **PPDTSPQ** | 695.34 | 723.33 |
| **PD** | 185.09 | 213.09 | **PDT** | 286.14 | 314.13 | **PDTS** | 373.17 | 401.17 |
| **PDTSP** | 470.22 | 498.22 | **PDTSPQ** | 598.28 | 626.28 | **DT** | 189.09 | 217.08 |
| **DTS** | 276.12 | 304.11 | **DTSP** | 373.17 | 401.17 | **DTSPQ** | 501.23 | 529.23 |
| **DTSPQE** | 630.27 | 658.27 | **TS** | 161.09 | 189.09 | **TSP** | 258.14 | 286.14 |
| **TSPQ** | 386.20 | 414.20 | **TSPQE** | 515.25 | 543.24 | **TSPQEA** | 586.28 | 614.28 |
| **TSPQEAT** | 687.33 | 715.33 | **SP** | 157.10 | 185.09 | **SPQ** | 285.16 | 313.15 |
| **SPQE** | 414.20 | 442.19 | **SPQEA** | 485.24 | 513.23 | **SPQEAT** | 586.28 | 614.28 |
| **SPQEATG** | 643.30 | 671.30 | **PQ** | 198.12 | 226.12 | **PQE** | 327.17 | 355.16 |
| **PQEA** | 398.20 | 426.20 | **PQEAT** | 499.25 | 527.25 | **PQEATG** | 556.27 | 584.27 |
| **PQEATGG** | 613.29 | 641.29 | **QE** | 230.11 | 258.11 | **QEA** | 301.15 | 329.15 |
| **QEAT** | 402.20 | 430.19 | **QEATG** | 459.22 | 487.21 | **QEATGG** | 516.24 | 544.24 |
| **QEATGGE** | 645.28 | 673.28 | **EA** | 173.09 | 201.09 | **EAT** | 274.14 | 302.13 |
| **EATG** | 331.16 | 359.16 | **EATGG** | 388.18 | 416.18 | **EATGGE** | 517.23 | 545.22 |
| **AT** | 145.10 | 173.09 | **ATG** | 202.12 | 230.11 | **ATGG** | 259.14 | **287.13** |
| **ATGGE** | 388.18 | 416.18 | **TG** | 131.08 | 159.08 | **TGG** | **188.10** | **216.10** |
| **TGGE** | 317.15 | 345.14 | **GG** | 87.06 | 115.05 | **GGE** | **216.10** | 244.09 |
| **GE** | 159.08 | 187.07 |  |  |  |  |  |  |

31. Tb11.01.0390

Match to: **Tb11.01.0390** Score: **65**

**dynein heavy chain, putative; Trypanosoma bruceichr 11Manual**

Nominal mass (M_r_): **510592**; Calculated pI value: **5.47**

NCBI BLAST search of [Tb11.01.0390](http://www.ncbi.nlm.nih.gov/blast/Blast.cgi?ALIGNMENTS=50&ALIGNMENT_VIEW=Pairwise&AUTO_FORMAT=Semiauto&CDD_SEARCH=on&CLIENT=web&COMPOSITION_BASED_STATISTICS=on&DATABASE=nr&DESCRIPTIONS=100&ENTREZ_QUERY=(none)&EXPECT=10&FILTER=L&FORMAT_BLOCK_ON_RESPAGE=None&FORMAT_OBJECT=Alignment&FORMAT_TYPE=HTML&GAPCOSTS=11+1&I_THRESH=0.001&LAYOUT=TwoWindows&MATRIX_NAME=BLOSUM62&NCBI_GI=on&PAGE=Proteins&PROGRAM=blastp&QUERY=Tb11.01.0390&SERVICE=plain&SET_DEFAULTS.x=21&SET_DEFAULTS.y=7&SHOW_OVERVIEW=on&WORD_SIZE=3&END_OF_HTTPGET=Yes) against nr

Unformatted [sequence string](../../../../D:%5CProteomic%20data%5C2010-1-8%5Ccgi%5Cgetseq.pl%3FTBA927_IPI+Tb11%2E01%2E0390+seq) for pasting into other applications

Fixed modifications: MMTS (C),(N-TERM)_iTRAQ,Lysine(K)_iTRAQ

Variable modifications: Oxidation (M)

Cleavage by Trypsin: cuts C-term side of KR unless next residue is P

Sequence Coverage: **3%**

Matched peptides shown in **Bold Red**

**1** MLQHFLRPEV LGTTGKDEEN GTKDHQPGQQ RPTTSRLDSQ SLTATDTVSE

**51** RPKTAEPLDL VSISATYKET AVLFPDAHQA AFEPK**VQVPF EFQR**GRIPRE

**101** IEIERRRRLY ESKDVSRLVQ VAGLTLKLLA HKSSQELPLQ VFDDTSYDSR

**151** IPAEWMEIAA QNENPAGR**YL PAEGIYEFMN SDFR**MRPCRV IGWDVARNEV

**201** KLLWGAKPVP DETPVVVPRF HVRLLAEDPV VYVERLVNAQ KQRVKAMAWI

**251** RYRLCCDAMP TDGLPGLDSN LSDRLLRLGT GIPNLNKAVF PDVDERAQRL

**301** IAELTLEWQR SHNRILLQDL MQRDESTLRM VANTTQMSLQ ELVRGPNVEI

**351** RRTIKSDPSS VIPIGDFDFA ERERSFTFST YYTQPEVVTA LTGVRSECMK

**401** VLEGSLFNLP KARQMQLSEF QKLQRDHMAA VEKYLKGEWT ENICNVIRNS

**451** FVSAGKGWLN VHESKQEIYE ISKLKKFFTT VKFMMEDTLF DLVYTSLQDF

**501** TVFFEEVSEF TVNVIDMNNV ENKWPGSDAD DCVEKQPLFT IRLAEQDGSF

**551** TYSISFKDFE EAIIDLFFSA IQCTDAIPQV EKFVMSQYFW RREGEGPFLD

**601** SVKQQEERVC LLRDRVRHAL QNSMKPLRDY LQTYDDLLPL VRLDKKVFIT

**651** EYAAQEHTME EMKEEIRAHL KAKKVVAQKL PAFITVGNYV VDCQSFGQIM

**701** ASKEHELAKL VMNLICKIAK TKTSYIREEF TKIVRVVEKQ PQTPEKLYEL

**751** KAIIVNTPER ISELSAEIEE MRQYYNVLDG FQYELSDEES RQKWEAISWP

**801** RQLTLRIQET NKQLEKVEEE LHARLQKEAE EFSKKVDALQ RVVATFSKYT

**851** DATEAEKVAA EVKVNSIEIR KCIEQARSIN SDQRLFGDKL TDYRSVFELE

**901** KEFKPYSDLW LTTYQWQDCY RRWHADPFDS LDHEEIDTVV TNAFKTMTQL

**951** SKTFKDKNAT LKIVSEIRGK VEAFKKWVPI VTSLRQPGMK ERHWKGLSEK

**1001** LNLPLVPGET ILLMEDLEPL LGFKDVIVPH CEVAAKEAQI EKALKDMRAK

**1051** WESRVFIIEP YKESNTYIIK DSSEIVELLD EHLNLTQQLQ FSPFKAYYAE

**1101** AITDWERSLN LISDIIEQWL ECQRAWRYLE PIFNAKDIAL QLPRLTKLFD

**1151** RVDKTWRRVM GTVHHQPNVL DFCIGTSKLL ESLRESNRIL EEVQRGLNDY

**1201** LAEKRQSFPR FYFLSDEELL EILSQSKEVR RIDAHISKLF EFIQRLSWTE

**1251** NNEINGFFSG EGEHVPSVNV VYPEGNVEMW LGSVETMMKE AVAEQLRQSF

**1301** YAYSNTPRAK WVLEWAAQCV IAVSQIFWTN GCEEGLVAEK SVENYFRVLE

**1351** HQLFELVDVV QSPLNARERI NMGALITVEV HAKDTVEAMT RHKVDSIQSF

**1401** EWIKQLRFYF DTDDRMCHIK QVDAHFVYGG EYLGNTGRLV VTPLTDRIYL

**1451** TLTGALALCL GGAPAGPAGT GKTETTKDLA KALAKQCVVF NCQEGMTCLS

**1501** MAKFFKGLAW AGAWACFDEF NRIDVEVLSV VAQQVTDLQQ ACVTKQYRIV

**1551** FEGSEVVVDP THAVFITMNP GYAGRTELPD NLKVLFRPVA CMVPDYAMIG

**1601** EIRLFSYGYK KARSLAQKMV MTFK**LSSEQL SSQDHYDFGM R**AVNTVISAA

**1651** GLNKRENPNE DEDLLLLRAL RDSNAPKFLR DDIILFEGII SDLFPGTKLS

**1701** PTEYGVVVDS LRQVVTSSQL QPVPGFIEKC LQLYDVTTLR HGLMLVGPAG

**1751** SGKTMAYTSL QKALSGCSVM QSKGQDVGAR DYMKVFTHIC NPKAVTMDQL

**1801** YGAYDENGEW KDGVLCVLFR RAAKYGDEGN QIGKHWVMFD GPVDALWIES

**1851** MNTVLDENKK LCLVSGEIIQ MSRDMTMMFE VEDLAVASPA TVSRCGMIYM

**1901** EPTACVPTQA LTKSWKERLP KYVAPQADYL EQLVELYVDE LIEYVRANLR

**1951** EYVPSTNVIL VHSFFRMMDG YIESFGGLPG QRGPPTLSPE RLEIMAKCIT

**2001** PLFFMAITWS IGATCDEVGR EKFADMLREM ATRNNHADSL PESGSVYDYC

**2051** FVYYPSPDDD EEARWTHWDE LRATCDIART TKFEDVLVPT IDNTRQKYVL

**2101** THLLERKVNV VAVGPTGTGK **SVAAGGLVMN GISDR**LLGLA FSFTPQTKAG

**2151** VLQDSLMSKF DKRRSHVYGA PVGKHFLVFI DDANLPQKER YGAQPPLELL

**2201** RQLLGHGGLY SFVGGIKWNL VIDTSFVMAM GPPGGSRTQV SNRLMRYFNY

**2251** VSFPEMSEAS KRTILNTILK GGLHQRGVKE EVVDFITNLV DGTLNVFKRC

**2301** RKAFVPTPSH VHYSFNMRDV MRVFPMIYIN DTNSLPNRDV LLKQWVHEMQ

**2351** RVFCDRLICN EDREEFLSFI DDEIIQIGYE GGYKSLLPDG RLIFGDFMST

**2401** GERSYQQITD MDALAAFFNE QLLAYNNANE NPMGLVLFLD AIEHVCRITR

**2451** VLSMPNGHCL LLGIGGSGRK SLTRLACFLI PEMDVFTIEF TKNFGVKEWR

**2501** EALARLLLDC GKDGKKRTFL FSDTQIINQT LMEDVAALLT AGDVPNLFED

**2551** QDIEIINERF KGVCMSENLP TTKVSMYARF IKEVRSNLHI VLAFSPIGEV

**2601** FRTRLRMFPA LITCCTIDWF AEWPGEALLS VARAQLQSAK GDLGDDEGDR

**2651** LSRCFKSLHL SAAETTERFF VETHRRSYIT PTSYLSLLNT YISLVESKRK

**2701** FGREQASRLE NGLEKLYDTE VRVVELEGQL K**AQQPVLEMK** KLEIRGIMEK

**2751** LRVDRKDAAE KEASARTEEV AATTKAEECA RMRRECASRL AEAEPALQEA

**2801** VKVLSKIKAA EISELNKYQN PPKGVQYVME AVALLLTFGN CPKEFYSGPP

**2851** GGKKTPDWWL CAKSYMKNAN QLLDTLVQPP GKGGFDREAM DMPLIEKVRT

**2901** YYENDEFQPE KVKSVSVPCM AMCQWVRAMY KWFFVNREIQ PLRERLADAE

**2951** RELKRVNRAL AETRRKLDAV VEAVAKLEKE FEDAMATQTA LENEVEQTSE

**3001** KLQRAARLIA GLGGEKVRWK ELVEQYK**VKD TCVSGDMVIA AASIAYFGPL**

**3051 TGPYR**KHLLQ TWSASLAELG IKTSENSDLL STTGDAVQIH DWQLCGLPKD

**3101** PLSTENAIIL SNARTWPLLI DPQGQANSWI RNLHKDDNLQ VCKASDDKFM

**3151** KTVEGAIRLG LPCLLENVGE SLDPALEPVL HR**NVFLIGCT PHIR**VGDSAI

**3201** PYNEKFRLYM TTKLPNPSYT PETIVIVSLL NFFITRSGLE DQILARTVEK

**3251** ERNDLEQEKQ RLTRDCAEKN RELKELQENI LRMLEEAEGD ILDQEELIDA

**3301** LEKSKLKSTE ISEDLVR**ARA TEVTIDETR**N KYRPHAYRGA LLFFCVSELS

**3351** TVDPMYQFSL QWYINLVLLA IENTEAAVDI EERVEKLIEF FTYSFYTNVC

**3401** RSLFERHKLT FSFFLCTSIL QQQDELDGNE YHYLLTGPTG SGGEEPNPAP

**3451** DWLTENSWNE IQFVSSNLPN FAGFAEHVTQ CINYYKELFD SLNAHTYPLA

**3501** AEWQGRETPL QRLVVVRCFR RDKVASAIQE FVKHYMGERF IIVPQFDLMD

**3551** AYKDSTCLTP LIFIISPGSD PMNDLLRFAE HMRMSKKLDK VSLGQGQGRK

**3601** AEELLSNGRE RGQWVLLQNC HLATSWMPTL EAIVESFTLE TVRKEFRLWL

**3651** TSMPSDSFPV AVLQISVKMT NEPPMGLRAN VTRSYYGLTD DDLEHPTKPN

**3701** QFKKMVFAFC LFHAVIQERR KFGSLGFNIA YEFNDSDRNV CLLQLRKFIS

**3751** LYEDVPFDVL TFLTGEINYG GRVTDDWDRR CMMALIKDFI TPGVLEEGYS

**3801** FSPSGTYHTV EACSRAFYLD YLGTWPLNPE PEVFGLSDNA DITCAQSESA

**3851** SILATILSLV SRESSGSSHQ SREEMLIKTA QHIMEKLPPT FNVQEFHAKY

**3901** PTKYEESMNT VLVQEAVRYN RLLRFVQK**SL SEFSK**AVRGE VDMSAELEAV

**3951** GSSFFINAVP ASWAALAYPS LKPLSSWVED LLRRVQFVQS WYDKGMPNAL

**4001** WMGGFFFPQA FLTGTLQNYA RRKDVAIDSV SFNFSFLQDE TPTTVAAPEQ

**4051** GAIVYGLYLE GARWDGAGRT LAESRPK**ELY VDVPLLHLDP VVDR**VADPND

**4101** YICPVYKTLT RAGTLSTTGH STNFVLSITI PTVAPPEHWI KRGVACVISL

**4151** NF

**Start - End Observed Mr(expt) Mr(calc) Delta Miss Sequence**

**86 - 94 1293.66 1292.66 1292.70 -0.04 0 K.VQVPFEFQR.G**  ([Ions score 15](../../../../D:%5CProteomic%20data%5C2010-1-8%5CZQ%5C1167.htm))

**169 - 184 2095.99 2094.98 2094.98 -0.00 0 R.YLPAEGIYEFMNSDFR.M**  ([Ions score 65](../../../../D:%5CProteomic%20data%5C2010-1-8%5CZQ%5C1172.htm))

**1625 - 1641 2143.99 2142.98 2142.98 0.00 0 K.LSSEQLSSQDHYDFGMR.A**  ([Ions score 8](../../../../D:%5CProteomic%20data%5C2010-1-8%5CZQ%5C1174.htm))

**2121 - 2135 1590.75 1589.74 1589.83 -0.09 0 K.SVAAGGLVMNGISDR.L**  ([Ions score 10](../../../../D:%5CProteomic%20data%5C2010-1-8%5CZQ%5C1171.htm))

**2732 - 2740 1347.72 1346.72 1346.66 0.06 0 K.AQQPVLEMK.K**  Oxidation (M) ([Ions score 0](../../../../D:%5CProteomic%20data%5C2010-1-8%5CZQ%5C1168.htm))

**3028 - 3055 3236.52 3235.51 3235.54 -0.03 1 K.VKDTCVSGDMVIAAASIAYFGPLTGPYR.K**  ([Ions score 0](../../../../D:%5CProteomic%20data%5C2010-1-8%5CZQ%5C1175.htm))

**3183 - 3194 1559.89 1558.88 1558.82 0.06 0 R.NVFLIGCTPHIR.V**  ([Ions score 1](../../../../D:%5CProteomic%20data%5C2010-1-8%5CZQ%5C1170.htm))

**3318 - 3329 1505.80 1504.79 1504.80 -0.00 1 R.ARATEVTIDETR.N**  ([Ions score 8](../../../../D:%5CProteomic%20data%5C2010-1-8%5CZQ%5C1169.htm))

**3929 - 3935 1085.56 1084.55 1084.51 0.04 0 K.SLSEFSK.A**  ([Ions score 6](../../../../D:%5CProteomic%20data%5C2010-1-8%5CZQ%5C1166.htm))

**4078 - 4094 2136.18 2135.17 2135.18 -0.00 0 K.ELYVDVPLLHLDPVVDR.V**  ([Ions score 1](../../../../D:%5CProteomic%20data%5C2010-1-8%5CZQ%5C1173.htm))

MS/MS Fragmentation of **YLPAEGIYEFMNSDFR**
Found in **Tb11.01.0390**, dynein heavy chain, putative; Trypanosoma bruceichr 11Manual


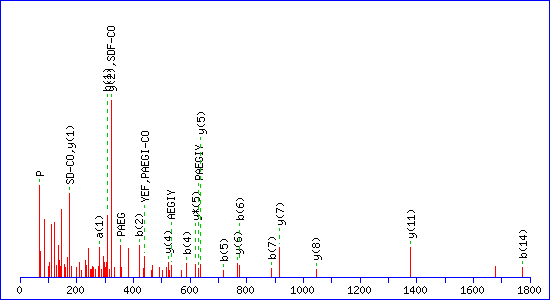


**MONOISOTOPIC mass of neutral peptide Mr(calc):** 2094.98

**Fixed modifications:** MMTS (C),(N-TERM)_iTRAQ,Lysine(K)_iTRAQ

**Ions Score:** 65 **Expect:** 3.4e-005

**Matches (Bold Red):** 26/243 fragment ions using 27 most intense peaks

| **#** | **Immon.** | **a** | **a*** | **a^0^** | **b** | **b*** | **b^0^** | **Seq.** | **v** | **w** | **w'** | **y** | **y*** | **y^0^** | **#** |
| --- | --- | --- | --- | --- | --- | --- | --- | --- | --- | --- | --- | --- | --- | --- | --- |
| **1** | 136.08 | **280.18** |  |  | **308.17** |  |  | **Y** |  |  |  |  |  |  | **16** |
| **2** | 86.10 | 393.26 |  |  | **421.26** |  |  | **L** | 1730.75 | 1729.75 |  | 1788.83 | 1771.80 | 1770.82 | **15** |
| **3** | **70.07** | 490.31 |  |  | 518.31 |  |  | **P** | 1633.70 | 1632.70 |  | 1675.74 | 1658.72 | 1657.73 | **14** |
| **4** | 44.05 | 561.35 |  |  | **589.35** |  |  | **A** | 1562.66 |  |  | 1578.69 | 1561.66 | 1560.68 | **13** |
| **5** | 102.05 | 690.39 |  | 672.38 | **718.39** |  | 700.38 | **E** | 1433.62 | 1432.62 |  | 1507.65 | 1490.63 | 1489.64 | **12** |
| **6** | 30.03 | 747.42 |  | 729.41 | **775.41** |  | 757.40 | **G** |  |  |  | **1378.61** | 1361.58 | 1360.60 | **11** |
| **7** | 86.10 | 860.50 |  | 842.49 | **888.50** |  | 870.48 | **I** | 1263.51 | 1276.53 | 1290.55 | 1321.59 | 1304.56 | 1303.58 | **10** |
| **8** | 136.08 | 1023.56 |  | 1005.55 | 1051.56 |  | 1033.55 | **Y** | 1100.45 |  |  | 1208.50 | 1191.48 | 1190.49 | **9** |
| **9** | 102.05 | 1152.61 |  | 1134.60 | 1180.60 |  | 1162.59 | **E** | 971.40 | 970.41 |  | **1045.44** | 1028.41 | 1027.43 | **8** |
| **10** | 120.08 | 1299.67 |  | 1281.66 | 1327.67 |  | 1309.66 | **F** | 824.34 |  |  | **916.40** | 899.37 | 898.39 | **7** |
| **11** | 104.05 | 1430.71 |  | 1412.70 | 1458.71 |  | 1440.70 | **M** | 693.30 | 692.30 |  | **769.33** | 752.30 | 751.32 | **6** |
| **12** | 87.06 | 1544.76 | 1527.73 | 1526.75 | 1572.75 | 1555.73 | 1554.74 | **N** | 579.25 | 578.26 |  | **638.29** | **621.26** | 620.28 | **5** |
| **13** | 60.04 | 1631.79 | 1614.76 | 1613.78 | 1659.78 | 1642.76 | 1641.77 | **S** | 492.22 | 491.22 |  | **524.25** | 507.22 | 506.24 | **4** |
| **14** | 88.04 | 1746.82 | 1729.79 | 1728.81 | **1774.81** | 1757.79 | 1756.80 | **D** | 377.19 | 376.20 |  | 437.21 | 420.19 | 419.20 | **3** |
| **15** | 120.08 | 1893.89 | 1876.86 | 1875.87 | 1921.88 | 1904.85 | 1903.87 | **F** | 230.12 |  |  | **322.19** | 305.16 |  | **2** |
| **16** | 129.11 |  |  |  |  |  |  | **R** | 74.02 | 73.03 |  | **175.12** | 158.09 |  | **1** |

| **Seq** | **ya** | **yb** | **Seq** | **ya** | **yb** | **Seq** | **ya** | **yb** |
| --- | --- | --- | --- | --- | --- | --- | --- | --- |
| **LP** | 183.15 | 211.14 | **LPA** | 254.19 | 282.18 | **LPAE** | 383.23 | 411.22 |
| **LPAEG** | **440.25** | 468.25 | **LPAEGI** | 553.33 | 581.33 | **PA** | 141.10 | 169.10 |
| **PAE** | 270.14 | 298.14 | **PAEG** | 327.17 | **355.16** | **PAEGI** | **440.25** | 468.25 |
| **PAEGIY** | 603.31 | **631.31** | **AE** | 173.09 | 201.09 | **AEG** | 230.11 | 258.11 |
| **AEGI** | 343.20 | 371.19 | **AEGIY** | 506.26 | **534.26** | **AEGIYE** | 635.30 | 663.30 |
| **EG** | 159.08 | 187.07 | **EGI** | 272.16 | 300.16 | **EGIY** | 435.22 | 463.22 |
| **EGIYE** | 564.27 | 592.26 | **GI** | 143.12 | 171.11 | **GIY** | 306.18 | 334.18 |
| **GIYE** | 435.22 | 463.22 | **GIYEF** | 582.29 | 610.29 | **IY** | 249.16 | 277.15 |
| **IYE** | 378.20 | 406.20 | **IYEF** | 525.27 | 553.27 | **IYEFM** | 656.31 | 684.31 |
| **YE** | 265.12 | 293.11 | **YEF** | 412.19 | **440.18** | **YEFM** | 543.23 | 571.22 |
| **YEFMN** | 657.27 | 685.27 | **EF** | 249.12 | 277.12 | **EFM** | 380.16 | 408.16 |
| **EFMN** | 494.21 | 522.20 | **EFMNS** | 581.24 | 609.23 | **EFMNSD** | 696.27 | 724.26 |
| **FM** | 251.12 | 279.12 | **FMN** | 365.16 | 393.16 | **FMNS** | 452.20 | 480.19 |
| **FMNSD** | 567.22 | 595.22 | **MN** | 218.10 | 246.09 | **MNS** | 305.13 | 333.12 |
| **MNSD** | 420.15 | 448.15 | **MNSDF** | 567.22 | 595.22 | **NS** | 174.09 | 202.08 |
| **NSD** | 289.11 | 317.11 | **NSDF** | 436.18 | 464.18 | **SD** | **175.07** | 203.07 |
| **SDF** | **322.14** | 350.13 | **DF** | 235.11 | 263.10 |  |  |  |

32. Tb09.160.5560

Match to: **Tb09.160.5560** Score: **61**

**adenylosuccinate lyase, putative; Trypanosoma bruceichr 9Manual**

Nominal mass (M_r_): **57101**; Calculated pI value: **6.77**

NCBI BLAST search of [Tb09.160.5560](http://www.ncbi.nlm.nih.gov/blast/Blast.cgi?ALIGNMENTS=50&ALIGNMENT_VIEW=Pairwise&AUTO_FORMAT=Semiauto&CDD_SEARCH=on&CLIENT=web&COMPOSITION_BASED_STATISTICS=on&DATABASE=nr&DESCRIPTIONS=100&ENTREZ_QUERY=(none)&EXPECT=10&FILTER=L&FORMAT_BLOCK_ON_RESPAGE=None&FORMAT_OBJECT=Alignment&FORMAT_TYPE=HTML&GAPCOSTS=11+1&I_THRESH=0.001&LAYOUT=TwoWindows&MATRIX_NAME=BLOSUM62&NCBI_GI=on&PAGE=Proteins&PROGRAM=blastp&QUERY=MEKGSPSDLNGVDYSVDNPLFALSPLDGRYKRQTKALRAFFSEYGFFRYRVLVEVEYFTALCKDVPTIVPLRSVTDEQLQKLRKITLDCFSVSSAEEIKRLERVTNHDIKAVEYFIKERMDTCGLSHVTEFVHFGLTSQDINNTAIPMMIRDAIVTLYLPALDGIIGSLTSKLVDWDVPMLARTHGQPASPTNLAKEFVVWIERLREQRRQLCEVPTTGKFGGATGNFNAHLVAYPSVNWRAFADMFLAKYLGLKRQQATTQIENYDHLAALCDACARLHVILIDMCRDVWQYISMGFFKQKVKEGEVGSSTMPHKVNPIDFENAEGNLALSNALLNFFASKLPISRLQRDLTDSTVLRNLGVPIGHACVAFASISQGLEKLMISRETISRELSSNWAVVAEGIQTVLRRECYPKPYETLKKLTQGNTDVTEEQVRNFINGLTDISDDVRAELLAITPFTYVGYVPRFSAK&SERVICE=plain&SET_DEFAULTS.x=9&SET_DEFAULTS.y=5&SHOW_OVERVIEW=on&WORD_SIZE=3&END_OF_HTTPGET=Yes) against nr

Unformatted [sequence string](../../../../D:%5CProteomic%20data%5C2010-1-8%5Ccgi%5Cgetseq.pl%3FTBA927_IPI+Tb09%2E160%2E5560+seq) for pasting into other applications

Fixed modifications: MMTS (C),(N-TERM)_iTRAQ,Lysine(K)_iTRAQ

Variable modifications: Oxidation (M)

Cleavage by Trypsin: cuts C-term side of KR unless next residue is P

Sequence Coverage: **3%**

Matched peptides shown in **Bold Red**

**1** MEKGSPSDLN GVDYSVDNPL FALSPLDGRY KRQTKALRAF FSEYGFFRYR

**51** VLVEVEYFTA LCKDVPTIVP LRSVTDEQLQ KLRKITLDCF SVSSAEEIKR

**101** LERVTNHDIK AVEYFIKERM DTCGLSHVTE FVHFGLTSQD INNTAIPMMI

**151** RDAIVTLYLP ALDGIIGSLT SKLVDWDVPM LARTHGQPAS PTNLAKEFVV

**201** WIERLREQRR QLCEVPTTGK FGGATGNFNA HLVAYPSVNW RAFADMFLAK

**251** YLGLKRQQAT TQIENYDHLA ALCDACARLH VILIDMCRDV WQYISMGFFK

**301** QKVKEGEVGS STMPHKVNPI DFENAEGNLA LSNALLNFFA SKLPISRLQR

**351** DLTDSTVLRN LGVPIGHACV AFASISQGLE KLMISRETIS R**ELSSNWAVV**

**401 AEGIQTVLR**R ECYPKPYETL KKLTQGNTDV TEEQVRNFIN GLTDISDDVR

**451** AELLAITPFT YVGYVPRFSA K

MS/MS Fragmentation of **ELSSNWAVVAEGIQTVLR**
Found in **Tb09.160.5560**, adenylosuccinate lyase, putative; Trypanosoma bruceichr 9Manual


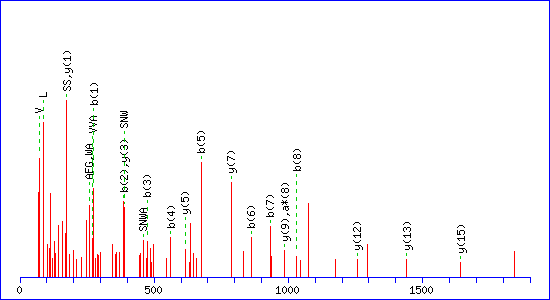


**MONOISOTOPIC mass of neutral peptide Mr(calc):** 2115.14

**Fixed modifications:** MMTS (C),(N-TERM)_iTRAQ,Lysine(K)_iTRAQ

**Ions Score:** 61 **Expect:** 6.5e-005

**Matches (Bold Red):** 30/330 fragment ions using 27 most intense peaks

| **#** | **Immon.** | **a** | **a*** | **a^0^** | **b** | **b*** | **b^0^** | **Seq.** | **v** | **w** | **w'** | **y** | **y*** | **y^0^** | **#** |
| --- | --- | --- | --- | --- | --- | --- | --- | --- | --- | --- | --- | --- | --- | --- | --- |
| **1** | 102.05 | 246.16 |  | 228.15 | **274.15** |  | 256.14 | **E** |  |  |  |  |  |  | **18** |
| **2** | **86.10** | 359.24 |  | 341.23 | **387.24** |  | 369.23 | **L** | 1784.93 | 1783.93 |  | 1843.01 | 1825.98 | 1825.00 | **17** |
| **3** | 60.04 | 446.27 |  | 428.26 | **474.27** |  | 456.26 | **S** | 1697.90 | 1696.90 |  | 1729.92 | 1712.90 | 1711.91 | **16** |
| **4** | 60.04 | 533.31 |  | 515.29 | **561.30** |  | 543.29 | **S** | 1610.86 | 1609.87 |  | **1642.89** | 1625.86 | 1624.88 | **15** |
| **5** | 87.06 | 647.35 | 630.32 | 629.34 | **675.34** | 658.32 | 657.33 | **N** | 1496.82 | 1495.83 |  | 1555.86 | 1538.83 | 1537.85 | **14** |
| **6** | 159.09 | 833.43 | 816.40 | 815.42 | **861.42** | 844.40 | 843.41 | **W** | 1310.74 |  |  | **1441.82** | 1424.79 | 1423.81 | **13** |
| **7** | 44.05 | 904.46 | 887.44 | 886.45 | **932.46** | 915.43 | 914.45 | **A** | 1239.71 |  |  | **1255.74** | 1238.71 | 1237.73 | **12** |
| **8** | **72.08** | 1003.53 | **986.51** | 985.52 | **1031.53** | 1014.50 | 1013.52 | **V** | 1140.64 | 1153.66 |  | 1184.70 | 1167.67 | 1166.69 | **11** |
| **9** | **72.08** | 1102.60 | 1085.58 | 1084.59 | 1130.60 | 1113.57 | 1112.59 | **V** | 1041.57 | 1054.59 |  | 1085.63 | 1068.60 | 1067.62 | **10** |
| **10** | 44.05 | 1173.64 | 1156.61 | 1155.63 | 1201.63 | 1184.61 | 1183.62 | **A** | 970.53 |  |  | **986.56** | 969.54 | 968.55 | **9** |
| **11** | 102.05 | 1302.68 | 1285.65 | 1284.67 | 1330.68 | 1313.65 | 1312.67 | **E** | 841.49 | 840.49 |  | 915.53 | 898.50 | 897.52 | **8** |
| **12** | 30.03 | 1359.70 | 1342.68 | 1341.69 | 1387.70 | 1370.67 | 1369.69 | **G** |  |  |  | **786.48** | 769.46 | 768.47 | **7** |
| **13** | **86.10** | 1472.79 | 1455.76 | 1454.78 | 1500.78 | 1483.76 | 1482.77 | **I** | 671.38 | 684.40 | 698.42 | 729.46 | 712.44 | 711.45 | **6** |
| **14** | 101.07 | 1600.85 | 1583.82 | 1582.83 | 1628.84 | 1611.81 | 1610.83 | **Q** | 543.32 | 542.33 |  | **616.38** | 599.35 | 598.37 | **5** |
| **15** | 74.06 | 1701.89 | 1684.87 | 1683.88 | 1729.89 | 1712.86 | 1711.88 | **T** | 442.28 | 455.30 | 457.28 | 488.32 | 471.29 | 470.31 | **4** |
| **16** | **72.08** | 1800.96 | 1783.94 | 1782.95 | 1828.96 | 1811.93 | 1810.95 | **V** | 343.21 | 356.23 |  | **387.27** | 370.24 |  | **3** |
| **17** | **86.10** | 1914.05 | 1897.02 | 1896.04 | 1942.04 | 1925.01 | 1924.03 | **L** | 230.12 | 229.13 |  | 288.20 | 271.18 |  | **2** |
| **18** | 129.11 |  |  |  |  |  |  | **R** | 74.02 | 73.03 |  | **175.12** | 158.09 |  | **1** |

| **Seq** | **ya** | **yb** | **Seq** | **ya** | **yb** | **Seq** | **ya** | **yb** |
| --- | --- | --- | --- | --- | --- | --- | --- | --- |
| **LS** | 173.13 | 201.12 | **LSS** | 260.16 | 288.16 | **LSSN** | 374.20 | 402.20 |
| **LSSNW** | 560.28 | 588.28 | **LSSNWA** | 631.32 | 659.31 | **SS** | 147.08 | **175.07** |
| **SSN** | 261.12 | 289.11 | **SSNW** | 447.20 | 475.19 | **SSNWA** | 518.24 | 546.23 |
| **SSNWAV** | 617.30 | 645.30 | **SN** | 174.09 | 202.08 | **SNW** | 360.17 | **388.16** |
| **SNWA** | 431.20 | **459.20** | **SNWAV** | 530.27 | 558.27 | **SNWAVV** | 629.34 | 657.34 |
| **NW** | 273.13 | 301.13 | **NWA** | 344.17 | 372.17 | **NWAV** | 443.24 | 471.24 |
| **NWAVV** | 542.31 | 570.30 | **NWAVVA** | 613.35 | 641.34 | **WA** | 230.13 | **258.12** |
| **WAV** | 329.20 | 357.19 | **WAVV** | 428.27 | 456.26 | **WAVVA** | 499.30 | 527.30 |
| **WAVVAE** | 628.35 | 656.34 | **WAVVAEG** | 685.37 | 713.36 | **AV** | 143.12 | 171.11 |
| **AVV** | 242.19 | **270.18** | **AVVA** | 313.22 | 341.22 | **AVVAE** | 442.27 | 470.26 |
| **AVVAEG** | 499.29 | 527.28 | **AVVAEGI** | 612.37 | 640.37 | **VV** | 171.15 | 199.14 |
| **VVA** | 242.19 | **270.18** | **VVAE** | 371.23 | 399.22 | **VVAEG** | 428.25 | 456.25 |
| **VVAEGI** | 541.33 | 569.33 | **VVAEGIQ** | 669.39 | 697.39 | **VA** | 143.12 | 171.11 |
| **VAE** | 272.16 | 300.16 | **VAEG** | 329.18 | 357.18 | **VAEGI** | 442.27 | 470.26 |
| **VAEGIQ** | 570.32 | 598.32 | **VAEGIQT** | 671.37 | 699.37 | **AE** | 173.09 | 201.09 |
| **AEG** | 230.11 | **258.11** | **AEGI** | 343.20 | 371.19 | **AEGIQ** | 471.26 | 499.25 |
| **AEGIQT** | 572.30 | 600.30 | **AEGIQTV** | 671.37 | 699.37 | **EG** | 159.08 | 187.07 |
| **EGI** | 272.16 | 300.16 | **EGIQ** | 400.22 | 428.21 | **EGIQT** | 501.27 | 529.26 |
| **EGIQTV** | 600.34 | 628.33 | **GI** | 143.12 | 171.11 | **GIQ** | 271.18 | 299.17 |
| **GIQT** | 372.22 | 400.22 | **GIQTV** | 471.29 | 499.29 | **GIQTVL** | 584.38 | 612.37 |
| **IQ** | 214.16 | 242.15 | **IQT** | 315.20 | 343.20 | **IQTV** | 414.27 | 442.27 |
| **IQTVL** | 527.36 | 555.35 | **QT** | 202.12 | 230.11 | **QTV** | 301.19 | 329.18 |
| **QTVL** | 414.27 | 442.27 | **TV** | 173.13 | 201.12 | **TVL** | 286.21 | 314.21 |
| **VL** | 185.16 | 213.16 |  |  |  |  |  |  |

33. Tb927.3.4500

Match to: **Tb927.3.4500** Score: **61**

**fumarate hydratase, putative; Trypanosoma bruceichr 3Manual**

Nominal mass (M_r_): **67671**; Calculated pI value: **6.17**

NCBI BLAST search of [Tb927.3.4500](http://www.ncbi.nlm.nih.gov/blast/Blast.cgi?ALIGNMENTS=50&ALIGNMENT_VIEW=Pairwise&AUTO_FORMAT=Semiauto&CDD_SEARCH=on&CLIENT=web&COMPOSITION_BASED_STATISTICS=on&DATABASE=nr&DESCRIPTIONS=100&ENTREZ_QUERY=(none)&EXPECT=10&FILTER=L&FORMAT_BLOCK_ON_RESPAGE=None&FORMAT_OBJECT=Alignment&FORMAT_TYPE=HTML&GAPCOSTS=11+1&I_THRESH=0.001&LAYOUT=TwoWindows&MATRIX_NAME=BLOSUM62&NCBI_GI=on&PAGE=Proteins&PROGRAM=blastp&QUERY=MSLCENCELGENCHGVDDIEETLPITTEFHYEPIFQPSDPHHDKTEYYNIPGDYVKEIEVMGRKVLSVDPTALTVLAQHAFTDVHHYFREDHLSGWRRVLDDPEATDNDRFVATTLLQNACVAAGRILPACQDTGTAIVLGKRGELCWTGGEDEKYLSHGIWKCYVSRNLRYSQTAALDMFKEANTGDNLPAQMDLMAVPGNEYHFLFVAKGGGSANKAFLYQQTKALLNPKSLRAFVEEKLKTLGTSACPPYHIALVIGGTSAEMTMKTVKLASCRYYDSLPTTGNKSGRAFRDIEWEEIILEMTRNLGIGAQFGGKYFAHQIRVIRLPRHGASCPVGLGVSCSADRQILGKITSEGVFLEKLVRDPAKYLPDVPLTSLGNGTVEVDLHQPMSKIRELLTQYPVTTRLSLTGPLIVARDIAHAKIMERLNADEPLPQYMKDHPIYYAGPAKTPKGMASGSFGPTTAGRMDSYVAPFMAAGGSFVTLAKGNRSKVVTDACKKYGGFYLGSIGGPAAILARDNIKKVEVVEYPELGMEAVWRIEVVNFPAFIIVDDKGNDFYAKLV&SERVICE=plain&SET_DEFAULTS.x=9&SET_DEFAULTS.y=5&SHOW_OVERVIEW=on&WORD_SIZE=3&END_OF_HTTPGET=Yes) against nr

Unformatted [sequence string](../../../../D:%5CProteomic%20data%5C2010-1-8%5Ccgi%5Cgetseq.pl%3FTBA927_IPI+Tb927%2E3%2E4500+seq) for pasting into other applications

Fixed modifications: MMTS (C),(N-TERM)_iTRAQ,Lysine(K)_iTRAQ

Variable modifications: Oxidation (M)

Cleavage by Trypsin: cuts C-term side of KR unless next residue is P

Sequence Coverage: **4%**

Matched peptides shown in **Bold Red**

**1** MSLCENCELG ENCHGVDDIE ETLPITTEFH YEPIFQPSDP HHDKTEYYNI

**51** PGDYVK**EIEV MGR**KVLSVDP TALTVLAQHA FTDVHHYFRE DHLSGWRRVL

**101** DDPEATDNDR FVATTLLQNA CVAAGRILPA CQDTGTAIVL GKRGELCWTG

**151** GEDEKYLSHG IWKCYVSRNL RYSQTAALDM FKEANTGDNL PAQMDLMAVP

**201** GNEYHFLFVA KGGGSANKAF LYQQTKALLN PKSLRAFVEE KLKTLGTSAC

**251** PPYHIALVIG GTSAEMTMKT VKLASCRYYD SLPTTGNKSG RAFRDIEWEE

**301** IILEMTRNLG IGAQFGGKYF AHQIRVIRLP RHGASCPVGL GVSCSADRQI

**351** LGKITSEGVF LEKLVRDPAK YLPDVPLTSL GNGTVEVDLH QPMSKIRELL

**401** TQYPVTTRLS LTGPLIVARD IAHAKIMERL NADEPLPQYM KDHPIYYAGP

**451** AKTPKGMASG SFGPTTAGRM DSYVAPFMAA GGSFVTLAKG NRSKVVTDAC

**501** KK**YGGFYLGS IGGPAAILAR** DNIKKVEVVE YPELGMEAVW RIEVVNFPAF

**551** IIVDDKGNDF YAKLV

**Start - End Observed Mr(expt) Mr(calc) Delta Miss Sequence**

**57 - 63 977.52 976.51 976.51 -0.00 0 K.EIEVMGR.K**  ([Ions score 20](../../../../D:%5CProteomic%20data%5C2010-1-8%5CZQ%5C1188.htm))

**503 - 520 1927.05 1926.04 1926.05 -0.01 0 K.YGGFYLGSIGGPAAILAR.D**  ([Ions score 61](../../../../D:%5CProteomic%20data%5C2010-1-8%5CZQ%5C1189.htm))

MS/MS Fragmentation of **YGGFYLGSIGGPAAILAR**
Found in **Tb927.3.4500**, fumarate hydratase, putative; Trypanosoma bruceichr 3Manual


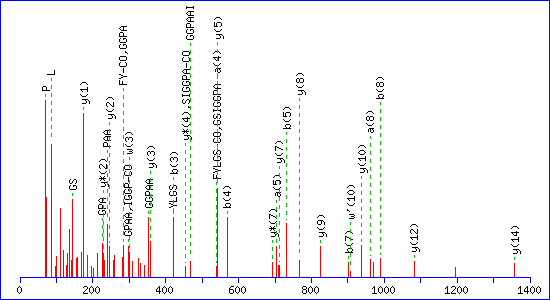


**MONOISOTOPIC mass of neutral peptide Mr(calc):** 1926.05

**Fixed modifications:** MMTS (C),(N-TERM)_iTRAQ,Lysine(K)_iTRAQ

**Ions Score:** 61 **Expect:** 7.2e-005

**Matches (Bold Red):** 42/292 fragment ions using 38 most intense peaks

| **#** | **Immon.** | **a** | **a^0^** | **b** | **b^0^** | **Seq.** | **v** | **w** | **w'** | **y** | **y*** | **y^0^** | **#** |
| --- | --- | --- | --- | --- | --- | --- | --- | --- | --- | --- | --- | --- | --- |
| **1** | 136.08 | 280.18 |  | 308.17 |  | **Y** |  |  |  |  |  |  | **18** |
| **2** | 30.03 | 337.20 |  | 365.19 |  | **G** |  |  |  | 1619.89 | 1602.86 | 1601.88 | **17** |
| **3** | 30.03 | 394.22 |  | **422.22** |  | **G** |  |  |  | 1562.87 | 1545.84 | 1544.86 | **16** |
| **4** | 120.08 | **541.29** |  | **569.28** |  | **F** | 1413.78 |  |  | 1505.85 | 1488.82 | 1487.84 | **15** |
| **5** | 136.08 | **704.35** |  | **732.35** |  | **Y** | 1250.72 |  |  | **1358.78** | 1341.75 | 1340.77 | **14** |
| **6** | **86.10** | 817.44 |  | 845.43 |  | **L** | 1137.64 | 1136.64 |  | 1195.72 | 1178.69 | 1177.71 | **13** |
| **7** | 30.03 | 874.46 |  | **902.45** |  | **G** |  |  |  | **1082.63** | 1065.61 | 1064.62 | **12** |
| **8** | 60.04 | **961.49** | 943.48 | **989.49** | 971.47 | **S** | 993.58 | 992.59 |  | 1025.61 | 1008.58 | 1007.60 | **11** |
| **9** | **86.10** | 1074.57 | 1056.56 | 1102.57 | 1084.56 | **I** | 880.50 | 893.52 | **907.54** | **938.58** | 921.55 |  | **10** |
| **10** | 30.03 | 1131.60 | 1113.59 | 1159.59 | 1141.58 | **G** |  |  |  | **825.49** | 808.47 |  | **9** |
| **11** | 30.03 | 1188.62 | 1170.61 | 1216.61 | 1198.60 | **G** |  |  |  | **768.47** | 751.45 |  | **8** |
| **12** | **70.07** | 1285.67 | 1267.66 | 1313.66 | 1295.65 | **P** | 669.40 | 668.41 |  | **711.45** | **694.42** |  | **7** |
| **13** | 44.05 | 1356.71 | 1338.70 | 1384.70 | 1366.69 | **A** | 598.37 |  |  | 614.40 | 597.37 |  | **6** |
| **14** | 44.05 | 1427.74 | 1409.73 | 1455.74 | 1437.73 | **A** | 527.33 |  |  | **543.36** | 526.33 |  | **5** |
| **15** | **86.10** | 1540.83 | 1522.82 | 1568.82 | 1550.81 | **I** | 414.25 | 427.27 | 441.28 | 472.32 | **455.30** |  | **4** |
| **16** | **86.10** | 1653.91 | 1635.90 | 1681.91 | 1663.90 | **L** | 301.16 | **300.17** |  | **359.24** | 342.21 |  | **3** |
| **17** | 44.05 | 1724.95 | 1706.94 | 1752.94 | 1734.93 | **A** | 230.12 |  |  | **246.16** | **229.13** |  | **2** |
| **18** | 129.11 |  |  |  |  | **R** | 74.02 | 73.03 |  | **175.12** | 158.09 |  | **1** |

| **Seq** | **ya** | **yb** | **Seq** | **ya** | **yb** | **Seq** | **ya** | **yb** |
| --- | --- | --- | --- | --- | --- | --- | --- | --- |
| **GG** | 87.06 | 115.05 | **GGF** | 234.12 | 262.12 | **GGFY** | 397.19 | 425.18 |
| **GGFYL** | 510.27 | 538.27 | **GGFYLG** | 567.29 | 595.29 | **GGFYLGS** | 654.32 | 682.32 |
| **GF** | 177.10 | 205.10 | **GFY** | 340.17 | 368.16 | **GFYL** | 453.25 | 481.24 |
| **GFYLG** | 510.27 | 538.27 | **GFYLGS** | 597.30 | 625.30 | **FY** | **283.14** | 311.14 |
| **FYL** | 396.23 | 424.22 | **FYLG** | 453.25 | 481.24 | **FYLGS** | **540.28** | 568.28 |
| **FYLGSI** | 653.37 | 681.36 | **YL** | 249.16 | 277.15 | **YLG** | 306.18 | 334.18 |
| **YLGS** | 393.21 | **421.21** | **YLGSI** | 506.30 | 534.29 | **YLGSIG** | 563.32 | 591.31 |
| **YLGSIGG** | 620.34 | 648.34 | **LG** | 143.12 | 171.11 | **LGS** | 230.15 | 258.14 |
| **LGSI** | 343.23 | 371.23 | **LGSIG** | 400.26 | 428.25 | **LGSIGG** | 457.28 | 485.27 |
| **LGSIGGP** | 554.33 | 582.32 | **LGSIGGPA** | 625.37 | 653.36 | **LGSIGGPAA** | 696.40 | 724.40 |
| **GS** | 117.07 | **145.06** | **GSI** | 230.15 | 258.14 | **GSIG** | 287.17 | 315.17 |
| **GSIGG** | 344.19 | 372.19 | **GSIGGP** | 441.25 | 469.24 | **GSIGGPA** | 512.28 | **540.28** |
| **GSIGGPAA** | 583.32 | 611.31 | **GSIGGPAAI** | 696.40 | 724.40 | **SI** | 173.13 | 201.12 |
| **SIG** | 230.15 | 258.14 | **SIGG** | 287.17 | 315.17 | **SIGGP** | 384.22 | 412.22 |
| **SIGGPA** | **455.26** | 483.26 | **SIGGPAA** | 526.30 | 554.29 | **SIGGPAAI** | 639.38 | 667.38 |
| **IG** | 143.12 | 171.11 | **IGG** | 200.14 | 228.13 | **IGGP** | **297.19** | 325.19 |
| **IGGPA** | 368.23 | 396.22 | **IGGPAA** | 439.27 | **467.26** | **IGGPAAI** | 552.35 | 580.35 |
| **IGGPAAIL** | 665.43 | 693.43 | **GG** | 87.06 | 115.05 | **GGP** | 184.11 | 212.10 |
| **GGPA** | 255.15 | **283.14** | **GGPAA** | 326.18 | **354.18** | **GGPAAI** | 439.27 | **467.26** |
| **GGPAAIL** | 552.35 | 580.35 | **GGPAAILA** | 623.39 | 651.38 | **GP** | 127.09 | 155.08 |
| **GPA** | 198.12 | **226.12** | **GPAA** | 269.16 | **297.16** | **GPAAI** | 382.24 | 410.24 |
| **GPAAIL** | 495.33 | 523.32 | **GPAAILA** | 566.37 | 594.36 | **PA** | 141.10 | 169.10 |
| **PAA** | 212.14 | **240.13** | **PAAI** | 325.22 | 353.22 | **PAAIL** | 438.31 | 466.30 |
| **PAAILA** | 509.34 | 537.34 | **AA** | 115.09 | 143.08 | **AAI** | 228.17 | 256.17 |
| **AAIL** | 341.25 | 369.25 | **AAILA** | 412.29 | 440.29 | **AI** | 157.13 | 185.13 |
| **AIL** | 270.22 | 298.21 | **AILA** | 341.25 | 369.25 | **IL** | 199.18 | 227.18 |
| **ILA** | 270.22 | 298.21 | **LA** | 157.13 | 185.13 |  |  |  |

34. Tb927.7.2680

Match to: **Tb927.7.2680** Score: **61**

**ZC3H22hypothetical protein, conserved; Trypanosoma bruceichr 7Manual**

Nominal mass (M_r_): **80839**; Calculated pI value: **6.53**

NCBI BLAST search of [Tb927.7.2680](http://www.ncbi.nlm.nih.gov/blast/Blast.cgi?ALIGNMENTS=50&ALIGNMENT_VIEW=Pairwise&AUTO_FORMAT=Semiauto&CDD_SEARCH=on&CLIENT=web&COMPOSITION_BASED_STATISTICS=on&DATABASE=nr&DESCRIPTIONS=100&ENTREZ_QUERY=(none)&EXPECT=10&FILTER=L&FORMAT_BLOCK_ON_RESPAGE=None&FORMAT_OBJECT=Alignment&FORMAT_TYPE=HTML&GAPCOSTS=11+1&I_THRESH=0.001&LAYOUT=TwoWindows&MATRIX_NAME=BLOSUM62&NCBI_GI=on&PAGE=Proteins&PROGRAM=blastp&QUERY=MFLPEQSNIPGSVTYVSRAAGIPSSSTTQQYQGGECMGVPSELDAIDGGCAWRTASKGVCASQLPRGGSVAVSVSAPFIHLTDWGDATVSTREISDNAAEVPAARSIGNREGVDGSLTSSVFPSGDVNSTIDLPISLVNSALPNQEKNNMEFQDDCLAHVFSRPESPFKDFSAESEDPFTIMQTRSVTCNPNIWEVEGKDVQSSLIHDAVKVGMSQITPSLFSPITEAMESAIDKVKVAPDGLNDCRTVGPQQPTEEQRIPVVDPQRSKLHVPLSSIIPTKALGGRVSFPSLCLLFQSGRCLRGASCYQMHIDPQVVQRLRQINESLPYCCAFHGECNANKWDAEANAHRTILIKGAAVPLSRVAYTNGLERFVLKNNASRSLNTCAVCRLHGKPGGCRYGADCWYVHICRDVLKELVATIKLDLEDPGSEDQCKVPTKTESTGNRVVSGDLLGALSQQQNRRKQQNYPTVAFHPSKSPTMSHLHKGRSIVSSERDGTHSGKLSSHGSASTNTFSARASPTVGQITSDGTTCRNVLVQQRPPVYEDGPLGETRHGLATTMRLHGVAFPVTDPSEEKSVPSPSQFSLNDRQSVTPISGREVSTPSVPCCAMYFPQKQSSVAPCPQPMQNGHTVWPSTVLVPSTTVMYPMISATGLPVASAQQQMGTDGQTYGPFCFVSGREDGFRNLCLPMYGSGKGNMPSQVPLHVPF&SERVICE=plain&SET_DEFAULTS.x=9&SET_DEFAULTS.y=5&SHOW_OVERVIEW=on&WORD_SIZE=3&END_OF_HTTPGET=Yes) against nr

Unformatted [sequence string](../../../../D:%5CProteomic%20data%5C2010-1-8%5Ccgi%5Cgetseq.pl%3FTBA927_IPI+Tb927%2E7%2E2680+seq) for pasting into other applications

Fixed modifications: MMTS (C),(N-TERM)_iTRAQ,Lysine(K)_iTRAQ

Variable modifications: Oxidation (M)

Cleavage by Trypsin: cuts C-term side of KR unless next residue is P

Sequence Coverage: **4%**

Matched peptides shown in **Bold Red**

**1** MFLPEQSNIP GSVTYVSRAA GIPSSSTTQQ YQGGECMGVP SELDAIDGGC

**51** AWRTASKGVC ASQLPRGGSV AVSVSAPFIH LTDWGDATVS TREISDNAAE

**101** VPAARSIGNR EGVDGSLTSS VFPSGDVNST IDLPISLVNS ALPNQEKNNM

**151** EFQDDCLAHV FSRPESPFKD FSAESEDPFT IMQTRSVTCN PNIWEVEGKD

**201** VQSSLIHDAV KVGMSQITPS LFSPITEAME SAIDKVKVAP DGLNDCR**TVG**

**251 PQQPTEEQR**I PVVDPQRSKL HVPLSSIIPT KALGGRVSFP SLCLLFQSGR

**301** CLRGASCYQM HIDPQVVQRL RQINESLPYC CAFHGECNAN KWDAEANAHR

**351** TILIK**GAAVP LSRVAYTNGL ER**FVLKNNAS RSLNTCAVCR LHGKPGGCRY

**401** GADCWYVHIC RDVLKELVAT IKLDLEDPGS EDQCKVPTKT ESTGNRVVSG

**451** DLLGALSQQQ NRRKQQNYPT VAFHPSKSPT MSHLHKGRSI VSSERDGTHS

**501** GKLSSHGSAS TNTFSARASP TVGQITSDGT TCRNVLVQQR PPVYEDGPLG

**551** ETRHGLATTM RLHGVAFPVT DPSEEKSVPS PSQFSLNDRQ SVTPISGREV

**601** STPSVPCCAM YFPQKQSSVA PCPQPMQNGH TVWPSTVLVP STTVMYPMIS

**651** ATGLPVASAQ QQMGTDGQTY GPFCFVSGRE DGFRNLCLPM YGSGKGNMPS

**701** QVPLHVPF

**Start - End Observed Mr(expt) Mr(calc) Delta Miss Sequence**

**248 - 259 1513.77 1512.76 1512.77 -0.00 0 R.TVGPQQPTEEQR.I**  ([Ions score 61](../../../../D:%5CProteomic%20data%5C2010-1-8%5CZQ%5C1198.htm))

**356 - 372 1917.99 1916.98 1917.06 -0.07 1 K.GAAVPLSRVAYTNGLER.F**  ([Ions score 2](../../../../D:%5CProteomic%20data%5C2010-1-8%5CZQ%5C1199.htm))

MS/MS Fragmentation of **TVGPQQPTEEQR**
Found in **Tb927.7.2680**, ZC3H22hypothetical protein, conserved; Trypanosoma bruceichr 7Manual


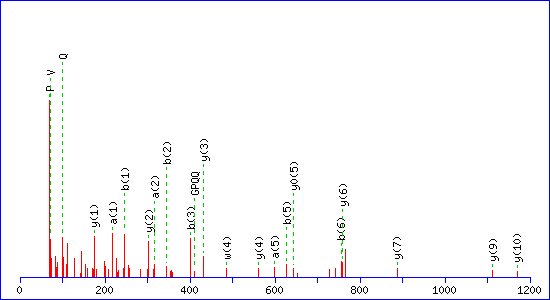


**MONOISOTOPIC mass of neutral peptide Mr(calc):** 1512.77

**Fixed modifications:** MMTS (C),(N-TERM)_iTRAQ,Lysine(K)_iTRAQ

**Ions Score:** 61 **Expect:** 0.00011

**Matches (Bold Red):** 25/191 fragment ions using 24 most intense peaks

| **#** | **Immon.** | **a** | **a*** | **a^0^** | **b** | **b*** | **b^0^** | **Seq.** | **v** | **w** | **w'** | **y** | **y*** | **y^0^** | **#** |
| --- | --- | --- | --- | --- | --- | --- | --- | --- | --- | --- | --- | --- | --- | --- | --- |
| **1** | 74.06 | **218.16** |  | 200.15 | **246.16** |  | 228.15 | **T** |  |  |  |  |  |  | **12** |
| **2** | **72.08** | **317.23** |  | 299.22 | **345.23** |  | 327.22 | **V** | 1224.56 | 1237.58 |  | 1268.62 | 1251.60 | 1250.61 | **11** |
| **3** | 30.03 | 374.25 |  | 356.24 | **402.25** |  | 384.24 | **G** |  |  |  | **1169.55** | 1152.53 | 1151.54 | **10** |
| **4** | **70.07** | 471.31 |  | 453.29 | 499.30 |  | 481.29 | **P** | 1070.49 | 1069.49 |  | **1112.53** | 1095.51 | 1094.52 | **9** |
| **5** | **101.07** | **599.36** | 582.34 | 581.35 | **627.36** | 610.33 | 609.35 | **Q** | 942.43 | 941.43 |  | 1015.48 | 998.45 | 997.47 | **8** |
| **6** | **101.07** | 727.42 | 710.40 | 709.41 | **755.42** | 738.39 | 737.41 | **Q** | 814.37 | 813.37 |  | **887.42** | 870.40 | 869.41 | **7** |
| **7** | **70.07** | 824.48 | 807.45 | 806.46 | 852.47 | 835.44 | 834.46 | **P** | 717.32 | 716.32 |  | **759.36** | 742.34 | 741.35 | **6** |
| **8** | 74.06 | 925.52 | 908.50 | 907.51 | 953.52 | 936.49 | 935.51 | **T** | 616.27 | 629.29 | 631.27 | 662.31 | 645.28 | **644.30** | **5** |
| **9** | 102.05 | 1054.57 | 1037.54 | 1036.55 | 1082.56 | 1065.53 | 1064.55 | **E** | 487.23 | **486.23** |  | **561.26** | 544.24 | 543.25 | **4** |
| **10** | 102.05 | 1183.61 | 1166.58 | 1165.60 | 1211.60 | 1194.58 | 1193.59 | **E** | 358.18 | 357.19 |  | **432.22** | 415.19 | 414.21 | **3** |
| **11** | **101.07** | 1311.67 | 1294.64 | 1293.66 | 1339.66 | 1322.63 | 1321.65 | **Q** | 230.12 | 229.13 |  | **303.18** | 286.15 |  | **2** |
| **12** | 129.11 |  |  |  |  |  |  | **R** | 74.02 | 73.03 |  | **175.12** | 158.09 |  | **1** |

| **Seq** | **ya** | **yb** | **Seq** | **ya** | **yb** | **Seq** | **ya** | **yb** |
| --- | --- | --- | --- | --- | --- | --- | --- | --- |
| **VG** | 129.10 | 157.10 | **VGP** | 226.15 | 254.15 | **VGPQ** | 354.21 | 382.21 |
| **VGPQQ** | 482.27 | 510.27 | **VGPQQP** | 579.32 | 607.32 | **VGPQQPT** | 680.37 | 708.37 |
| **GP** | 127.09 | 155.08 | **GPQ** | 255.15 | 283.14 | **GPQQ** | 383.20 | **411.20** |
| **GPQQP** | 480.26 | 508.25 | **GPQQPT** | 581.30 | 609.30 | **PQ** | 198.12 | 226.12 |
| **PQQ** | 326.18 | 354.18 | **PQQP** | 423.24 | 451.23 | **PQQPT** | 524.28 | 552.28 |
| **PQQPTE** | 653.33 | 681.32 | **QQ** | 229.13 | 257.12 | **QQP** | 326.18 | 354.18 |
| **QQPT** | 427.23 | 455.22 | **QQPTE** | 556.27 | 584.27 | **QQPTEE** | 685.32 | 713.31 |
| **QP** | 198.12 | 226.12 | **QPT** | 299.17 | 327.17 | **QPTE** | 428.21 | 456.21 |
| **QPTEE** | 557.26 | 585.25 | **QPTEEQ** | 685.32 | 713.31 | **PT** | 171.11 | 199.11 |
| **PTE** | 300.16 | 328.15 | **PTEE** | 429.20 | 457.19 | **PTEEQ** | 557.26 | 585.25 |
| **TE** | 203.10 | 231.10 | **TEE** | 332.15 | 360.14 | **TEEQ** | 460.20 | 488.20 |
| **EE** | 231.10 | 259.09 | **EEQ** | 359.16 | 387.15 | **EQ** | 230.11 | 258.11 |

35. Tb11.02.2130

Match to: **Tb11.02.2130** Score: **59**

**hypothetical protein, conserved; Trypanosoma bruceichr 11Manual**

Nominal mass (M_r_): **38672**; Calculated pI value: **9.42**

NCBI BLAST search of [Tb11.02.2130](http://www.ncbi.nlm.nih.gov/blast/Blast.cgi?ALIGNMENTS=50&ALIGNMENT_VIEW=Pairwise&AUTO_FORMAT=Semiauto&CDD_SEARCH=on&CLIENT=web&COMPOSITION_BASED_STATISTICS=on&DATABASE=nr&DESCRIPTIONS=100&ENTREZ_QUERY=(none)&EXPECT=10&FILTER=L&FORMAT_BLOCK_ON_RESPAGE=None&FORMAT_OBJECT=Alignment&FORMAT_TYPE=HTML&GAPCOSTS=11+1&I_THRESH=0.001&LAYOUT=TwoWindows&MATRIX_NAME=BLOSUM62&NCBI_GI=on&PAGE=Proteins&PROGRAM=blastp&QUERY=MSNALESITAATQLRRAVMEAQRELDAKRELYLTRMARAHEIEETIAQGRAKLQDKLVRYYKFIQDNEVKRSRAMRKAVTEERIRKEREAQVEELTKKLQNLHDRSEELRGLYDVYSRYQRYLEEVLQRNDSDEYQGPRDIIQRWNTLHENTKVLQRRKTQLEEELLRNKNALNVKRQRKNNESVQLQNQLNELQARFGQLQKNIKIKQDELERCISQRSTTSRTISHVRMACKNLYDRCITWTAPYSGRGKFESREADVLFQLHVIGDCLRDFQDVIEAHHQRQQQLALARASRDDDA&SERVICE=plain&SET_DEFAULTS.x=9&SET_DEFAULTS.y=5&SHOW_OVERVIEW=on&WORD_SIZE=3&END_OF_HTTPGET=Yes) against nr

Unformatted [sequence string](../../../../D:%5CProteomic%20data%5C2010-1-8%5Ccgi%5Cgetseq.pl%3FTBA927_IPI+Tb11%2E02%2E2130+seq) for pasting into other applications

Fixed modifications: MMTS (C),(N-TERM)_iTRAQ,Lysine(K)_iTRAQ

Variable modifications: Oxidation (M)

Cleavage by Trypsin: cuts C-term side of KR unless next residue is P

Sequence Coverage: **9%**

Matched peptides shown in **Bold Red**

**1** M**SNALESITA ATQLRR**AVME AQRELDAKRE LYLTRMAR**AH EIEETIAQGR**

**51** AKLQDKLVRY YKFIQDNEVK RSRAMRKAVT EERIRKEREA QVEELTKKLQ

**101** NLHDRSEELR GLYDVYSRYQ RYLEEVLQRN DSDEYQGPRD IIQRWNTLHE

**151** NTKVLQRRKT QLEEELLRNK NALNVKRQRK NNESVQLQNQ LNELQARFGQ

**201** LQKNIKIKQD ELERCISQRS TTSRTISHVR MACKNLYDRC ITWTAPYSGR

**251** GKFESREADV LFQLHVIGDC LRDFQDVIEA HHQRQQQLAL ARASRDDDA

**Start - End Observed Mr(expt) Mr(calc) Delta Miss Sequence**

**2 - 16 1774.91 1773.91 1773.98 -0.07 1 M.SNALESITAATQLRR.A**  ([Ions score 6](../../../../D:%5CProteomic%20data%5C2010-1-8%5CZQ%5C1202.htm))

**39 - 50 1497.77 1496.77 1496.77 -0.00 0 R.AHEIEETIAQGR.A**  ([Ions score 59](../../../../D:%5CProteomic%20data%5C2010-1-8%5CZQ%5C1201.htm))

MS/MS Fragmentation of **AHEIEETIAQGR**
Found in **Tb11.02.2130**, hypothetical protein, conserved; Trypanosoma bruceichr 11Manual


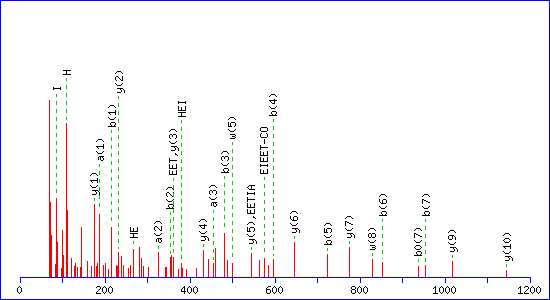


**MONOISOTOPIC mass of neutral peptide Mr(calc):** 1496.77

**Fixed modifications:** MMTS (C),(N-TERM)_iTRAQ,Lysine(K)_iTRAQ

**Ions Score:** 59 **Expect:** 0.00019

**Matches (Bold Red):** 30/172 fragment ions using 37 most intense peaks

| **#** | **Immon.** | **a** | **a*** | **a^0^** | **b** | **b*** | **b^0^** | **Seq.** | **v** | **w** | **w'** | **y** | **y*** | **y^0^** | **#** |
| --- | --- | --- | --- | --- | --- | --- | --- | --- | --- | --- | --- | --- | --- | --- | --- |
| **1** | 44.05 | **188.15** |  |  | **216.15** |  |  | **A** |  |  |  |  |  |  | **12** |
| **2** | **110.07** | **325.21** |  |  | **353.21** |  |  | **H** | 1200.59 |  |  | 1282.64 | 1265.61 | 1264.63 | **11** |
| **3** | 102.05 | **454.25** |  | 436.24 | **482.25** |  | 464.24 | **E** | 1071.54 | 1070.55 |  | **1145.58** | 1128.55 | 1127.57 | **10** |
| **4** | **86.10** | 567.34 |  | 549.33 | **595.33** |  | 577.32 | **I** | 958.46 | 971.48 | 985.49 | **1016.54** | 999.51 | 998.53 | **9** |
| **5** | 102.05 | 696.38 |  | 678.37 | **724.37** |  | 706.36 | **E** | 829.42 | **828.42** |  | 903.45 | 886.43 | 885.44 | **8** |
| **6** | 102.05 | 825.42 |  | 807.41 | **853.42** |  | 835.41 | **E** | 700.37 | 699.38 |  | **774.41** | 757.38 | 756.40 | **7** |
| **7** | 74.06 | 926.47 |  | 908.46 | **954.47** |  | **936.45** | **T** | 599.33 | 612.35 | 614.33 | **645.37** | 628.34 | 627.36 | **6** |
| **8** | **86.10** | 1039.55 |  | 1021.54 | 1067.55 |  | 1049.54 | **I** | 486.24 | **499.26** | 513.28 | **544.32** | 527.29 |  | **5** |
| **9** | 44.05 | 1110.59 |  | 1092.58 | 1138.59 |  | 1120.58 | **A** | 415.20 |  |  | **431.24** | 414.21 |  | **4** |
| **10** | 101.07 | 1238.65 | 1221.62 | 1220.64 | 1266.64 | 1249.62 | 1248.63 | **Q** | 287.15 | 286.15 |  | **360.20** | 343.17 |  | **3** |
| **11** | 30.03 | 1295.67 | 1278.64 | 1277.66 | 1323.67 | 1306.64 | 1305.66 | **G** |  |  |  | **232.14** | 215.11 |  | **2** |
| **12** | 129.11 |  |  |  |  |  |  | **R** | 74.02 | 73.03 |  | **175.12** | 158.09 |  | **1** |

| **Seq** | **ya** | **yb** | **Seq** | **ya** | **yb** | **Seq** | **ya** | **yb** |
| --- | --- | --- | --- | --- | --- | --- | --- | --- |
| **HE** | 239.11 | **267.11** | **HEI** | 352.20 | **380.19** | **HEIE** | 481.24 | 509.24 |
| **HEIEE** | 610.28 | 638.28 | **EI** | 215.14 | 243.13 | **EIE** | 344.18 | 372.18 |
| **EIEE** | 473.22 | 501.22 | **EIEET** | **574.27** | 602.27 | **EIEETI** | 687.36 | 715.35 |
| **IE** | 215.14 | 243.13 | **IEE** | 344.18 | 372.18 | **IEET** | 445.23 | 473.22 |
| **IEETI** | 558.31 | 586.31 | **IEETIA** | 629.35 | 657.35 | **EE** | 231.10 | 259.09 |
| **EET** | 332.15 | **360.14** | **EETI** | 445.23 | 473.22 | **EETIA** | 516.27 | **544.26** |
| **EETIAQ** | 644.32 | 672.32 | **ET** | 203.10 | 231.10 | **ETI** | 316.19 | 344.18 |
| **ETIA** | 387.22 | 415.22 | **ETIAQ** | 515.28 | 543.28 | **ETIAQG** | 572.30 | 600.30 |
| **TI** | 187.14 | 215.14 | **TIA** | 258.18 | 286.18 | **TIAQ** | 386.24 | 414.23 |
| **TIAQG** | 443.26 | 471.26 | **IA** | 157.13 | 185.13 | **IAQ** | 285.19 | 313.19 |
| **IAQG** | 342.21 | 370.21 | **AQ** | 172.11 | 200.10 | **AQG** | 229.13 | 257.12 |
| **QG** | 158.09 | 186.09 |  |  |  |  |  |  |

36. Tb927.2.5760

Match to: **Tb927.2.5760** Score: **59**

**1F7.250hypothetical protein, conserved; Trypanosoma bruceichr 2Manual**

Nominal mass (M_r_): **356214**; Calculated pI value: **5.99**

NCBI BLAST search of [Tb927.2.5760](http://www.ncbi.nlm.nih.gov/blast/Blast.cgi?ALIGNMENTS=50&ALIGNMENT_VIEW=Pairwise&AUTO_FORMAT=Semiauto&CDD_SEARCH=on&CLIENT=web&COMPOSITION_BASED_STATISTICS=on&DATABASE=nr&DESCRIPTIONS=100&ENTREZ_QUERY=(none)&EXPECT=10&FILTER=L&FORMAT_BLOCK_ON_RESPAGE=None&FORMAT_OBJECT=Alignment&FORMAT_TYPE=HTML&GAPCOSTS=11+1&I_THRESH=0.001&LAYOUT=TwoWindows&MATRIX_NAME=BLOSUM62&NCBI_GI=on&PAGE=Proteins&PROGRAM=blastp&QUERY=Tb927.2.5760&SERVICE=plain&SET_DEFAULTS.x=21&SET_DEFAULTS.y=7&SHOW_OVERVIEW=on&WORD_SIZE=3&END_OF_HTTPGET=Yes) against nr

Unformatted [sequence string](../../../../D:%5CProteomic%20data%5C2010-1-8%5Ccgi%5Cgetseq.pl%3FTBA927_IPI+Tb927%2E2%2E5760+seq) for pasting into other applications

Fixed modifications: MMTS (C),(N-TERM)_iTRAQ,Lysine(K)_iTRAQ

Variable modifications: Oxidation (M)

Cleavage by Trypsin: cuts C-term side of KR unless next residue is P

Sequence Coverage: **2%**

Matched peptides shown in **Bold Red**

**1** MGEADAPSGG QESNYLPVAA VVNSGSASGS GAHRGPRHQR ERSGGNENTE

**51** NVPPLPKTGL TNASFVRLED PRDIGEATST EILSEPDDEE EHLVTYHGVK

**101** LNGDNWVDVL R**GSRAVFDDA LGADVCDMLA LPR**SSVRDVK VGADGQSVMF

**151** GVRHPFSLQK DEVNKALESC RFTSTLKLYD LQTDSGPLET DSPVLKASES

**201** APPSLRDEES SQHGDIKGAS SVKKIKPRNH NIGACSITGH SASVEVLYGA

**251** IYDEDAAVTH HRVKFNGNNW DSIVHHK**GMA IREAFDTDVS NALGLPR**GSV

**301** EDVTLAPDGL SMSFSVRHPA SLRRKDVNKI LAKCPFENTW RLYESKKSFN

**351** NSRVASFVLR SSVNAPPSLR AEEDVGVVLG DWDANPPFEF LVPATASQMT

**401** VVRPPNAASG ECNINSMPDD ILINTEGEGF GTVTRHEVKF EGDNWGVVHE

**451** KKRVAFDEAF DADICGALGV PRGSVEDMVL ASDGQLVSFG VRHPASLGKK

**501** DINKALSSCP FENTWKLYEP KKSASPSELN SPVMKATKGE LAPLRAGEVV

**551** GPTPLEVGGP RDLAADGATA KTSGQPVDVE TNVLTRHRVK FHGDNWGVVH

**601** EKKRVAFDEA FDADICGALG VPRGSVEDMV LASDGQLVSF GVRHPASLGK

**651** KDINKALSSC PFENTWKLYE PKKSASPSEL NSPVMKATKG ELAPLRAGEV

**701** VGPTPLEVGG PRDLAADGAT AKTSGQPVDV ETNVLTRHRV KFHGDNWGVV

**751** HEKKRVAFDE AFDADICGAL GVPRGSVEDM VLASDGQLVS FGVRHPASLG

**801** KKDINKALSS CPFENTWKLY EPKKSASPSE LNSPVMKATK GELAPLRAGE

**851** VVGPTPLEVG GPRDLAADGA TAKTSGQPVE REDGCVTSHA LKLEGDNWGA

**901** VYEKKRAAFD EAFDADICDT LGVPRGSVED MVLASDGQLV SFGVRHPASL

**951** GKKDINKALS SCPFENTWKL YEPKKSASPS ELNSPVMKAT KGELAPLRAG

**1001** EVVGPTPLEV GGPRDLAADG ATAKTSGQPV EREDGCVTSH ALKLEGDNWG

**1051** AVYEKKRAAF DEAFDADICD TLGVPRGSVE DMVLASDGQL VSFGVRHPAS

**1101** LGKKDINKAL SSCPFENTWK LYEPKKSASP SRFDSLVLMT TKGELAPLRA

**1151** EEEAAGPLPD ASDAFPSATA AVSNLLPAKA IRSRYFSSGE CNITTSGATP

**1201** IALYGALCDS DTVVTRHRVR FNGENWDLVL QRRRAELDDA LDADVSEALD

**1251** LPRGSVSDIE YVPDGLTASF GVRHPAAFRR RDIARLLSKG QFANLWKMYP

**1301** RSRNIDTLGP SLSSLKASEK APPNIVGGEV VPPVPNFCQS RAKVDAAGLP

**1351** TRASDTVTPS DYTNGQQSVM KTEKRGSVVD GGDTIITCHR VKLSGANWVE

**1401** VVETKRAAFD EAFDTDVCEA LGFPLGCVED VLLGPDGHSV TFAVRHSASL

**1451** QKSDVKKVLR LCPFANTLQL YHSRRTLPSS EVASTVLRAT EKEKPAPTPR

**1501** SHGSAKPSTV KVAASCDLEG RGADGDPAFV EVFNGLISDE DTLVTRHGVL

**1551** LNGENWLKLH ESKSAAFNDA FDADVCEALG LPCGNVEGIK LNPDGHLVTF

**1601** GVRHPISQRR EDINRALGKC RYQNLMKLYN SRSAVYSCVA TSPMLRATET

**1651** ARAALATQES ISQPAVSEVA NTTSVGVAKS RNLLDGSAAA SNSASVAGLH

**1701** AFLSDQPVVT QHGIKFSGAN WADVIEGRAM FDDALDADVC EAVGLPRGSV

**1751** EDILLSSDGR SVAFGVRHPA SISKGDINQA LDNCRFVNTL RLYNMRSAVY

**1801** SCVAKSPMLR ATETARAALA TQESISQPAV SEVANTTSVG VAKSRNLLDG

**1851** SAAASNSASV AGLHAFLSDQ PVVTQHGIKF SGANWADVIE GRAMFDDALD

**1901** ADVCEAVGLP RGSVEDILLS SDGRSVAFGV RHPASISKGD INQALDNCRF

**1951** VNTLRLYVSR CPPVVYEASR PPLKAAEAEQ LSLKAAETKR PPLKAAEAEQ

**2001** SSLKAAETKR PPLKATEAEQ SSLKAAETKR PPLKAAEAEQ SSLKAAETKR

**2051** PPLKAAEVGR PPLKATEAEQ SSLKAAETKR PPLKAAEVGR PPLKATEAEQ

**2101** SSLKAAETKR PPLKATEAEQ SPLKVTEARR APPARATEVE RPPLKVVEIS

**2151** SPTSFVAGPR SVGLVNPPSL VDTGIIGSSS ASFDILYGSL KDEGAVVTEY

**2201** RAKLNGSHWR VLLQHRFLEF EEAFYGDVCD ALSLTKGSVD NMELDSDMQA

**2251** FSFGVRHRRS LEPSAVLRQL GEYHFSKTRE FCDSQKRFGV VVDDHDQLLT

**2301** LEIASKQELP EADDGCDEDL RHLQTSGRSD LPSSPSSPLC IKKTNTRTMH

**2351** FGNTGAGAVR LPKTRKYEAA PANATVTQHR VQFVAENCGH VADETREKVP

**2401** QALNLDVCDA LGISWKCVEE VTLSPDGRLV TFGVRHSPKM SRADVRSALI

**2451** AHDFPYTWKV YEPDTLVEFE SGVERTTAEQ KSQATPKATS TSSIYFSPSS

**2501** VPPFVRVAEP QNFVKDSFTI SNTASLEVLH TAIGDGTVVT KHRVKFNGDN

**2551** WGAVLEKKRA AFDEAFDADV CDALDLPRGS VEGITLGSDG LSIDFGVRHP

**2601** ASLHKDEVDH FLSTSEFPAM WNLYEPKKKV LSSIVMGDTE ERETTSFSTS

**2651** LPQRASPVSA KEFPSCLHPA PGVASSSVPM DRADGASGSG NVCHLYRVGF

**2701** VGGFWSGIVD KRFTAAVDCF MRDAAVGEGL VPRSVERVVS LDSGNVVFSV

**2751** WMEHAAALSQ SEVCTAMDNA PFPSMWKLYD DLINEGVVGR TTTLHRVGLV

**2801** GSEWAHVANV DLVK**EVFIQD VAEALR**LSPE DINVNEYTVN DKLVIGFYVS

**2851** HSHTLTEVDI DDMLANAPLS RVWGLCPVPG TNSWKAAHCK GSARSNSGSF

**2901** PHTEPLRGGR FRDLSPIPLS GGIPAETPHD RRSNSQYRRS LGPLQGVVSP

**2951** PMRRYDGHNQ HSGRFYQANK RSPGQFSPPN QPVVNTKGVH AMRMSPQSGR

**3001** FKAGSARGGG SLIAGSDPVR ASLPRVDLLK VGELLALMRR RQLEEGSTRL

**3051** PPLTGR**AQPY PSCAPSTVK**R YVGSK

**Start - End Observed Mr(expt) Mr(calc) Delta Miss Sequence**

**112 - 133 2498.34 2497.33 2497.19 0.14 1 R.GSRAVFDDALGADVCDMLALPR.S**  Oxidation (M) ([Ions score 1](../../../../D:%5CProteomic%20data%5C2010-1-8%5CZQ%5C1207.htm))

**278 - 297 2293.11 2292.11 2292.17 -0.06 1 K.GMAIREAFDTDVSNALGLPR.G**  Oxidation (M) ([Ions score 5](../../../../D:%5CProteomic%20data%5C2010-1-8%5CZQ%5C1206.htm))

**2815 - 2826 1533.83 1532.82 1532.83 -0.01 0 K.EVFIQDVAEALR.L**  ([Ions score 59](../../../../D:%5CProteomic%20data%5C2010-1-8%5CZQ%5C1204.htm))

**3057 - 3069 1682.84 1681.83 1681.75 0.08 0 R.AQPYPSCAPSTVK.R**  ([Ions score 2](../../../../D:%5CProteomic%20data%5C2010-1-8%5CZQ%5C1205.htm))

MS/MS Fragmentation of **EVFIQDVAEALR**
Found in **Tb927.2.5760**, 1F7.250hypothetical protein, conserved; Trypanosoma bruceichr 2Manual


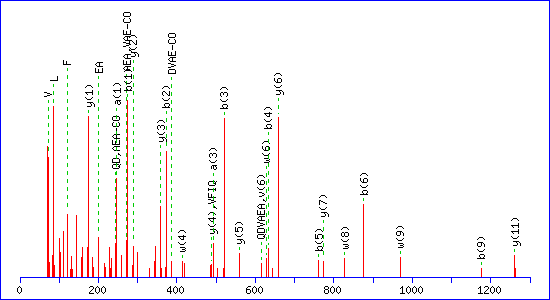


**MONOISOTOPIC mass of neutral peptide Mr(calc):** 1532.83

**Fixed modifications:** MMTS (C),(N-TERM)_iTRAQ,Lysine(K)_iTRAQ

**Ions Score:** 59 **Expect:** 0.00019

**Matches (Bold Red):** 36/191 fragment ions using 33 most intense peaks

| **#** | **Immon.** | **a** | **a*** | **a^0^** | **b** | **b*** | **b^0^** | **Seq.** | **v** | **w** | **w'** | **y** | **y*** | **y^0^** | **#** |
| --- | --- | --- | --- | --- | --- | --- | --- | --- | --- | --- | --- | --- | --- | --- | --- |
| **1** | 102.05 | **246.16** |  | 228.15 | **274.15** |  | 256.14 | **E** |  |  |  |  |  |  | **12** |
| **2** | **72.08** | 345.23 |  | 327.22 | **373.22** |  | 355.21 | **V** | 1216.63 | 1229.65 |  | **1260.69** | 1243.67 | 1242.68 | **11** |
| **3** | **120.08** | **492.29** |  | 474.28 | **520.29** |  | 502.28 | **F** | 1069.56 |  |  | 1161.63 | 1144.60 | 1143.62 | **10** |
| **4** | **86.10** | 605.38 |  | 587.37 | **633.37** |  | 615.36 | **I** | 956.48 | **969.50** | 983.52 | 1014.56 | 997.53 | 996.55 | **9** |
| **5** | 101.07 | 733.44 | 716.41 | 715.43 | **761.43** | 744.41 | 743.42 | **Q** | 828.42 | **827.43** |  | 901.47 | 884.45 | 883.46 | **8** |
| **6** | 88.04 | 848.46 | 831.44 | 830.45 | **876.46** | 859.43 | 858.45 | **D** | 713.39 | 712.40 |  | **773.42** | 756.39 | 755.40 | **7** |
| **7** | **72.08** | 947.53 | 930.51 | 929.52 | 975.53 | 958.50 | 957.52 | **V** | **614.33** | **627.35** |  | **658.39** | 641.36 | 640.38 | **6** |
| **8** | 44.05 | 1018.57 | 1001.54 | 1000.56 | 1046.56 | 1029.54 | 1028.55 | **A** | 543.29 |  |  | **559.32** | 542.29 | 541.31 | **5** |
| **9** | 102.05 | 1147.61 | 1130.59 | 1129.60 | **1175.61** | 1158.58 | 1157.60 | **E** | 414.25 | **413.25** |  | **488.28** | 471.26 | 470.27 | **4** |
| **10** | 44.05 | 1218.65 | 1201.62 | 1200.64 | 1246.64 | 1229.62 | 1228.63 | **A** | 343.21 |  |  | **359.24** | 342.21 |  | **3** |
| **11** | **86.10** | 1331.73 | 1314.71 | 1313.72 | 1359.73 | 1342.70 | 1341.72 | **L** | 230.12 | 229.13 |  | **288.20** | 271.18 |  | **2** |
| **12** | 129.11 |  |  |  |  |  |  | **R** | 74.02 | 73.03 |  | **175.12** | 158.09 |  | **1** |

| **Seq** | **ya** | **yb** | **Seq** | **ya** | **yb** | **Seq** | **ya** | **yb** |
| --- | --- | --- | --- | --- | --- | --- | --- | --- |
| **VF** | 219.15 | 247.14 | **VFI** | 332.23 | 360.23 | **VFIQ** | 460.29 | **488.29** |
| **VFIQD** | 575.32 | 603.31 | **VFIQDV** | 674.39 | 702.38 | **FI** | 233.16 | 261.16 |
| **FIQ** | 361.22 | 389.22 | **FIQD** | 476.25 | 504.25 | **FIQDV** | 575.32 | 603.31 |
| **FIQDVA** | 646.36 | 674.35 | **IQ** | 214.16 | 242.15 | **IQD** | 329.18 | 357.18 |
| **IQDV** | 428.25 | 456.25 | **IQDVA** | 499.29 | 527.28 | **IQDVAE** | 628.33 | 656.32 |
| **IQDVAEA** | 699.37 | 727.36 | **QD** | 216.10 | **244.09** | **QDV** | 315.17 | 343.16 |
| **QDVA** | 386.20 | 414.20 | **QDVAE** | 515.25 | 543.24 | **QDVAEA** | 586.28 | **614.28** |
| **QDVAEAL** | 699.37 | 727.36 | **DV** | 187.11 | 215.10 | **DVA** | 258.14 | 286.14 |
| **DVAE** | **387.19** | 415.18 | **DVAEA** | 458.22 | 486.22 | **DVAEAL** | 571.31 | 599.30 |
| **VA** | 143.12 | 171.11 | **VAE** | **272.16** | 300.16 | **VAEA** | 343.20 | 371.19 |
| **VAEAL** | 456.28 | 484.28 | **AE** | 173.09 | **201.09** | **AEA** | **244.13** | **272.12** |
| **AEAL** | 357.21 | 385.21 | **EA** | 173.09 | **201.09** | **EAL** | 286.18 | 314.17 |
| **AL** | 157.13 | 185.13 |  |  |  |  |  |  |

37. Tb927.5.2850

Match to: **Tb927.5.2850** Score: **58**

**radial spoke protein RSP2, putative; Trypanosoma bruceichr 5Manual**

Nominal mass (M_r_): **61525**; Calculated pI value: **4.76**

NCBI BLAST search of [Tb927.5.2850](http://www.ncbi.nlm.nih.gov/blast/Blast.cgi?ALIGNMENTS=50&ALIGNMENT_VIEW=Pairwise&AUTO_FORMAT=Semiauto&CDD_SEARCH=on&CLIENT=web&COMPOSITION_BASED_STATISTICS=on&DATABASE=nr&DESCRIPTIONS=100&ENTREZ_QUERY=(none)&EXPECT=10&FILTER=L&FORMAT_BLOCK_ON_RESPAGE=None&FORMAT_OBJECT=Alignment&FORMAT_TYPE=HTML&GAPCOSTS=11+1&I_THRESH=0.001&LAYOUT=TwoWindows&MATRIX_NAME=BLOSUM62&NCBI_GI=on&PAGE=Proteins&PROGRAM=blastp&QUERY=MTESAYLKCSVGPVLAKAVAETVLAQPSNPQEYIALYLLHVLQEEQNAAIAATRQAKVEALRQAWAGRRALREKRAADTIQRFFRQCQAVLRARRAEEEELWNKYEEAEAEADDLLGDVAGEKDHSGDALPDAADVDDAAAAVEDARVEFYKAHRFMLYIRKALLGMLKKELVDRREEVRMEQDKMHDALEVATEEAQKKDEAEAIAAATKGTLPSSDAMEKLVRQVTLRQHEKISAPMILFRVLRCWCYFLFDSTPKQVSTPADVAALLKPFKLMQLLRAFNPVGSYQRSRPLRLEDNLQNANDMNSGDDMQDGDVPIPQPKPRQARRVGRVLRVLLHDGEYICGVNPADHIDAEGSGADEEHEALEAAAAAADRAANITSRVEETAKKHSVILYALLRLLRTASAYRDARDKWLQLLTQAGREVPATVELPEEDVNDPNDEEALRDEDDEVDEAAVRRLLLQIGVDTDEALAKLWIEADSVERAKWEGIAAARLEEEGQEEGSGGDGEGSDGDEGN&SERVICE=plain&SET_DEFAULTS.x=9&SET_DEFAULTS.y=5&SHOW_OVERVIEW=on&WORD_SIZE=3&END_OF_HTTPGET=Yes) against nr

Unformatted [sequence string](../../../../D:%5CProteomic%20data%5C2010-1-8%5Ccgi%5Cgetseq.pl%3FTBA927_IPI+Tb927%2E5%2E2850+seq) for pasting into other applications

Fixed modifications: MMTS (C),(N-TERM)_iTRAQ,Lysine(K)_iTRAQ

Variable modifications: Oxidation (M)

Cleavage by Trypsin: cuts C-term side of KR unless next residue is P

Sequence Coverage: **5%**

Matched peptides shown in **Bold Red**

**1** MTESAYLKCS VGPVLAKAVA ETVLAQPSNP QEYIALYLLH VLQEEQNAAI

**51** AATRQAKVEA LRQAWAGRRA LREKRAADTI QRFFRQCQAV LRARRAEEEE

**101** LWNKYEEAEA EADDLLGDVA GEKDHSGDAL PDAADVDDAA AAVEDARVEF

**151** YKAHRFMLYI RKALLGMLKK ELVDRREEVR MEQDKMHDAL EVATEEAQKK

**201** DEAEAIAAAT KGTLPSSDAM EK**LVRQVTLR** QHEKISAPMI LFRVLRCWCY

**251** FLFDSTPKQV STPADVAALL KPFKLMQLLR **AFNPVGSYQR** SRPLRLEDNL

**301** QNANDMNSGD DMQDGDVPIP QPKPRQARRV GRVLRVLLHD GEYICGVNPA

**351** DHIDAEGSGA DEEHEALEAA AAAADRAANI TSRVEETAKK HSVILYALLR

**401** LLRTASAYRD ARDKWLQLLT QAGREVPATV ELPEEDVNDP NDEEALRDED

**451** DEVDEAAVRR LLLQIGVDTD EALAK**LWIEA DSVER**AKWEG IAAARLEEEG

**501** QEEGSGGDGE GSDGDEGN

**Start - End Observed Mr(expt) Mr(calc) Delta Miss Sequence**

**223 - 230 1128.65 1127.64 1127.73 -0.08 1 K.LVRQVTLR.Q**  ([Ions score 9](../../../../D:%5CProteomic%20data%5C2010-1-8%5CZQ%5C1209.htm))

**281 - 290 1282.66 1281.66 1281.66 -0.00 0 R.AFNPVGSYQR.S**  ([Ions score 58](../../../../D:%5CProteomic%20data%5C2010-1-8%5CZQ%5C1210.htm))

**476 - 485 1361.68 1360.68 1360.71 -0.03 0 K.LWIEADSVER.A**  ([Ions score 15](../../../../D:%5CProteomic%20data%5C2010-1-8%5CZQ%5C1211.htm))

MS/MS Fragmentation of **AFNPVGSYQR**
Found in **Tb927.5.2850**, radial spoke protein RSP2, putative; Trypanosoma bruceichr 5Manual


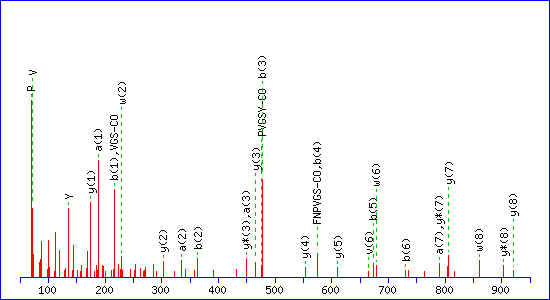


**MONOISOTOPIC mass of neutral peptide Mr(calc):** 1281.66

**Fixed modifications:** MMTS (C),(N-TERM)_iTRAQ,Lysine(K)_iTRAQ

**Ions Score:** 58 **Expect:** 0.00023

**Matches (Bold Red):** 30/136 fragment ions using 27 most intense peaks

| **#** | **Immon.** | **a** | **a*** | **a^0^** | **b** | **b*** | **b^0^** | **Seq.** | **v** | **w** | **y** | **y*** | **y^0^** | **#** |
| --- | --- | --- | --- | --- | --- | --- | --- | --- | --- | --- | --- | --- | --- | --- |
| **1** | 44.05 | **188.15** |  |  | **216.15** |  |  | **A** |  |  |  |  |  | **10** |
| **2** | 120.08 | **335.22** |  |  | **363.22** |  |  | **F** | 975.46 |  | 1067.53 | 1050.50 | 1049.52 | **9** |
| **3** | 87.06 | **449.26** | 432.24 |  | **477.26** | 460.23 |  | **N** | 861.42 | **860.43** | **920.46** | **903.43** | 902.45 | **8** |
| **4** | **70.07** | 546.32 | 529.29 |  | **574.31** | 557.28 |  | **P** | 764.37 | 763.37 | **806.42** | **789.39** | 788.40 | **7** |
| **5** | **72.08** | 645.38 | 628.36 |  | **673.38** | 656.35 |  | **V** | **665.30** | **678.32** | 709.36 | 692.34 | 691.35 | **6** |
| **6** | 30.03 | 702.41 | 685.38 |  | **730.40** | 713.37 |  | **G** |  |  | **610.29** | 593.27 | 592.28 | **5** |
| **7** | 60.04 | **789.44** | 772.41 | 771.43 | 817.43 | 800.41 | 799.42 | **S** | 521.25 | 520.25 | **553.27** | 536.25 | 535.26 | **4** |
| **8** | **136.08** | 952.50 | 935.47 | 934.49 | 980.50 | 963.47 | 962.49 | **Y** | 358.18 |  | **466.24** | **449.21** |  | **3** |
| **9** | 101.07 | 1080.56 | 1063.53 | 1062.55 | 1108.55 | 1091.53 | 1090.54 | **Q** | 230.12 | **229.13** | **303.18** | 286.15 |  | **2** |
| **10** | 129.11 |  |  |  |  |  |  | **R** | 74.02 | 73.03 | **175.12** | 158.09 |  | **1** |

| **Seq** | **ya** | **yb** | **Seq** | **ya** | **yb** | **Seq** | **ya** | **yb** |
| --- | --- | --- | --- | --- | --- | --- | --- | --- |
| **FN** | 234.12 | 262.12 | **FNP** | 331.18 | 359.17 | **FNPV** | 430.24 | 458.24 |
| **FNPVG** | 487.27 | 515.26 | **FNPVGS** | **574.30** | 602.29 | **NP** | 184.11 | 212.10 |
| **NPV** | 283.18 | 311.17 | **NPVG** | 340.20 | 368.19 | **NPVGS** | 427.23 | 455.22 |
| **NPVGSY** | 590.29 | 618.29 | **PV** | 169.13 | 197.13 | **PVG** | 226.15 | 254.15 |
| **PVGS** | 313.19 | 341.18 | **PVGSY** | **476.25** | 504.25 | **PVGSYQ** | 604.31 | 632.30 |
| **VG** | 129.10 | 157.10 | **VGS** | **216.13** | 244.13 | **VGSY** | 379.20 | 407.19 |
| **VGSYQ** | 507.26 | 535.25 | **GS** | 117.07 | 145.06 | **GSY** | 280.13 | 308.12 |
| **GSYQ** | 408.19 | 436.18 | **SY** | 223.11 | 251.10 | **SYQ** | 351.17 | 379.16 |
| **YQ** | 264.13 | 292.13 |  |  |  |  |  |  |

38. Tb09.211.1790

Match to: **Tb09.211.1790** Score: **56**

**TAX-1; Trypanosoma bruceichr 9Manual**

Nominal mass (M_r_): **44253**; Calculated pI value: **6.24**

NCBI BLAST search of [Tb09.211.1790](http://www.ncbi.nlm.nih.gov/blast/Blast.cgi?ALIGNMENTS=50&ALIGNMENT_VIEW=Pairwise&AUTO_FORMAT=Semiauto&CDD_SEARCH=on&CLIENT=web&COMPOSITION_BASED_STATISTICS=on&DATABASE=nr&DESCRIPTIONS=100&ENTREZ_QUERY=(none)&EXPECT=10&FILTER=L&FORMAT_BLOCK_ON_RESPAGE=None&FORMAT_OBJECT=Alignment&FORMAT_TYPE=HTML&GAPCOSTS=11+1&I_THRESH=0.001&LAYOUT=TwoWindows&MATRIX_NAME=BLOSUM62&NCBI_GI=on&PAGE=Proteins&PROGRAM=blastp&QUERY=MQGANLKVLPSTRAAAKELVDPLDITNVGWSTLEPKFDELMQLMDAPSSGINALAARSAHREKVGAVIEQLLTRAQDESRRLLVEGNGEAAAEAGVKTLRLKERFYGKGSVKLVPAHFHLARTNQFLKRYGNAEEILSLAHFIILQNPDEADATIKAELHQTFGLLYAADNKLDVSVKHLTCATYYLSVMNGPEHVLTTFAYFDLANVFATKACMEAAMALYDTVKNIWLKHLRRVLKDIVDETMAAKLVKRYDDDEVTHEVGHASARAFGKENLADVSKMLFGIFSIQKERLTISHPTTARAQFLLGLYLLWVNKNDEAAEHLLSARTTSQKFYGERHPIVQDIEDWCIWFEIPFRGVAAEQ&SERVICE=plain&SET_DEFAULTS.x=9&SET_DEFAULTS.y=5&SHOW_OVERVIEW=on&WORD_SIZE=3&END_OF_HTTPGET=Yes) against nr

Unformatted [sequence string](../../../../D:%5CProteomic%20data%5C2010-1-8%5Ccgi%5Cgetseq.pl%3FTBA927_IPI+Tb09%2E211%2E1790+seq) for pasting into other applications

Fixed modifications: MMTS (C),(N-TERM)_iTRAQ,Lysine(K)_iTRAQ

Variable modifications: Oxidation (M)

Cleavage by Trypsin: cuts C-term side of KR unless next residue is P

Sequence Coverage: **3%**

Matched peptides shown in **Bold Red**

**1** MQGANLKVLP STRAAAKELV DPLDITNVGW STLEPKFDEL MQLMDAPSSG

**51** INALAARSAH R**EKVGAVIEQ LLTR**AQDESR RLLVEGNGEA AAEAGVKTLR

**101** LKERFYGKGS VKLVPAHFHL ARTNQFLKRY GNAEEILSLA HFIILQNPDE

**151** ADATIKAELH QTFGLLYAAD NKLDVSVKHL TCATYYLSVM NGPEHVLTTF

**201** AYFDLANVFA TKACMEAAMA LYDTVKNIWL KHLRRVLKDI VDETMAAKLV

**251** KRYDDDEVTH EVGHASARAF GKENLADVSK MLFGIFSIQK ERLTISHPTT

**301** ARAQFLLGLY LLWVNKNDEA AEHLLSARTT SQKFYGERHP IVQDIEDWCI

**351** WFEIPFRGVA AEQ

MS/MS Fragmentation of **VGAVIEQLLTR**
Found in **Tb09.211.1790**, TAX-1; Trypanosoma bruceichr 9Manual


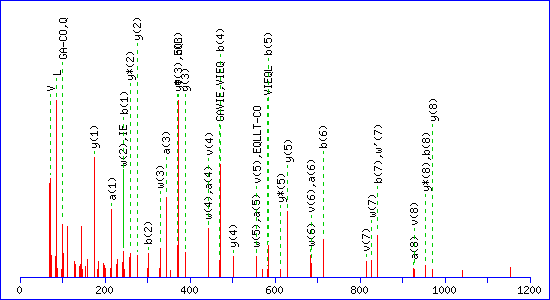


**MONOISOTOPIC mass of neutral peptide Mr(calc):** 1341.81

**Fixed modifications:** MMTS (C),(N-TERM)_iTRAQ,Lysine(K)_iTRAQ

**Ions Score:** 56 **Expect:** 0.00017

**Matches (Bold Red):** 51/158 fragment ions using 39 most intense peaks

| **#** | **Immon.** | **a** | **a*** | **a^0^** | **b** | **b*** | **b^0^** | **Seq.** | **v** | **w** | **w'** | **y** | **y*** | **y^0^** | **#** |
| --- | --- | --- | --- | --- | --- | --- | --- | --- | --- | --- | --- | --- | --- | --- | --- |
| **1** | **72.08** | **216.18** |  |  | **244.18** |  |  | **V** |  |  |  |  |  |  | **11** |
| **2** | 30.03 | 273.20 |  |  | **301.20** |  |  | **G** |  |  |  | 1099.65 | 1082.62 | 1081.64 | **10** |
| **3** | 44.05 | **344.24** |  |  | **372.24** |  |  | **A** | 1026.59 |  |  | 1042.63 | 1025.60 | 1024.61 | **9** |
| **4** | **72.08** | **443.31** |  |  | **471.31** |  |  | **V** | **927.53** | 940.55 |  | **971.59** | **954.56** | 953.58 | **8** |
| **5** | **86.10** | **556.39** |  |  | **584.39** |  |  | **I** | **814.44** | **827.46** | **841.48** | 872.52 | 855.49 | 854.51 | **7** |
| **6** | 102.05 | **685.44** |  | 667.43 | **713.43** |  | 695.42 | **E** | **685.40** | **684.40** |  | 759.44 | 742.41 | 741.43 | **6** |
| **7** | **101.07** | 813.50 | 796.47 | 795.48 | **841.49** | 824.46 | 823.48 | **Q** | **557.34** | **556.35** |  | **630.39** | **613.37** | 612.38 | **5** |
| **8** | **86.10** | **926.58** | 909.55 | 908.57 | **954.57** | 937.55 | 936.56 | **L** | **444.26** | **443.26** |  | **502.33** | 485.31 | 484.32 | **4** |
| **9** | **86.10** | 1039.66 | 1022.64 | 1021.65 | 1067.66 | 1050.63 | 1049.65 | **L** | 331.17 | **330.18** |  | **389.25** | **372.22** | **371.24** | **3** |
| **10** | 74.06 | 1140.71 | 1123.68 | 1122.70 | 1168.71 | 1151.68 | 1150.70 | **T** | 230.12 | **243.15** | 245.12 | **276.17** | **259.14** | 258.16 | **2** |
| **11** | 129.11 |  |  |  |  |  |  | **R** | 74.02 | 73.03 |  | **175.12** | 158.09 |  | **1** |

| **Seq** | **ya** | **yb** | **Seq** | **ya** | **yb** | **Seq** | **ya** | **yb** |
| --- | --- | --- | --- | --- | --- | --- | --- | --- |
| **GA** | **101.07** | 129.07 | **GAV** | 200.14 | 228.13 | **GAVI** | 313.22 | 341.22 |
| **GAVIE** | 442.27 | **470.26** | **GAVIEQ** | 570.32 | 598.32 | **GAVIEQL** | 683.41 | 711.40 |
| **AV** | 143.12 | 171.11 | **AVI** | 256.20 | 284.20 | **AVIE** | 385.24 | 413.24 |
| **AVIEQ** | 513.30 | 541.30 | **AVIEQL** | 626.39 | 654.38 | **VI** | 185.16 | 213.16 |
| **VIE** | 314.21 | 342.20 | **VIEQ** | 442.27 | **470.26** | **VIEQL** | 555.35 | **583.34** |
| **VIEQLL** | 668.43 | 696.43 | **IE** | 215.14 | **243.13** | **IEQ** | 343.20 | **371.19** |
| **IEQL** | 456.28 | 484.28 | **IEQLL** | 569.37 | 597.36 | **IEQLLT** | 670.41 | 698.41 |
| **EQ** | 230.11 | 258.11 | **EQL** | 343.20 | **371.19** | **EQLL** | 456.28 | 484.28 |
| **EQLLT** | **557.33** | 585.32 | **QL** | 214.16 | 242.15 | **QLL** | 327.24 | 355.23 |
| **QLLT** | 428.29 | 456.28 | **LL** | 199.18 | 227.18 | **LLT** | 300.23 | 328.22 |
| **LT** | 187.14 | 215.14 |  |  |  |  |  |  |

39. Tb10.70.4610

Match to: **Tb10.70.4610** Score: **56**

**hypothetical protein, conserved; Trypanosoma bruceichr 10Manual**

Nominal mass (M_r_): **220497**; Calculated pI value: **7.76**

NCBI BLAST search of [Tb10.70.4610](http://www.ncbi.nlm.nih.gov/blast/Blast.cgi?ALIGNMENTS=50&ALIGNMENT_VIEW=Pairwise&AUTO_FORMAT=Semiauto&CDD_SEARCH=on&CLIENT=web&COMPOSITION_BASED_STATISTICS=on&DATABASE=nr&DESCRIPTIONS=100&ENTREZ_QUERY=(none)&EXPECT=10&FILTER=L&FORMAT_BLOCK_ON_RESPAGE=None&FORMAT_OBJECT=Alignment&FORMAT_TYPE=HTML&GAPCOSTS=11+1&I_THRESH=0.001&LAYOUT=TwoWindows&MATRIX_NAME=BLOSUM62&NCBI_GI=on&PAGE=Proteins&PROGRAM=blastp&QUERY=Tb10.70.4610&SERVICE=plain&SET_DEFAULTS.x=21&SET_DEFAULTS.y=7&SHOW_OVERVIEW=on&WORD_SIZE=3&END_OF_HTTPGET=Yes) against nr

Unformatted [sequence string](../../../../D:%5CProteomic%20data%5C2010-1-8%5Ccgi%5Cgetseq.pl%3FTBA927_IPI+Tb10%2E70%2E4610+seq) for pasting into other applications

Fixed modifications: MMTS (C),(N-TERM)_iTRAQ,Lysine(K)_iTRAQ

Variable modifications: Oxidation (M)

Cleavage by Trypsin: cuts C-term side of KR unless next residue is P

Sequence Coverage: **2%**

Matched peptides shown in **Bold Red**

**1** MFKAIASGKS DAVWYCRCFN AFIFFLFFPH LQTKYCSFHI PCMRYAKFFF

**51** KSMFVFFYAN RLLHTHTRSK EKKRRKDLWS MGDADVATDQ QQQRLKQVPN

**101** RGDAINANAA NSFDDEDGNQ CLWHRRSREV HAHLKRRVAG ILTQIRCTSS

**151** ANHFDRHRVE VDELLRCDAP MGFTPSMILA GVPDIIHLLT DAVRDRRVLE

**201** VGRENRQAIS ALTPICQSSN FLITPNPFFV KEIASSIWRD SSKFKGDRGN

**251** NTPSNAMSSV GSNGAKGDNI DCFLPPLVRH AK**GSAPQEYP HTTSASPTR**E

**301** GKRLVARIGK SNEVTTAAGT AAGSRKCVRD GSSSPLDKSG PTLPRIDANR

**351** QSANRHVNGT GQGNQPLQQQ SNGFPSACAV SDAANAKCAP LLCDIPSTVE

**401** AMMYHCAFPL VCITPEDYES RASATHILVS TLSSIIRKTS DNNLHTECEG

**451** ISGVDNVASQ STSDDGKPGS NINGDYNCNA PRPTNAEQKN CDLEHKNMLY

**501** RLRRAALHTL HALLSIMDSE NASLVNKHFA ERDRQLYDAF GDINFSTDNT

**551** IMDMCSPMDE TR**ENDNER**VE SVSVKVLSGG TMGSPRAVSP RRRLSTACVK

**601** VMQRTQRNML VRRVAPLSVY NNAVDVNVLI PLLERWTFIL DKGTGSNGVL

**651** DSARFLTQRP GAWEVEERSN KMANKKLAMA SITRCGQEGH HVIPAEMHSE

**701** KITTGGGRDE TVGAVVRMCE KDVEEIYLLL RICLQLSLYK HHLNVSARMA

**751** DTFGGTGNNT FKSIPVLVCL TMARCMEGDR HLPLCVECLW NMLDVIPEEA

**801** AKEILHHTGD ESGAHFQQEG KK**QSEYATTN SPLSR**LSATE ALLHCLLQFL

**851** VSGHRLEYRE LRNDIIVLML IVLRTHTAGL RQQEETLLYN SELEHPALPP

**901** GSTIEAITTM AKTVFDLVCG PELQVFGTTA ELTLESPELF RRYHTVLSPT

**951** TRRENLQFKV LGWRFLEAFH EWQNVRLTLE LRCRPHMSRT TLSTRAPAPS

**1001** GYVFDTSNTV FDVCGAGFVD LLLMYVDTEC SNELVLAWSR EDLLTLQEES

**1051** WRVILAMVEP HAEHSVTKAF EGVGADHTWQ NGKEVNLFSG NPGGGLWVEG

**1101** VGEEWPLLQA DLAFVGTNGI ACAMRYIQET CNGDTEMIGY LAIHVLSVLS

**1151** RFPIHREALL ALRTPAPTTG EPVPLLLATT VQVIQRLFNQ VENSAKINSN

**1201** SNGTRGMPKP DGISRAESVQ HVVRDATVSK GNYGSSVRWQ KDSEGRKKTL

**1251** KESQSVQTRP VHKGSSLFAF SNAGCSSTQR TYGMLQLSNT HVYFPAQTIS

**1301** VFLKCLSLLH NVADGSRDEV VATQRAFILM DGITHILRLV WNSLTPSTSN

**1351** SKFSRFQVEE RNKELLAAAL NCVRVFLIGN EAGQAQFVRG DGVNTLLTVI

**1401** EAHLGEDSGQ LFDTVEGGHP PALHLTLTIL ADLLRENAEA RDAFLKWFNY

**1451** TSQVVEKADD GKVNAVQLLL RLWNEGAPSS ETHDGSQRVE SGCDVSSVAT

**1501** TVGREPRLRK RDFDYLKIST TSTTLTKTYD VCDEDIDDAS AGRPAVATEL

**1551** RPSSWGLNSL GLQGRELVTA ALWRRYADLE RMDRFNTEAS SSGELASPRG

**1601** FRELLSAPIC KVLGLRVGVY ACLAALGFER ISEVKASPSE RVKLLHIAAL

**1651** EALCKDEVWG AIAESVDLAG RVVIRAEKRP CTSTMGSFYG GKHRNSVSFR

**1701** NSTLLVPIIP DADK**LVSVLA DVDTRAR**EMK RFEAVTLDMQ GVHNFEDLHR

**1751** SLLAFVRHDD KVPACTIAEQ FRNGCKKASA VRLISPPLTV HTGSEGAEAM

**1801** NDLVTIDQYP TLSGNNAPTT PMGSWRPYLT MPTGFSEKKR KKEAMIKNSF

**1851** KSSKAQCKKS MTSTCHM

**Start - End Observed Mr(expt) Mr(calc) Delta Miss Sequence**

**283 - 299 1930.94 1929.94 1929.93 0.01 0 K.GSAPQEYPHTTSASPTR.E**  ([Ions score 56](../../../../D:%5CProteomic%20data%5C2010-1-8%5CZQ%5C1219.htm))

**563 - 568 920.51 919.50 919.41 0.09 0 R.ENDNER.V**  ([Ions score 7](../../../../D:%5CProteomic%20data%5C2010-1-8%5CZQ%5C1216.htm))

**823 - 835 1597.84 1596.83 1596.79 0.04 0 K.QSEYATTNSPLSR.L**  ([Ions score 2](../../../../D:%5CProteomic%20data%5C2010-1-8%5CZQ%5C1218.htm))

**1715 - 1727 1558.85 1557.84 1557.90 -0.05 1 K.LVSVLADVDTRAR.E**  ([Ions score 7](../../../../D:%5CProteomic%20data%5C2010-1-8%5CZQ%5C1217.htm))

MS/MS Fragmentation of **GSAPQEYPHTTSASPTR**
Found in **Tb10.70.4610**, hypothetical protein, conserved; Trypanosoma bruceichr 10Manual


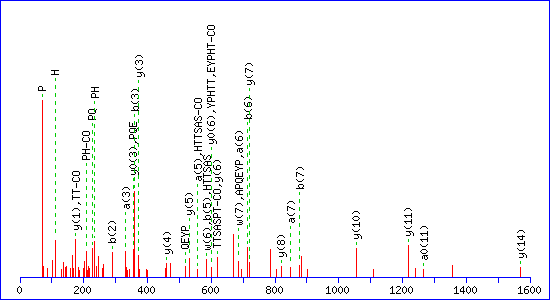


**MONOISOTOPIC mass of neutral peptide Mr(calc):** 1929.93

**Fixed modifications:** MMTS (C),(N-TERM)_iTRAQ,Lysine(K)_iTRAQ

**Ions Score:** 56 **Expect:** 0.00028

**Matches (Bold Red):** 41/301 fragment ions using 43 most intense peaks

| **#** | **Immon.** | **a** | **a*** | **a^0^** | **b** | **b*** | **b^0^** | **Seq.** | **v** | **w** | **w'** | **y** | **y*** | **y^0^** | **#** |
| --- | --- | --- | --- | --- | --- | --- | --- | --- | --- | --- | --- | --- | --- | --- | --- |
| **1** | 30.03 | 174.14 |  |  | 202.13 |  |  | **G** |  |  |  |  |  |  | **17** |
| **2** | 60.04 | 261.17 |  | 243.16 | **289.16** |  | 271.15 | **S** | 1697.79 | 1696.79 |  | 1729.81 | 1712.79 | 1711.80 | **16** |
| **3** | 44.05 | **332.21** |  | 314.19 | **360.20** |  | 342.19 | **A** | 1626.75 |  |  | 1642.78 | 1625.76 | 1624.77 | **15** |
| **4** | **70.07** | 429.26 |  | 411.25 | 457.25 |  | 439.24 | **P** | 1529.70 | 1528.70 |  | **1571.74** | 1554.72 | 1553.73 | **14** |
| **5** | 101.07 | **557.32** | 540.29 | 539.31 | **585.31** | 568.29 | 567.30 | **Q** | 1401.64 | 1400.64 |  | 1474.69 | 1457.67 | 1456.68 | **13** |
| **6** | 102.05 | **686.36** | 669.33 | 668.35 | **714.35** | 697.33 | 696.34 | **E** | 1272.60 | 1271.60 |  | 1346.63 | 1329.61 | 1328.62 | **12** |
| **7** | 136.08 | **849.42** | 832.40 | 831.41 | **877.42** | 860.39 | 859.41 | **Y** | 1109.53 |  |  | **1217.59** | 1200.56 | 1199.58 | **11** |
| **8** | **70.07** | 946.48 | 929.45 | 928.46 | 974.47 | 957.44 | 956.46 | **P** | 1012.48 | 1011.49 |  | **1054.53** | 1037.50 | 1036.52 | **10** |
| **9** | **110.07** | 1083.53 | 1066.51 | 1065.52 | 1111.53 | 1094.50 | 1093.52 | **H** | 875.42 |  |  | 957.47 | 940.45 | 939.46 | **9** |
| **10** | 74.06 | 1184.58 | 1167.56 | 1166.57 | 1212.58 | 1195.55 | 1194.57 | **T** | 774.37 | 787.39 | 789.37 | **820.42** | 803.39 | 802.41 | **8** |
| **11** | 74.06 | 1285.63 | 1268.60 | **1267.62** | 1313.62 | 1296.60 | 1295.61 | **T** | 673.33 | **686.35** | 688.33 | **719.37** | 702.34 | 701.36 | **7** |
| **12** | 60.04 | 1372.66 | 1355.64 | 1354.65 | 1400.66 | 1383.63 | 1382.65 | **S** | 586.29 | **585.30** |  | **618.32** | 601.29 | **600.31** | **6** |
| **13** | 44.05 | 1443.70 | 1426.67 | 1425.69 | 1471.69 | 1454.67 | 1453.68 | **A** | 515.26 |  |  | **531.29** | 514.26 | 513.28 | **5** |
| **14** | 60.04 | 1530.73 | 1513.70 | 1512.72 | 1558.73 | 1541.70 | 1540.72 | **S** | 428.23 | 427.23 |  | **460.25** | 443.22 | 442.24 | **4** |
| **15** | **70.07** | 1627.78 | 1610.76 | 1609.77 | 1655.78 | 1638.75 | 1637.77 | **P** | 331.17 | 330.18 |  | **373.22** | 356.19 | **355.21** | **3** |
| **16** | 74.06 | 1728.83 | 1711.80 | 1710.82 | 1756.83 | 1739.80 | 1738.82 | **T** | 230.12 | 243.15 | 245.12 | 276.17 | 259.14 | 258.16 | **2** |
| **17** | 129.11 |  |  |  |  |  |  | **R** | 74.02 | 73.03 |  | **175.12** | 158.09 |  | **1** |

| **Seq** | **ya** | **yb** | **Seq** | **ya** | **yb** | **Seq** | **ya** | **yb** |
| --- | --- | --- | --- | --- | --- | --- | --- | --- |
| **SA** | 131.08 | 159.08 | **SAP** | 228.13 | 256.13 | **SAPQ** | 356.19 | 384.19 |
| **SAPQE** | 485.24 | 513.23 | **SAPQEY** | 648.30 | 676.29 | **AP** | 141.10 | 169.10 |
| **APQ** | 269.16 | 297.16 | **APQE** | 398.20 | 426.20 | **APQEY** | 561.27 | 589.26 |
| **APQEYP** | 658.32 | **686.31** | **PQ** | 198.12 | **226.12** | **PQE** | 327.17 | **355.16** |
| **PQEY** | 490.23 | **518.22** | **PQEYP** | 587.28 | 615.28 | **QE** | 230.11 | 258.11 |
| **QEY** | 393.18 | 421.17 | **QEYP** | 490.23 | **518.22** | **QEYPH** | 627.29 | 655.28 |
| **EY** | 265.12 | 293.11 | **EYP** | 362.17 | 390.17 | **EYPH** | 499.23 | 527.22 |
| **EYPHT** | **600.28** | 628.27 | **YP** | 233.13 | 261.12 | **YPH** | 370.19 | 398.18 |
| **YPHT** | 471.24 | 499.23 | **YPHTT** | 572.28 | **600.28** | **YPHTTS** | 659.31 | 687.31 |
| **PH** | **207.12** | **235.12** | **PHT** | 308.17 | 336.17 | **PHTT** | 409.22 | 437.21 |
| **PHTTS** | 496.25 | 524.25 | **PHTTSA** | 567.29 | 595.28 | **PHTTSAS** | 654.32 | 682.32 |
| **HT** | 211.12 | 239.11 | **HTT** | 312.17 | 340.16 | **HTTS** | 399.20 | 427.19 |
| **HTTSA** | 470.24 | 498.23 | **HTTSAS** | **557.27** | **585.26** | **HTTSASP** | 654.32 | 682.32 |
| **TT** | **175.11** | 203.10 | **TTS** | 262.14 | 290.13 | **TTSA** | 333.18 | 361.17 |
| **TTSAS** | 420.21 | 448.20 | **TTSASP** | 517.26 | 545.26 | **TTSASPT** | **618.31** | 646.30 |
| **TS** | 161.09 | 189.09 | **TSA** | 232.13 | 260.12 | **TSAS** | 319.16 | 347.16 |
| **TSASP** | 416.21 | 444.21 | **TSASPT** | 517.26 | 545.26 | **SA** | 131.08 | 159.08 |
| **SAS** | 218.11 | 246.11 | **SASP** | 315.17 | 343.16 | **SASPT** | 416.21 | 444.21 |
| **AS** | 131.08 | 159.08 | **ASP** | 228.13 | 256.13 | **ASPT** | 329.18 | 357.18 |
| **SP** | 157.10 | 185.09 | **SPT** | 258.14 | 286.14 | **PT** | 171.11 | 199.11 |

40. Tb10.6k15.3080

Match to: **Tb10.6k15.3080** Score: **53**

**dihydrolipoamide acetyltransferase E2 subunit, putative; Trypanosoma bruceichr 10Manual**

Nominal mass (M_r_): **52529**; Calculated pI value: **7.01**

NCBI BLAST search of [Tb10.6k15.3080](http://www.ncbi.nlm.nih.gov/blast/Blast.cgi?ALIGNMENTS=50&ALIGNMENT_VIEW=Pairwise&AUTO_FORMAT=Semiauto&CDD_SEARCH=on&CLIENT=web&COMPOSITION_BASED_STATISTICS=on&DATABASE=nr&DESCRIPTIONS=100&ENTREZ_QUERY=(none)&EXPECT=10&FILTER=L&FORMAT_BLOCK_ON_RESPAGE=None&FORMAT_OBJECT=Alignment&FORMAT_TYPE=HTML&GAPCOSTS=11+1&I_THRESH=0.001&LAYOUT=TwoWindows&MATRIX_NAME=BLOSUM62&NCBI_GI=on&PAGE=Proteins&PROGRAM=blastp&QUERY=MFRRPVAQHIFPVCFARFLTVTPIPMPALSPTMEKGKISEWVKKVGDAVETGDTWCKVETDKAVVSYDNVSEDGFVARILVQVGEEATVGDAVCLIVDEASGVNSDEVKNWQAAGSSPAATQSKVQEVPSPTQVAPLPAGGKEAGGRVKASPLARKTAAELNVSLDTIEGTGGGVGRIVRKDVEAAASKREHAAPAAAPAAKPVVPVIATTPSTQNYTDIPVTNMRSTIAKRLTQSKNVEIPHYYLFEECCAENMMALVQQLNSKGDGKYKITLNDYIIKAVARANMLVPEANSSWQGDFIRQYRTVDVSVAVATPTGLITPIIKDAQARGLVDISNEMKVLAKKAREGTLQPHEFIGGTVSVSNLGASGIPGFTAIINPPQALIVAVGSAKPRPRMSLDPDTGKYTVGAEAEMFVRFTASFDHRVVDGAVASQWCKHFKDAVENPLSLLL&SERVICE=plain&SET_DEFAULTS.x=9&SET_DEFAULTS.y=5&SHOW_OVERVIEW=on&WORD_SIZE=3&END_OF_HTTPGET=Yes) against nr

Unformatted [sequence string](../../../../D:%5CProteomic%20data%5C2010-1-8%5Ccgi%5Cgetseq.pl%3FTBA927_IPI+Tb10%2E6k15%2E3080+seq) for pasting into other applications

Fixed modifications: MMTS (C),(N-TERM)_iTRAQ,Lysine(K)_iTRAQ

Variable modifications: Oxidation (M)

Cleavage by Trypsin: cuts C-term side of KR unless next residue is P

Sequence Coverage: **3%**

Matched peptides shown in **Bold Red**

**1** MFRRPVAQHI FPVCFARFLT VTPIPMPALS PTMEKGKISE WVKKVGDAVE

**51** TGDTWCKVET DKAVVSYDNV SEDGFVARIL VQVGEEATVG DAVCLIVDEA

**101** SGVNSDEVK**N WQAAGSSPAA TQSK**VQEVPS PTQVAPLPAG GKEAGGRVKA

**151** SPLARKTAAE LNVSLDTIEG TGGGVGRIVR KDVEAAASKR EHAAPAAAPA

**201** AKPVVPVIAT TPSTQNYTDI PVTNMRSTIA KRLTQSKNVE IPHYYLFEEC

**251** CAENMMALVQ QLNSKGDGKY KITLNDYIIK AVARANMLVP EANSSWQGDF

**301** IRQYRTVDVS VAVATPTGLI TPIIKDAQAR GLVDISNEMK VLAKKAREGT

**351** LQPHEFIGGT VSVSNLGASG IPGFTAIINP PQALIVAVGS AKPRPRMSLD

**401** PDTGKYTVGA EAEMFVRFTA SFDHRVVDGA VASQWCKHFK DAVENPLSLL

**451** L

MS/MS Fragmentation of **NWQAAGSSPAATQSK**
Found in **Tb10.6k15.3080**, dihydrolipoamide acetyltransferase E2 subunit, putative; Trypanosoma bruceichr 10Manual


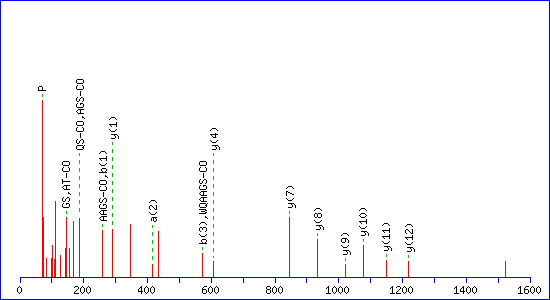


**MONOISOTOPIC mass of neutral peptide Mr(calc):** 1790.83

**Fixed modifications:** MMTS (C),(N-TERM)_iTRAQ,Lysine(K)_iTRAQ

**Ions Score:** 53 **Expect:** 0.00054

**Matches (Bold Red):** 18/250 fragment ions using 20 most intense peaks

| **#** | **Immon.** | **a** | **a*** | **a^0^** | **b** | **b*** | **b^0^** | **Seq.** | **y** | **y*** | **y^0^** | **#** |
| --- | --- | --- | --- | --- | --- | --- | --- | --- | --- | --- | --- | --- |
| **1** | 87.06 | 231.16 | 214.13 |  | **259.15** | 242.13 |  | **N** |  |  |  | **15** |
| **2** | 159.09 | **417.24** | 400.21 |  | 445.23 | 428.21 |  | **W** | 1533.69 | 1516.66 | 1515.68 | **14** |
| **3** | 101.07 | 545.30 | 528.27 |  | **573.29** | 556.26 |  | **Q** | 1347.61 | 1330.58 | 1329.60 | **13** |
| **4** | 44.05 | 616.33 | 599.31 |  | 644.33 | 627.30 |  | **A** | **1219.55** | 1202.52 | 1201.54 | **12** |
| **5** | 44.05 | 687.37 | 670.34 |  | 715.36 | 698.34 |  | **A** | **1148.51** | 1131.49 | 1130.50 | **11** |
| **6** | 30.03 | 744.39 | 727.36 |  | 772.39 | 755.36 |  | **G** | **1077.48** | 1060.45 | 1059.47 | **10** |
| **7** | 60.04 | 831.42 | 814.40 | 813.41 | 859.42 | 842.39 | 841.41 | **S** | **1020.45** | 1003.43 | 1002.44 | **9** |
| **8** | 60.04 | 918.46 | 901.43 | 900.44 | 946.45 | 929.42 | 928.44 | **S** | **933.42** | 916.40 | 915.41 | **8** |
| **9** | **70.07** | 1015.51 | 998.48 | 997.50 | 1043.50 | 1026.48 | 1025.49 | **P** | **846.39** | 829.36 | 828.38 | **7** |
| **10** | 44.05 | 1086.55 | 1069.52 | 1068.53 | 1114.54 | 1097.51 | 1096.53 | **A** | 749.34 | 732.31 | 731.33 | **6** |
| **11** | 44.05 | 1157.58 | 1140.56 | 1139.57 | 1185.58 | 1168.55 | 1167.57 | **A** | 678.30 | 661.27 | 660.29 | **5** |
| **12** | 74.06 | 1258.63 | 1241.60 | 1240.62 | 1286.62 | 1269.60 | 1268.61 | **T** | **607.26** | 590.24 | 589.25 | **4** |
| **13** | 101.07 | 1386.69 | 1369.66 | 1368.68 | 1414.68 | 1397.66 | 1396.67 | **Q** | 506.22 | 489.19 | 488.21 | **3** |
| **14** | 60.04 | 1473.72 | 1456.69 | 1455.71 | 1501.72 | 1484.69 | 1483.70 | **S** | 378.16 | 361.13 | 360.15 | **2** |
| **15** | 245.12 |  |  |  |  |  |  | **K** | **291.13** | 274.10 |  | **1** |

| **Seq** | **ya** | **yb** | **Seq** | **ya** | **yb** | **Seq** | **ya** | **yb** |
| --- | --- | --- | --- | --- | --- | --- | --- | --- |
| **WQ** | 287.15 | 315.15 | **WQA** | 358.19 | 386.18 | **WQAA** | 429.22 | 457.22 |
| **WQAAG** | 486.25 | 514.24 | **WQAAGS** | **573.28** | 601.27 | **WQAAGSS** | 660.31 | 688.30 |
| **QA** | 172.11 | 200.10 | **QAA** | 243.15 | 271.14 | **QAAG** | 300.17 | 328.16 |
| **QAAGS** | 387.20 | 415.19 | **QAAGSS** | 474.23 | 502.23 | **QAAGSSP** | 571.28 | 599.28 |
| **QAAGSSPA** | 642.32 | 670.32 | **AA** | 115.09 | 143.08 | **AAG** | 172.11 | 200.10 |
| **AAGS** | **259.14** | 287.13 | **AAGSS** | 346.17 | 374.17 | **AAGSSP** | 443.22 | 471.22 |
| **AAGSSPA** | 514.26 | 542.26 | **AAGSSPAA** | 585.30 | 613.29 | **AAGSSPAAT** | 686.35 | 714.34 |
| **AG** | 101.07 | 129.07 | **AGS** | **188.10** | 216.10 | **AGSS** | 275.13 | 303.13 |
| **AGSSP** | 372.19 | 400.18 | **AGSSPA** | 443.22 | 471.22 | **AGSSPAA** | 514.26 | 542.26 |
| **AGSSPAAT** | 615.31 | 643.30 | **GS** | 117.07 | **145.06** | **GSS** | 204.10 | 232.09 |
| **GSSP** | 301.15 | 329.15 | **GSSPA** | 372.19 | 400.18 | **GSSPAA** | 443.22 | 471.22 |
| **GSSPAAT** | 544.27 | 572.27 | **GSSPAATQ** | 672.33 | 700.33 | **SS** | 147.08 | 175.07 |
| **SSP** | 244.13 | 272.12 | **SSPA** | 315.17 | 343.16 | **SSPAA** | 386.20 | 414.20 |
| **SSPAAT** | 487.25 | 515.25 | **SSPAATQ** | 615.31 | 643.30 | **SP** | 157.10 | 185.09 |
| **SPA** | 228.13 | 256.13 | **SPAA** | 299.17 | 327.17 | **SPAAT** | 400.22 | 428.21 |
| **SPAATQ** | 528.28 | 556.27 | **SPAATQS** | 615.31 | 643.30 | **PA** | 141.10 | 169.10 |
| **PAA** | 212.14 | 240.13 | **PAAT** | 313.19 | 341.18 | **PAATQ** | 441.25 | 469.24 |
| **PAATQS** | 528.28 | 556.27 | **AA** | 115.09 | 143.08 | **AAT** | 216.13 | 244.13 |
| **AATQ** | 344.19 | 372.19 | **AATQS** | 431.22 | 459.22 | **AT** | **145.10** | 173.09 |
| **ATQ** | 273.16 | 301.15 | **ATQS** | 360.19 | 388.18 | **TQ** | 202.12 | 230.11 |
| **TQS** | 289.15 | 317.15 | **QS** | **188.10** | 216.10 |  |  |  |

41. Tb10.70.4990

Match to: **Tb10.70.4990** Score: **53**

**hypothetical protein, conserved; Trypanosoma bruceichr 10Manual**

Nominal mass (M_r_): **14765**; Calculated pI value: **5.29**

NCBI BLAST search of [Tb10.70.4990](http://www.ncbi.nlm.nih.gov/blast/Blast.cgi?ALIGNMENTS=50&ALIGNMENT_VIEW=Pairwise&AUTO_FORMAT=Semiauto&CDD_SEARCH=on&CLIENT=web&COMPOSITION_BASED_STATISTICS=on&DATABASE=nr&DESCRIPTIONS=100&ENTREZ_QUERY=(none)&EXPECT=10&FILTER=L&FORMAT_BLOCK_ON_RESPAGE=None&FORMAT_OBJECT=Alignment&FORMAT_TYPE=HTML&GAPCOSTS=11+1&I_THRESH=0.001&LAYOUT=TwoWindows&MATRIX_NAME=BLOSUM62&NCBI_GI=on&PAGE=Proteins&PROGRAM=blastp&QUERY=MEQKENEITTVYASNNPDIPTLTEAATLILAPEEMVKLRRNMTAEKVAHARYLREHPEIDAIMRYAMRKLIMERSEDPVKVLLEFFSTADLRAALAEENPEAEGRAAMLREKRGLTIVLPA&SERVICE=plain&SET_DEFAULTS.x=9&SET_DEFAULTS.y=5&SHOW_OVERVIEW=on&WORD_SIZE=3&END_OF_HTTPGET=Yes) against nr

Unformatted [sequence string](../../../../D:%5CProteomic%20data%5C2010-1-8%5Ccgi%5Cgetseq.pl%3FTBA927_IPI+Tb10%2E70%2E4990+seq) for pasting into other applications

Fixed modifications: MMTS (C),(N-TERM)_iTRAQ,Lysine(K)_iTRAQ

Variable modifications: Oxidation (M)

Cleavage by Trypsin: cuts C-term side of KR unless next residue is P

Sequence Coverage: **10%**

Matched peptides shown in **Bold Red**

**1** MEQKENEITT VYASNNPDIP TLTEAATLIL APEEMVKLRR NMTAEKVAHA

**51** RYLREHPEID AIMRYAMRKL IMERSEDPVK VLLEFFSTAD LR**AALAEENP**

**101 EAEGR**AAMLR EKRGLTIVLP A

MS/MS Fragmentation of **AALAEENPEAEGR**
Found in **Tb10.70.4990**, hypothetical protein, conserved; Trypanosoma bruceichr 10Manual


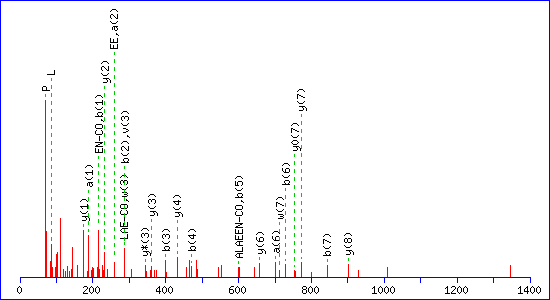


**MONOISOTOPIC mass of neutral peptide Mr(calc):** 1499.73

**Fixed modifications:** MMTS (C),(N-TERM)_iTRAQ,Lysine(K)_iTRAQ

**Ions Score:** 53 **Expect:** 0.00075

**Matches (Bold Red):** 28/200 fragment ions using 36 most intense peaks

| **#** | **Immon.** | **a** | **a*** | **a^0^** | **b** | **b*** | **b^0^** | **Seq.** | **v** | **w** | **y** | **y*** | **y^0^** | **#** |
| --- | --- | --- | --- | --- | --- | --- | --- | --- | --- | --- | --- | --- | --- | --- |
| **1** | 44.05 | **188.15** |  |  | **216.15** |  |  | **A** |  |  |  |  |  | **13** |
| **2** | 44.05 | **259.19** |  |  | **287.18** |  |  | **A** | 1269.57 |  | 1285.60 | 1268.58 | 1267.59 | **12** |
| **3** | **86.10** | 372.27 |  |  | **400.27** |  |  | **L** | 1156.49 | 1155.49 | 1214.56 | 1197.54 | 1196.55 | **11** |
| **4** | 44.05 | 443.31 |  |  | **471.31** |  |  | **A** | 1085.45 |  | 1101.48 | 1084.45 | 1083.47 | **10** |
| **5** | 102.05 | 572.35 |  | 554.34 | **600.35** |  | 582.34 | **E** | 956.41 | 955.41 | 1030.44 | 1013.42 | 1012.43 | **9** |
| **6** | 102.05 | **701.40** |  | 683.38 | **729.39** |  | 711.38 | **E** | 827.36 | 826.37 | **901.40** | 884.37 | 883.39 | **8** |
| **7** | 87.06 | 815.44 | 798.41 | 797.43 | **843.43** | 826.41 | 825.42 | **N** | 713.32 | **712.33** | **772.36** | 755.33 | **754.35** | **7** |
| **8** | **70.07** | 912.49 | 895.46 | 894.48 | 940.49 | 923.46 | 922.48 | **P** | 616.27 | 615.27 | **658.32** | 641.29 | 640.30 | **6** |
| **9** | 102.05 | 1041.53 | 1024.51 | 1023.52 | 1069.53 | 1052.50 | 1051.52 | **E** | 487.23 | 486.23 | 561.26 | 544.24 | 543.25 | **5** |
| **10** | 44.05 | 1112.57 | 1095.54 | 1094.56 | 1140.57 | 1123.54 | 1122.56 | **A** | 416.19 |  | **432.22** | 415.19 | 414.21 | **4** |
| **11** | 102.05 | 1241.61 | 1224.59 | 1223.60 | 1269.61 | 1252.58 | 1251.60 | **E** | **287.15** | **286.15** | **361.18** | **344.16** | 343.17 | **3** |
| **12** | 30.03 | 1298.63 | 1281.61 | 1280.62 | 1326.63 | 1309.60 | 1308.62 | **G** |  |  | **232.14** | 215.11 |  | **2** |
| **13** | 129.11 |  |  |  |  |  |  | **R** | 74.02 | 73.03 | **175.12** | 158.09 |  | **1** |

| **Seq** | **ya** | **yb** | **Seq** | **ya** | **yb** | **Seq** | **ya** | **yb** |
| --- | --- | --- | --- | --- | --- | --- | --- | --- |
| **AL** | 157.13 | 185.13 | **ALA** | 228.17 | 256.17 | **ALAE** | 357.21 | 385.21 |
| **ALAEE** | 486.26 | 514.25 | **ALAEEN** | **600.30** | 628.29 | **ALAEENP** | 697.35 | 725.35 |
| **LA** | 157.13 | 185.13 | **LAE** | **286.18** | 314.17 | **LAEE** | 415.22 | 443.21 |
| **LAEEN** | 529.26 | 557.26 | **LAEENP** | 626.31 | 654.31 | **AE** | 173.09 | 201.09 |
| **AEE** | 302.13 | 330.13 | **AEEN** | 416.18 | 444.17 | **AEENP** | 513.23 | 541.23 |
| **AEENPE** | 642.27 | 670.27 | **EE** | 231.10 | **259.09** | **EEN** | 345.14 | 373.14 |
| **EENP** | 442.19 | 470.19 | **EENPE** | 571.24 | 599.23 | **EENPEA** | 642.27 | 670.27 |
| **EN** | **216.10** | 244.09 | **ENP** | 313.15 | 341.15 | **ENPE** | 442.19 | 470.19 |
| **ENPEA** | 513.23 | 541.23 | **ENPEAE** | 642.27 | 670.27 | **ENPEAEG** | 699.29 | 727.29 |
| **NP** | 184.11 | 212.10 | **NPE** | 313.15 | 341.15 | **NPEA** | 384.19 | 412.18 |
| **NPEAE** | 513.23 | 541.23 | **NPEAEG** | 570.25 | 598.25 | **PE** | 199.11 | 227.10 |
| **PEA** | 270.14 | 298.14 | **PEAE** | 399.19 | 427.18 | **PEAEG** | 456.21 | 484.20 |
| **EA** | 173.09 | 201.09 | **EAE** | 302.13 | 330.13 | **EAEG** | 359.16 | 387.15 |
| **AE** | 173.09 | 201.09 | **AEG** | 230.11 | 258.11 | **EG** | 159.08 | 187.07 |

42. Tb10.70.6570

Match to: **Tb10.70.6570** Score: **52**

**hypothetical protein, conserved; Trypanosoma bruceichr 10Manual**

Nominal mass (M_r_): **354979**; Calculated pI value: **4.58**

NCBI BLAST search of [Tb10.70.6570](http://www.ncbi.nlm.nih.gov/blast/Blast.cgi?ALIGNMENTS=50&ALIGNMENT_VIEW=Pairwise&AUTO_FORMAT=Semiauto&CDD_SEARCH=on&CLIENT=web&COMPOSITION_BASED_STATISTICS=on&DATABASE=nr&DESCRIPTIONS=100&ENTREZ_QUERY=(none)&EXPECT=10&FILTER=L&FORMAT_BLOCK_ON_RESPAGE=None&FORMAT_OBJECT=Alignment&FORMAT_TYPE=HTML&GAPCOSTS=11+1&I_THRESH=0.001&LAYOUT=TwoWindows&MATRIX_NAME=BLOSUM62&NCBI_GI=on&PAGE=Proteins&PROGRAM=blastp&QUERY=Tb10.70.6570&SERVICE=plain&SET_DEFAULTS.x=21&SET_DEFAULTS.y=7&SHOW_OVERVIEW=on&WORD_SIZE=3&END_OF_HTTPGET=Yes) against nr

Unformatted [sequence string](../../../../D:%5CProteomic%20data%5C2010-1-8%5Ccgi%5Cgetseq.pl%3FTBA927_IPI+Tb10%2E70%2E6570+seq) for pasting into other applications

Fixed modifications: MMTS (C),(N-TERM)_iTRAQ,Lysine(K)_iTRAQ

Variable modifications: Oxidation (M)

Cleavage by Trypsin: cuts C-term side of KR unless next residue is P

Sequence Coverage: **0%**

Matched peptides shown in **Bold Red**

**1** MDGPFATMDE ETRKTYMERG RAMMEARRAE ILSHSPEKFE QLRRQGYIIF

**51** KRYLQGEQWM PLQGKLHSPL ITTSEEPSKA HKQNMGSSGS SHLPQDDNIT

**101** AKEYNMPTTK LAEWCAPTDK PLFPESTPAE PEELHHVALL TMCGPSVSCV

**151** DGDDEDFKQL SYNKDVATLH KTMEIQHQQI EQLNAQLRNQ ESEIDVLTNE

**201** LKGVTEANAR QLQKEEELQS LQEALQERDR ELQLRLESVN EQNDSLKTQI

**251** MELHILLELK AKELQKLKDD NNALRGEVVT IQNEGDTIKE SETKCKERIL

**301** QLEAANSELG VQVDVSKKEV EAAHQRIKNL EQELTRERKI NEDSTVAYNK

**351** VKKELECKEK TEQQKQEEIK QRKRAEKAMI RELSELQDRI HTLESTQETE

**401** NNSSTAGIPL NPFAKMVIQG EASQPRVEDD DDAVVFGVGD SVTPSVCVET

**451** GMALRVKELE MCISGLQQQL ESERADAARE VDSLRHRIAE HDELLRQKLE

**501** EYKAEQEAK**F LEELEACR**TG NESDDAILER LNTLQACNDQ LKEELRELEE

**551** RQQVELANVT QEAADRIAEH DELLRQKLEE YKAEQEAKFL EELEACRTGN

**601** ESDDAILERL NALQACNDQL KEELRELEER QQVELANVTQ EAADRIAEHD

**651** ELLRQKLEEY KAEQEAKFLE ELEACRTGNE SDDAILERLN TLQACNDQLK

**701** EELRELEERQ QVELANVTQE AADRIAEHDE LLRQKLEEYK AEQEAKFLEE

**751** LEACRTGNES DDAILERLNA LQACNDQLKE ELRELEERQQ VELANVTQEA

**801** ADRIAEHDEL LRQKLEEYKA EQEAKFLEEL EACRTGNESD DAILERLNTL

**851** QACNDQLKEE LRELEERQQV ELANVTQEAA DRIAEHDELL RQKLEEYKAE

**901** QEAKFLEELE ACRTGNESDD AILERLNTLQ ACNDQLKEEL RELEERQQVE

**951** LANVTQEAAD RIAEHDELLR QKLEEYKAEQ EAKFLEELET CRTGNESDDA

**1001** ILERLNTLQA CNDQLKEELR ELEERQQVEL ANVTQEAADR IAEHDELLRQ

**1051** KLEEYKAEQE AKFLEELEAC RTGNESDDAI LERLNTLQAC NDQLKEELRE

**1101** LEERQQVELA NVTQEAADRI AEHDELLRQK LEEYKAEQEA KFLEELEACR

**1151** TGNESDDAIL ERLNALQACN DQLKEELREL EERQQVELAN VTQEAADRIA

**1201** EHDELLRQKL EEYKAEQEAK FLEELEACRT GNESDDAILE RLNALQACND

**1251** QLKEELRELE ERQQVELANV TQEAADRIAE HDELLRQKLE EYKAEQEAKF

**1301** LEELEACRTG NESDDAILER LNTLQACNDQ LKEELRELEE RQQVELANVT

**1351** QEAADRIAEH DELLRQKLEE YKAEQEAKFL EELETCRTGN ESDDAILERL

**1401** NTLQACNDQL KEELRELEER QQVELANVTQ EAADRIAEHD ELLRQKLEEY

**1451** KAEQEAKFLE ELEACRTGNE SDDAILERLN TLQACNDQLK EELRELEERQ

**1501** QVELANVTQE AADRIAEHDE LLRQKLEEYK AEQEAKFLEE LEACRTGNES

**1551** DDAILERLNA LQACNDQLKE ELRELEERQQ VELANVTQEA ADRIAEHDEL

**1601** LRQKLEEYKA EQEAKFLEEL EACRTGNESD DAILERLNTL QACNDQLKEE

**1651** LRELEERQQV ELANVTQEAA DRIAEHDELL RQKLEEYKAE QEAKFLEELE

**1701** ACRTGNESDD AILERLNTLQ ACNDQLKEEL RELEERQQVE LANVTQEAAD

**1751** RIAEHDELLR QKLEEYKAEQ EAKFLEELEA CRTGNESDDA ILERLNTLQA

**1801** CNDQLKEELR ELEERQQVEL ANVTQEAADR IAEHDELLRQ KLEEYKAEQE

**1851** AKFLEELETC RTGNESDDAI LERLNALQAC NDQLKEELRE LEERQQVELA

**1901** NVTQEAGGTR GYCGVTVFVA STSSSVGSVR SLLGADSCLP CINLETADCG

**1951** AKEAECCISH REVVGNGSHC VDVLKGSEKV SSRVQTDSPE AIGVVCRSFV

**2001** DMTEDRENGE RVDYGNLNGL SYCNFSGDIP DDIGMGNLVI CRKQSKCNKS

**2051** CQVDDGVVPL DDSNEAELRV EDCQTLPDVT RNRVELQREE QSRLHSENGG

**2101** SEKEVHTAEL DHTTLKHNVV EDLFAEKVLT IPLVVEYLFR AVLDAHEYLV

**2151** RHHVNFQRKL LSRCDVIERA ASDAMIEGER QCEEFMAVVE GAEVERQGLL

**2201** RQITELQQEV ATFKASSERV ESELCSNKEE HSKVIRKLHE KLDATQHEME

**2251** VVKVEYANSL HKMELVKTEN ANVLHLFSEL KKTAADVEDE QRREVVRLQE

**2301** ALREKTHTSR ELLELTEQKL AAALEEVDQC KCSREDLEKQ LRSTKSSLDR

**2351** VIPRLERAET ERDSLESQLL DATQEVQLLT QRNRTAEEAS RRQADALTAT

**2401** VEELREANTK LGESCKEQQA QLDSLKAQLA VAETELERLR MRATQQESDA

**2451** QRLNGKLCEL ESSSTLQVEE AAAALRAAEM RIAALEPRKA ALQEEVDHVR

**2501** RKNCELQGCC DELKERLRQA ADTTQNAEQM HNRRVQQLNE QVEALQLEVK

**2551** QLQEAHDGLV ADREQLVREQ VGAAQETERI KQNLERCNQQ KERLQNEIQW

**2601** QRESYESEIQ AAQETLNGAR EELSKWRHTL EEAEAQQNAM RDTISMLQVE

**2651** RTSNHEQIRA VRLQLREYQE LLAQERGYLH ETGLKFLRSC SDECSVQKSP

**2701** SVPYTPQREC GTRETCSLTS APAGCCDSQK TQGSASQKSI ARLRNIVAQK

**2751** SLLLEEREAQ VESLYSEMKT AIVHVVQEAR NAQLGSKALA PVDQRELLLN

**2801** NIMHNLLLRL GRLSRVVREP STDSTGSGSR DPANAEVSPN SSTNPEAVLN

**2851** FSSAATPKER VSGGGDESTD AR

MS/MS Fragmentation of **FLEELEACR**
Found in **Tb10.70.6570**, hypothetical protein, conserved; Trypanosoma bruceichr 10Manual


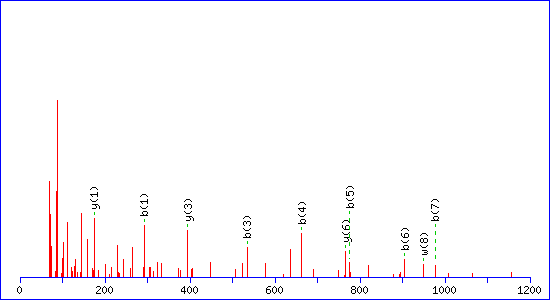


**MONOISOTOPIC mass of neutral peptide Mr(calc):** 1298.61

**Fixed modifications:** MMTS (C),(N-TERM)_iTRAQ,Lysine(K)_iTRAQ

**Ions Score:** 52 **Expect:** 0.00081

**Matches (Bold Red):** 10/112 fragment ions using 13 most intense peaks

| **#** | **Immon.** | **a** | **a^0^** | **b** | **b^0^** | **Seq.** | **v** | **w** | **y** | **y*** | **y^0^** | **#** |
| --- | --- | --- | --- | --- | --- | --- | --- | --- | --- | --- | --- | --- |
| **1** | 120.08 | 264.18 |  | **292.18** |  | **F** |  |  |  |  |  | **9** |
| **2** | 86.10 | 377.27 |  | 405.26 |  | **L** | 950.37 | **949.38** | 1008.45 | 991.42 | 990.44 | **8** |
| **3** | 102.05 | 506.31 | 488.30 | **534.30** | 516.29 | **E** | 821.33 | 820.33 | 895.36 | 878.34 | 877.35 | **7** |
| **4** | 102.05 | 635.35 | 617.34 | **663.35** | 645.34 | **E** | 692.29 | 691.29 | **766.32** | 749.30 | 748.31 | **6** |
| **5** | 86.10 | 748.44 | 730.43 | **776.43** | 758.42 | **L** | 579.20 | 578.21 | 637.28 | 620.25 | 619.27 | **5** |
| **6** | 102.05 | 877.48 | 859.47 | **905.47** | 887.46 | **E** | 450.16 | 449.16 | 524.20 | 507.17 | 506.18 | **4** |
| **7** | 44.05 | 948.52 | 930.51 | **976.51** | 958.50 | **A** | 379.12 |  | **395.15** | 378.13 |  | **3** |
| **8** | 122.01 | 1097.51 | 1079.50 | 1125.51 | 1107.50 | **C** | 230.12 | 229.13 | 324.12 | 307.09 |  | **2** |
| **9** | 129.11 |  |  |  |  | **R** | 74.02 | 73.03 | **175.12** | 158.09 |  | **1** |

| **Seq** | **ya** | **yb** | **Seq** | **ya** | **yb** | **Seq** | **ya** | **yb** |
| --- | --- | --- | --- | --- | --- | --- | --- | --- |
| **LE** | 215.14 | 243.13 | **LEE** | 344.18 | 372.18 | **LEEL** | 457.27 | 485.26 |
| **LEELE** | 586.31 | 614.30 | **LEELEA** | 657.35 | 685.34 | **EE** | 231.10 | 259.09 |
| **EEL** | 344.18 | 372.18 | **EELE** | 473.22 | 501.22 | **EELEA** | 544.26 | 572.26 |
| **EELEAC** | 693.26 | 721.25 | **EL** | 215.14 | 243.13 | **ELE** | 344.18 | 372.18 |
| **ELEA** | 415.22 | 443.21 | **ELEAC** | 564.22 | 592.21 | **LE** | 215.14 | 243.13 |
| **LEA** | 286.18 | 314.17 | **LEAC** | 435.17 | 463.17 | **EA** | 173.09 | 201.09 |
| **EAC** | 322.09 | 350.08 | **AC** | 193.05 | 221.04 |  |  |  |

43. Tb927.3.1670

Match to: **Tb927.3.1670** Score: **49**

**hypothetical protein, conserved; Trypanosoma bruceichr 3Manual**

Nominal mass (M_r_): **119086**; Calculated pI value: **5.38**

NCBI BLAST search of [Tb927.3.1670](http://www.ncbi.nlm.nih.gov/blast/Blast.cgi?ALIGNMENTS=50&ALIGNMENT_VIEW=Pairwise&AUTO_FORMAT=Semiauto&CDD_SEARCH=on&CLIENT=web&COMPOSITION_BASED_STATISTICS=on&DATABASE=nr&DESCRIPTIONS=100&ENTREZ_QUERY=(none)&EXPECT=10&FILTER=L&FORMAT_BLOCK_ON_RESPAGE=None&FORMAT_OBJECT=Alignment&FORMAT_TYPE=HTML&GAPCOSTS=11+1&I_THRESH=0.001&LAYOUT=TwoWindows&MATRIX_NAME=BLOSUM62&NCBI_GI=on&PAGE=Proteins&PROGRAM=blastp&QUERY=MAYSTRVIRKVEPSSTHESLALEWAFGLHNDYKANIHNLSTSTTERVVFYTVGHVGVIYDAIQNTQKHLMGHRHMIVASACSRNRRFIVTADSGSTKSSDCQDACGRSASGNHSTAGGAGEKSGVERYNNESAVIAPEGIYSSNEGRDATMIIWDVQTAIPIRKINTGEYGGVVACAMSLDGMYIATLNRTVPQEIMVWGWTADADTGAEVMRNGADEDPLLHDSMAPEYRHLIAAQDEQISIRFSDDDPHLIVTNGQYRVLFWSWAEGKLKYYSPPIIAKNFKVPIGHFTQTVFVPGTTMACSGTVDGDVLLWEVQQRDRVTKEQDKTMLKMVRVHSSGVSFLTWSNGYIVTGGIDGDVKFLDPRLRLVAWFEDLKGGAITSISFDRPSGTAATAVNELRREFKSITQKKMVQVGTNAVGDFSASDFMVSTSNAMIIDVSANAFHAGVPELLRGRLVVQGQENGVHCIAAHPKLSRLAVAGHSGGLQVWDYLLKRVVMIVVFRGVEINCMAFDPEGVWLAIGCTNGVVKFLDSANLEERKSIKPKRPSSITRMVFASSGRLLATGDDTGCVSLFWYEHIQGNTSKAMGWDVVGRHKTHKGTITGLQFGDDSGLHRLLSVGEDQRLVEYDLIDSEPETGLLVRSAHKIAQSSTPTGFLWMDEDGIISDVSRRPDAAHTITNGLLIANSGYKISAYFSDWSRQCVKTVLAPTFGGPVTEMFTVPTHPGSDKSSLFYATKEKVIGFIQLPLEGDPCLSMGLLAHAGPITSVAKSYDGAYVFTAGGLDQSVMQWRVNGNKIVPEEASKISASVAAEGNGEVPLDHLIAVVEGGREGEFMREIVDYFYYAQIRLQGEETTAKRELLGAVPFSQVPNLFRALGYYPTEMELGRLTYEVANLYGPVEESVDECDVSSIPLKFSQFMRLYVNYRPIFGISRQAVEQAFLVLGADALTGQISRDVLFKKLTTHGEPLQQTEITAALRSLLGEDVKLDDIQDTITARLFAENLLGFEDYDAMAQGDDGGEEEMSLQ&SERVICE=plain&SET_DEFAULTS.x=9&SET_DEFAULTS.y=5&SHOW_OVERVIEW=on&WORD_SIZE=3&END_OF_HTTPGET=Yes) against nr

Unformatted [sequence string](../../../../D:%5CProteomic%20data%5C2010-1-8%5Ccgi%5Cgetseq.pl%3FTBA927_IPI+Tb927%2E3%2E1670+seq) for pasting into other applications

Fixed modifications: MMTS (C),(N-TERM)_iTRAQ,Lysine(K)_iTRAQ

Variable modifications: Oxidation (M)

Cleavage by Trypsin: cuts C-term side of KR unless next residue is P

Sequence Coverage: **6%**

Matched peptides shown in **Bold Red**

**1** MAYSTRVIRK VEPSSTHESL ALEWAFGLHN DYKANIHNLS TSTTERVVFY

**51** TVGHVGVIYD AIQNTQKHLM GHR**HMIVASA CSR**NRRFIVT ADSGSTKSSD

**101** CQDACGRSAS GNHSTAGGAG EKSGVERYNN ESAVIAPEGI YSSNEGRDAT

**151** MIIWDVQTAI PIRKINTGEY GGVVACAMSL DGMYIATLNR TVPQEIMVWG

**201** WTADADTGAE VMRNGADEDP LLHDSMAPEY RHLIAAQDEQ ISIRFSDDDP

**251** HLIVTNGQYR VLFWSWAEGK LKYYSPPIIA KNFKVPIGHF TQTVFVPGTT

**301** MACSGTVDGD VLLWEVQQRD RVTKEQDKTM LKMVRVHSSG VSFLTWSNGY

**351** IVTGGIDGDV KFLDPRLRLV AWFEDLKGGA ITSISFDRPS GTAATAVNEL

**401** RREFKSITQK KMVQVGTNAV GDFSASDFMV STSNAMIIDV SANAFHAGVP

**451** ELLRGRLVVQ GQENGVHCIA AHPKLSRLAV AGHSGGLQVW DYLLKRVVMI

**501** VVFRGVEINC MAFDPEGVWL AIGCTNGVVK FLDSANLEER KSIKPKRPSS

**551** ITRMVFASSG RLLATGDDTG CVSLFWYEHI QGNTSKAMGW DVVGRHKTHK

**601** **GTITGLQFGD DSGLHR**LLSV GEDQRLVEYD LIDSEPETGL LVRSAHKIAQ

**651** SSTPTGFLWM DEDGIISDVS RRPDAAHTIT NGLLIANSGY KISAYFSDWS

**701** RQCVKTVLAP TFGGPVTEMF TVPTHPGSDK SSLFYATKEK VIGFIQLPLE

**751** GDPCLSMGLL AHAGPITSVA KSYDGAYVFT AGGLDQSVMQ WRVNGNKIVP

**801** EEASKISASV AAEGNGEVPL DHLIAVVEGG REGEFMREIV DYFYYAQIRL

**851** QGEETTAKR**E LLGAVPFSQV PNLFR**ALGYY PTEMELGRLT YEVANLYGPV

**901** EESVDECDVS SIPLKFSQFM RLYVNYRPIF GISR**QAVEQA FLVLGADALT**

**951 GQISR**DVLFK KLTTHGEPLQ QTEITAALRS LLGEDVKLDD IQDTITARLF

**1001** AENLLGFEDY DAMAQGDDGG EEEMSLQ

**Start - End Observed Mr(expt) Mr(calc) Delta Miss Sequence**

**74 - 83 1264.62 1263.62 1263.60 0.01 0 R.HMIVASACSR.N**  ([Ions score 1](../../../../D:%5CProteomic%20data%5C2010-1-8%5CZQ%5C1256.htm))

**601 - 616 1817.92 1816.91 1816.92 -0.01 0 K.GTITGLQFGDDSGLHR.L**  ([Ions score 18](../../../../D:%5CProteomic%20data%5C2010-1-8%5CZQ%5C1257.htm))

**860 - 875 1931.07 1930.06 1930.08 -0.02 0 R.ELLGAVPFSQVPNLFR.A**  ([Ions score 49](../../../../D:%5CProteomic%20data%5C2010-1-8%5CZQ%5C1258.htm))

**935 - 955 2331.27 2330.26 2330.27 -0.01 0 R.QAVEQAFLVLGADALTGQISR.D**  ([Ions score 7](../../../../D:%5CProteomic%20data%5C2010-1-8%5CZQ%5C1259.htm))

MS/MS Fragmentation of **ELLGAVPFSQVPNLFR**
Found in **Tb927.3.1670**, hypothetical protein, conserved; Trypanosoma bruceichr 3Manual


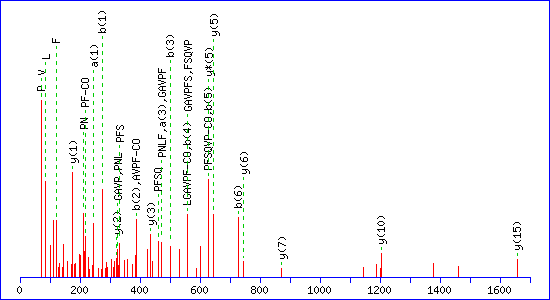


**MONOISOTOPIC mass of neutral peptide Mr(calc):** 1930.08

**Fixed modifications:** MMTS (C),(N-TERM)_iTRAQ,Lysine(K)_iTRAQ

**Ions Score:** 49 **Expect:** 0.0012

**Matches (Bold Red):** 42/267 fragment ions using 36 most intense peaks

| **#** | **Immon.** | **a** | **a*** | **a^0^** | **b** | **b*** | **b^0^** | **Seq.** | **v** | **w** | **y** | **y*** | **y^0^** | **#** |
| --- | --- | --- | --- | --- | --- | --- | --- | --- | --- | --- | --- | --- | --- | --- |
| **1** | 102.05 | **246.16** |  | 228.15 | **274.15** |  | 256.14 | **E** |  |  |  |  |  | **16** |
| **2** | **86.10** | 359.24 |  | 341.23 | **387.24** |  | 369.23 | **L** | 1599.86 | 1598.87 | **1657.94** | 1640.92 | 1639.93 | **15** |
| **3** | **86.10** | **472.33** |  | 454.31 | **500.32** |  | 482.31 | **L** | 1486.78 | 1485.78 | 1544.86 | 1527.83 | 1526.85 | **14** |
| **4** | 30.03 | 529.35 |  | 511.34 | **557.34** |  | 539.33 | **G** |  |  | 1431.77 | 1414.75 | 1413.76 | **13** |
| **5** | 44.05 | 600.38 |  | 582.37 | **628.38** |  | 610.37 | **A** | 1358.72 |  | 1374.75 | 1357.73 | 1356.74 | **12** |
| **6** | **72.08** | 699.45 |  | 681.44 | **727.45** |  | 709.44 | **V** | 1259.65 | 1272.67 | 1303.72 | 1286.69 | 1285.71 | **11** |
| **7** | **70.07** | 796.51 |  | 778.49 | 824.50 |  | 806.49 | **P** | 1162.60 | 1161.61 | **1204.65** | 1187.62 | 1186.64 | **10** |
| **8** | **120.08** | 943.57 |  | 925.56 | 971.57 |  | 953.56 | **F** | 1015.53 |  | 1107.59 | 1090.57 | 1089.58 | **9** |
| **9** | 60.04 | 1030.61 |  | 1012.60 | 1058.60 |  | 1040.59 | **S** | 928.50 | 927.50 | 960.53 | 943.50 | 942.52 | **8** |
| **10** | 101.07 | 1158.66 | 1141.64 | 1140.65 | 1186.66 | 1169.63 | 1168.65 | **Q** | 800.44 | 799.45 | **873.49** | 856.47 |  | **7** |
| **11** | **72.08** | 1257.73 | 1240.71 | 1239.72 | 1285.73 | 1268.70 | 1267.72 | **V** | 701.37 | 714.39 | **745.44** | 728.41 |  | **6** |
| **12** | **70.07** | 1354.79 | 1337.76 | 1336.77 | 1382.78 | 1365.75 | 1364.77 | **P** | 604.32 | 603.32 | **646.37** | **629.34** |  | **5** |
| **13** | 87.06 | 1468.83 | 1451.80 | 1450.82 | 1496.82 | 1479.80 | 1478.81 | **N** | 490.28 | 489.28 | 549.31 | 532.29 |  | **4** |
| **14** | **86.10** | 1581.91 | 1564.89 | 1563.90 | 1609.91 | 1592.88 | 1591.90 | **L** | 377.19 | 376.20 | **435.27** | 418.24 |  | **3** |
| **15** | **120.08** | 1728.98 | 1711.95 | 1710.97 | 1756.98 | 1739.95 | 1738.97 | **F** | 230.12 |  | **322.19** | 305.16 |  | **2** |
| **16** | 129.11 |  |  |  |  |  |  | **R** | 74.02 | 73.03 | **175.12** | 158.09 |  | **1** |

| **Seq** | **ya** | **yb** | **Seq** | **ya** | **yb** | **Seq** | **ya** | **yb** |
| --- | --- | --- | --- | --- | --- | --- | --- | --- |
| **LL** | 199.18 | 227.18 | **LLG** | 256.20 | 284.20 | **LLGA** | 327.24 | 355.23 |
| **LLGAV** | 426.31 | 454.30 | **LLGAVP** | 523.36 | 551.36 | **LLGAVPF** | 670.43 | 698.42 |
| **LG** | 143.12 | 171.11 | **LGA** | 214.15 | 242.15 | **LGAV** | 313.22 | 341.22 |
| **LGAVP** | 410.28 | 438.27 | **LGAVPF** | **557.34** | 585.34 | **LGAVPFS** | 644.38 | 672.37 |
| **GA** | 101.07 | 129.07 | **GAV** | 200.14 | 228.13 | **GAVP** | 297.19 | **325.19** |
| **GAVPF** | 444.26 | **472.26** | **GAVPFS** | 531.29 | **559.29** | **GAVPFSQ** | 659.35 | 687.35 |
| **AV** | 143.12 | 171.11 | **AVP** | 240.17 | 268.17 | **AVPF** | **387.24** | 415.23 |
| **AVPFS** | 474.27 | 502.27 | **AVPFSQ** | 602.33 | 630.32 | **VP** | 169.13 | 197.13 |
| **VPF** | 316.20 | 344.20 | **VPFS** | 403.23 | 431.23 | **VPFSQ** | 531.29 | **559.29** |
| **VPFSQV** | 630.36 | 658.36 | **PF** | **217.13** | 245.13 | **PFS** | 304.17 | **332.16** |
| **PFSQ** | 432.22 | **460.22** | **PFSQV** | 531.29 | **559.29** | **PFSQVP** | **628.35** | 656.34 |
| **FS** | 207.11 | 235.11 | **FSQ** | 335.17 | 363.17 | **FSQV** | 434.24 | 462.23 |
| **FSQVP** | 531.29 | **559.29** | **FSQVPN** | 645.34 | 673.33 | **SQ** | 188.10 | 216.10 |
| **SQV** | 287.17 | 315.17 | **SQVP** | 384.22 | 412.22 | **SQVPN** | 498.27 | 526.26 |
| **SQVPNL** | 611.35 | 639.35 | **QV** | 200.14 | 228.13 | **QVP** | 297.19 | **325.19** |
| **QVPN** | 411.24 | 439.23 | **QVPNL** | 524.32 | 552.31 | **QVPNLF** | 671.39 | 699.38 |
| **VP** | 169.13 | 197.13 | **VPN** | 283.18 | 311.17 | **VPNL** | 396.26 | 424.26 |
| **VPNLF** | 543.33 | 571.32 | **PN** | 184.11 | **212.10** | **PNL** | 297.19 | **325.19** |
| **PNLF** | 444.26 | **472.26** | **NL** | 200.14 | 228.13 | **NLF** | 347.21 | 375.20 |
| **LF** | 233.16 | 261.16 |  |  |  |  |  |  |

44. Tb10.61.2090

Match to: **Tb10.61.2090** Score: **49**

**60S ribosomal protein L17, putative; Trypanosoma bruceichr 10Manual**

Nominal mass (M_r_): **22400**; Calculated pI value: **10.99**

NCBI BLAST search of [Tb10.61.2090](http://www.ncbi.nlm.nih.gov/blast/Blast.cgi?ALIGNMENTS=50&ALIGNMENT_VIEW=Pairwise&AUTO_FORMAT=Semiauto&CDD_SEARCH=on&CLIENT=web&COMPOSITION_BASED_STATISTICS=on&DATABASE=nr&DESCRIPTIONS=100&ENTREZ_QUERY=(none)&EXPECT=10&FILTER=L&FORMAT_BLOCK_ON_RESPAGE=None&FORMAT_OBJECT=Alignment&FORMAT_TYPE=HTML&GAPCOSTS=11+1&I_THRESH=0.001&LAYOUT=TwoWindows&MATRIX_NAME=BLOSUM62&NCBI_GI=on&PAGE=Proteins&PROGRAM=blastp&QUERY=MVHYSRKPQVSSKTAKAKIADLRCHYKNTFETANVINGMKLRKAQQLYRQVLAKTRCIPFKRYNGKIGNTAQAKEWGQTKGRWPRKSVVAMLSLLKNAEANAIEKGLDPGKMVIKHVQVDQAPRVRRRTFRAHGRITPYMRSPCHVQLFMTQPQERVPVPKSKPKK&SERVICE=plain&SET_DEFAULTS.x=9&SET_DEFAULTS.y=5&SHOW_OVERVIEW=on&WORD_SIZE=3&END_OF_HTTPGET=Yes) against nr

Unformatted [sequence string](../../../../D:%5CProteomic%20data%5C2010-1-8%5Ccgi%5Cgetseq.pl%3FTBA927_IPI+Tb10%2E61%2E2090+seq) for pasting into other applications

Fixed modifications: MMTS (C),(N-TERM)_iTRAQ,Lysine(K)_iTRAQ

Variable modifications: Oxidation (M)

Cleavage by Trypsin: cuts C-term side of KR unless next residue is P

Sequence Coverage: **5%**

Matched peptides shown in **Bold Red**

**1** MVHYSRKPQV SSKTAKAKIA DLRCHYKNTF ETANVINGMK LRKAQQLYRQ

**51** VLAKTRCIPF KRYNGKIGNT AQAKEWGQTK GRWPRKSVVA MLSLLKNAEA

**101** NAIEKGLDPG KMVIK**HVQVD QAPR**VRRRTF RAHGRITPYM RSPCHVQLFM

**151** TQPQERVPVP KSKPKK

MS/MS Fragmentation of **HVQVDQAPR**
Found in **Tb10.61.2090**, 60S ribosomal protein L17, putative; Trypanosoma bruceichr 10Manual


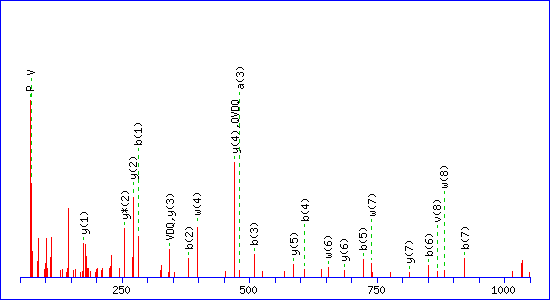


**MONOISOTOPIC mass of neutral peptide Mr(calc):** 1192.64

**Fixed modifications:** MMTS (C),(N-TERM)_iTRAQ,Lysine(K)_iTRAQ

**Ions Score:** 49 **Expect:** 0.0025

**Matches (Bold Red):** 27/120 fragment ions using 29 most intense peaks

| **#** | **Immon.** | **a** | **a*** | **a^0^** | **b** | **b*** | **b^0^** | **Seq.** | **v** | **w** | **y** | **y*** | **y^0^** | **#** |
| --- | --- | --- | --- | --- | --- | --- | --- | --- | --- | --- | --- | --- | --- | --- |
| **1** | 110.07 | 254.17 |  |  | **282.17** |  |  | **H** |  |  |  |  |  | **9** |
| **2** | **72.08** | 353.24 |  |  | **381.24** |  |  | **V** | **868.43** | **881.45** | 912.49 | 895.46 | 894.48 | **8** |
| **3** | 101.07 | **481.30** | 464.27 |  | **509.30** | 492.27 |  | **Q** | 740.37 | **739.37** | **813.42** | 796.39 | 795.41 | **7** |
| **4** | **72.08** | 580.37 | 563.34 |  | **608.36** | 591.34 |  | **V** | 641.30 | **654.32** | **685.36** | 668.34 | 667.35 | **6** |
| **5** | 88.04 | 695.40 | 678.37 | 677.39 | **723.39** | 706.36 | 705.38 | **D** | 526.27 | 525.28 | **586.29** | 569.27 | 568.28 | **5** |
| **6** | 101.07 | 823.45 | 806.43 | 805.44 | **851.45** | 834.42 | 833.44 | **Q** | 398.21 | **397.22** | **471.27** | 454.24 |  | **4** |
| **7** | 44.05 | 894.49 | 877.47 | 876.48 | **922.49** | 905.46 | 904.48 | **A** | 327.18 |  | **343.21** | 326.18 |  | **3** |
| **8** | **70.07** | 991.54 | 974.52 | 973.53 | 1019.54 | 1002.51 | 1001.53 | **P** | 230.12 | 229.13 | **272.17** | **255.15** |  | **2** |
| **9** | 129.11 |  |  |  |  |  |  | **R** | 74.02 | 73.03 | **175.12** | 158.09 |  | **1** |

| **Seq** | **ya** | **yb** | **Seq** | **ya** | **yb** | **Seq** | **ya** | **yb** |
| --- | --- | --- | --- | --- | --- | --- | --- | --- |
| **VQ** | 200.14 | 228.13 | **VQV** | 299.21 | 327.20 | **VQVD** | 414.23 | 442.23 |
| **VQVDQ** | 542.29 | 570.29 | **VQVDQA** | 613.33 | 641.33 | **QV** | 200.14 | 228.13 |
| **QVD** | 315.17 | **343.16** | **QVDQ** | 443.22 | **471.22** | **QVDQA** | 514.26 | 542.26 |
| **QVDQAP** | 611.31 | 639.31 | **VD** | 187.11 | 215.10 | **VDQ** | 315.17 | **343.16** |
| **VDQA** | 386.20 | 414.20 | **VDQAP** | 483.26 | 511.25 | **DQ** | 216.10 | 244.09 |
| **DQA** | 287.13 | 315.13 | **DQAP** | 384.19 | 412.18 | **QA** | 172.11 | 200.10 |
| **QAP** | 269.16 | 297.16 | **AP** | 141.10 | 169.10 |  |  |  |

45. Tb10.389.0910

Match to: **Tb10.389.0910** Score: **48**

**60S ribosomal protein L34, putative; Trypanosoma bruceichr 10Manual**

Nominal mass (M_r_): **23577**; Calculated pI value: **11.99**

NCBI BLAST search of [Tb10.389.0910](http://www.ncbi.nlm.nih.gov/blast/Blast.cgi?ALIGNMENTS=50&ALIGNMENT_VIEW=Pairwise&AUTO_FORMAT=Semiauto&CDD_SEARCH=on&CLIENT=web&COMPOSITION_BASED_STATISTICS=on&DATABASE=nr&DESCRIPTIONS=100&ENTREZ_QUERY=(none)&EXPECT=10&FILTER=L&FORMAT_BLOCK_ON_RESPAGE=None&FORMAT_OBJECT=Alignment&FORMAT_TYPE=HTML&GAPCOSTS=11+1&I_THRESH=0.001&LAYOUT=TwoWindows&MATRIX_NAME=BLOSUM62&NCBI_GI=on&PAGE=Proteins&PROGRAM=blastp&QUERY=MSCPRVQYRRRMHYATRGNRMRLVRTPGNRLVMQKRGKRSQGPHTPWVLGHKRLAGTKALRHTKARLAPRHQKTTSRPYGGVLSHEQVRDRIVRAFLIEEQRIVKRALKAHAKVQKEKKRRAAKRKSKEEKVAAVAKKVAAKVGTKSLVAKKEAPKRKAGKAPVGAKLKK&SERVICE=plain&SET_DEFAULTS.x=9&SET_DEFAULTS.y=5&SHOW_OVERVIEW=on&WORD_SIZE=3&END_OF_HTTPGET=Yes) against nr

Unformatted [sequence string](../../../../D:%5CProteomic%20data%5C2010-1-8%5Ccgi%5Cgetseq.pl%3FTBA927_IPI+Tb10%2E389%2E0910+seq) for pasting into other applications

Fixed modifications: MMTS (C),(N-TERM)_iTRAQ,Lysine(K)_iTRAQ

Variable modifications: Oxidation (M)

Cleavage by Trypsin: cuts C-term side of KR unless next residue is P

Sequence Coverage: **4%**

Matched peptides shown in **Bold Red**

**1** MSCPRVQYRR RMHYATRGNR MRLVRTPGNR LVMQKRGKRS QGPHTPWVLG

**51** HKRLAGTKAL RHTKARLAPR HQKTTSRPYG GVLSHEQVRD RIVR**AFLIEE**

**101 QR**IVKRALKA HAKVQKEKKR RAAKRKSKEE KVAAVAKKVA AKVGTKSLVA

**151** KKEAPKRKAG KAPVGAKLKK

MS/MS Fragmentation of **AFLIEEQR**
Found in **Tb10.389.0910**, 60S ribosomal protein L34, putative; Trypanosoma bruceichr 10Manual


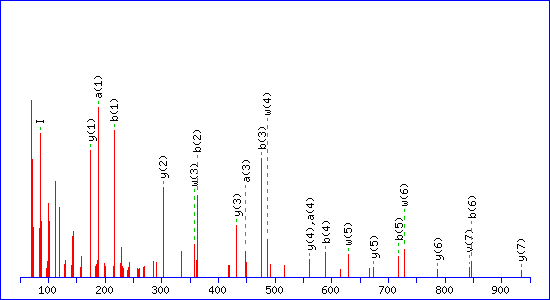


**MONOISOTOPIC mass of neutral peptide Mr(calc):** 1148.63

**Fixed modifications:** MMTS (C),(N-TERM)_iTRAQ,Lysine(K)_iTRAQ

**Ions Score:** 48 **Expect:** 0.0029

**Matches (Bold Red):** 23/91 fragment ions using 25 most intense peaks

| **#** | **Immon.** | **a** | **a*** | **a^0^** | **b** | **b*** | **b^0^** | **Seq.** | **v** | **w** | **w'** | **y** | **y*** | **y^0^** | **#** |
| --- | --- | --- | --- | --- | --- | --- | --- | --- | --- | --- | --- | --- | --- | --- | --- |
| **1** | 44.05 | **188.15** |  |  | **216.15** |  |  | **A** |  |  |  |  |  |  | **8** |
| **2** | 120.08 | 335.22 |  |  | **363.22** |  |  | **F** | **842.44** |  |  | **934.50** | 917.47 | 916.49 | **7** |
| **3** | **86.10** | **448.30** |  |  | **476.30** |  |  | **L** | 729.35 | **728.36** |  | **787.43** | 770.40 | 769.42 | **6** |
| **4** | **86.10** | **561.39** |  |  | **589.38** |  |  | **I** | 616.27 | **629.29** | 643.30 | **674.35** | 657.32 | 656.34 | **5** |
| **5** | 102.05 | 690.43 |  | 672.42 | **718.43** |  | 700.42 | **E** | 487.23 | **486.23** |  | **561.26** | 544.24 | 543.25 | **4** |
| **6** | 102.05 | 819.47 |  | 801.46 | **847.47** |  | 829.46 | **E** | 358.18 | **357.19** |  | **432.22** | 415.19 | 414.21 | **3** |
| **7** | 101.07 | 947.53 | 930.51 | 929.52 | 975.53 | 958.50 | 957.52 | **Q** | 230.12 | 229.13 |  | **303.18** | 286.15 |  | **2** |
| **8** | 129.11 |  |  |  |  |  |  | **R** | 74.02 | 73.03 |  | **175.12** | 158.09 |  | **1** |

| **Seq** | **ya** | **yb** | **Seq** | **ya** | **yb** | **Seq** | **ya** | **yb** |
| --- | --- | --- | --- | --- | --- | --- | --- | --- |
| **FL** | 233.16 | 261.16 | **FLI** | 346.25 | 374.24 | **FLIE** | 475.29 | 503.29 |
| **FLIEE** | 604.33 | 632.33 | **LI** | 199.18 | 227.18 | **LIE** | 328.22 | 356.22 |
| **LIEE** | 457.27 | 485.26 | **LIEEQ** | 585.32 | 613.32 | **IE** | 215.14 | 243.13 |
| **IEE** | 344.18 | 372.18 | **IEEQ** | 472.24 | 500.24 | **EE** | 231.10 | 259.09 |
| **EEQ** | 359.16 | 387.15 | **EQ** | 230.11 | 258.11 |  |  |  |

46. Tb927.6.1400

Match to: **Tb927.6.1400** Score: **48**

**PPIasecyclophilin-type peptidyl-prolyl cis-trans isomerase, putative; Trypanosoma bruceichr 6Manual**

Nominal mass (M_r_): **30385**; Calculated pI value: **5.09**

NCBI BLAST search of [Tb927.6.1400](http://www.ncbi.nlm.nih.gov/blast/Blast.cgi?ALIGNMENTS=50&ALIGNMENT_VIEW=Pairwise&AUTO_FORMAT=Semiauto&CDD_SEARCH=on&CLIENT=web&COMPOSITION_BASED_STATISTICS=on&DATABASE=nr&DESCRIPTIONS=100&ENTREZ_QUERY=(none)&EXPECT=10&FILTER=L&FORMAT_BLOCK_ON_RESPAGE=None&FORMAT_OBJECT=Alignment&FORMAT_TYPE=HTML&GAPCOSTS=11+1&I_THRESH=0.001&LAYOUT=TwoWindows&MATRIX_NAME=BLOSUM62&NCBI_GI=on&PAGE=Proteins&PROGRAM=blastp&QUERY=MSTTKLEGVRHSRRKKGDSRLQRPENDEDSATLQSRYDDVEERRLKEWENYQRSHRMKEEQNSCRAFLDMSIDDVLSGRLVFELFDDVVPRTVENFRALITGSCGIDTNTGVKLDYLGTQVHHVDHNNNIIVLGELDSFNLSSTGTPIADEGYRHRHTERGLLTMISEGPHTSGSVFGITLGPSPSLDFKQVVFGRAIDDLSLLEKLEAVPLDDVGRPVLPVTVSFCGALTGEKPPGRQQVPAAADDSASSEHVSCAGEE&SERVICE=plain&SET_DEFAULTS.x=9&SET_DEFAULTS.y=5&SHOW_OVERVIEW=on&WORD_SIZE=3&END_OF_HTTPGET=Yes) against nr

Unformatted [sequence string](../../../../D:%5CProteomic%20data%5C2010-1-8%5Ccgi%5Cgetseq.pl%3FTBA927_IPI+Tb927%2E6%2E1400+seq) for pasting into other applications

Fixed modifications: MMTS (C),(N-TERM)_iTRAQ,Lysine(K)_iTRAQ

Variable modifications: Oxidation (M)

Cleavage by Trypsin: cuts C-term side of KR unless next residue is P

Sequence Coverage: **6%**

Matched peptides shown in **Bold Red**

**1** **MSTTK**LEGVR HSRRKKGDSR LQRPENDEDS ATLQSRYDDV EERRLKEWEN

**51** YQRSHRMKEE QNSCRAFLDM SIDDVLSGR**L VFELFDDVVP R**TVENFRALI

**101** TGSCGIDTNT GVKLDYLGTQ VHHVDHNNNI IVLGELDSFN LSSTGTPIAD

**151** EGYRHRHTER GLLTMISEGP HTSGSVFGIT LGPSPSLDFK QVVFGRAIDD

**201** LSLLEKLEAV PLDDVGRPVL PVTVSFCGAL TGEKPPGRQQ VPAAADDSAS

**251** SEHVSCAGEE

**Start - End Observed Mr(expt) Mr(calc) Delta Miss Sequence**

**1 - 5 855.45 854.44 854.39 0.06 0 -.MSTTK.L**  ([Ions score 5](../../../../D:%5CProteomic%20data%5C2010-1-8%5CZQ%5C1265.htm))

**80 - 91 1592.89 1591.88 1591.87 0.00 0 R.LVFELFDDVVPR.T**  ([Ions score 48](../../../../D:%5CProteomic%20data%5C2010-1-8%5CZQ%5C1266.htm))

MS/MS Fragmentation of **LVFELFDDVVPR**
Found in **Tb927.6.1400**, PPIasecyclophilin-type peptidyl-prolyl cis-trans isomerase, putative; Trypanosoma bruceichr 6Manual


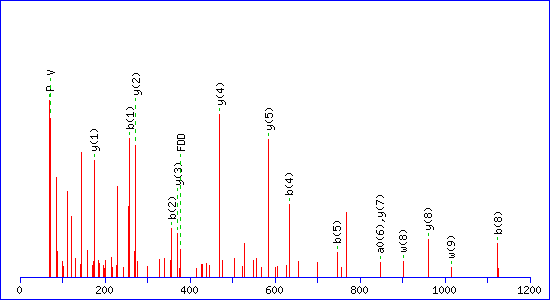


**MONOISOTOPIC mass of neutral peptide Mr(calc):** 1591.87

**Fixed modifications:** MMTS (C),(N-TERM)_iTRAQ,Lysine(K)_iTRAQ

**Ions Score:** 48 **Expect:** 0.0018

**Matches (Bold Red):** 20/164 fragment ions using 21 most intense peaks

| **#** | **Immon.** | **a** | **a^0^** | **b** | **b^0^** | **Seq.** | **v** | **w** | **y** | **y*** | **y^0^** | **#** |
| --- | --- | --- | --- | --- | --- | --- | --- | --- | --- | --- | --- | --- |
| **1** | 86.10 | 230.20 |  | **258.19** |  | **L** |  |  |  |  |  | **12** |
| **2** | **72.08** | 329.27 |  | **357.26** |  | **V** | 1291.63 | 1304.65 | 1335.69 | 1318.67 | 1317.68 | **11** |
| **3** | 120.08 | 476.34 |  | 504.33 |  | **F** | 1144.56 |  | 1236.63 | 1219.60 | 1218.62 | **10** |
| **4** | 102.05 | 605.38 | 587.37 | **633.37** | 615.36 | **E** | 1015.52 | **1014.53** | 1089.56 | 1072.53 | 1071.55 | **9** |
| **5** | 86.10 | 718.46 | 700.45 | **746.46** | 728.45 | **L** | 902.44 | **901.44** | **960.51** | 943.49 | 942.50 | **8** |
| **6** | 120.08 | 865.53 | **847.52** | 893.53 | 875.52 | **F** | 755.37 |  | **847.43** | 830.40 | 829.42 | **7** |
| **7** | 88.04 | 980.56 | 962.55 | 1008.55 | 990.54 | **D** | 640.34 | 639.35 | 700.36 | 683.34 | 682.35 | **6** |
| **8** | 88.04 | 1095.58 | 1077.57 | **1123.58** | 1105.57 | **D** | 525.31 | 524.32 | **585.34** | 568.31 | 567.32 | **5** |
| **9** | **72.08** | 1194.65 | 1176.64 | 1222.65 | 1204.64 | **V** | 426.25 | 439.27 | **470.31** | 453.28 |  | **4** |
| **10** | **72.08** | 1293.72 | 1275.71 | 1321.72 | 1303.71 | **V** | 327.18 | 340.20 | **371.24** | 354.21 |  | **3** |
| **11** | **70.07** | 1390.77 | 1372.76 | 1418.77 | 1400.76 | **P** | 230.12 | 229.13 | **272.17** | 255.15 |  | **2** |
| **12** | 129.11 |  |  |  |  | **R** | 74.02 | 73.03 | **175.12** | 158.09 |  | **1** |

| **Seq** | **ya** | **yb** | **Seq** | **ya** | **yb** | **Seq** | **ya** | **yb** |
| --- | --- | --- | --- | --- | --- | --- | --- | --- |
| **VF** | 219.15 | 247.14 | **VFE** | 348.19 | 376.19 | **VFEL** | 461.28 | 489.27 |
| **VFELF** | 608.34 | 636.34 | **FE** | 249.12 | 277.12 | **FEL** | 362.21 | 390.20 |
| **FELF** | 509.28 | 537.27 | **FELFD** | 624.30 | 652.30 | **EL** | 215.14 | 243.13 |
| **ELF** | 362.21 | 390.20 | **ELFD** | 477.23 | 505.23 | **ELFDD** | 592.26 | 620.26 |
| **ELFDDV** | 691.33 | 719.32 | **LF** | 233.16 | 261.16 | **LFD** | 348.19 | 376.19 |
| **LFDD** | 463.22 | 491.21 | **LFDDV** | 562.29 | 590.28 | **LFDDVV** | 661.36 | 689.35 |
| **FD** | 235.11 | 263.10 | **FDD** | 350.13 | **378.13** | **FDDV** | 449.20 | 477.20 |
| **FDDVV** | 548.27 | 576.27 | **FDDVVP** | 645.32 | 673.32 | **DD** | 203.07 | 231.06 |
| **DDV** | 302.13 | 330.13 | **DDVV** | 401.20 | 429.20 | **DDVVP** | 498.26 | 526.25 |
| **DV** | 187.11 | 215.10 | **DVV** | 286.18 | 314.17 | **DVVP** | 383.23 | 411.22 |
| **VV** | 171.15 | 199.14 | **VVP** | 268.20 | 296.20 | **VP** | 169.13 | 197.13 |

47. Tb10.70.4840

Match to: **Tb10.70.4840** Score: **47**

**hypothetical protein, conserved; Trypanosoma bruceichr 10Manual**

Nominal mass (M_r_): **41237**; Calculated pI value: **4.94**

NCBI BLAST search of [Tb10.70.4840](http://www.ncbi.nlm.nih.gov/blast/Blast.cgi?ALIGNMENTS=50&ALIGNMENT_VIEW=Pairwise&AUTO_FORMAT=Semiauto&CDD_SEARCH=on&CLIENT=web&COMPOSITION_BASED_STATISTICS=on&DATABASE=nr&DESCRIPTIONS=100&ENTREZ_QUERY=(none)&EXPECT=10&FILTER=L&FORMAT_BLOCK_ON_RESPAGE=None&FORMAT_OBJECT=Alignment&FORMAT_TYPE=HTML&GAPCOSTS=11+1&I_THRESH=0.001&LAYOUT=TwoWindows&MATRIX_NAME=BLOSUM62&NCBI_GI=on&PAGE=Proteins&PROGRAM=blastp&QUERY=MCNDDARQEGVQWPSSQQLAALADIEGDVQMKAAQLGLLSSEYRANIARVLHHYEPQAATFRTIIYASEQKRKAVERELVWTERIALLQEQRCGTTIVSTRDLMVLEREPLHPVECSLKECYQKLENVAYNELCVLRNYEYPPRQAVATMRMIMRVRGEEDLSWENVQVVLSENYFYTFFVSRMRTLLQKRLPDDVLEELEQYCLNPEHAPEALAVVSVPLGVIGSLLHAVRDYFQVMDLVKRPVEPMSVEERRKKAGELRRALQTLKEDAATATEGMADLKARIASKFVTVRDEYDDTMCPLHDDLEKKTEDFLKVLSGDIPAEEGATGEGECPDAM&SERVICE=plain&SET_DEFAULTS.x=9&SET_DEFAULTS.y=5&SHOW_OVERVIEW=on&WORD_SIZE=3&END_OF_HTTPGET=Yes) against nr

Unformatted [sequence string](../../../../D:%5CProteomic%20data%5C2010-1-8%5Ccgi%5Cgetseq.pl%3FTBA927_IPI+Tb10%2E70%2E4840+seq) for pasting into other applications

Fixed modifications: MMTS (C),(N-TERM)_iTRAQ,Lysine(K)_iTRAQ

Variable modifications: Oxidation (M)

Cleavage by Trypsin: cuts C-term side of KR unless next residue is P

Sequence Coverage: **11%**

Matched peptides shown in **Bold Red**

**1** MCNDDARQEG VQWPSSQQLA ALADIEGDVQ MKAAQLGLLS SEYRANIARV

**51** LHHYEPQAAT FRTIIYASEQ KRKAVER**ELV WTERIALLQE QR**CGTTIVST

**101** RDLMVLEREP LHPVECSLKE CYQK**LENVAY NELCVLR**NYE YPPRQAVATM

**151** RMIMRVRGEE DLSWENVQVV LSENYFYTFF VSRMRTLLQK RLPDDVLEEL

**201** EQYCLNPEHA PEALAVVSVP LGVIGSLLHA VRDYFQVMDL VK**RPVEPMSV**

**251 EER**RKKAGEL RRALQTLKED AATATEGMAD LKARIASKFV TVRDEYDDTM

**301** CPLHDDLEKK TEDFLKVLSG DIPAEEGATG EGECPDAM

**Start - End Observed Mr(expt) Mr(calc) Delta Miss Sequence**

**78 - 84 1076.57 1075.56 1075.58 -0.02 0 R.ELVWTER.I**  ([Ions score 19](../../../../D:%5CProteomic%20data%5C2010-1-8%5CZQ%5C1279.htm))

**85 - 92 1114.66 1113.65 1113.66 -0.01 0 R.IALLQEQR.C**  ([Ions score 47](../../../../D:%5CProteomic%20data%5C2010-1-8%5CZQ%5C1280.htm))

**125 - 137 1725.88 1724.88 1724.87 0.01 0 K.LENVAYNELCVLR.N**  ([Ions score 8](../../../../D:%5CProteomic%20data%5C2010-1-8%5CZQ%5C1282.htm))

**243 - 253 1472.78 1471.77 1471.76 0.02 0 K.RPVEPMSVEER.R**  ([Ions score 13](../../../../D:%5CProteomic%20data%5C2010-1-8%5CZQ%5C1281.htm))

MS/MS Fragmentation of **IALLQEQR**
Found in **Tb10.70.4840**, hypothetical protein, conserved; Trypanosoma bruceichr 10Manual


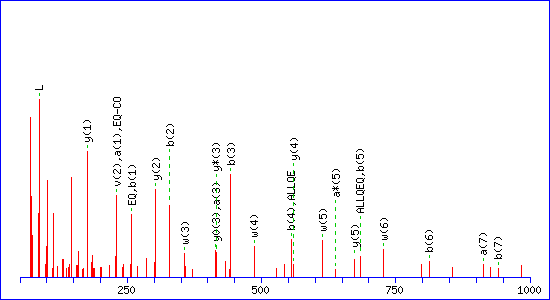


**MONOISOTOPIC mass of neutral peptide Mr(calc):** 1113.66

**Fixed modifications:** MMTS (C),(N-TERM)_iTRAQ,Lysine(K)_iTRAQ

**Ions Score:** 47 **Expect:** 0.0017

**Matches (Bold Red):** 31/94 fragment ions using 27 most intense peaks

| **#** | **Immon.** | **a** | **a*** | **a^0^** | **b** | **b*** | **b^0^** | **Seq.** | **v** | **w** | **y** | **y*** | **y^0^** | **#** |
| --- | --- | --- | --- | --- | --- | --- | --- | --- | --- | --- | --- | --- | --- | --- |
| **1** | **86.10** | **230.20** |  |  | **258.19** |  |  | **I** |  |  |  |  |  | **8** |
| **2** | 44.05 | 301.24 |  |  | **329.23** |  |  | **A** | 841.45 |  | 857.48 | 840.46 | 839.47 | **7** |
| **3** | **86.10** | **414.32** |  |  | **442.31** |  |  | **L** | 728.37 | **727.37** | 786.45 | 769.42 | 768.44 | **6** |
| **4** | **86.10** | 527.40 |  |  | **555.40** |  |  | **L** | 615.28 | **614.29** | **673.36** | 656.34 | 655.35 | **5** |
| **5** | 101.07 | 655.46 | **638.44** |  | **683.46** | 666.43 |  | **Q** | 487.23 | **486.23** | **560.28** | 543.25 | 542.27 | **4** |
| **6** | 102.05 | 784.51 | 767.48 | 766.49 | **812.50** | 795.47 | 794.49 | **E** | 358.18 | **357.19** | 432.22 | **415.19** | **414.21** | **3** |
| **7** | 101.07 | **912.56** | 895.54 | 894.55 | **940.56** | 923.53 | 922.55 | **Q** | **230.12** | 229.13 | **303.18** | 286.15 |  | **2** |
| **8** | 129.11 |  |  |  |  |  |  | **R** | 74.02 | 73.03 | **175.12** | 158.09 |  | **1** |

| **Seq** | **ya** | **yb** | **Seq** | **ya** | **yb** | **Seq** | **ya** | **yb** |
| --- | --- | --- | --- | --- | --- | --- | --- | --- |
| **AL** | 157.13 | 185.13 | **ALL** | 270.22 | 298.21 | **ALLQ** | 398.28 | 426.27 |
| **ALLQE** | 527.32 | **555.31** | **ALLQEQ** | 655.38 | **683.37** | **LL** | 199.18 | 227.18 |
| **LLQ** | 327.24 | 355.23 | **LLQE** | 456.28 | 484.28 | **LLQEQ** | 584.34 | 612.34 |
| **LQ** | 214.16 | 242.15 | **LQE** | 343.20 | 371.19 | **LQEQ** | 471.26 | 499.25 |
| **QE** | **230.11** | **258.11** | **QEQ** | 358.17 | 386.17 | **EQ** | **230.11** | **258.11** |

48. Tb11.02.4150

Match to: **Tb11.02.4150** Score: **45**

**PPDKpyruvate phosphate dikinase; Trypanosoma bruceichr 11Manual**

Nominal mass (M_r_): **108612**; Calculated pI value: **8.93**

NCBI BLAST search of [Tb11.02.4150](http://www.ncbi.nlm.nih.gov/blast/Blast.cgi?ALIGNMENTS=50&ALIGNMENT_VIEW=Pairwise&AUTO_FORMAT=Semiauto&CDD_SEARCH=on&CLIENT=web&COMPOSITION_BASED_STATISTICS=on&DATABASE=nr&DESCRIPTIONS=100&ENTREZ_QUERY=(none)&EXPECT=10&FILTER=L&FORMAT_BLOCK_ON_RESPAGE=None&FORMAT_OBJECT=Alignment&FORMAT_TYPE=HTML&GAPCOSTS=11+1&I_THRESH=0.001&LAYOUT=TwoWindows&MATRIX_NAME=BLOSUM62&NCBI_GI=on&PAGE=Proteins&PROGRAM=blastp&QUERY=MVAKKWVYYFGGGKADGNKNMKELLGGKGANLAEMVNLGIPVPPGFTITTEACKTYQETETIPQEVADQVRENVSRVEKEMGAKFGDPTNPLLFSVRSGAAASMPGMMDTVLNLGLNKVTVDAWVRRAPRLERFVYDSYRRFITMYADIVMQVGREDFEEALSRMKERRGTKFDTDLTASDLKELCDGYLELFELKTGCSFPQDPVMQLFAAIKAVFRSWGNPRATIYRRMNNITGLLGTAVNVQAMVFGNINDRSATGVAFSRSPSTGENFFFGEYLVNAQGEDVVAGIRTPQQINHSLSLRWAKAHGVGEEERRKRYPSMEEAMPENYRLLCDVRKRLENHYRDMQDLEFTVQDGRLWLLQCRNGKRTIHAAVRIAIDMVNEGLISREEAVLRIDPYQVDHLMHPNLEPGAEKANKPIGRGLAASPGAAVGQVVFDAESAKEWSGRGKKVIMVRLETSPEDLAGMDAACGILTARGGMTSHAAVVARGMGKCCVSGCGDMVIRGKSFKLNGSVFREGDYITIDGSKGLIYAGKLKLRSPDLKGSFQTILQWCQEMKRLGVRTNADTPADAAKARSFGAEGVGLCRTEHMFFEGSRINFIREMILADSASGRKAALDKLLPIQRADFVGILRAMRGLPVTIRLLDPPLHEFVPHDAAAQFELAQKLGMPAEKVRNRVNALHELNPMLGHRGCRLGITYPEIYNMQVRAIIEAAIAVSEEGSSVIPEIMVPLVGKKEELSLIREEVVKTAEAVITKSGKRVHYTVGTMIEVPRAAVTADSIAQKADFFSFGTNDLTQMGCGFSRDDAGPFLRHYGNLGIYAQDPFQSIDQEGIGELVRIAVTKGRRVKPMLKMGICGEHGGDPATIGFCHKVGLDYVSCSPFRVPVAIVAAAHASIKDRRAAMKARKGFAAKL&SERVICE=plain&SET_DEFAULTS.x=9&SET_DEFAULTS.y=5&SHOW_OVERVIEW=on&WORD_SIZE=3&END_OF_HTTPGET=Yes) against nr

Unformatted [sequence string](../../../../D:%5CProteomic%20data%5C2010-1-8%5Ccgi%5Cgetseq.pl%3FTBA927_IPI+Tb11%2E02%2E4150+seq) for pasting into other applications

Fixed modifications: MMTS (C),(N-TERM)_iTRAQ,Lysine(K)_iTRAQ

Variable modifications: Oxidation (M)

Cleavage by Trypsin: cuts C-term side of KR unless next residue is P

Sequence Coverage: **5%**

Matched peptides shown in **Bold Red**

**1** MVAKKWVYYF GGGKADGNKN MKELLGGKGA NLAEMVNLGI PVPPGFTITT

**51** EACKTYQETE TIPQEVADQV RENVSRVEKE MGAKFGDPTN PLLFSVRSGA

**101** AASMPGMMDT VLNLGLNKVT VDAWVRRAPR LER**FVYDSYR** RFITMYADIV

**151** MQVGREDFEE ALSRMKERRG TKFDTDLTAS DLKELCDGYL ELFELKTGCS

**201** FPQDPVMQLF AAIKAVFRSW GNPRATIYRR MNNITGLLGT AVNVQAMVFG

**251** NINDRSATGV AFSRSPSTGE NFFFGEYLVN AQGEDVVAGI R**TPQQINHSL**

**301 SLR**WAKAHGV GEEERRKRYP SMEEAMPENY RLLCDVRKRL ENHYRDMQDL

**351** EFTVQDGRLW LLQCRNGK**RT IHAAVR**IAID MVNEGLISRE EAVLRIDPYQ

**401** VDHLMHPNLE PGAEKANKPI GRGLAASPGA AVGQVVFDAE SAKEWSGRGK

**451** KVIMVRLETS PEDLAGMDAA CGILTARGGM TSHAAVVARG MGKCCVSGCG

**501** DMVIRGKSFK LNGSVFREGD YITIDGSKGL IYAGKLKLRS PDLKGSFQTI

**551** LQWCQEMKRL GVR**TNADTPA DAAK**ARSFGA EGVGLCRTEH MFFEGSRINF

**601** IREMILADSA SGRKAALDKL LPIQRADFVG ILRAMRGLPV TIRLLDPPLH

**651** EFVPHDAAAQ FELAQKLGMP AEKVRNR**VNA LHELNPMLGH R**GCRLGITYP

**701** EIYNMQVRAI IEAAIAVSEE GSSVIPEIMV PLVGKKEELS LIREEVVKTA

**751** EAVITKSGKR VHYTVGTMIE VPRAAVTADS IAQKADFFSF GTNDLTQMGC

**801** GFSRDDAGPF LRHYGNLGIY AQDPFQSIDQ EGIGELVRIA VTKGRRVKPM

**851** LKMGICGEHG GDPATIGFCH KVGLDYVSCS PFRVPVAIVA AAHASIKDRR

**901** AAMKARKGFA AKL

**Start - End Observed Mr(expt) Mr(calc) Delta Miss Sequence**

**134 - 140 1093.59 1092.58 1092.54 0.04 0 R.FVYDSYR.R**  ([Ions score 3](../../../../D:%5CProteomic%20data%5C2010-1-8%5CZQ%5C1305.htm))

**292 - 303 1537.85 1536.84 1536.85 -0.01 0 R.TPQQINHSLSLR.W**  ([Ions score 45](../../../../D:%5CProteomic%20data%5C2010-1-8%5CZQ%5C1307.htm))

**369 - 376 1067.56 1066.55 1066.65 -0.10 1 K.RTIHAAVR.I**  ([Ions score 10](../../../../D:%5CProteomic%20data%5C2010-1-8%5CZQ%5C1304.htm))

**564 - 574 1362.70 1361.70 1361.61 0.08 0 R.TNADTPADAAK.A**  ([Ions score 4](../../../../D:%5CProteomic%20data%5C2010-1-8%5CZQ%5C1306.htm))

**678 - 691 1744.89 1743.88 1743.93 -0.05 0 R.VNALHELNPMLGHR.G**  ([Ions score 1](../../../../D:%5CProteomic%20data%5C2010-1-8%5CZQ%5C1308.htm))

MS/MS Fragmentation of **TPQQINHSLSLR**
Found in **Tb11.02.4150**, PPDKpyruvate phosphate dikinase; Trypanosoma bruceichr 11Manual


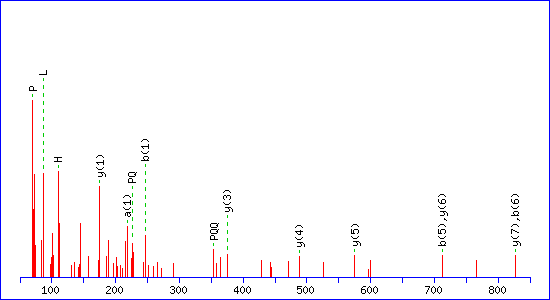


**MONOISOTOPIC mass of neutral peptide Mr(calc):** 1536.85

**Fixed modifications:** MMTS (C),(N-TERM)_iTRAQ,Lysine(K)_iTRAQ

**Ions Score:** 45 **Expect:** 0.0038

**Matches (Bold Red):** 17/195 fragment ions using 16 most intense peaks

| **#** | **Immon.** | **a** | **a*** | **a^0^** | **b** | **b*** | **b^0^** | **Seq.** | **v** | **w** | **w'** | **y** | **y*** | **y^0^** | **#** |
| --- | --- | --- | --- | --- | --- | --- | --- | --- | --- | --- | --- | --- | --- | --- | --- |
| **1** | 74.06 | **218.16** |  | 200.15 | **246.16** |  | 228.15 | **T** |  |  |  |  |  |  | **12** |
| **2** | **70.07** | 315.22 |  | 297.20 | 343.21 |  | 325.20 | **P** | 1250.66 | 1249.66 |  | 1292.71 | 1275.68 | 1274.70 | **11** |
| **3** | 101.07 | 443.27 | 426.25 | 425.26 | 471.27 | 454.24 | 453.26 | **Q** | 1122.60 | 1121.61 |  | 1195.65 | 1178.63 | 1177.64 | **10** |
| **4** | 101.07 | 571.33 | 554.31 | 553.32 | 599.33 | 582.30 | 581.32 | **Q** | 994.54 | 993.55 |  | 1067.60 | 1050.57 | 1049.59 | **9** |
| **5** | **86.10** | 684.42 | 667.39 | 666.41 | **712.41** | 695.38 | 694.40 | **I** | 881.46 | 894.48 | 908.49 | 939.54 | 922.51 | 921.53 | **8** |
| **6** | 87.06 | 798.46 | 781.43 | 780.45 | **826.45** | 809.43 | 808.44 | **N** | 767.42 | 766.42 |  | **826.45** | 809.43 | 808.44 | **7** |
| **7** | **110.07** | 935.52 | 918.49 | 917.51 | 963.51 | 946.49 | 945.50 | **H** | 630.36 |  |  | **712.41** | 695.38 | 694.40 | **6** |
| **8** | 60.04 | 1022.55 | 1005.52 | 1004.54 | 1050.55 | 1033.52 | 1032.53 | **S** | 543.32 | 542.33 |  | **575.35** | 558.32 | 557.34 | **5** |
| **9** | **86.10** | 1135.63 | 1118.61 | 1117.62 | 1163.63 | 1146.60 | 1145.62 | **L** | 430.24 | 429.25 |  | **488.32** | 471.29 | 470.31 | **4** |
| **10** | 60.04 | 1222.67 | 1205.64 | 1204.66 | 1250.66 | 1233.63 | 1232.65 | **S** | 343.21 | 342.21 |  | **375.24** | 358.21 | 357.22 | **3** |
| **11** | **86.10** | 1335.75 | 1318.72 | 1317.74 | 1363.75 | 1346.72 | 1345.73 | **L** | 230.12 | 229.13 |  | 288.20 | 271.18 |  | **2** |
| **12** | 129.11 |  |  |  |  |  |  | **R** | 74.02 | 73.03 |  | **175.12** | 158.09 |  | **1** |

| **Seq** | **ya** | **yb** | **Seq** | **ya** | **yb** | **Seq** | **ya** | **yb** |
| --- | --- | --- | --- | --- | --- | --- | --- | --- |
| **PQ** | 198.12 | **226.12** | **PQQ** | 326.18 | **354.18** | **PQQI** | 439.27 | 467.26 |
| **PQQIN** | 553.31 | 581.30 | **PQQINH** | 690.37 | 718.36 | **QQ** | 229.13 | 257.12 |
| **QQI** | 342.21 | 370.21 | **QQIN** | 456.26 | 484.25 | **QQINH** | 593.32 | 621.31 |
| **QQINHS** | 680.35 | 708.34 | **QI** | 214.16 | 242.15 | **QIN** | 328.20 | 356.19 |
| **QINH** | 465.26 | 493.25 | **QINHS** | 552.29 | 580.28 | **QINHSL** | 665.37 | 693.37 |
| **IN** | 200.14 | 228.13 | **INH** | 337.20 | 365.19 | **INHS** | 424.23 | 452.23 |
| **INHSL** | 537.31 | 565.31 | **INHSLS** | 624.35 | 652.34 | **NH** | 224.11 | 252.11 |
| **NHS** | 311.15 | 339.14 | **NHSL** | 424.23 | 452.23 | **NHSLS** | 511.26 | 539.26 |
| **NHSLSL** | 624.35 | 652.34 | **HS** | 197.10 | 225.10 | **HSL** | 310.19 | 338.18 |
| **HSLS** | 397.22 | 425.21 | **HSLSL** | 510.30 | 538.30 | **SL** | 173.13 | 201.12 |
| **SLS** | 260.16 | 288.16 | **SLSL** | 373.24 | 401.24 | **LS** | 173.13 | 201.12 |
| **LSL** | 286.21 | 314.21 | **SL** | 173.13 | 201.12 |  |  |  |

49. Tb11.01.4390

Match to: **Tb11.01.4390** Score: **45**

**leucine-rich repeat protein (LRRP), putative; Trypanosoma bruceichr 11Manual**

Nominal mass (M_r_): **74855**; Calculated pI value: **5.24**

NCBI BLAST search of [Tb11.01.4390](http://www.ncbi.nlm.nih.gov/blast/Blast.cgi?ALIGNMENTS=50&ALIGNMENT_VIEW=Pairwise&AUTO_FORMAT=Semiauto&CDD_SEARCH=on&CLIENT=web&COMPOSITION_BASED_STATISTICS=on&DATABASE=nr&DESCRIPTIONS=100&ENTREZ_QUERY=(none)&EXPECT=10&FILTER=L&FORMAT_BLOCK_ON_RESPAGE=None&FORMAT_OBJECT=Alignment&FORMAT_TYPE=HTML&GAPCOSTS=11+1&I_THRESH=0.001&LAYOUT=TwoWindows&MATRIX_NAME=BLOSUM62&NCBI_GI=on&PAGE=Proteins&PROGRAM=blastp&QUERY=MQQLAETLIMEQHVEALSLPEVELDDEGLITLFTLITRRDHLGSPNTNWETCDNENQALQESGPAAASEAAPNDAQTENKESASPEAGAALTPVGKSSPTKTLWFGVRCLDLSRCKVTDPTNVFSLFRCPVLEVLVLPPSNQLGDVHLRGILEGCPHLHTIDISGNTALTTACVKYIGRHSTIKVLRLENCPGIDQLDLPNVEVLFSSLSYVIKLHAPELRRLPVPVVHSRVLFNFKAPRLREITLKGIVVDRGTLDSLKETAGEDSACEPKLARSVLSSCDKSAYSDGQKQSTAATMQLLCAGFIDCTFTAESEVREFTKKQKALLRFSLHGCKGVVDANLTRLPATLMDLDVSDAARLTNRGLEIIATTLPQLIRLNLKNAGPQISNEGIRLLRGLVNLEVLNLLRLPQILPEVVSAVANDLPWLRKLYHETAVVGPRSVVAATSAPVHLDVLREDDEDTTRNAVGECAKELLQLRNETALAFWMDAQLPKPTSLIPPRTAAADSIDLNAPNPPLTANTTFYEAACRKLSRSGNGSAFGSQRRAITPRNADAAANQNEESEKVNVLVVSSGDDSPLMASMSGGVEGVNADCGDVDDKSFSTAPDQLRAMENALDEKNRLVIEKKPLGPWDEEDLVEKHRRIA&SERVICE=plain&SET_DEFAULTS.x=9&SET_DEFAULTS.y=5&SHOW_OVERVIEW=on&WORD_SIZE=3&END_OF_HTTPGET=Yes) against nr

Unformatted [sequence string](../../../../D:%5CProteomic%20data%5C2010-1-8%5Ccgi%5Cgetseq.pl%3FTBA927_IPI+Tb11%2E01%2E4390+seq) for pasting into other applications

Fixed modifications: MMTS (C),(N-TERM)_iTRAQ,Lysine(K)_iTRAQ

Variable modifications: Oxidation (M)

Cleavage by Trypsin: cuts C-term side of KR unless next residue is P

Sequence Coverage: **3%**

Matched peptides shown in **Bold Red**

**1** MQQLAETLIM EQHVEALSLP EVELDDEGLI TLFTLITRRD HLGSPNTNWE

**51** TCDNENQALQ ESGPAAASEA APNDAQTENK ESASPEAGAA LTPVGKSSPT

**101** KTLWFGVRCL DLSRCKVTDP TNVFSLFRCP VLEVLVLPPS NQLGDVHLRG

**151** ILEGCPHLHT IDISGNTALT TACVKYIGRH STIKVLRLEN CPGIDQLDLP

**201** NVEVLFSSLS YVIKLHAPEL RRLPVPVVHS RVLFNFKAPR LREITLKGIV

**251** VDRGTLDSLK ETAGEDSACE PKLARSVLSS CDKSAYSDGQ KQSTAATMQL

**301** LCAGFIDCTF TAESEVREFT KKQKALLRFS LHGCKGVVDA NLTRLPATLM

**351** DLDVSDAARL TNR**GLEIIAT TLPQLIR**LNL KNAGPQISNE GIRLLRGLVN

**401** LEVLNLLRLP QILPEVVSAV ANDLPWLRKL YHETAVVGPR SVVAATSAPV

**451** HLDVLREDDE DTTRNAVGEC AKELLQLRNE TALAFWMDAQ LPKPTSLIPP

**501** RTAAADSIDL NAPNPPLTAN TTFYEAACRK LSRSGNGSAF GSQRRAITPR

**551** NADAAANQNE ESEKVNVLVV SSGDDSPLMA SMSGGVEGVN ADCGDVDDK**S**

**601 FSTAPDQLR**A MENALDEKNR LVIEKKPLGP WDEEDLVEKH RRIA

**Start - End Observed Mr(expt) Mr(calc) Delta Miss Sequence**

**364 - 377 1682.02 1681.01 1681.03 -0.01 0 R.GLEIIATTLPQLIR.L**  ([Ions score 45](../../../../D:%5CProteomic%20data%5C2010-1-8%5CZQ%5C1311.htm))

**600 - 609 1265.67 1264.66 1264.65 0.01 0 K.SFSTAPDQLR.A**  ([Ions score 12](../../../../D:%5CProteomic%20data%5C2010-1-8%5CZQ%5C1310.htm))

MS/MS Fragmentation of **GLEIIATTLPQLIR**
Found in **Tb11.01.4390**, leucine-rich repeat protein (LRRP), putative; Trypanosoma bruceichr 11Manual


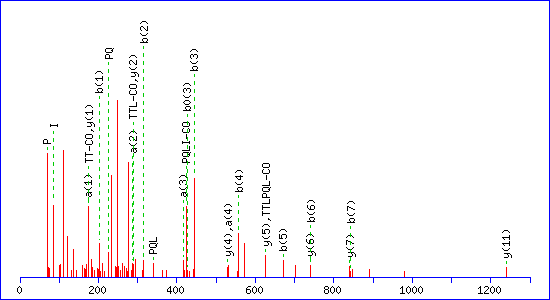


**MONOISOTOPIC mass of neutral peptide Mr(calc):** 1681.03

**Fixed modifications:** MMTS (C),(N-TERM)_iTRAQ,Lysine(K)_iTRAQ

**Ions Score:** 45 **Expect:** 0.0014

**Matches (Bold Red):** 34/224 fragment ions using 42 most intense peaks

| **#** | **Immon.** | **a** | **a*** | **a^0^** | **b** | **b*** | **b^0^** | **Seq.** | **v** | **w** | **w'** | **y** | **y*** | **y^0^** | **#** |
| --- | --- | --- | --- | --- | --- | --- | --- | --- | --- | --- | --- | --- | --- | --- | --- |
| **1** | 30.03 | **174.14** |  |  | **202.13** |  |  | **G** |  |  |  |  |  |  | **14** |
| **2** | **86.10** | **287.22** |  |  | **315.22** |  |  | **L** | 1422.83 | 1421.84 |  | 1480.91 | 1463.88 | 1462.90 | **13** |
| **3** | 102.05 | **416.26** |  | 398.25 | **444.26** |  | **426.25** | **E** | 1293.79 | 1292.79 |  | 1367.83 | 1350.80 | 1349.82 | **12** |
| **4** | **86.10** | **529.35** |  | 511.34 | **557.34** |  | 539.33 | **I** | 1180.70 | 1193.73 | 1207.74 | **1238.78** | 1221.76 | 1220.77 | **11** |
| **5** | **86.10** | 642.43 |  | 624.42 | **670.43** |  | 652.42 | **I** | 1067.62 | 1080.64 | 1094.66 | 1125.70 | 1108.67 | 1107.69 | **10** |
| **6** | 44.05 | 713.47 |  | 695.46 | **741.46** |  | 723.45 | **A** | 996.58 |  |  | 1012.61 | 995.59 | 994.60 | **9** |
| **7** | 74.06 | 814.52 |  | 796.51 | **842.51** |  | 824.50 | **T** | 895.54 | 908.56 | 910.54 | 941.58 | 924.55 | 923.57 | **8** |
| **8** | 74.06 | 915.56 |  | 897.55 | 943.56 |  | 925.55 | **T** | 794.49 | 807.51 | 809.49 | **840.53** | 823.50 | 822.52 | **7** |
| **9** | **86.10** | 1028.65 |  | 1010.64 | 1056.64 |  | 1038.63 | **L** | 681.40 | 680.41 |  | **739.48** | 722.46 |  | **6** |
| **10** | **70.07** | 1125.70 |  | 1107.69 | 1153.70 |  | 1135.68 | **P** | 584.35 | 583.36 |  | **626.40** | 609.37 |  | **5** |
| **11** | 101.07 | 1253.76 | 1236.73 | 1235.75 | 1281.75 | 1264.73 | 1263.74 | **Q** | 456.29 | 455.30 |  | **529.35** | 512.32 |  | **4** |
| **12** | **86.10** | 1366.84 | 1349.82 | 1348.83 | 1394.84 | 1377.81 | 1376.83 | **L** | 343.21 | 342.21 |  | 401.29 | 384.26 |  | **3** |
| **13** | **86.10** | 1479.93 | 1462.90 | 1461.92 | 1507.92 | 1490.90 | 1489.91 | **I** | 230.12 | 243.15 | 257.16 | **288.20** | 271.18 |  | **2** |
| **14** | 129.11 |  |  |  |  |  |  | **R** | 74.02 | 73.03 |  | **175.12** | 158.09 |  | **1** |

| **Seq** | **ya** | **yb** | **Seq** | **ya** | **yb** | **Seq** | **ya** | **yb** |
| --- | --- | --- | --- | --- | --- | --- | --- | --- |
| **LE** | 215.14 | 243.13 | **LEI** | 328.22 | 356.22 | **LEII** | 441.31 | 469.30 |
| **LEIIA** | 512.34 | 540.34 | **LEIIAT** | 613.39 | 641.39 | **EI** | 215.14 | 243.13 |
| **EII** | 328.22 | 356.22 | **EIIA** | 399.26 | 427.26 | **EIIAT** | 500.31 | 528.30 |
| **EIIATT** | 601.36 | 629.35 | **II** | 199.18 | 227.18 | **IIA** | 270.22 | 298.21 |
| **IIAT** | 371.27 | 399.26 | **IIATT** | 472.31 | 500.31 | **IIATTL** | 585.40 | 613.39 |
| **IIATTLP** | 682.45 | 710.44 | **IA** | 157.13 | 185.13 | **IAT** | 258.18 | 286.18 |
| **IATT** | 359.23 | 387.22 | **IATTL** | 472.31 | 500.31 | **IATTLP** | 569.37 | 597.36 |
| **IATTLPQ** | 697.42 | 725.42 | **AT** | 145.10 | 173.09 | **ATT** | 246.14 | 274.14 |
| **ATTL** | 359.23 | 387.22 | **ATTLP** | 456.28 | 484.28 | **ATTLPQ** | 584.34 | 612.34 |
| **ATTLPQL** | 697.42 | 725.42 | **TT** | **175.11** | 203.10 | **TTL** | **288.19** | 316.19 |
| **TTLP** | 385.24 | 413.24 | **TTLPQ** | 513.30 | 541.30 | **TTLPQL** | **626.39** | 654.38 |
| **TL** | 187.14 | 215.14 | **TLP** | 284.20 | 312.19 | **TLPQ** | 412.26 | 440.25 |
| **TLPQL** | 525.34 | 553.33 | **TLPQLI** | 638.42 | 666.42 | **LP** | 183.15 | 211.14 |
| **LPQ** | 311.21 | **339.20** | **LPQL** | **424.29** | 452.29 | **LPQLI** | 537.38 | 565.37 |
| **PQ** | 198.12 | **226.12** | **PQL** | 311.21 | **339.20** | **PQLI** | **424.29** | 452.29 |
| **QL** | 214.16 | 242.15 | **QLI** | 327.24 | 355.23 | **LI** | 199.18 | 227.18 |

50. Tb10.70.2280

Match to: **Tb10.70.2280** Score: **44**

**hypothetical protein, conserved; Trypanosoma bruceichr 10Manual**

Nominal mass (M_r_): **60257**; Calculated pI value: **8.95**

NCBI BLAST search of [Tb10.70.2280](http://www.ncbi.nlm.nih.gov/blast/Blast.cgi?ALIGNMENTS=50&ALIGNMENT_VIEW=Pairwise&AUTO_FORMAT=Semiauto&CDD_SEARCH=on&CLIENT=web&COMPOSITION_BASED_STATISTICS=on&DATABASE=nr&DESCRIPTIONS=100&ENTREZ_QUERY=(none)&EXPECT=10&FILTER=L&FORMAT_BLOCK_ON_RESPAGE=None&FORMAT_OBJECT=Alignment&FORMAT_TYPE=HTML&GAPCOSTS=11+1&I_THRESH=0.001&LAYOUT=TwoWindows&MATRIX_NAME=BLOSUM62&NCBI_GI=on&PAGE=Proteins&PROGRAM=blastp&QUERY=MSDSNCYCRCRYIYVHIRFLFATISKVTRSYLLTEDKKKRMAFPHREQIDELKLIAEMLDTFARTRPEFHHQVGPLPDRIRDAIGPIESALRESGRTMTGDITEKERPKRADGLPELVNEGRQTPGQRRSNEGKKVVITKPSSVTAYRIPREKVPGGPLQISKEVSKYIAATLYNILEGATRISKASSSHIFIRKDDEMISIANCAARLAFPPQLVHHRCLGSVDAEVLGSGIALNQYTVDSSRVTSSLLIFPVFTTETPRKSAVAVVHMENKCQGTMPFSKSDEGVILTTSQLIGQFMSMFPQMDWVNSFFDPVTQHILAPFEPKKRLPKPTRRGWKGKVSVTTEEPGAVESQIDENYWRKIEECEPPLLIKRESLPRLGSNKASPQGLSAVPTLREIDAYVENMQSCWNRGISNYVGLSEEEHSNNIELKVVRRELTRIKALYEEAEEQLRLYRLEGQDYECGFRSIKGELDSYIRKRNKTDIN&SERVICE=plain&SET_DEFAULTS.x=9&SET_DEFAULTS.y=5&SHOW_OVERVIEW=on&WORD_SIZE=3&END_OF_HTTPGET=Yes) against nr

Unformatted [sequence string](../../../../D:%5CProteomic%20data%5C2010-1-8%5Ccgi%5Cgetseq.pl%3FTBA927_IPI+Tb10%2E70%2E2280+seq) for pasting into other applications

Fixed modifications: MMTS (C),(N-TERM)_iTRAQ,Lysine(K)_iTRAQ

Variable modifications: Oxidation (M)

Cleavage by Trypsin: cuts C-term side of KR unless next residue is P

Sequence Coverage: **2%**

Matched peptides shown in **Bold Red**

**1** MSDSNCYCRC RYIYVHIRFL FATISKVTRS YLLTEDKKKR MAFPHREQID

**51** ELK**LIAEMLD TFAR**TRPEFH HQVGPLPDRI RDAIGPIESA LRESGRTMTG

**101** DITEKERPKR ADGLPELVNE GRQTPGQRRS NEGKKVVITK PSSVTAYRIP

**151** REKVPGGPLQ ISKEVSKYIA ATLYNILEGA TRISKASSSH IFIRKDDEMI

**201** SIANCAARLA FPPQLVHHRC LGSVDAEVLG SGIALNQYTV DSSRVTSSLL

**251** IFPVFTTETP RKSAVAVVHM ENKCQGTMPF SKSDEGVILT TSQLIGQFMS

**301** MFPQMDWVNS FFDPVTQHIL APFEPKKRLP KPTRRGWKGK VSVTTEEPGA

**351** VESQIDENYW RKIEECEPPL LIKRESLPRL GSNKASPQGL SAVPTLREID

**401** AYVENMQSCW NRGISNYVGL SEEEHSNNIE LKVVRRELTR IKALYEEAEE

**451** QLRLYRLEGQ DYECGFRSIK GELDSYIRKR NKTDIN

MS/MS Fragmentation of **LIAEMLDTFAR**
Found in **Tb10.70.2280**, hypothetical protein, conserved; Trypanosoma bruceichr 10Manual


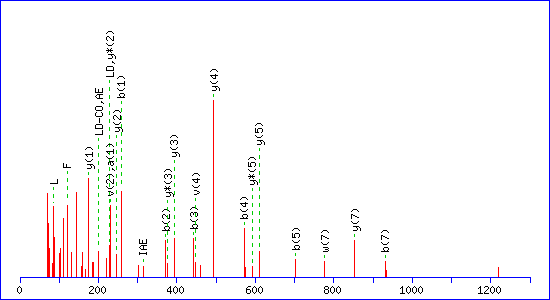


**MONOISOTOPIC mass of neutral peptide Mr(calc):** 1422.77

**Fixed modifications:** MMTS (C),(N-TERM)_iTRAQ,Lysine(K)_iTRAQ

**Ions Score:** 44 **Expect:** 0.0053

**Matches (Bold Red):** 27/149 fragment ions using 32 most intense peaks

| **#** | **Immon.** | **a** | **a^0^** | **b** | **b^0^** | **Seq.** | **v** | **w** | **w'** | **y** | **y*** | **y^0^** | **#** |
| --- | --- | --- | --- | --- | --- | --- | --- | --- | --- | --- | --- | --- | --- |
| **1** | **86.10** | **230.20** |  | **258.19** |  | **L** |  |  |  |  |  |  | **11** |
| **2** | **86.10** | 343.28 |  | **371.28** |  | **I** | 1108.51 | 1121.53 | 1135.55 | 1166.59 | 1149.56 | 1148.58 | **10** |
| **3** | 44.05 | 414.32 |  | **442.31** |  | **A** | 1037.47 |  |  | 1053.50 | 1036.48 | 1035.49 | **9** |
| **4** | 102.05 | 543.36 | 525.35 | **571.36** | 553.35 | **E** | 908.43 | 907.43 |  | 982.47 | 965.44 | 964.46 | **8** |
| **5** | 104.05 | 674.40 | 656.39 | **702.40** | 684.39 | **M** | 777.39 | **776.39** |  | **853.42** | 836.40 | 835.41 | **7** |
| **6** | **86.10** | 787.49 | 769.48 | 815.48 | 797.47 | **L** | 664.30 | 663.31 |  | 722.38 | 705.36 | 704.37 | **6** |
| **7** | 88.04 | 902.51 | 884.50 | **930.51** | 912.50 | **D** | 549.28 | 548.28 |  | **609.30** | **592.27** | 591.29 | **5** |
| **8** | 74.06 | 1003.56 | 985.55 | 1031.56 | 1013.55 | **T** | **448.23** | 461.25 | 463.23 | **494.27** | 477.25 | 476.26 | **4** |
| **9** | **120.08** | 1150.63 | 1132.62 | 1178.63 | 1160.61 | **F** | 301.16 |  |  | **393.22** | **376.20** |  | **3** |
| **10** | 44.05 | 1221.67 | 1203.66 | 1249.66 | 1231.65 | **A** | **230.12** |  |  | **246.16** | **229.13** |  | **2** |
| **11** | 129.11 |  |  |  |  | **R** | 74.02 | 73.03 |  | **175.12** | 158.09 |  | **1** |

| **Seq** | **ya** | **yb** | **Seq** | **ya** | **yb** | **Seq** | **ya** | **yb** |
| --- | --- | --- | --- | --- | --- | --- | --- | --- |
| **IA** | 157.13 | 185.13 | **IAE** | 286.18 | **314.17** | **IAEM** | 417.22 | 445.21 |
| **IAEML** | 530.30 | 558.30 | **IAEMLD** | 645.33 | 673.32 | **AE** | 173.09 | **201.09** |
| **AEM** | 304.13 | 332.13 | **AEML** | 417.22 | 445.21 | **AEMLD** | 532.24 | 560.24 |
| **AEMLDT** | 633.29 | 661.29 | **EM** | 233.10 | 261.09 | **EML** | 346.18 | 374.17 |
| **EMLD** | 461.21 | 489.20 | **EMLDT** | 562.25 | 590.25 | **ML** | 217.14 | 245.13 |
| **MLD** | 332.16 | 360.16 | **MLDT** | 433.21 | 461.21 | **MLDTF** | 580.28 | 608.27 |
| **MLDTFA** | 651.32 | 679.31 | **LD** | **201.12** | **229.12** | **LDT** | 302.17 | 330.17 |
| **LDTF** | 449.24 | 477.23 | **LDTFA** | 520.28 | 548.27 | **DT** | 189.09 | 217.08 |
| **DTF** | 336.16 | 364.15 | **DTFA** | 407.19 | 435.19 | **TF** | 221.13 | 249.12 |
| **TFA** | 292.17 | 320.16 | **FA** | 191.12 | 219.11 |  |  |  |

51. Tb927.7.6890

Match to: **Tb927.7.6890** Score: **44**

**hypothetical protein, conserved; Trypanosoma bruceichr 7Manual**

Nominal mass (M_r_): **30734**; Calculated pI value: **5.51**

NCBI BLAST search of [Tb927.7.6890](http://www.ncbi.nlm.nih.gov/blast/Blast.cgi?ALIGNMENTS=50&ALIGNMENT_VIEW=Pairwise&AUTO_FORMAT=Semiauto&CDD_SEARCH=on&CLIENT=web&COMPOSITION_BASED_STATISTICS=on&DATABASE=nr&DESCRIPTIONS=100&ENTREZ_QUERY=(none)&EXPECT=10&FILTER=L&FORMAT_BLOCK_ON_RESPAGE=None&FORMAT_OBJECT=Alignment&FORMAT_TYPE=HTML&GAPCOSTS=11+1&I_THRESH=0.001&LAYOUT=TwoWindows&MATRIX_NAME=BLOSUM62&NCBI_GI=on&PAGE=Proteins&PROGRAM=blastp&QUERY=MSKYANPDYWEERYRSNDTTFDWYLTFDNLEPVLRPMLQPAEQIHVLVVGCGNSRLSPCMYEHLNVRKITNVDVSPTVISQMTRRYKGMDEMRWICCDLIHTAPDKLLTLLCPEDALFDFVIDKGLVDATLGGSNSFHNLYTLTKNLARVMKNGGRFLSVSYGAPETRIDHFRRRKLNFDVEHRTIEKSVFASGAAPTGSYHVYIMTKLGERQAVTAGAADDGALSGDTDDDDDFYDRFMTKANTTSADAARFD&SERVICE=plain&SET_DEFAULTS.x=9&SET_DEFAULTS.y=5&SHOW_OVERVIEW=on&WORD_SIZE=3&END_OF_HTTPGET=Yes) against nr

Unformatted [sequence string](../../../../D:%5CProteomic%20data%5C2010-1-8%5Ccgi%5Cgetseq.pl%3FTBA927_IPI+Tb927%2E7%2E6890+seq) for pasting into other applications

Fixed modifications: MMTS (C),(N-TERM)_iTRAQ,Lysine(K)_iTRAQ

Variable modifications: Oxidation (M)

Cleavage by Trypsin: cuts C-term side of KR unless next residue is P

Sequence Coverage: **3%**

Matched peptides shown in **Bold Red**

**1** MSK**YANPDYW EER**YRSNDTT FDWYLTFDNL EPVLRPMLQP AEQIHVLVVG

**51** CGNSRLSPCM YEHLNVRKIT NVDVSPTVIS QMTRRYKGMD EMRWICCDLI

**101** HTAPDKLLTL LCPEDALFDF VIDKGLVDAT LGGSNSFHNL YTLTKNLARV

**151** MKNGGRFLSV SYGAPETRID HFRRRKLNFD VEHRTIEKSV FASGAAPTGS

**201** YHVYIMTKLG ERQAVTAGAA DDGALSGDTD DDDDFYDRFM TKANTTSADA

**251** ARFD

MS/MS Fragmentation of **YANPDYWEER**
Found in **Tb927.7.6890**, hypothetical protein, conserved; Trypanosoma bruceichr 7Manual


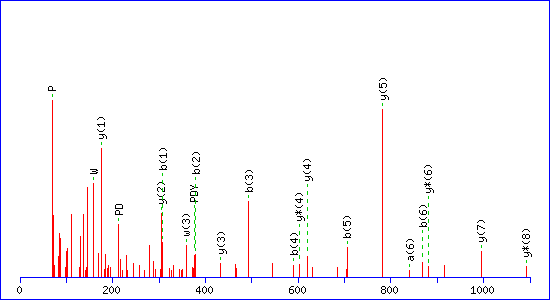


**MONOISOTOPIC mass of neutral peptide Mr(calc):** 1485.66

**Fixed modifications:** MMTS (C),(N-TERM)_iTRAQ,Lysine(K)_iTRAQ

**Ions Score:** 44 **Expect:** 0.005

**Matches (Bold Red):** 21/136 fragment ions using 27 most intense peaks

| **#** | **Immon.** | **a** | **a*** | **a^0^** | **b** | **b*** | **b^0^** | **Seq.** | **v** | **w** | **y** | **y*** | **y^0^** | **#** |
| --- | --- | --- | --- | --- | --- | --- | --- | --- | --- | --- | --- | --- | --- | --- |
| **1** | 136.08 | 280.18 |  |  | **308.17** |  |  | **Y** |  |  |  |  |  | **10** |
| **2** | 44.05 | 351.22 |  |  | **379.21** |  |  | **A** | 1163.48 |  | 1179.51 | 1162.48 | 1161.50 | **9** |
| **3** | 87.06 | 465.26 | 448.23 |  | **493.25** | 476.23 |  | **N** | 1049.43 | 1048.44 | 1108.47 | **1091.44** | 1090.46 | **8** |
| **4** | **70.07** | 562.31 | 545.28 |  | **590.31** | 573.28 |  | **P** | 952.38 | 951.38 | **994.43** | 977.40 | 976.42 | **7** |
| **5** | 88.04 | 677.34 | 660.31 | 659.33 | **705.33** | 688.31 | 687.32 | **D** | 837.35 | 836.36 | 897.37 | **880.35** | 879.36 | **6** |
| **6** | 136.08 | **840.40** | 823.37 | 822.39 | **868.40** | 851.37 | 850.39 | **Y** | 674.29 |  | **782.35** | 765.32 | 764.34 | **5** |
| **7** | **159.09** | 1026.48 | 1009.45 | 1008.47 | 1054.48 | 1037.45 | 1036.46 | **W** | 488.21 |  | **619.28** | **602.26** | 601.27 | **4** |
| **8** | 102.05 | 1155.52 | 1138.50 | 1137.51 | 1183.52 | 1166.49 | 1165.51 | **E** | 359.17 | **358.17** | **433.20** | 416.18 | 415.19 | **3** |
| **9** | 102.05 | 1284.57 | 1267.54 | 1266.56 | 1312.56 | 1295.53 | 1294.55 | **E** | 230.12 | 229.13 | **304.16** | 287.13 | 286.15 | **2** |
| **10** | 129.11 |  |  |  |  |  |  | **R** | 74.02 | 73.03 | **175.12** | 158.09 |  | **1** |

| **Seq** | **ya** | **yb** | **Seq** | **ya** | **yb** | **Seq** | **ya** | **yb** |
| --- | --- | --- | --- | --- | --- | --- | --- | --- |
| **AN** | 158.09 | 186.09 | **ANP** | 255.15 | 283.14 | **ANPD** | 370.17 | 398.17 |
| **ANPDY** | 533.24 | 561.23 | **NP** | 184.11 | 212.10 | **NPD** | 299.13 | 327.13 |
| **NPDY** | 462.20 | 490.19 | **NPDYW** | 648.28 | 676.27 | **PD** | 185.09 | **213.09** |
| **PDY** | 348.16 | **376.15** | **PDYW** | 534.23 | 562.23 | **PDYWE** | 663.28 | 691.27 |
| **DY** | 251.10 | 279.10 | **DYW** | 437.18 | 465.18 | **DYWE** | 566.22 | 594.22 |
| **DYWEE** | 695.27 | 723.26 | **YW** | 322.16 | 350.15 | **YWE** | 451.20 | 479.19 |
| **YWEE** | 580.24 | 608.24 | **WE** | 288.13 | 316.13 | **WEE** | 417.18 | 445.17 |
| **EE** | 231.10 | 259.09 |  |  |  |  |  |  |

52. Tb11.02.4230

Match to: **Tb11.02.4230** Score: **43**

**hypothetical protein, conserved; Trypanosoma bruceichr 11Manual**

Nominal mass (M_r_): **100672**; Calculated pI value: **5.06**

NCBI BLAST search of [Tb11.02.4230](http://www.ncbi.nlm.nih.gov/blast/Blast.cgi?ALIGNMENTS=50&ALIGNMENT_VIEW=Pairwise&AUTO_FORMAT=Semiauto&CDD_SEARCH=on&CLIENT=web&COMPOSITION_BASED_STATISTICS=on&DATABASE=nr&DESCRIPTIONS=100&ENTREZ_QUERY=(none)&EXPECT=10&FILTER=L&FORMAT_BLOCK_ON_RESPAGE=None&FORMAT_OBJECT=Alignment&FORMAT_TYPE=HTML&GAPCOSTS=11+1&I_THRESH=0.001&LAYOUT=TwoWindows&MATRIX_NAME=BLOSUM62&NCBI_GI=on&PAGE=Proteins&PROGRAM=blastp&QUERY=MLFVTPTGESRFAGDEGLYAAIRAAQLARALHGATIRAHVSSNQPINPNSIPLALPPAKWVIPSTAERAMREVLSSISGADAAAVAGYGLQVPVALPDEGQLEALRVVLPYIHHLKPHPVMTFDDVQRLERLMTLYNSNVTCLNLGDGAVMPHNHAEAPPSTVVAKINELMQRFPLPAPKPAKGSDDGTEDTEEEEMEDETDYSINEELMAWCQSQEVQYTTYDDAIRQRAAYELDAFRNICKILMNDTKIRVLLLDHNQLCAPNEDERVSLVPLRMLAKVIDANETIKVLDLSSNMLGPFGFGVIAKALTKNISIVALDLSDNQLGTPSPDTDEDPEHQPDDPVFGEEYSGLEAISEVLKKNKFLRCLRLAHNDIHSGGEGEEAPPVEVNELDPENDATTVDVESWQDLPLWHLMGPLRHYHRLRVLDLSGNLLGPVGAHMVATALAENHSVEVLDLTDNGIGFHGLHYISKVLLSSQKTVLNTLILRRNQLAGKKTSKAQQKMALAAMQATAAALRENGRLRRLSVAGNYLGTTLASALLSTIATVSSLEELDLESNDICGDVAAPHDTTALGFVAAALYSTAMCNRRPTLRVLNLANNNIRSSGLNVLFPSAASMPISLVDVNLSRNNIDNAVDALTHLMISSPVLQRLTLAHNAITDASVVVPGVSSNTYLAELDLSHNLLGSRKPQYCEDPQAQMKNVERLVDVFNNHPSLEDVNLSFNDFEDVHGPILARLCEDHGSKGKLRRINLCGNHEIKQCDINNMVRALPQKSGIEVFYISSTYPATTTSGGVIFPVGRDAALDAPTDRQQQQIPLLKLMHETVHQCPSLLDINCDLQRSAMKSEESADGDAGADVGGRTVEEIKQCLLLNALMAPQV&SERVICE=plain&SET_DEFAULTS.x=9&SET_DEFAULTS.y=5&SHOW_OVERVIEW=on&WORD_SIZE=3&END_OF_HTTPGET=Yes) against nr

Unformatted [sequence string](../../../../D:%5CProteomic%20data%5C2010-1-8%5Ccgi%5Cgetseq.pl%3FTBA927_IPI+Tb11%2E02%2E4230+seq) for pasting into other applications

Fixed modifications: MMTS (C),(N-TERM)_iTRAQ,Lysine(K)_iTRAQ

Variable modifications: Oxidation (M)

Cleavage by Trypsin: cuts C-term side of KR unless next residue is P

Sequence Coverage: **2%**

Matched peptides shown in **Bold Red**

**1** MLFVTPTGES R**FAGDEGLYA AIR**AAQLARA LHGATIRAHV SSNQPINPNS

**51** IPLALPPAK**W VIPSTAER**AM REVLSSISGA DAAAVAGYGL QVPVALPDEG

**101** QLEALRVVLP YIHHLKPHPV MTFDDVQRLE RLMTLYNSNV TCLNLGDGAV

**151** MPHNHAEAPP STVVAKINEL MQRFPLPAPK PAKGSDDGTE DTEEEEMEDE

**201** TDYSINEELM AWCQSQEVQY TTYDDAIRQR AAYELDAFRN ICKILMNDTK

**251** IRVLLLDHNQ LCAPNEDERV SLVPLRMLAK VIDANETIKV LDLSSNMLGP

**301** FGFGVIAKAL TKNISIVALD LSDNQLGTPS PDTDEDPEHQ PDDPVFGEEY

**351** SGLEAISEVL KKNKFLRCLR LAHNDIHSGG EGEEAPPVEV NELDPENDAT

**401** TVDVESWQDL PLWHLMGPLR HYHRLRVLDL SGNLLGPVGA HMVATALAEN

**451** HSVEVLDLTD NGIGFHGLHY ISKVLLSSQK TVLNTLILRR NQLAGKKTSK

**501** AQQKMALAAM QATAAALREN GRLRRLSVAG NYLGTTLASA LLSTIATVSS

**551** LEELDLESND ICGDVAAPHD TTALGFVAAA LYSTAMCNRR PTLRVLNLAN

**601** NNIRSSGLNV LFPSAASMPI SLVDVNLSRN NIDNAVDALT HLMISSPVLQ

**651** RLTLAHNAIT DASVVVPGVS SNTYLAELDL SHNLLGSRKP QYCEDPQAQM

**701** KNVERLVDVF NNHPSLEDVN LSFNDFEDVH GPILARLCED HGSKGKLRRI

**751** NLCGNHEIKQ CDINNMVRAL PQKSGIEVFY ISSTYPATTT SGGVIFPVGR

**801** DAALDAPTDR QQQQIPLLKL MHETVHQCPS LLDINCDLQR SAMKSEESAD

**851** GDAGADVGGR TVEEIKQCLL LNALMAPQV

**Start - End Observed Mr(expt) Mr(calc) Delta Miss Sequence**

**12 - 23 1426.70 1425.70 1425.74 -0.04 0 R.FAGDEGLYAAIR.A**  ([Ions score 43](../../../../D:%5CProteomic%20data%5C2010-1-8%5CZQ%5C1332.htm))

**60 - 68 1202.66 1201.65 1201.66 -0.01 0 K.WVIPSTAER.A**  ([Ions score 20](../../../../D:%5CProteomic%20data%5C2010-1-8%5CZQ%5C1331.htm))

MS/MS Fragmentation of **FAGDEGLYAAIR**
Found in **Tb11.02.4230**, hypothetical protein, conserved; Trypanosoma bruceichr 11Manual


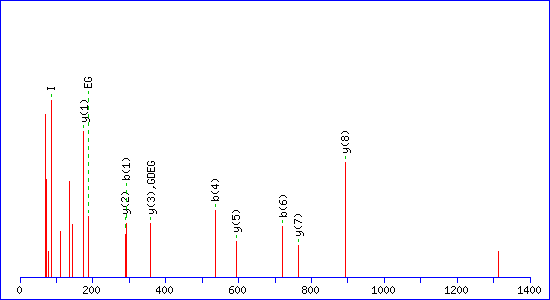


**MONOISOTOPIC mass of neutral peptide Mr(calc):** 1425.74

**Fixed modifications:** MMTS (C),(N-TERM)_iTRAQ,Lysine(K)_iTRAQ

**Ions Score:** 43 **Expect:** 0.0078

**Matches (Bold Red):** 13/165 fragment ions using 14 most intense peaks

| **#** | **Immon.** | **a** | **a^0^** | **b** | **b^0^** | **Seq.** | **v** | **w** | **w'** | **y** | **y*** | **y^0^** | **#** |
| --- | --- | --- | --- | --- | --- | --- | --- | --- | --- | --- | --- | --- | --- |
| **1** | 120.08 | 264.18 |  | **292.18** |  | **F** |  |  |  |  |  |  | **12** |
| **2** | 44.05 | 335.22 |  | 363.22 |  | **A** | 1119.54 |  |  | 1135.57 | 1118.55 | 1117.56 | **11** |
| **3** | 30.03 | 392.24 |  | 420.24 |  | **G** |  |  |  | 1064.54 | 1047.51 | 1046.53 | **10** |
| **4** | 88.04 | 507.27 | 489.26 | **535.26** | 517.25 | **D** | 947.49 | 946.50 |  | 1007.52 | 990.49 | 989.51 | **9** |
| **5** | 102.05 | 636.31 | 618.30 | 664.31 | 646.30 | **E** | 818.45 | 817.46 |  | **892.49** | 875.46 | 874.48 | **8** |
| **6** | 30.03 | 693.33 | 675.32 | **721.33** | 703.32 | **G** |  |  |  | **763.45** | 746.42 |  | **7** |
| **7** | **86.10** | 806.42 | 788.41 | 834.41 | 816.40 | **L** | 648.35 | 647.35 |  | 706.42 | 689.40 |  | **6** |
| **8** | 136.08 | 969.48 | 951.47 | 997.48 | 979.46 | **Y** | 485.28 |  |  | **593.34** | 576.31 |  | **5** |
| **9** | 44.05 | 1040.52 | 1022.51 | 1068.51 | 1050.50 | **A** | 414.25 |  |  | 430.28 | 413.25 |  | **4** |
| **10** | 44.05 | 1111.55 | 1093.54 | 1139.55 | 1121.54 | **A** | 343.21 |  |  | **359.24** | 342.21 |  | **3** |
| **11** | **86.10** | 1224.64 | 1206.63 | 1252.63 | 1234.62 | **I** | 230.12 | 243.15 | 257.16 | **288.20** | 271.18 |  | **2** |
| **12** | 129.11 |  |  |  |  | **R** | 74.02 | 73.03 |  | **175.12** | 158.09 |  | **1** |

| **Seq** | **ya** | **yb** | **Seq** | **ya** | **yb** | **Seq** | **ya** | **yb** |
| --- | --- | --- | --- | --- | --- | --- | --- | --- |
| **AG** | 101.07 | 129.07 | **AGD** | 216.10 | 244.09 | **AGDE** | 345.14 | 373.14 |
| **AGDEG** | 402.16 | 430.16 | **AGDEGL** | 515.25 | 543.24 | **AGDEGLY** | 678.31 | 706.30 |
| **GD** | 145.06 | 173.06 | **GDE** | 274.10 | 302.10 | **GDEG** | 331.12 | **359.12** |
| **GDEGL** | 444.21 | 472.20 | **GDEGLY** | 607.27 | 635.27 | **GDEGLYA** | 678.31 | 706.30 |
| **DE** | 217.08 | 245.08 | **DEG** | 274.10 | 302.10 | **DEGL** | 387.19 | 415.18 |
| **DEGLY** | 550.25 | 578.25 | **DEGLYA** | 621.29 | 649.28 | **DEGLYAA** | 692.32 | 720.32 |
| **EG** | 159.08 | **187.07** | **EGL** | 272.16 | 300.16 | **EGLY** | 435.22 | 463.22 |
| **EGLYA** | 506.26 | 534.26 | **EGLYAA** | 577.30 | 605.29 | **EGLYAAI** | 690.38 | 718.38 |
| **GL** | 143.12 | 171.11 | **GLY** | 306.18 | 334.18 | **GLYA** | 377.22 | 405.21 |
| **GLYAA** | 448.26 | 476.25 | **GLYAAI** | 561.34 | 589.33 | **LY** | 249.16 | 277.15 |
| **LYA** | 320.20 | 348.19 | **LYAA** | 391.23 | 419.23 | **LYAAI** | 504.32 | 532.31 |
| **YA** | 207.11 | 235.11 | **YAA** | 278.15 | 306.14 | **YAAI** | 391.23 | 419.23 |
| **AA** | 115.09 | 143.08 | **AAI** | 228.17 | 256.17 | **AI** | 157.13 | 185.13 |

53. Tb927.3.2110

Match to: **Tb927.3.2110** Score: **42**

**TFIIF-stimulated CTD phosphatase, putative; Trypanosoma bruceichr 3Manual**

Nominal mass (M_r_): **49612**; Calculated pI value: **6.23**

NCBI BLAST search of [Tb927.3.2110](http://www.ncbi.nlm.nih.gov/blast/Blast.cgi?ALIGNMENTS=50&ALIGNMENT_VIEW=Pairwise&AUTO_FORMAT=Semiauto&CDD_SEARCH=on&CLIENT=web&COMPOSITION_BASED_STATISTICS=on&DATABASE=nr&DESCRIPTIONS=100&ENTREZ_QUERY=(none)&EXPECT=10&FILTER=L&FORMAT_BLOCK_ON_RESPAGE=None&FORMAT_OBJECT=Alignment&FORMAT_TYPE=HTML&GAPCOSTS=11+1&I_THRESH=0.001&LAYOUT=TwoWindows&MATRIX_NAME=BLOSUM62&NCBI_GI=on&PAGE=Proteins&PROGRAM=blastp&QUERY=MNHDAMSFFQNISARLSTVRNCTKKPHCRREEEAQPQQPIALPKQSSTPPPLISSVAPPTQSVVNEKAASTPVVLPSRVQPTVSARWSGPEAEVPESAARPRRNRTPCPPVKHRGEKSEEELRQVSSIIMTQAQEPASKSVRTGAAASLVEAYRCVNSIRDTGEPPGERTDDVTNINGSGDNDDGGNTKQARAKLYENAVSEQVRRIQKMPKNTSPVNAKNHASLLPRQLPRFRDKITLILDLDETLVHSSLTSQSRHHDLVLDVRMENTSTTVYVAFRPFMREFLQAVAPLFEVIIFTASVSVYCNQLMDAIDTDNILGSLRLYREHCSILNGAYVKDLSLLGRDLDRVAIIDNSPVAYLFQQRNAIPIPSWFDDPGDNELQQLIPMLEILAAESEVYTVLDQYNAVLHLQQEQARQNSPHY&SERVICE=plain&SET_DEFAULTS.x=9&SET_DEFAULTS.y=5&SHOW_OVERVIEW=on&WORD_SIZE=3&END_OF_HTTPGET=Yes) against nr

Unformatted [sequence string](../../../../D:%5CProteomic%20data%5C2010-1-8%5Ccgi%5Cgetseq.pl%3FTBA927_IPI+Tb927%2E3%2E2110+seq) for pasting into other applications

Fixed modifications: MMTS (C),(N-TERM)_iTRAQ,Lysine(K)_iTRAQ

Variable modifications: Oxidation (M)

Cleavage by Trypsin: cuts C-term side of KR unless next residue is P

Sequence Coverage: **5%**

Matched peptides shown in **Bold Red**

**1** MNHDAMSFFQ NISARLSTVR NCTKKPHCRR EEEAQPQQPI ALPKQSSTPP

**51** PLISSVAPPT QSVVNEKAAS TPVVLPSRVQ PTVSAR**WSGP EAEVPESAAR**

**101 PR**RNRTPCPP VKHRGEKSEE ELRQVSSIIM TQAQEPASKS VRTGAAASLV

**151** EAYRCVNSIR DTGEPPGERT DDVTNINGSG DNDDGGNTKQ ARAKLYENAV

**201** SEQVRRIQKM PKNTSPVNAK NHASLLPRQL PRFRDKITLI LDLDETLVHS

**251** SLTSQSR**HHD LVLDVR**MENT STTVYVAFRP FMREFLQAVA PLFEVIIFTA

**301** SVSVYCNQLM DAIDTDNILG SLRLYREHCS ILNGAYVKDL SLLGRDLDRV

**351** AIIDNSPVAY LFQQRNAIPI PSWFDDPGDN ELQQLIPMLE ILAAESEVYT

**401** VLDQYNAVLH LQQEQARQNS PHY

**Start - End Observed Mr(expt) Mr(calc) Delta Miss Sequence**

**87 - 102 1882.95 1881.94 1881.95 -0.00 0 R.WSGPEAEVPESAARPR.R**  ([Ions score 42](../../../../D:%5CProteomic%20data%5C2010-1-8%5CZQ%5C1335.htm))

**258 - 266 1247.61 1246.61 1246.69 -0.08 0 R.HHDLVLDVR.M**  ([Ions score 3](../../../../D:%5CProteomic%20data%5C2010-1-8%5CZQ%5C1334.htm))

MS/MS Fragmentation of **WSGPEAEVPESAARPR**
Found in **Tb927.3.2110**, TFIIF-stimulated CTD phosphatase, putative; Trypanosoma bruceichr 3Manual


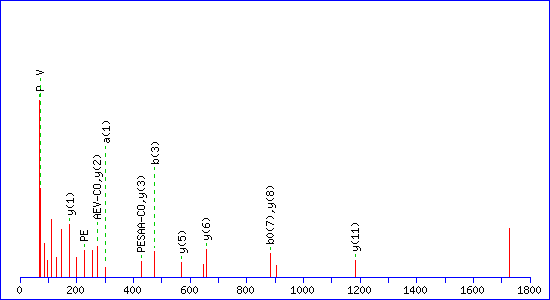


**MONOISOTOPIC mass of neutral peptide Mr(calc):** 1881.95

**Fixed modifications:** MMTS (C),(N-TERM)_iTRAQ,Lysine(K)_iTRAQ

**Ions Score:** 42 **Expect:** 0.0071

**Matches (Bold Red):** 18/265 fragment ions using 15 most intense peaks

| **#** | **Immon.** | **a** | **a*** | **a^0^** | **b** | **b*** | **b^0^** | **d** | **Seq.** | **v** | **w** | **y** | **y*** | **y^0^** | **#** |
| --- | --- | --- | --- | --- | --- | --- | --- | --- | --- | --- | --- | --- | --- | --- | --- |
| **1** | 159.09 | **303.19** |  |  | 331.19 |  |  |  | **W** |  |  |  |  |  | **16** |
| **2** | 60.04 | 390.23 |  | 372.22 | 418.22 |  | 400.21 |  | **S** | 1520.75 | 1519.75 | 1552.77 | 1535.74 | 1534.76 | **15** |
| **3** | 30.03 | 447.25 |  | 429.24 | **475.24** |  | 457.23 |  | **G** |  |  | 1465.74 | 1448.71 | 1447.73 | **14** |
| **4** | **70.07** | 544.30 |  | 526.29 | 572.30 |  | 554.28 |  | **P** | 1366.67 | 1365.68 | 1408.72 | 1391.69 | 1390.71 | **13** |
| **5** | 102.05 | 673.34 |  | 655.33 | 701.34 |  | 683.33 |  | **E** | 1237.63 | 1236.63 | 1311.67 | 1294.64 | 1293.65 | **12** |
| **6** | 44.05 | 744.38 |  | 726.37 | 772.37 |  | 754.36 |  | **A** | 1166.59 |  | **1182.62** | 1165.60 | 1164.61 | **11** |
| **7** | 102.05 | 873.42 |  | 855.41 | 901.42 |  | **883.41** |  | **E** | 1037.55 | 1036.55 | 1111.59 | 1094.56 | 1093.57 | **10** |
| **8** | **72.08** | 972.49 |  | 954.48 | 1000.49 |  | 982.48 |  | **V** | 938.48 | 951.50 | 982.54 | 965.52 | 964.53 | **9** |
| **9** | **70.07** | 1069.54 |  | 1051.53 | 1097.54 |  | 1079.53 |  | **P** | 841.43 | 840.43 | **883.47** | 866.45 | 865.46 | **8** |
| **10** | 102.05 | 1198.59 |  | 1180.58 | 1226.58 |  | 1208.57 |  | **E** | 712.38 | 711.39 | 786.42 | 769.40 | 768.41 | **7** |
| **11** | 60.04 | 1285.62 |  | 1267.61 | 1313.61 |  | 1295.60 |  | **S** | 625.35 | 624.36 | **657.38** | 640.35 | 639.37 | **6** |
| **12** | 44.05 | 1356.66 |  | 1338.64 | 1384.65 |  | 1366.64 |  | **A** | 554.32 |  | **570.35** | 553.32 |  | **5** |
| **13** | 44.05 | 1427.69 |  | 1409.68 | 1455.69 |  | 1437.68 |  | **A** | 483.28 |  | 499.31 | 482.28 |  | **4** |
| **14** | 129.11 | 1583.79 | 1566.77 | 1565.78 | 1611.79 | 1594.76 | 1593.78 | 1498.73 | **R** | 327.18 | 326.18 | **428.27** | 411.25 |  | **3** |
| **15** | **70.07** | 1680.85 | 1663.82 | 1662.84 | 1708.84 | 1691.81 | 1690.83 | 1654.83 | **P** | 230.12 | 229.13 | **272.17** | 255.15 |  | **2** |
| **16** | 129.11 |  |  |  |  |  |  |  | **R** | 74.02 | 73.03 | **175.12** | 158.09 |  | **1** |

| **Seq** | **ya** | **yb** | **Seq** | **ya** | **yb** | **Seq** | **ya** | **yb** |
| --- | --- | --- | --- | --- | --- | --- | --- | --- |
| **SG** | 117.07 | 145.06 | **SGP** | 214.12 | 242.11 | **SGPE** | 343.16 | 371.16 |
| **SGPEA** | 414.20 | 442.19 | **SGPEAE** | 543.24 | 571.24 | **SGPEAEV** | 642.31 | 670.30 |
| **GP** | 127.09 | 155.08 | **GPE** | 256.13 | 284.12 | **GPEA** | 327.17 | 355.16 |
| **GPEAE** | 456.21 | 484.20 | **GPEAEV** | 555.28 | 583.27 | **GPEAEVP** | 652.33 | 680.32 |
| **PE** | 199.11 | **227.10** | **PEA** | 270.14 | 298.14 | **PEAE** | 399.19 | 427.18 |
| **PEAEV** | 498.26 | 526.25 | **PEAEVP** | 595.31 | 623.30 | **EA** | 173.09 | 201.09 |
| **EAE** | 302.13 | 330.13 | **EAEV** | 401.20 | 429.20 | **EAEVP** | 498.26 | 526.25 |
| **EAEVPE** | 627.30 | 655.29 | **AE** | 173.09 | 201.09 | **AEV** | **272.16** | 300.16 |
| **AEVP** | 369.21 | 397.21 | **AEVPE** | 498.26 | 526.25 | **AEVPES** | 585.29 | 613.28 |
| **AEVPESA** | 656.32 | 684.32 | **EV** | 201.12 | 229.12 | **EVP** | 298.18 | 326.17 |
| **EVPE** | 427.22 | 455.21 | **EVPES** | 514.25 | 542.25 | **EVPESA** | 585.29 | 613.28 |
| **EVPESAA** | 656.32 | 684.32 | **VP** | 169.13 | 197.13 | **VPE** | 298.18 | 326.17 |
| **VPES** | 385.21 | 413.20 | **VPESA** | 456.25 | 484.24 | **VPESAA** | 527.28 | 555.28 |
| **VPESAAR** | 683.38 | 711.38 | **PE** | 199.11 | **227.10** | **PES** | 286.14 | 314.13 |
| **PESA** | 357.18 | 385.17 | **PESAA** | **428.21** | 456.21 | **PESAAR** | 584.32 | 612.31 |
| **PESAARP** | 681.37 | 709.36 | **ES** | 189.09 | 217.08 | **ESA** | 260.12 | 288.12 |
| **ESAA** | 331.16 | 359.16 | **ESAAR** | 487.26 | 515.26 | **ESAARP** | 584.32 | 612.31 |
| **SA** | 131.08 | 159.08 | **SAA** | 202.12 | 230.11 | **SAAR** | 358.22 | 386.21 |
| **SAARP** | 455.27 | 483.27 | **AA** | 115.09 | 143.08 | **AAR** | 271.19 | 299.18 |
| **AARP** | 368.24 | 396.24 | **AR** | 200.15 | 228.15 | **ARP** | 297.20 | 325.20 |
| **RP** | 226.17 | 254.16 |  |  |  |  |  |  |

54. Tb10.70.5350

Match to: **Tb10.70.5350** Score: **41**

**hypothetical protein, conserved; Trypanosoma bruceichr 10Manual**

Nominal mass (M_r_): **126080**; Calculated pI value: **5.65**

NCBI BLAST search of [Tb10.70.5350](http://www.ncbi.nlm.nih.gov/blast/Blast.cgi?ALIGNMENTS=50&ALIGNMENT_VIEW=Pairwise&AUTO_FORMAT=Semiauto&CDD_SEARCH=on&CLIENT=web&COMPOSITION_BASED_STATISTICS=on&DATABASE=nr&DESCRIPTIONS=100&ENTREZ_QUERY=(none)&EXPECT=10&FILTER=L&FORMAT_BLOCK_ON_RESPAGE=None&FORMAT_OBJECT=Alignment&FORMAT_TYPE=HTML&GAPCOSTS=11+1&I_THRESH=0.001&LAYOUT=TwoWindows&MATRIX_NAME=BLOSUM62&NCBI_GI=on&PAGE=Proteins&PROGRAM=blastp&QUERY=MPYFSAWAFCYHESVVSCRSLPIYWRYSSCTPPAHILATGSDNRRVPTSASVRCLRECHTHFLLRLLTNAGFFRRFLYFLCPIRQRLITLAGRLLNTPVADLLRSSVNIDFLCFLLFSTTHIITLMRAYTFPVYCNAEKITMTTEVSNDGMDIVNHMDIKVHGIQNVPPSWWSDGKHPTYPEHPFRYEVSFTIDGNQTFTLANGRLLQYLPQYRDYIENAALPPLSSTAKDVLRQMHEVPEAPTKGKSISTTKTTGNSQAVEDDAQPEARIIWITPPTSERAPTPSEELLGKRRKKTAGNSVGEDLTPVAVPEDDDTPPCMIRMPLDERHIMALEGMMLSGRPLELKFTRVLRPGLPTEWEDKQEWYFRAIIPVDLAPLSDPGSRELSADIQLQPVKAAVRTEEVKKTSKRSKNMAQGLRHEEIDTEGEHPYVTCKTSAVVSIELEKTLVQLPKDRIRPAVLPTNLIPKRPPPRKEFRDSTRQFSDMVASIAERMMHDCAALQDADSERKEEEILEVFENSGRLESYKEQLLPLVVNVVREKFLSDKDASPDVISRLTNELYVHLLNSIHCTLHNMVEGMSTPEQENANRVATAKPSTASPQGPDDGIDYVWLERAEEAETIRDYARATRCHQARIASCACAESFPDVWYDAASYFVRIGETTRAEQCFREAISHDPTHAPSLMAYGALLLTFDRFDEATVYLQAAVDAKPSSLSWGLISLLCDMHVLNLERGPRYESQRAHWEHEGTIAMREALSFSTDVDHTSVSKEVADYLLKLQHPGLANISLTRCSRGGHTEVLYARLFALGEQYNEALETLKNGEGLEPYIEEVTILRGDCYAALGRSDEAIREYKSVLCAKDKEPRRRFGPSYIHLGNLLITAGCYNDALGAFTIGIQAWPCSLTWLGAGIAYYRLNKIDAAEECLSESNTLNNTNPRTWAYLSLVCLRKERVELKTVLRQAIMQGLADPGLLTELGRDLVRACMGELGESCLRKALAVERECGREDSAVCCTAMYYLAGAVEGNNAEEAWMLYTAVANKTMDEVLRAKAEEQLAALGKS&SERVICE=plain&SET_DEFAULTS.x=9&SET_DEFAULTS.y=5&SHOW_OVERVIEW=on&WORD_SIZE=3&END_OF_HTTPGET=Yes) against nr

Unformatted [sequence string](../../../../D:%5CProteomic%20data%5C2010-1-8%5Ccgi%5Cgetseq.pl%3FTBA927_IPI+Tb10%2E70%2E5350+seq) for pasting into other applications

Fixed modifications: MMTS (C),(N-TERM)_iTRAQ,Lysine(K)_iTRAQ

Variable modifications: Oxidation (M)

Cleavage by Trypsin: cuts C-term side of KR unless next residue is P

Sequence Coverage: **1%**

Matched peptides shown in **Bold Red**

**1** MPYFSAWAFC YHESVVSCRS LPIYWRYSSC TPPAHILATG SDNRRVPTSA

**51** SVRCLRECHT HFLLRLLTNA GFFRRFLYFL CPIRQRLITL AGRLLNTPVA

**101** DLLRSSVNID FLCFLLFSTT HIITLMRAYT FPVYCNAEKI TMTTEVSNDG

**151** MDIVNHMDIK VHGIQNVPPS WWSDGKHPTY PEHPFRYEVS FTIDGNQTFT

**201** LANGRLLQYL PQYRDYIENA ALPPLSSTAK DVLRQMHEVP EAPTKGKSIS

**251** TTKTTGNSQA VEDDAQPEAR IIWITPPTSE RAPTPSEELL GKRRKKTAGN

**301** SVGEDLTPVA VPEDDDTPPC MIRMPLDERH IMALEGMMLS GRPLELKFTR

**351** VLRPGLPTEW EDKQEWYFRA IIPVDLAPLS DPGSRELSAD IQLQPVKAAV

**401** RTEEVKKTSK RSKNMAQGLR HEEIDTEGEH PYVTCKTSAV VSIELEKTLV

**451** QLPKDRIRPA VLPTNLIPKR PPPRKEFRDS TRQFSDMVAS IAERMMHDCA

**501** ALQDADSERK EEEILEVFEN SGRLESYKEQ LLPLVVNVVR EKFLSDKDAS

**551** PDVISRLTNE LYVHLLNSIH CTLHNMVEGM STPEQENANR VATAKPSTAS

**601** PQGPDDGIDY VWLERAEEAE TIRDYARATR CHQARIASCA CAESFPDVWY

**651** DAASYFVRIG ETTRAEQCFR EAISHDPTHA PSLMAYGALL LTFDRFDEAT

**701** VYLQAAVDAK PSSLSWGLIS LLCDMHVLNL ERGPRYESQR AHWEHEGTIA

**751** MREALSFSTD VDHTSVSKEV ADYLLK**LQHP GLANISLTR**C SRGGHTEVLY

**801** ARLFALGEQY NEALETLKNG EGLEPYIEEV TILRGDCYAA LGRSDEAIRE

**851** YKSVLCAKDK EPRRRFGPSY IHLGNLLITA GCYNDALGAF TIGIQAWPCS

**901** LTWLGAGIAY YRLNKIDAAE ECLSESNTLN NTNPRTWAYL SLVCLRKERV

**951** ELKTVLRQAI MQGLADPGLL TELGRDLVRA CMGELGESCL RKALAVEREC

**1001** GREDSAVCCT AMYYLAGAVE GNNAEEAWML YTAVANKTMD EVLRAKAEEQ

**1051** LAALGKS

MS/MS Fragmentation of **LQHPGLANISLTR**
Found in **Tb10.70.5350**, hypothetical protein, conserved; Trypanosoma bruceichr 10Manual


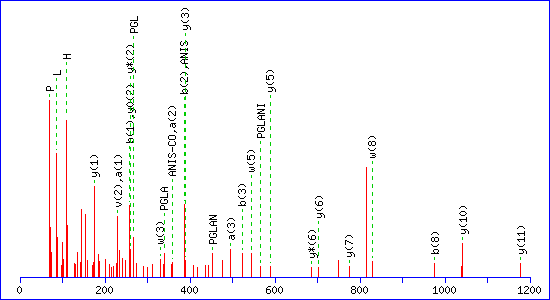


**MONOISOTOPIC mass of neutral peptide Mr(calc):** 1562.90

**Fixed modifications:** MMTS (C),(N-TERM)_iTRAQ,Lysine(K)_iTRAQ

**Ions Score:** 41 **Expect:** 0.0077

**Matches (Bold Red):** 33/209 fragment ions using 29 most intense peaks

| **#** | **Immon.** | **a** | **a*** | **a^0^** | **b** | **b*** | **b^0^** | **Seq.** | **v** | **w** | **w'** | **y** | **y*** | **y^0^** | **#** |
| --- | --- | --- | --- | --- | --- | --- | --- | --- | --- | --- | --- | --- | --- | --- | --- |
| **1** | **86.10** | **230.20** |  |  | **258.19** |  |  | **L** |  |  |  |  |  |  | **13** |
| **2** | 101.07 | **358.26** | 341.23 |  | **386.25** | 369.23 |  | **Q** | 1233.67 | 1232.67 |  | 1306.72 | 1289.70 | 1288.71 | **12** |
| **3** | **110.07** | **495.32** | 478.29 |  | **523.31** | 506.28 |  | **H** | 1096.61 |  |  | **1178.66** | 1161.64 | 1160.65 | **11** |
| **4** | **70.07** | 592.37 | 575.34 |  | 620.36 | 603.34 |  | **P** | 999.56 | 998.56 |  | **1041.61** | 1024.58 | 1023.59 | **10** |
| **5** | 30.03 | 649.39 | 632.36 |  | 677.39 | 660.36 |  | **G** |  |  |  | 944.55 | 927.53 | 926.54 | **9** |
| **6** | **86.10** | 762.47 | 745.45 |  | 790.47 | 773.44 |  | **L** | 829.45 | **828.46** |  | 887.53 | 870.50 | 869.52 | **8** |
| **7** | 44.05 | 833.51 | 816.49 |  | 861.51 | 844.48 |  | **A** | 758.42 |  |  | **774.45** | 757.42 | 756.44 | **7** |
| **8** | 87.06 | 947.55 | 930.53 |  | **975.55** | 958.52 |  | **N** | 644.37 | 643.38 |  | **703.41** | **686.38** | 685.40 | **6** |
| **9** | **86.10** | 1060.64 | 1043.61 |  | 1088.63 | 1071.61 |  | **I** | 531.29 | **544.31** | 558.32 | **589.37** | 572.34 | 571.36 | **5** |
| **10** | 60.04 | 1147.67 | 1130.64 | 1129.66 | 1175.67 | 1158.64 | 1157.66 | **S** | 444.26 | 443.26 |  | 476.28 | 459.26 | 458.27 | **4** |
| **11** | **86.10** | 1260.75 | 1243.73 | 1242.74 | 1288.75 | 1271.72 | 1270.74 | **L** | 331.17 | **330.18** |  | **389.25** | 372.22 | 371.24 | **3** |
| **12** | 74.06 | 1361.80 | 1344.78 | 1343.79 | 1389.80 | 1372.77 | 1371.79 | **T** | **230.12** | 243.15 | 245.12 | 276.17 | **259.14** | **258.16** | **2** |
| **13** | 129.11 |  |  |  |  |  |  | **R** | 74.02 | 73.03 |  | **175.12** | 158.09 |  | **1** |

| **Seq** | **ya** | **yb** | **Seq** | **ya** | **yb** | **Seq** | **ya** | **yb** |
| --- | --- | --- | --- | --- | --- | --- | --- | --- |
| **QH** | 238.13 | 266.12 | **QHP** | 335.18 | 363.18 | **QHPG** | 392.20 | 420.20 |
| **QHPGL** | 505.29 | 533.28 | **QHPGLA** | 576.33 | 604.32 | **QHPGLAN** | 690.37 | 718.36 |
| **HP** | 207.12 | 235.12 | **HPG** | 264.15 | 292.14 | **HPGL** | 377.23 | 405.22 |
| **HPGLA** | 448.27 | 476.26 | **HPGLAN** | 562.31 | 590.30 | **HPGLANI** | 675.39 | 703.39 |
| **PG** | 127.09 | 155.08 | **PGL** | 240.17 | **268.17** | **PGLA** | 311.21 | **339.20** |
| **PGLAN** | 425.25 | **453.25** | **PGLANI** | 538.33 | **566.33** | **PGLANIS** | 625.37 | 653.36 |
| **GL** | 143.12 | 171.11 | **GLA** | 214.15 | 242.15 | **GLAN** | 328.20 | 356.19 |
| **GLANI** | 441.28 | 469.28 | **GLANIS** | 528.31 | 556.31 | **GLANISL** | 641.40 | 669.39 |
| **LA** | 157.13 | 185.13 | **LAN** | 271.18 | 299.17 | **LANI** | 384.26 | 412.26 |
| **LANIS** | 471.29 | 499.29 | **LANISL** | 584.38 | 612.37 | **LANISLT** | 685.42 | 713.42 |
| **AN** | 158.09 | 186.09 | **ANI** | 271.18 | 299.17 | **ANIS** | **358.21** | **386.20** |
| **ANISL** | 471.29 | 499.29 | **ANISLT** | 572.34 | 600.34 | **NI** | 200.14 | 228.13 |
| **NIS** | 287.17 | 315.17 | **NISL** | 400.26 | 428.25 | **NISLT** | 501.30 | 529.30 |
| **IS** | 173.13 | 201.12 | **ISL** | 286.21 | 314.21 | **ISLT** | 387.26 | 415.26 |
| **SL** | 173.13 | 201.12 | **SLT** | 274.18 | 302.17 | **LT** | 187.14 | 215.14 |

55. Tb11.01.7960

Match to: **Tb11.01.7960** Score: **41**

**60S ribosomal protein L2, putative; Trypanosoma bruceichr 11Manual**

Nominal mass (M_r_): **31210**; Calculated pI value: **10.76**

NCBI BLAST search of [Tb11.01.7960](http://www.ncbi.nlm.nih.gov/blast/Blast.cgi?ALIGNMENTS=50&ALIGNMENT_VIEW=Pairwise&AUTO_FORMAT=Semiauto&CDD_SEARCH=on&CLIENT=web&COMPOSITION_BASED_STATISTICS=on&DATABASE=nr&DESCRIPTIONS=100&ENTREZ_QUERY=(none)&EXPECT=10&FILTER=L&FORMAT_BLOCK_ON_RESPAGE=None&FORMAT_OBJECT=Alignment&FORMAT_TYPE=HTML&GAPCOSTS=11+1&I_THRESH=0.001&LAYOUT=TwoWindows&MATRIX_NAME=BLOSUM62&NCBI_GI=on&PAGE=Proteins&PROGRAM=blastp&QUERY=MGKTVLTCRKGNGSVYQVHGHKRLGPAKLRILDYAERHGFMRGVVKAIEHEPGRGAPLARVEFRHPYKYRRVKELMVAPEGMFTGQSMLCGVKAPLAIGNVLPLGQITEGCIVCNVEAKVGDRGTLARASGDYCIIISHNTETGRTRLKLPSGQKKTVPSSCRAMIGIVAGGGRIEKPVLKAGNSFYRFRGKRNCWPKVRGVARNPVEHPHGGGNHQHIGHPSTVSRHAPPGQKVGLIAARRTGRIRGSRAVRGAWHPEE&SERVICE=plain&SET_DEFAULTS.x=9&SET_DEFAULTS.y=5&SHOW_OVERVIEW=on&WORD_SIZE=3&END_OF_HTTPGET=Yes) against nr

Unformatted [sequence string](../../../../D:%5CProteomic%20data%5C2010-1-8%5Ccgi%5Cgetseq.pl%3FTBA927_IPI+Tb11%2E01%2E7960+seq) for pasting into other applications

Fixed modifications: MMTS (C),(N-TERM)_iTRAQ,Lysine(K)_iTRAQ

Variable modifications: Oxidation (M)

Cleavage by Trypsin: cuts C-term side of KR unless next residue is P

Sequence Coverage: **10%**

Matched peptides shown in **Bold Red**

**1** MGKTVLTCRK GNGSVYQVHG HKRLGPAKLR **ILDYAER**HGF MRGVVK**AIEH**

**51 EPGR**GAPLAR VEFRHPYKYR RVKELMVAPE GMFTGQSMLC GVKAPLAIGN

**101** VLPLGQITEG CIVCNVEAKV GDRGTLARAS GDYCIIISHN TETGRTRLKL

**151** PSGQKKTVPS SCR**AMIGIVA GGGR**IEKPVL KAGNSFYRFR GKRNCWPKVR

**201** GVARNPVEHP HGGGNHQHIG HPSTVSRHAP PGQKVGLIAA RRTGRIRGSR

**251** AVRGAWHPEE

**Start - End Observed Mr(expt) Mr(calc) Delta Miss Sequence**

**31 - 37 1023.56 1022.56 1022.55 0.01 0 R.ILDYAER.H**  ([Ions score 20](../../../../D:%5CProteomic%20data%5C2010-1-8%5CZQ%5C1339.htm))

**47 - 54 1052.56 1051.56 1051.55 0.00 0 K.AIEHEPGR.G**  ([Ions score 41](../../../../D:%5CProteomic%20data%5C2010-1-8%5CZQ%5C1340.htm))

**164 - 174 1145.65 1144.64 1144.65 -0.01 0 R.AMIGIVAGGGR.I**  ([Ions score 17](../../../../D:%5CProteomic%20data%5C2010-1-8%5CZQ%5C1341.htm))

MS/MS Fragmentation of **AIEHEPGR**
Found in **Tb11.01.7960**, 60S ribosomal protein L2, putative; Trypanosoma bruceichr 11Manual


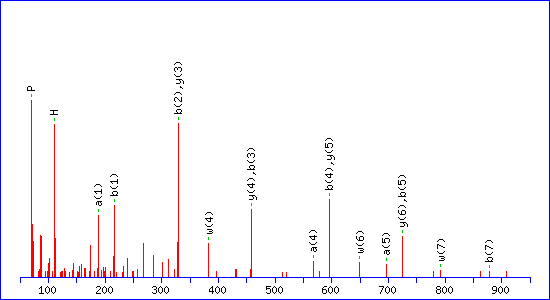


**MONOISOTOPIC mass of neutral peptide Mr(calc):** 1051.55

**Fixed modifications:** MMTS (C),(N-TERM)_iTRAQ,Lysine(K)_iTRAQ

**Ions Score:** 41 **Expect:** 0.013

**Matches (Bold Red):** 18/92 fragment ions using 18 most intense peaks

| **#** | **Immon.** | **a** | **a^0^** | **b** | **b^0^** | **Seq.** | **v** | **w** | **w'** | **y** | **y*** | **y^0^** | **#** |
| --- | --- | --- | --- | --- | --- | --- | --- | --- | --- | --- | --- | --- | --- |
| **1** | 44.05 | **188.15** |  | **216.15** |  | **A** |  |  |  |  |  |  | **8** |
| **2** | 86.10 | 301.24 |  | **329.23** |  | **I** | 779.34 | **792.36** | 806.38 | 837.42 | 820.39 | 819.41 | **7** |
| **3** | 102.05 | 430.28 | 412.27 | **458.27** | 440.26 | **E** | 650.30 | **649.31** |  | **724.34** | 707.31 | 706.33 | **6** |
| **4** | **110.07** | **567.34** | 549.33 | **595.33** | 577.32 | **H** | 513.24 |  |  | **595.29** | 578.27 | 577.28 | **5** |
| **5** | 102.05 | **696.38** | 678.37 | **724.37** | 706.36 | **E** | 384.20 | **383.20** |  | **458.24** | 441.21 | 440.23 | **4** |
| **6** | **70.07** | 793.43 | 775.42 | 821.43 | 803.42 | **P** | 287.15 | 286.15 |  | **329.19** | 312.17 |  | **3** |
| **7** | 30.03 | 850.45 | 832.44 | **878.45** | 860.44 | **G** |  |  |  | 232.14 | 215.11 |  | **2** |
| **8** | 129.11 |  |  |  |  | **R** | 74.02 | 73.03 |  | 175.12 | 158.09 |  | **1** |

| **Seq** | **ya** | **yb** | **Seq** | **ya** | **yb** | **Seq** | **ya** | **yb** |
| --- | --- | --- | --- | --- | --- | --- | --- | --- |
| **IE** | 215.14 | 243.13 | **IEH** | 352.20 | 380.19 | **IEHE** | 481.24 | 509.24 |
| **IEHEP** | 578.29 | 606.29 | **IEHEPG** | 635.31 | 663.31 | **EH** | 239.11 | 267.11 |
| **EHE** | 368.16 | 396.15 | **EHEP** | 465.21 | 493.20 | **EHEPG** | 522.23 | 550.23 |
| **HE** | 239.11 | 267.11 | **HEP** | 336.17 | 364.16 | **HEPG** | 393.19 | 421.18 |
| **EP** | 199.11 | 227.10 | **EPG** | 256.13 | 284.12 | **PG** | 127.09 | 155.08 |

56. Tb11.02.4170

Match to: **Tb11.02.4170** Score: **40**

**40S ribosomal protein S5, putative; Trypanosoma bruceichr 11Manual**

Nominal mass (M_r_): **24057**; Calculated pI value: **10.31**

NCBI BLAST search of [Tb11.02.4170](http://www.ncbi.nlm.nih.gov/blast/Blast.cgi?ALIGNMENTS=50&ALIGNMENT_VIEW=Pairwise&AUTO_FORMAT=Semiauto&CDD_SEARCH=on&CLIENT=web&COMPOSITION_BASED_STATISTICS=on&DATABASE=nr&DESCRIPTIONS=100&ENTREZ_QUERY=(none)&EXPECT=10&FILTER=L&FORMAT_BLOCK_ON_RESPAGE=None&FORMAT_OBJECT=Alignment&FORMAT_TYPE=HTML&GAPCOSTS=11+1&I_THRESH=0.001&LAYOUT=TwoWindows&MATRIX_NAME=BLOSUM62&NCBI_GI=on&PAGE=Proteins&PROGRAM=blastp&QUERY=MSAKAPKLFNKWSYENLQTTEIALNDYITRTPTYVPHSAGRWQKKRFRKARIPIVERLTNGLMFKGRGNGKKLQAVRLVRHTLEIIHLLTDQNPIQVVIDAVSKGAPREDSTRVGAGGVVRRQAVDVSPMRRVNEAIYLMCKGAREAAFRNLKTLPECLADEIVNASKGSSNSYAIKKKDEVERVAKANR&SERVICE=plain&SET_DEFAULTS.x=9&SET_DEFAULTS.y=5&SHOW_OVERVIEW=on&WORD_SIZE=3&END_OF_HTTPGET=Yes) against nr

Unformatted [sequence string](../../../../D:%5CProteomic%20data%5C2010-1-8%5Ccgi%5Cgetseq.pl%3FTBA927_IPI+Tb11%2E02%2E4170+seq) for pasting into other applications

Fixed modifications: MMTS (C),(N-TERM)_iTRAQ,Lysine(K)_iTRAQ

Variable modifications: Oxidation (M)

Cleavage by Trypsin: cuts C-term side of KR unless next residue is P

Sequence Coverage: **5%**

Matched peptides shown in **Bold Red**

**1** MSAKAPKLFN KWSYENLQTT EIALNDYITR **TPTYVPHSAG R**WQKKRFRKA

**51** RIPIVERLTN GLMFKGRGNG KKLQAVRLVR HTLEIIHLLT DQNPIQVVID

**101** AVSKGAPRED STRVGAGGVV RRQAVDVSPM RRVNEAIYLM CKGAREAAFR

**151** NLKTLPECLA DEIVNASKGS SNSYAIKKKD EVERVAKANR

MS/MS Fragmentation of **TPTYVPHSAGR**
Found in **Tb11.02.4170**, 40S ribosomal protein S5, putative; Trypanosoma bruceichr 11Manual


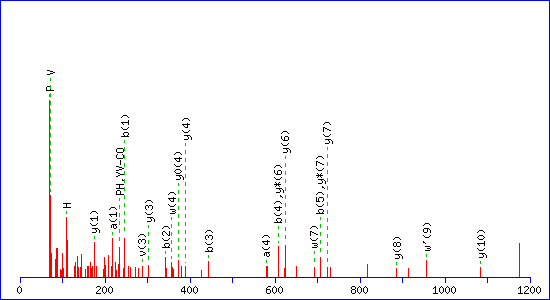


**MONOISOTOPIC mass of neutral peptide Mr(calc):** 1328.70

**Fixed modifications:** MMTS (C),(N-TERM)_iTRAQ,Lysine(K)_iTRAQ

**Ions Score:** 40 **Expect:** 0.014

**Matches (Bold Red):** 27/155 fragment ions using 31 most intense peaks

| **#** | **Immon.** | **a** | **a^0^** | **b** | **b^0^** | **Seq.** | **v** | **w** | **w'** | **y** | **y*** | **y^0^** | **#** |
| --- | --- | --- | --- | --- | --- | --- | --- | --- | --- | --- | --- | --- | --- |
| **1** | 74.06 | **218.16** | 200.15 | **246.16** | 228.15 | **T** |  |  |  |  |  |  | **11** |
| **2** | **70.07** | 315.22 | 297.20 | **343.21** | 325.20 | **P** | 1042.51 | 1041.51 |  | **1084.55** | 1067.53 | 1066.54 | **10** |
| **3** | 74.06 | 416.26 | 398.25 | **444.26** | 426.25 | **T** | 941.46 | 954.48 | **956.46** | 987.50 | 970.47 | 969.49 | **9** |
| **4** | 136.08 | **579.33** | 561.32 | **607.32** | 589.31 | **Y** | 778.40 |  |  | **886.45** | 869.43 | 868.44 | **8** |
| **5** | **72.08** | 678.39 | 660.38 | **706.39** | 688.38 | **V** | 679.33 | **692.35** |  | **723.39** | **706.36** | 705.38 | **7** |
| **6** | **70.07** | 775.45 | 757.44 | 803.44 | 785.43 | **P** | 582.27 | 581.28 |  | **624.32** | **607.29** | 606.31 | **6** |
| **7** | **110.07** | 912.51 | 894.50 | 940.50 | 922.49 | **H** | 445.22 |  |  | 527.27 | 510.24 | 509.26 | **5** |
| **8** | 60.04 | 999.54 | 981.53 | 1027.53 | 1009.52 | **S** | 358.18 | **357.19** |  | **390.21** | 373.18 | **372.20** | **4** |
| **9** | 44.05 | 1070.58 | 1052.56 | 1098.57 | 1080.56 | **A** | **287.15** |  |  | **303.18** | 286.15 |  | **3** |
| **10** | 30.03 | 1127.60 | 1109.59 | 1155.59 | 1137.58 | **G** |  |  |  | 232.14 | 215.11 |  | **2** |
| **11** | 129.11 |  |  |  |  | **R** | 74.02 | 73.03 |  | **175.12** | 158.09 |  | **1** |

| **Seq** | **ya** | **yb** | **Seq** | **ya** | **yb** | **Seq** | **ya** | **yb** |
| --- | --- | --- | --- | --- | --- | --- | --- | --- |
| **PT** | 171.11 | 199.11 | **PTY** | 334.18 | 362.17 | **PTYV** | 433.24 | 461.24 |
| **PTYVP** | 530.30 | 558.29 | **PTYVPH** | 667.36 | 695.35 | **TY** | 237.12 | 265.12 |
| **TYV** | 336.19 | 364.19 | **TYVP** | 433.24 | 461.24 | **TYVPH** | 570.30 | 598.30 |
| **TYVPHS** | 657.34 | 685.33 | **YV** | **235.14** | 263.14 | **YVP** | 332.20 | 360.19 |
| **YVPH** | 469.26 | 497.25 | **YVPHS** | 556.29 | 584.28 | **YVPHSA** | 627.32 | 655.32 |
| **YVPHSAG** | 684.35 | 712.34 | **VP** | 169.13 | 197.13 | **VPH** | 306.19 | 334.19 |
| **VPHS** | 393.22 | 421.22 | **VPHSA** | 464.26 | 492.26 | **VPHSAG** | 521.28 | 549.28 |
| **PH** | 207.12 | **235.12** | **PHS** | 294.16 | 322.15 | **PHSA** | 365.19 | 393.19 |
| **PHSAG** | 422.21 | 450.21 | **HS** | 197.10 | 225.10 | **HSA** | 268.14 | 296.14 |
| **HSAG** | 325.16 | 353.16 | **SA** | 131.08 | 159.08 | **SAG** | 188.10 | 216.10 |
| **AG** | 101.07 | 129.07 |  |  |  |  |  |  |

57. Tb11.02.0170

Match to: **Tb11.02.0170** Score: **40**

**hypothetical protein, conserved; Trypanosoma bruceichr 11Manual**

Nominal mass (M_r_): **68282**; Calculated pI value: **5.90**

NCBI BLAST search of [Tb11.02.0170](http://www.ncbi.nlm.nih.gov/blast/Blast.cgi?ALIGNMENTS=50&ALIGNMENT_VIEW=Pairwise&AUTO_FORMAT=Semiauto&CDD_SEARCH=on&CLIENT=web&COMPOSITION_BASED_STATISTICS=on&DATABASE=nr&DESCRIPTIONS=100&ENTREZ_QUERY=(none)&EXPECT=10&FILTER=L&FORMAT_BLOCK_ON_RESPAGE=None&FORMAT_OBJECT=Alignment&FORMAT_TYPE=HTML&GAPCOSTS=11+1&I_THRESH=0.001&LAYOUT=TwoWindows&MATRIX_NAME=BLOSUM62&NCBI_GI=on&PAGE=Proteins&PROGRAM=blastp&QUERY=MVATGTCNASTVVGVPPCAAASLFSDVMFMKEFGVSGLMTCCLQENACKPTDIGAVRRITLNIPMYVDKVLREKLIEVTNGAHQMISRMEYLPCACDDRRRSPFGFDGTRVLKSAVTTFTVTRVTTNPNKCFVDVSTNFTVDVALPPEGTASTKDEVLFNDIKHFWQQYVERTVLAASEYLLSIALPRIKRSVEAMYDSAYHDLEEAVCRLSVNRGKVLERPEILRIMEKALDAWSHTRRELKHQEMLNHRLEDDFQGARLVAAAAASAAAAAAAAASESQQKEQEYVGVSAVGKAADLPQPRVSVTSAGRRPTTNIDGGKTISGESAAAETKAVDEGSDIIEHNPFFSPTGGRTLSVHQVDEEEKVLNKEALELAAREARLQDISALIESFVTDKGLVDEVTAHALFTKLDVMRRGYVTEQEVVKVLRQLDPLGVYEDRNGAMKMLAAYREALSVGVYNKQGSEKVDSPHSKGLQSGDDMEQVSLMCQPSTFCSVKGGSGFLPVSDELKSVEGNRATLSAVLRRYCNLKEAMQDDAVRARASEMLHKYAFKVRGRLHYDEFCLMMLHVLKDY&SERVICE=plain&SET_DEFAULTS.x=9&SET_DEFAULTS.y=5&SHOW_OVERVIEW=on&WORD_SIZE=3&END_OF_HTTPGET=Yes) against nr

Unformatted [sequence string](../../../../D:%5CProteomic%20data%5C2010-1-8%5Ccgi%5Cgetseq.pl%3FTBA927_IPI+Tb11%2E02%2E0170+seq) for pasting into other applications

Fixed modifications: MMTS (C),(N-TERM)_iTRAQ,Lysine(K)_iTRAQ

Variable modifications: Oxidation (M)

Cleavage by Trypsin: cuts C-term side of KR unless next residue is P

Sequence Coverage: **4%**

Matched peptides shown in **Bold Red**

**1** MVATGTCNAS TVVGVPPCAA ASLFSDVMFM KEFGVSGLMT CCLQENACKP

**51** TDIGAVRRIT LNIPMYVDKV LREKLIEVTN GAHQMISRME YLPCACDDRR

**101** R**SPFGFDGTR** VLKSAVTTFT VTRVTTNPNK CFVDVSTNFT VDVALPPEGT

**151** ASTKDEVLFN DIKHFWQQYV ERTVLAASEY LLSIALPRIK RSVEAMYDSA

**201** YHDLEEAVCR LSVNRGK**VLE RPEILR**IMEK ALDAWSHTRR ELKHQEMLNH

**251** R**LEDDFQGAR** LVAAAAASAA AAAAAAASES QQKEQEYVGV SAVGKAADLP

**301** QPRVSVTSAG RRPTTNIDGG KTISGESAAA ETKAVDEGSD IIEHNPFFSP

**351** TGGRTLSVHQ VDEEEKVLNK EALELAAREA RLQDISALIE SFVTDKGLVD

**401** EVTAHALFTK LDVMRRGYVT EQEVVKVLRQ LDPLGVYEDR NGAMKMLAAY

**451** REALSVGVYN KQGSEKVDSP HSKGLQSGDD MEQVSLMCQP STFCSVKGGS

**501** GFLPVSDELK SVEGNRATLS AVLRRYCNLK EAMQDDAVRA RASEMLHKYA

**551** FKVRGRLHYD EFCLMMLHVL KDY

**Start - End Observed Mr(expt) Mr(calc) Delta Miss Sequence**

**102 - 110 1127.55 1126.54 1126.55 -0.01 0 R.SPFGFDGTR.V**  ([Ions score 4](../../../../D:%5CProteomic%20data%5C2010-1-8%5CZQ%5C1345.htm))

**218 - 226 1268.67 1267.66 1267.77 -0.11 0 K.VLERPEILR.I**  ([Ions score 12](../../../../D:%5CProteomic%20data%5C2010-1-8%5CZQ%5C1347.htm))

**252 - 260 1194.59 1193.58 1193.58 0.00 0 R.LEDDFQGAR.L**  ([Ions score 40](../../../../D:%5CProteomic%20data%5C2010-1-8%5CZQ%5C1346.htm))

MS/MS Fragmentation of **LEDDFQGAR**
Found in **Tb11.02.0170**, hypothetical protein, conserved; Trypanosoma bruceichr 11Manual


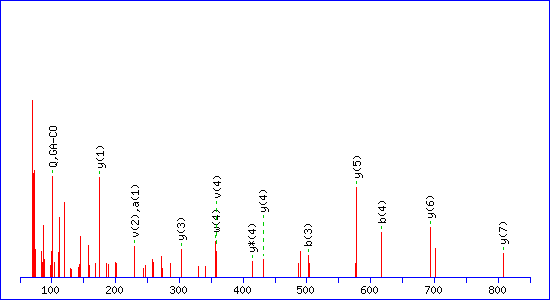


**MONOISOTOPIC mass of neutral peptide Mr(calc):** 1193.58

**Fixed modifications:** MMTS (C),(N-TERM)_iTRAQ,Lysine(K)_iTRAQ

**Ions Score:** 40 **Expect:** 0.017

**Matches (Bold Red):** 15/116 fragment ions using 17 most intense peaks

| **#** | **Immon.** | **a** | **a*** | **a^0^** | **b** | **b*** | **b^0^** | **Seq.** | **v** | **w** | **y** | **y*** | **y^0^** | **#** |
| --- | --- | --- | --- | --- | --- | --- | --- | --- | --- | --- | --- | --- | --- | --- |
| **1** | 86.10 | **230.20** |  |  | 258.19 |  |  | **L** |  |  |  |  |  | **9** |
| **2** | 102.05 | 359.24 |  | 341.23 | 387.24 |  | 369.23 | **E** | 863.36 | 862.37 | 937.40 | 920.37 | 919.39 | **8** |
| **3** | 88.04 | 474.27 |  | 456.26 | **502.26** |  | 484.25 | **D** | 748.34 | 747.34 | **808.36** | 791.33 | 790.35 | **7** |
| **4** | 88.04 | 589.30 |  | 571.28 | **617.29** |  | 599.28 | **D** | 633.31 | 632.32 | **693.33** | 676.30 | 675.32 | **6** |
| **5** | 120.08 | 736.36 |  | 718.35 | 764.36 |  | 746.35 | **F** | 486.24 |  | **578.30** | 561.28 |  | **5** |
| **6** | **101.07** | 864.42 | 847.40 | 846.41 | 892.42 | 875.39 | 874.41 | **Q** | **358.18** | **357.19** | **431.24** | **414.21** |  | **4** |
| **7** | 30.03 | 921.44 | 904.42 | 903.43 | 949.44 | 932.41 | 931.43 | **G** |  |  | **303.18** | 286.15 |  | **3** |
| **8** | 44.05 | 992.48 | 975.45 | 974.47 | 1020.48 | 1003.45 | 1002.47 | **A** | **230.12** |  | 246.16 | 229.13 |  | **2** |
| **9** | 129.11 |  |  |  |  |  |  | **R** | 74.02 | 73.03 | **175.12** | 158.09 |  | **1** |

| **Seq** | **ya** | **yb** | **Seq** | **ya** | **yb** | **Seq** | **ya** | **yb** |
| --- | --- | --- | --- | --- | --- | --- | --- | --- |
| **ED** | 217.08 | 245.08 | **EDD** | 332.11 | 360.10 | **EDDF** | 479.18 | 507.17 |
| **EDDFQ** | 607.24 | 635.23 | **EDDFQG** | 664.26 | 692.25 | **DD** | 203.07 | 231.06 |
| **DDF** | 350.13 | 378.13 | **DDFQ** | 478.19 | 506.19 | **DDFQG** | 535.21 | 563.21 |
| **DDFQGA** | 606.25 | 634.25 | **DF** | 235.11 | 263.10 | **DFQ** | 363.17 | 391.16 |
| **DFQG** | 420.19 | 448.18 | **DFQGA** | 491.22 | 519.22 | **FQ** | 248.14 | 276.13 |
| **FQG** | 305.16 | 333.16 | **FQGA** | 376.20 | 404.19 | **QG** | 158.09 | 186.09 |
| **QGA** | 229.13 | 257.12 | **GA** | **101.07** | 129.07 |  |  |  |

58. Tb10.6k15.0710

Match to: **Tb10.6k15.0710** Score: **40**

**hypothetical protein, conserved; Trypanosoma bruceichr 10Manual**

Nominal mass (M_r_): **42088**; Calculated pI value: **7.72**

NCBI BLAST search of [Tb10.6k15.0710](http://www.ncbi.nlm.nih.gov/blast/Blast.cgi?ALIGNMENTS=50&ALIGNMENT_VIEW=Pairwise&AUTO_FORMAT=Semiauto&CDD_SEARCH=on&CLIENT=web&COMPOSITION_BASED_STATISTICS=on&DATABASE=nr&DESCRIPTIONS=100&ENTREZ_QUERY=(none)&EXPECT=10&FILTER=L&FORMAT_BLOCK_ON_RESPAGE=None&FORMAT_OBJECT=Alignment&FORMAT_TYPE=HTML&GAPCOSTS=11+1&I_THRESH=0.001&LAYOUT=TwoWindows&MATRIX_NAME=BLOSUM62&NCBI_GI=on&PAGE=Proteins&PROGRAM=blastp&QUERY=MSGNSQTARALTRAASADGFNPSNTGAFSPDVEDFEALETQARAALEDLAANGSRNEALTVEYMCILRTYRQACERRAQYVEADLVQRVLRYMRLDEETRHVCGLTELQRQERDAIETLHREEVQEFHHAWNMRIDELEEEQLRLETALIERQNAELQSFYEEVNSLNPHAAKCSRGLLYARAVEHILASQREYVRAHKKKKEADTIEARDAERFVQAKVELLERREAMMRQKHEQERHVLEVKAKRRRAEMEAARKRELYILLRRYLNAQRELELHQNIVRSKTGTILLKHASNTKGNTSGTAVLVESAESGAFGIRARKQHLDNLVDSCTLPKIGTKPK&SERVICE=plain&SET_DEFAULTS.x=9&SET_DEFAULTS.y=5&SHOW_OVERVIEW=on&WORD_SIZE=3&END_OF_HTTPGET=Yes) against nr

Unformatted [sequence string](../../../../D:%5CProteomic%20data%5C2010-1-8%5Ccgi%5Cgetseq.pl%3FTBA927_IPI+Tb10%2E6k15%2E0710+seq) for pasting into other applications

Fixed modifications: MMTS (C),(N-TERM)_iTRAQ,Lysine(K)_iTRAQ

Variable modifications: Oxidation (M)

Cleavage by Trypsin: cuts C-term side of KR unless next residue is P

Sequence Coverage: **6%**

Matched peptides shown in **Bold Red**

**1** MSGNSQTARA LTRAASADGF NPSNTGAFSP DVEDFEALET QAR**AALEDLA**

**51 ANGSR**NEALT VEYMCILRTY RQACERRAQY VEADLVQRVL RYMRLDEETR

**101** HVCGLTELQR QERDAIETLH REEVQEFHHA WNMRIDELEE EQLRLETALI

**151** ERQNAELQSF YEEVNSLNPH AAKCSRGLLY ARAVEHILAS QREYVRAHKK

**201** KKEADTIEAR DAERFVQAKV ELLERREAMM RQKHEQERHV LEVKAKRRRA

**251** EMEAARKREL YILLRRYLNA QR**ELELHQNI VR**SKTGTILL KHASNTKGNT

**301** SGTAVLVESA ESGAFGIRAR KQHLDNLVDS CTLPKIGTKP K

**Start - End Observed Mr(expt) Mr(calc) Delta Miss Sequence**

**44 - 55 1331.80 1330.79 1330.70 0.10 0 R.AALEDLAANGSR.N**  ([Ions score 3](../../../../D:%5CProteomic%20data%5C2010-1-8%5CZQ%5C1360.htm))

**273 - 282 1394.78 1393.77 1393.78 -0.01 0 R.ELELHQNIVR.S**  ([Ions score 40](../../../../D:%5CProteomic%20data%5C2010-1-8%5CZQ%5C1361.htm))

MS/MS Fragmentation of **ELELHQNIVR**
Found in **Tb10.6k15.0710**, hypothetical protein, conserved; Trypanosoma bruceichr 10Manual


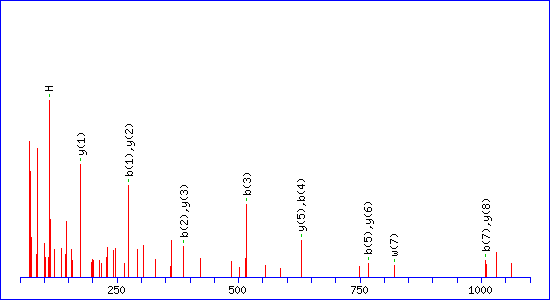


**MONOISOTOPIC mass of neutral peptide Mr(calc):** 1393.78

**Fixed modifications:** MMTS (C),(N-TERM)_iTRAQ,Lysine(K)_iTRAQ

**Ions Score:** 40 **Expect:** 0.015

**Matches (Bold Red):** 14/137 fragment ions using 13 most intense peaks

| **#** | **Immon.** | **a** | **a*** | **a^0^** | **b** | **b*** | **b^0^** | **Seq.** | **v** | **w** | **w'** | **y** | **y*** | **y^0^** | **#** |
| --- | --- | --- | --- | --- | --- | --- | --- | --- | --- | --- | --- | --- | --- | --- | --- |
| **1** | 102.05 | 246.16 |  | 228.15 | **274.15** |  | 256.14 | **E** |  |  |  |  |  |  | **10** |
| **2** | 86.10 | 359.24 |  | 341.23 | **387.24** |  | 369.23 | **L** | 1063.56 | 1062.57 |  | 1121.64 | 1104.62 | 1103.63 | **9** |
| **3** | 102.05 | 488.28 |  | 470.27 | **516.28** |  | 498.27 | **E** | 934.52 | 933.53 |  | **1008.56** | 991.53 | 990.55 | **8** |
| **4** | 86.10 | 601.37 |  | 583.36 | **629.36** |  | 611.35 | **L** | 821.44 | **820.44** |  | 879.52 | 862.49 |  | **7** |
| **5** | **110.07** | 738.43 |  | 720.42 | **766.42** |  | 748.41 | **H** | 684.38 |  |  | **766.43** | 749.41 |  | **6** |
| **6** | 101.07 | 866.49 | 849.46 | 848.48 | 894.48 | 877.45 | 876.47 | **Q** | 556.32 | 555.32 |  | **629.37** | 612.35 |  | **5** |
| **7** | 87.06 | 980.53 | 963.50 | 962.52 | **1008.52** | 991.50 | 990.51 | **N** | 442.28 | 441.28 |  | 501.31 | 484.29 |  | **4** |
| **8** | 86.10 | 1093.61 | 1076.59 | 1075.60 | 1121.61 | 1104.58 | 1103.60 | **I** | 329.19 | 342.21 | 356.23 | **387.27** | 370.24 |  | **3** |
| **9** | 72.08 | 1192.68 | 1175.65 | 1174.67 | 1220.68 | 1203.65 | 1202.67 | **V** | 230.12 | 243.15 |  | **274.19** | 257.16 |  | **2** |
| **10** | 129.11 |  |  |  |  |  |  | **R** | 74.02 | 73.03 |  | **175.12** | 158.09 |  | **1** |

| **Seq** | **ya** | **yb** | **Seq** | **ya** | **yb** | **Seq** | **ya** | **yb** |
| --- | --- | --- | --- | --- | --- | --- | --- | --- |
| **LE** | 215.14 | 243.13 | **LEL** | 328.22 | 356.22 | **LELH** | 465.28 | 493.28 |
| **LELHQ** | 593.34 | 621.34 | **EL** | 215.14 | 243.13 | **ELH** | 352.20 | 380.19 |
| **ELHQ** | 480.26 | 508.25 | **ELHQN** | 594.30 | 622.29 | **LH** | 223.16 | 251.15 |
| **LHQ** | 351.21 | 379.21 | **LHQN** | 465.26 | 493.25 | **LHQNI** | 578.34 | 606.34 |
| **LHQNIV** | 677.41 | 705.40 | **HQ** | 238.13 | 266.12 | **HQN** | 352.17 | 380.17 |
| **HQNI** | 465.26 | 493.25 | **HQNIV** | 564.33 | 592.32 | **QN** | 215.11 | 243.11 |
| **QNI** | 328.20 | 356.19 | **QNIV** | 427.27 | 455.26 | **NI** | 200.14 | 228.13 |
| **NIV** | 299.21 | 327.20 | **IV** | 185.16 | 213.16 |  |  |  |

59. Tb11.02.2490

Match to: **Tb11.02.2490** Score: **39**

**hypothetical protein, conserved; Trypanosoma bruceichr 11Manual**

Nominal mass (M_r_): **34562**; Calculated pI value: **9.35**

NCBI BLAST search of [Tb11.02.2490](http://www.ncbi.nlm.nih.gov/blast/Blast.cgi?ALIGNMENTS=50&ALIGNMENT_VIEW=Pairwise&AUTO_FORMAT=Semiauto&CDD_SEARCH=on&CLIENT=web&COMPOSITION_BASED_STATISTICS=on&DATABASE=nr&DESCRIPTIONS=100&ENTREZ_QUERY=(none)&EXPECT=10&FILTER=L&FORMAT_BLOCK_ON_RESPAGE=None&FORMAT_OBJECT=Alignment&FORMAT_TYPE=HTML&GAPCOSTS=11+1&I_THRESH=0.001&LAYOUT=TwoWindows&MATRIX_NAME=BLOSUM62&NCBI_GI=on&PAGE=Proteins&PROGRAM=blastp&QUERY=MSGESVFNLIEPDVESVQPQPLRKVKLGKGTAAPPTASTFGFHGTSAVVANVGGENTEPSVHPAKKPTGTFGREASSSVNPSKFLKKNEGVGTASRGVPIVDATKFVKAEKDASKRKDDVPNRYDKPVMGVKTEKNYVVANAVENVLALPTKCIPTPMNRAVDRADFGKVPKYLKEVKADIEERHALVERLKAAKREAEERWSELSGEELEQLKQGLQRRWDSLNKDYQSRGFSKLQTPSQKAQHEAVGKELNAVEFAMQKLSRAHVFVYDDRN&SERVICE=plain&SET_DEFAULTS.x=9&SET_DEFAULTS.y=5&SHOW_OVERVIEW=on&WORD_SIZE=3&END_OF_HTTPGET=Yes) against nr

Unformatted [sequence string](../../../../D:%5CProteomic%20data%5C2010-1-8%5Ccgi%5Cgetseq.pl%3FTBA927_IPI+Tb11%2E02%2E2490+seq) for pasting into other applications

Fixed modifications: MMTS (C),(N-TERM)_iTRAQ,Lysine(K)_iTRAQ

Variable modifications: Oxidation (M)

Cleavage by Trypsin: cuts C-term side of KR unless next residue is P

Sequence Coverage: **8%**

Matched peptides shown in **Bold Red**

**1** MSGESVFNLI EPDVESVQPQ PLRKVKLGKG TAAPPTASTF GFHGTSAVVA

**51** NVGGENTEPS VHPAKKPTGT FGREASSSVN PSKFLKKNEG VGTASR**GVPI**

**101 VDATK**FVKAE KDASKRKDDV PNRYDKPVMG VKTEKNYVVA NAVENVLALP

**151** TK**CIPTPMNR** AVDRADFGKV PKYLKEVKAD IEERHALVER LKAAKREAEE

**201** RWSELSGEEL EQLKQGLQRR WDSLNKDYQS RGFSK**LQTPS QK**AQHEAVGK

**251** ELNAVEFAMQ KLSRAHVFVY DDRN

**Start - End Observed Mr(expt) Mr(calc) Delta Miss Sequence**

**97 - 105 1187.71 1186.70 1186.63 0.07 0 R.GVPIVDATK.F**  ([Ions score 39](../../../../D:%5CProteomic%20data%5C2010-1-8%5CZQ%5C1365.htm))

**153 - 160 1121.52 1120.51 1120.53 -0.02 0 K.CIPTPMNR.A**  ([Ions score 13](../../../../D:%5CProteomic%20data%5C2010-1-8%5CZQ%5C1364.htm))

**236 - 242 1089.62 1088.61 1088.55 0.06 0 K.LQTPSQK.A**  ([Ions score 26](../../../../D:%5CProteomic%20data%5C2010-1-8%5CZQ%5C1363.htm))

MS/MS Fragmentation of **GVPIVDATK**
Found in **Tb11.02.2490**, hypothetical protein, conserved; Trypanosoma bruceichr 11Manual


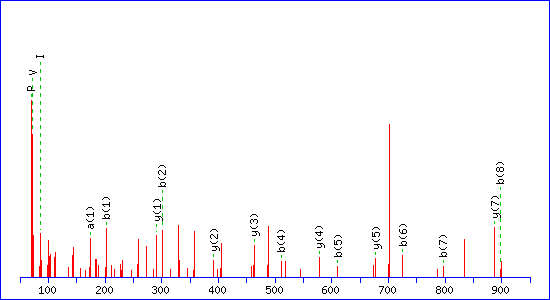


**MONOISOTOPIC mass of neutral peptide Mr(calc):** 1186.63

**Fixed modifications:** MMTS (C),(N-TERM)_iTRAQ,Lysine(K)_iTRAQ

**Ions Score:** 39 **Expect:** 0.011

**Matches (Bold Red):** 18/96 fragment ions using 30 most intense peaks

| **#** | **Immon.** | **a** | **a^0^** | **b** | **b^0^** | **Seq.** | **y** | **y*** | **y^0^** | **#** |
| --- | --- | --- | --- | --- | --- | --- | --- | --- | --- | --- |
| **1** | 30.03 | **174.14** |  | **202.13** |  | **G** |  |  |  | **9** |
| **2** | **72.08** | 273.20 |  | **301.20** |  | **V** | 986.51 | 969.48 | 968.50 | **8** |
| **3** | **70.07** | 370.26 |  | 398.25 |  | **P** | **887.44** | 870.42 | 869.43 | **7** |
| **4** | **86.10** | 483.34 |  | **511.34** |  | **I** | 790.39 | 773.36 | 772.38 | **6** |
| **5** | **72.08** | 582.41 |  | **610.40** |  | **V** | **677.31** | 660.28 | 659.29 | **5** |
| **6** | 88.04 | 697.44 | 679.43 | **725.43** | 707.42 | **D** | **578.24** | 561.21 | 560.23 | **4** |
| **7** | 44.05 | 768.47 | 750.46 | **796.47** | 778.46 | **A** | **463.21** | 446.18 | 445.20 | **3** |
| **8** | 74.06 | 869.52 | 851.51 | **897.52** | 879.51 | **T** | **392.17** | 375.15 | 374.16 | **2** |
| **9** | 245.12 |  |  |  |  | **K** | **291.13** | 274.10 |  | **1** |
[truncated: 152,501 more chars]
